# Acorane sesquiterpenes from the deep-sea derived *Penicillium bilaiae* fungus with anti-neuroinflammatory effects

**DOI:** 10.3389/fchem.2022.1036212

**Published:** 2022-11-23

**Authors:** Wenfang Zhang, Qingyu Meng, Jingshuai Wu, Wei Cheng, Dong Liu, Jian Huang, Aili Fan, Jing Xu, Wenhan Lin

**Affiliations:** ^1^ State Key Laboratory of Natural and Biomimetic Drugs, Institute of Ocean Research, Peking University, Beijing, China; ^2^ School of Chemical Engineering and Technology, Hainan University, Haikou, China; ^3^ Ningbo Institute of Marine Medicines, Peking University, Ningbo, China

**Keywords:** fungus, *Penicillium bilaiae*, sesquiterpene, bilaiaeacorenols A–R, structure elucidation, anti-neuroinflammation

## Abstract

Acorane-type sesquiterpenes comprise a unique class of natural products with a range of pharmaceutical effects. Genome sequencing and gene annotation, along with qRT-PCR detection, demonstrate that the deep-sea derived *Penicillium bilaiae* F-28 fungus shows potential to produce acorane sesquiterpenes. Chromatographic manipulation resulted in the isolation of 20 acorane sesquiterpenes from the large-scale fermented fungal strain. Their structures were established by the interpretation of spectroscopic data, together with X-ray diffraction, chemical conversion, and ECD data for configurational assignments. A total of 18 new sesquiterpenes, namely, bilaiaeacorenols A–R (**1–18**), were identified. Bilaiaeacorenols A and B represent structurally unique tricyclic acoranes. Compound **18** exhibited efficient reduction against NO production in LPS-induced BV-2 macrophages in a dose-dependent manner, and it abolished LPS-induced NF-κB in the nucleus of BV-2 microglial cells. In addition, marked reductions of iNOS and COX-2 in protein and mRNA levels were observed. This study extends the chemical diversity of acorane-type sesquiterpenoids and suggests that compound **18** is a promising lead for anti-neuroinflammation.

## Introduction

Acorane-type sesquiterpenes feature a spiro[4.5]decane core with an isopropyl unit at C-1 and dimethyl substitution at C-4 and C-8, which markedly differs from other types of the sesquiterpene family (Liu et al., 2015; Zhang et al., 2017; Guo et al., 2020). Hitherto, less than 30 acorane-based sesquiterpenes have been reported from plants and microorganisms. Acrorans in plants are characteristic of the volatile metabolites which play crucial roles as biocontrol and biostimulant agents and are also considered the chemotaxonomic markers of the plant (Zhang et al., 2020). Biogenetically, acrorans are synthesized from farnesyl diphosphate (FPP) as a common precursor by catalysis using sesquiterpene synthases, of which EfCAS in the plant catalyzes the cyclization of FFP to afford a spiro[4.5]decane core such as eupho-acorenols A and B (Zhu et al., 2021). Enzymatic catalysis to generate the acorane core in fungi is also documented (Bian et al., 2018). Due to the unique molecular scaffolds, acrorans exhibit a wide range of bioactivities. Chermebilaene A and its hydrolyzed product from a marine-derived fungus show significant activity against pathogenic bacteria (Meng et al., 2020), daphneaines from a plant show inhibitory effects against nitric oxide (NO) production in lipopolysaccharide (LPS)-induced RAW 264.7 macrophages (Guo et al., 2020), and rhodocoranes possess various cytotoxic and antifungal effects (Sandargo et al., 2019).

## Experiment

### General experimental procedures

Optical rotations were recorded on an AUTOPOL III Automatic Polarimeter, and IR spectra were performed on a Thermo Nicolet Nexus 470 FT-IR spectrometer. NMR spectra were measured on a Bruker Avance-400 NMR spectrometer with TMS as the internal standard. HRESIMS data were recorded on a Bruker APEX IV 70 eV FT-MS spectrometer. ESIMS spectra were detected on a Finnigan MAT-95 mass spectrometer. Silica gel (200–300 mesh) and HF254 silica gel for used for TLC were purchased from Qingdao Marine Chemistry Co., Ltd., while Sephadex LH-20 (18–110 μm; Pharmacia Co., Ltd.) and ODS (50 μm, YMC, Milford, MA) were used for separation. HPLC was performed on an Alltech instrument (426-HPLC pump) equipped with a UV detector. X-ray data were collected on a Bruker SMART APEX-II DUO instrument. Dulbecco’s modified Eagle’s medium (DMEM) and fetal bovine serum (FBS) were purchased from HyClone (Waltham, United States). 3-(4,5-Dimethylthiazol-2-yl)-2,5-diphenyltetrazolium bromide (MTT) and lipopolysaccharide (LPS) (*Escherichia coli* 055: B5) were supplied by Sigma Chemical Co., (St Louis, MO, United States). Griess reagent (ExCell Bio) and primary antibodies were supplied by Cell Signaling Technology (Danvers, United States).

### Fungal material and fermentation

The fungal strain *Penicillium bilaiae* F-28 was collected from deep-sea sediment (GPS 27.90 W, 6.43 S, depth of 5,610 m) in the South Atlantic Ocean. The DNA was collected and amplified by the ITS primers (ITS4 and ITS5). The ITS sequence (773 bp) was deposited in GenBank (accession number LN901118.1). Based on the BLAST search, the fungal strain was identical to *P. bilaiae*. Then, fermentation was performed in rice (80 g for each, 120 Fernbach flasks, 500 ml) with distilled H_2_O (80 ml for each), which was allowed to soak overnight. Each flask was seeded with 2.0 ml of the spore inoculum (10^7^/ml) and incubated at 25°C for 35 days. The EtOAc solvent was used for the extraction of the fermented material.

### Genome sequencing and analysis

Genome sequencing of *P. bilaiae* F-28 was detected by using an Illumina HiSeq 2000 system. The sequence was constructed on SPAdes version 3.5.0 (http://cab.spbu.ru/software/spades/), generating 160 scaffolds (ca. 36.7 Mb). Gene annotation was undertaken by Prokka (https://github.com/tseemann/prokka). Analysis of the genome sequence by anti-SMASH and correlation revealed nine isoprenoid biosyn-C1 superfamily terpenoid cyclase genes, which were then compared and annotated to the protein sequences in NCBI.

### Quantitative RT-PCR for terpenoid cyclase genes

The expression levels of nine terpenoid cyclase genes were detected by qRT-PCR. The total RNA of *P. bilaiae* F-28 in the rice culture medium was obtained. The synthesis of cDNA was performed with the guidance of the manufacturer’s instruction [1 μg of total RNA (20 μl) and TransScriptIIAll-in-One First-Strand cDNA Synthesis Super Mix (Transgene) for qPCR]. A measure of 0.4 μl of cDNA, together with the primer (10 μM) and reverse primer (10 μM), and 10 μl 2× TransStart Top Green qPCR SuperMix (Transgene) were supplied for RT-PCR in ddH_2_O (20 μl). Optimized PCR conditions were 94°C/5 min; 40 cycles of 94°C/20 s; 54°C/20 s; and 72°C/20 s; 72°C/5 min. Then, 4 μl of 6×DNA Loading buffer was added to the PCR product, and 8 μl was taken for agarose electrophoresis detection. The bands were observed under 300 nm UV and photographed. An internal reference gene is β-actin.

### UPLC-electrospray ionization-MS/MS data and molecular networking

The EtOAc extract of the cultured fungus was analyzed on a Thermo Vanquish F UPLC system coupled with the Thermo Q Exactive HF-X mass spectrometer equipped with an electrospray ionization (ESI) source operating with positive polarity at a mass range of *m/z* 50–500 Da. The 0.1 mg/ml MeOH solution was filtered through a 0.2-mm PTFE syringe filter (Carl Roth) and then injected (injection volume: 5.0 μl) into the system that was equipped with an Acquity UPLC HSS T3 column (high-strength silica C_18_, 1.8 μm, 100 mm × 2.1 mm i. d., Waters) operating at 40°C. Separation was achieved with a binary LC solvent system using mobile phase A [99.9% H_2_O/0.1% formic acid (ULC/MS grade)] and B [MeCN (ULC/MS grade)], pumped at a rate of 0.3 ml/min with the following gradients: 0–1 min, 100% A; 1–3 min, 100%–95% A; 3–20 min, 95%–0% A; 20–25 min, 0% A; 25–25.5, 0–100% A; and 25.5–30 min, 100% A. TIC and EIC spectra were extracted and analyzed on Thermo Xcalibur Qual Browser software. Instrumental parameters were set as follows: source voltage 3.5 kV, lens 1 voltage −10 V, capillary temperature 320°C, gate lens voltage −40 V, capillary voltage 40 V, and tube lens voltage 100 V. The CID parameters were set as follows: CE at 20% of the maximum and an activation time of 20 ms. Tandem mass spectra arising from UPLC-MS/MS were annotated in the Advanced Mass Spectral Database (https://www.mzcloud.org) and analyzed by Compound Discoverer 3.1.0.305 software. Subsequently, UPLC-MS/MS data were further analyzed using the GNPS platform (http://gnps.ucsd.edu). The MS/MS data were converted to mzXML format with MS-Convert and then uploaded on the GNPS. Parameters for molecular network generation were set as the precursor ion mass tolerance of 0.05 Da, product ion tolerance of 0.05 Da, and removing fragment ions below 10 counts from the MS/MS spectra. Molecular networks were generated using four minimum matched peaks and a cosine score of 0.70. Edges between two nodes were kept in the network if each of the nodes appeared in each other’s respective top 10 most similar nodes. The maximum size of a molecular family was set to 100, and the lowest scoring edges were removed from molecular families until the molecular family size was below this threshold. The spectra in the network were then searched against GNPS spectral libraries. The library spectra were filtered in the same manner as the input data. All matches kept between the network spectra and library spectra were required to have a score above 0.7 and at least six matched peaks. Data were visualized by Cytoscape 3.8.0 software.

### Extraction and isolation

The fermented fungus was extracted by EtOAc (3 L × 2 L), which was concentrated under reduced pressure to obtain the residue (38 g). The EtOAc extract was partitioned between MeOH-H_2_O (1:10) and petroleum ether (PE), and the MeOH layer was collected. The MeOH fraction (20 g) was chromatographed upon a silica gel (200–300 mesh) vacuum liquid column and eluted using CH_2_Cl_2_-MeOH (from 15:1 to 0:1, v/v) to collect nine fractions (F1–F9). The ^1^H NMR spectra of F3 and F5 fractions showed the resonances featured terpene analogs. F3 (0.32 g) was purified by an RP-C18 column with a mobile phase of MeOH-H_2_O (55:45, v/v) to yield adametacorenol A (160 mg). F5 (0.85 g) was fractionated upon an RP-C18 column and eluted using MeOH-H_2_O (1:4, v/v) to yield subfractions of F51–F56. F51 (260 mg) was subjected to a Sephadex LH-20 column and eluted with MeOH to purify compounds **8** (5.6 mg) and **16** (3.3 mg). F52 (90 mg) was fractionated using a semipreparative RP-C18 HPLC column with MeCN-H_2_O (30:70, v/v) as a mobile phase to yield compounds **6** (1.2 mg), **5** (1.0 mg), **13** (2 mg), and **18** (1.6 mg). F53 (42 mg) followed the same protocol as for F52 on a semipreparative RP-C18 HPLC column with MeOH-H_2_O (1:3, v/v) to obtain compounds **17** (0.8 mg), **14** (0.6 mg), **11** (1.2 mg), and **7** (1.0 mg). F54 (400 mg) was separated using a semipreparative RP-C18 HPLC column with MeCN-H_2_O (1:1, v/v) to collect compounds **9** (1 mg), **1** (1.1 mg), **10** (0.8 mg), **4** (4 mg), **3** (2.5 mg), **15** (4 mg), adametacorenol B (0.8 mg), **12** (1.0 mg), and **2** (3.6 mg).

### Compound characterization

Bilaiaeacorenol A (**1**): colorless monoclinic crystals (acetone); mp. 106–108°; (*α*) -120 (*c* 0.1, MeOH); UV (MeOH) λ_max_ 202 nm; IR (KBr) ν_max_ 3,306, 2,929, and 1,456 cm^−1^; ^1^H and ^13^C NMR data (DMSO-*d*_6_), see Supplementary Tables S3, S5; HRESIMS *m/z* 275.1623 [M + Na]^+^ (calcd for C_15_H_24_O_3_Na, 275.1623) (Supplementary Figures S1–S9); and Flack parameter: 0.00 (6).

Bilaiaeacorenol B (**2**): colorless monoclinic crystals (acetone); mp. 108–110°; (*α*) -12 (*c* 0.1, MeOH); UV (MeOH) λ_max_ 200 nm; IR (KBr) ν_max_ 3,348, 2,923, 1,456, and 1,374 cm^−1^; ^1^H and ^13^C NMR data (DMSO-*d*_6_), see Supplementary Tables S3, S5; HRESIMS *m/z* 253.1801 [M + H]^+^ (calcd for C_15_H_25_O_3_, 253.1804) (Supplementary Figures S10–S18); and Flack parameter: 0.05 (9).

Bilaiaeacorenol C (**3**): colorless oil; [α] -40 (*c* 0.1, MeOH); UV (MeOH) λ_max_ 200 nm; IR (KBr) ν_max_ 3,335, 2,932, and 1,679 cm^−1^; ^1^H and ^13^C NMR data (DMSO-*d*_6_), see Supplementary Tables S3, S5; and HRESIMS *m/z* 335.1833 [M +Na]+(calcd for C_17_H_28_O_5_Na, 335.1834) (Supplementary Figures S19–S27).

Bilaiaeacorenol D (**4**): colorless oil; [α] –30 (*c* 0.1, MeOH); UV (MeOH) λ_max_ 202 nm; IR (KBr) ν_max_ 3,360, 2,922, 1732, 1,667, 1,385, and 1,249 cm^−1^; ^1^H and ^13^C NMR data (DMSO-*d*_6_), see Supplementary Tables S3, S5; and HRESIMS *m/z* 319.1888 [M + Na]^+^ (calcd for C_17_H_28_O_4_Na, 319.1885) (Supplementary Figures S28–S36).

Bilaiaeacorenol E (**5**): colorless oil; [α] -8 (*c* 0.1, MeOH); UV (MeOH) λ_max_ 202 nm; IR (KBr) ν_max_ 3358, 1,648, and 1,321 cm^−1^; ^1^H and ^13^C NMR data (DMSO-*d*_6_), see Supplementary Tables S3, S5; and HRESIMS *m/z* 219.1749 [M–HO]+(calcd for C_15_H_23_O_2_, 219.1749) (Supplementary Figures S37–S45).

Bilaiaeacorenol F (**6**): colorless oil; [α] -20 (*c* 0.1, MeOH); UV (MeOH) λ_max_ 201 nm; IR (KBr) ν_max_ 3,400 and 1,388 cm^−1^; ^1^H and ^13^C NMR data (DMSO-*d*_6_), see Supplementary Tables S3, S5; and HRESIMS *m/z* 237.1852 [M + H]^+^ (calcd for C_15_H_25_O_2_, 237.1855) (Supplementary Figures S46–S54).

Bilaiaeacorenol G (**7**): colorless oil; [α] -20 (*c* 0.1, MeOH); UV (MeOH) λ_max_ 201 nm; IR (KBr) ν_max_ 3,312 and 1,643 cm^−1^; ^1^H and ^13^C NMR data (DMSO-*d*_6_), see Supplementary Tables S3, S5; and HRESIMS *m/z* 275.1621 [M + Na]^+^ (calcd for C_15_H_24_O_3_Na, 275.1623) (Supplementary Figures S55–S63).

Bilaiaeacorenol H (**8**): colorless monoclinic crystals (acetone); mp. 113–115°; [α] -20 (*c* 0.1, MeOH); UV (MeOH) λ_max_ 201 nm; IR (KBr) ν_max_ 3,312 and 1,643 cm^−1^; ^1^H and ^13^C NMR data (DMSO-*d*_6_), see Supplementary Tables S4, S5; HRESIMS *m/z* 275.1625 [M + Na]+(calcd for C_15_H_24_O_3_Na, 275.1623) (Supplementary Figures S64–S72); and Flack parameter: 0.01 (10).

Bilaiaeacorenol I (**9**): colorless oil; [α] -40 (*c* 0.1, MeOH); UV (MeOH) λ_max_ 201 nm; IR (KBr) ν_max_ 3,375, 1710, 1,374, and 1,260 cm^−1^; ^1^H and ^13^C NMR data (DMSO-*d*_6_), see Supplementary Tables S4, S5; and HRESIMS *m/z* 317.1724 [M + Na]+(calcd for C_17_H_26_O_4_Na, 317.1729) (Supplementary Figures S73–S81).

Bilaiaeacorenol J (**10**): colorless oil; [α] -20 (*c* 0.1, MeOH); UV (MeOH) λ_max_ 201 nm; IR (KBr) ν_max_ 3,380, 2,928, 2,872, 1734, 1375, and 1247 cm^−1^; ^1^H and ^13^C NMR data (DMSO-*d*_6_), see Supplementary Tables S4, S5; and HRESIMS *m/z* 317.1723 [M + Na]+(calcd for C_17_ H_26_O_4_Na, 317.1729) (Supplementary Figures S82–S90).

Bilaiaeacorenol K (**11**): colorless oil; [α] -20 (*c* 0.1, MeOH); UV (MeOH) λ_max_ 200 nm; IR (KBr) ν_max_ 3,365, 2,925, 2,872, 1,680, 1,456, and 1,374 cm^−1^; ^1^H and ^13^C NMR data (DMSO-*d*_6_), see Supplementary Tables S4, S5; and HRESIMS *m/z* 253.1817 [M + H] ^+^(calcd for C_15_H_25_O_3_, 253.1804) (Supplementary Figures S91–S99).

Bilaiaeacorenol L (**12**): colorless oil; [α] -40 (*c* 0.1, MeOH); UV (MeOH) λ_max_ 200 nm; IR (KBr) ν_max_ 3,335, 2,932, and 1,679 cm^−1^; ^1^H and ^13^C NMR data (DMSO-*d*_6_), see Supplementary Table S6; and HRESIMS *m/z* 253.1806 [M - H]^-^ (calcd for C_15_ H_25_O_3_, 253.1804) (Supplementary Figures S100–S108).

Bilaiaeacorenol M (**13**): colorless oil; [α] +8 (*c* 0.1, MeOH); UV (MeOH) λ_max_ 201 nm; IR (KBr) ν_max_ 3,366, 2,931, 1732, and 1,246 cm^−1^; ^1^H and ^13^C NMR data (DMSO-*d*_6_), see Supplementary Table S6; and HRESIMS *m/z* 335.1833 [M + Na]+(calcd for C_17_H_28_O_4_Na, 335.1834) (Supplementary Figures S109–S117).

Bilaiaeacorenol N (**14**): colorless oil; [α] +12 (*c* 0.1, MeOH); UV (MeOH) λ_max_ 200 nm; IR (KBr) ν_max_ 3,355, 2,929, 1,679, 1,447, and 1,204 cm^−1^; ^1^H and ^13^C NMR data (DMSO-*d*_6_), see Supplementary Table S6; and HRESIMS *m/z* 255.1959 [M +H]+(calcd for C_15_H_27_O_3_, 255.1960) (Supplementary Figures S118–S126).

Bilaiaeacorenol O (**15**): colorless monoclinic crystals (acetone); mp. 108–110°; [α] +12 (*c* 0.1, MeOH); UV (MeOH) λ_max_ 201 nm; IR (KBr) ν_max_ 3,420, 2,924, 2,854, 1732, 1,456, and 1,247 cm^−1^; ^1^H and ^13^C NMR data (DMSO-*d*_6_), see Supplementary Tables S4, S5; HRESIMS *m/z* 335.1833 [M + Na]^+^ (calcd for C_17_ H_28_O_5_Na, 335.1834); and Flack parameter: –0.03 (11) (Supplementary Figures S127–S135).

Bilaiaeacorenol P (**16**): colorless oil; [α] +10 (*c* 0.1, MeOH); UV (MeOH) λ_max_ 201 nm; IR (KBr) ν_max_ 3,365, 2,924, 2,870 1,435, and 1,374 cm^−1^; ^1^H and ^13^C NMR data (DMSO-*d*_6_), see Supplementary Tables S4, S5; and HRESIMS *m/z* 293.1723 [M + Na]+(calcd for C_15_H_26_O_4_Na, 293.1729) (Supplementary Figures S136–S144).

Bilaiaeacorenol Q (**17**): colorless oil; [α] -20 (*c* 0.1, MeOH); UV (MeOH) λ_max_ 199 nm; IR (KBr) ν_max_ 3,420, 1,648, and 1,387 cm^−1^; ^1^H and ^13^C NMR data (DMSO-*d*_6_), see Supplementary Table S7; and HRESIMS *m/z* 253.1801 [M + H]^+^ (calcd for C_15_H_27_O_4_, 253.1804) (Supplementary Figures S145–S153).

Bilaiaeacorenol R (**18**): colorless oil; [α] -4 (*c* 0.1, MeOH); UV (MeOH) λ_max_ 200 nm; IR (KBr) ν_max_ 3,354, 2,925, and 1,679 cm^−1^; ^1^H and ^13^C NMR data (DMSO-*d*_6_), see Supplementary Table S7; and HRESIMS *m/z* 267.1592 [M -H]^-^ (calcd for C_15_H_23_O_4_, 267.1596) (Supplementary Figures S154–S162).

### Hydrolysis

Analog **9** (1.0 mg) was dissolved in 1.0 ml MeOH, and 2.4 mg K_2_CO_3_ was added to stir at room temperature overnight. Subsequently, 1.0 ml H_2_O was added to the MeOH solution, which was extracted by 3 ml EtOAc. The EtOAc solution was dried under vacuum, and the hydrolyzed product was then detected by a ^1^H NMR spectrum (DMSO-*d*_6_) and optical rotation. Adametacorenols A and B, and analogs **13** and **15**, respectively, were hydrolyzed in the same manner as for compound **9**.

Hydrolyzed product of compound **9**: [α] -21 (*c* 0.05, MeOH), ^1^H NMR data, see Supplementary Figure S169.

Hydrolyzed product of compound **13**: [α] +14 (*c* 0.05, MeOH), ^1^H NMR data, see Supplementary Figure S170.

Hydrolyzed product of compound **15**: [α] +16 (*c* 0.1, MeOH), ^1^H NMR data, see Supplementary Figure S171.

Hydrolyzed product of adametacorenol A: [α] -10 (*c* 0.1, MeOH), ^1^H NMR data, see Supplementary Figure S172.

Hydrolyzed product of adametacorenol B: [α] -22 (*c* 0.1, MeOH), ^1^H NMR data, see Supplementary Figure S173.

### ECD calculation

By MacroModel 10.8.011 software using the MMFF94S force field with 2.5 kcal/mol energy cutoff, mixed torsional/low-mode conformational searches were carried out by SYBYL-X 2.0. Geometry re-optimizations of the resultant conformers (ωB97X/TZVP with the PCM solvent model for MeOH) and TDDFT calculations were performed with Gaussian 09 using B3LYP, the TZVP basis set, and the same solvent model, as in the DFT optimization step at the b3lyp/6–31 + g(d) level with the solvent of MeOH. First, 30 electronic excitations involving energies, oscillator strengths, and rotational strengths (velocity) were calculated by the TDDFT methodology at the b3lyp/6–31 + g (d,p) level. ECD data were simulated by the overlapping Gaussian function, and the simulated spectra of conformers were averaged on the basis of the Boltzmann distribution theory and the relative Gibbs free energy (ΔG). The Merck molecular force field (MMFF) conformational search resulted in initial conformers, which were re-optimized at the ωB97X/TZVP PCM/MeOH level, yielding low-energy conformers over 1% Boltzmann population.

### Crystal data

Crystal data on compounds **1**, **2**, **8**, and **15** were collected with Cu Kα radiation at T = 100.01 (10) K on a Rigaku Oxford Diffraction XtaLAB Synergy four-circle diffractometer, and the data were collected, as shown in Supplementary Figures S164–S168 and Supplementary Tables S8, S38. Crystallographic data have been deposited at the Cambridge Crystallographic Data Center as supplementary publications (CCDC 2211217 for **1**, CCDC 2211219 for **2**, CCDC 2064519 for **8**, and CCDC 2211218 for **15**).

### Cell culture and cell viability assay

Murine BV-2 microglial cells were obtained from the Cell Culture Center of Institute of Basic Medical Sciences, Chinese Academy of Medical Sciences, and the cells were cultured in Dulbecco’s modified Eagle’s medium (Gibco) together with 10% (v/v) fetal bovine serum (HyClone) within a 5% CO_2_ incubator at 37°C. The MTT method was utilized to detect the cytotoxicity of the compound to read the absorbance at 570 nm using a microplate spectrophotometer (Thermo Scientifics, United States).

### Measurement of nitric oxide production

In the presence or absence of LPS (1 μg/ml), murine BV-2 microglial cells were treated with compounds with different concentrations for 24 h. The same volume of Griess reagent was added to the supernatant of culture media. The Griess method was used to determine the NO levels under the absorbance at 540 nm measured using a microplate spectrophotometer (Thermo Scientifics, United States). Based on the established calibration curve of standard sodium nitrite solutions, the content of nitrite was calculated.

### Western blot

In 12-well plates, BV-2 cells were seeded to incubate with LPS (1 μg/ml) for 1 h. Each compound was incubated with BV-2 cells for 16 h. Phenylmethylsulfonyl fluoride-protease inhibitor cocktail as the cell extraction buffer was used to lyse cells. Nuclear and cytosolic extraction kits were applied for the collection of the cytosolic and nuclear extracts. Upon SDS-PAGE, proteins were purified and transferred to PVDF membranes (Millipore). After treatment with 5% (W/V) skim milk in TBST (Tris-buffered saline with 0.1% Tween 20) for 1 h, the membranes were maintained at 4°C overnight. The membrane was washed and then incubated with a secondary antibody at 20°C for 1 h. The target proteins were visualized under a chemiluminescence (ECL) detection system, and the relative optical densities were analyzed by Image Master™ 2D Elite software.

### Immunofluorescence assay

Prior to LPS induction, BV-2 cells were pretreated by the compound (2 μM) in DMSO. Cells were seeded in glass coverslips, which were then treated with cold 4% paraformaldehyde and 0.2% Triton X-100 (in PBS). Subsequently, 5% BSA (in PBS) was added to the coverslips to stay for 1 h, and cells were incubated with NF-κB p65, a primary antibody, at 4°C overnight. After adding labeled Alexa Fluor 594 (Proteintech) and the secondary antibody for 1 h, cells were stained with DAPI, and the washed coverslips were sealed. Images were detected using a fluorescence microscope (OLYMPUS IX83).

## Results and discussion

**FIGURE 1:**
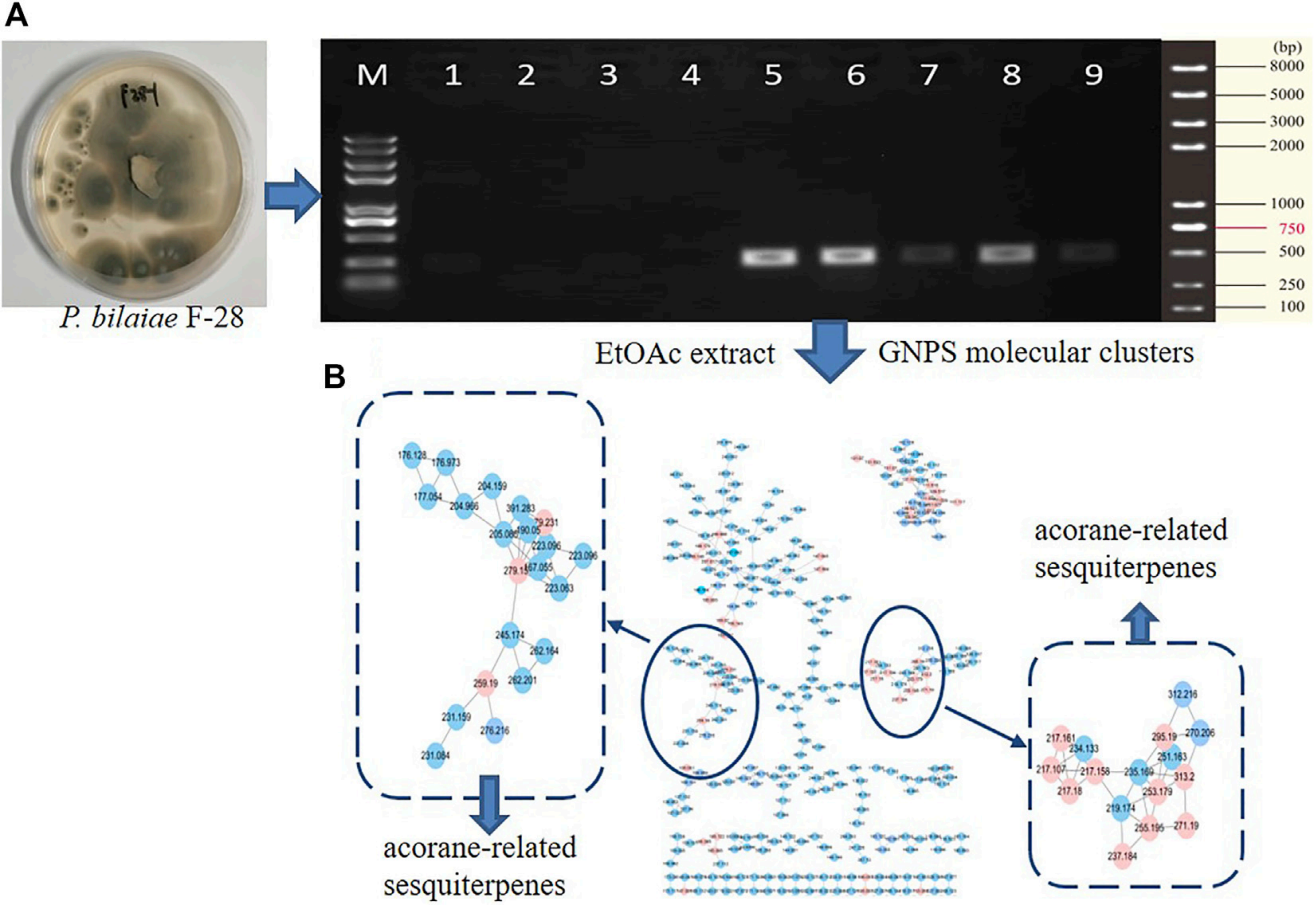
Genome characterization and molecular networks. **(A)** qRT-PCR detection of terpene genes. **(B)** GNPS-based molecular clusters.

**FIGURE 2:**
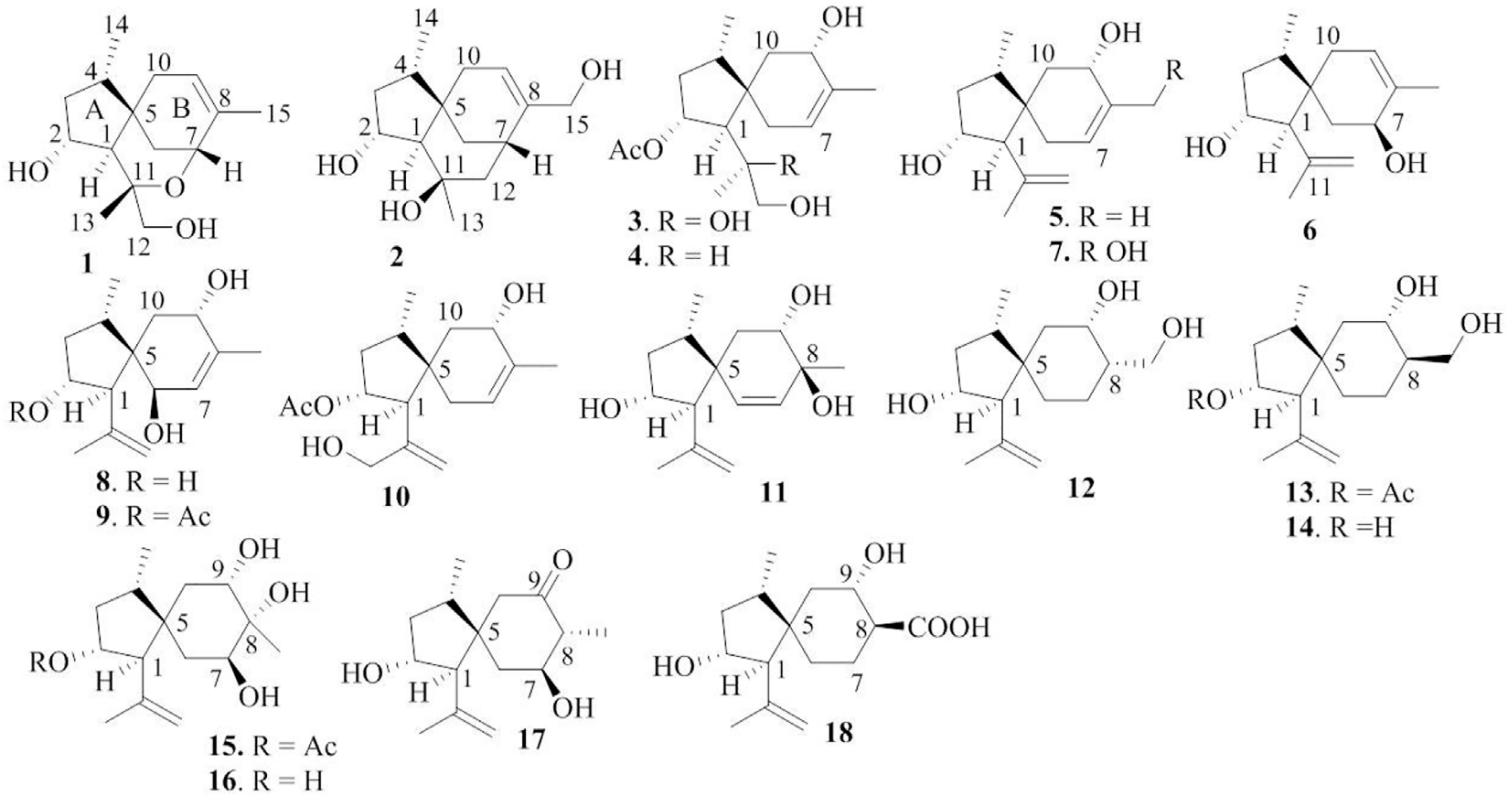
Chemical structures of bilaiaeacorenol A–R (**1–18**).

With the aim to discover chemical diversity with pharmaceutical bioactivity, a deep-sea derived fungus, *P. bilaiae* F28, was selected for chemical examination with the help of bioinformatics and chemo-informative data, which imply that this fungal strain is capable of producing diverse and novel acorane sesquiterpenes. Anti-SMASH genome sequence analysis revealed a total of nine terpene synthases (Supplementary Table S1) in the F28 strain, and qRT-PCR detection showed five terpene genes obviously expressed (Figure 1A). Gene annotation (Supplementary Table S2) revealed gene g10525 with a high homologous identity to ffsc6, a terpene cyclase for the catalysis of acorane core formation (Brock et al., 2013). These findings suggested that the F28 strain shows potential to produce acorane-related sesquiterpenes. To validate whether the terpene genes are really activated in culture conditions, an LC-MS/MS molecular networking approach was applied to detect the chemical metabolites of the cultured fungus by the GNPS database. Based on the LC-MS/MS spectral similarity, molecular networking categorizes the chemical metabolites with similar scaffolds into clusters (Figure 1B). Analyses of MS/MS data in the nodes of clusters annotated a profile of acorane-type analogs, indicating the activation of terpene genes in the culture medium. Chromatographic separation of the EtOAc extract of the large-scale cultured fungus resulted in the isolation of 20 acorane sesquiterpenes, including 18 undescribed acoranes, namely, bilaiacorenol A–R (**1–18**) (Figure 2), along with adametacorenols A (**19**) and B (**20**). Herein, the structural determination of new sesquiterpenes and their anti-neuroinflammation are described.

### Structure elucidation of new acoranes

**FIGURE 3:**
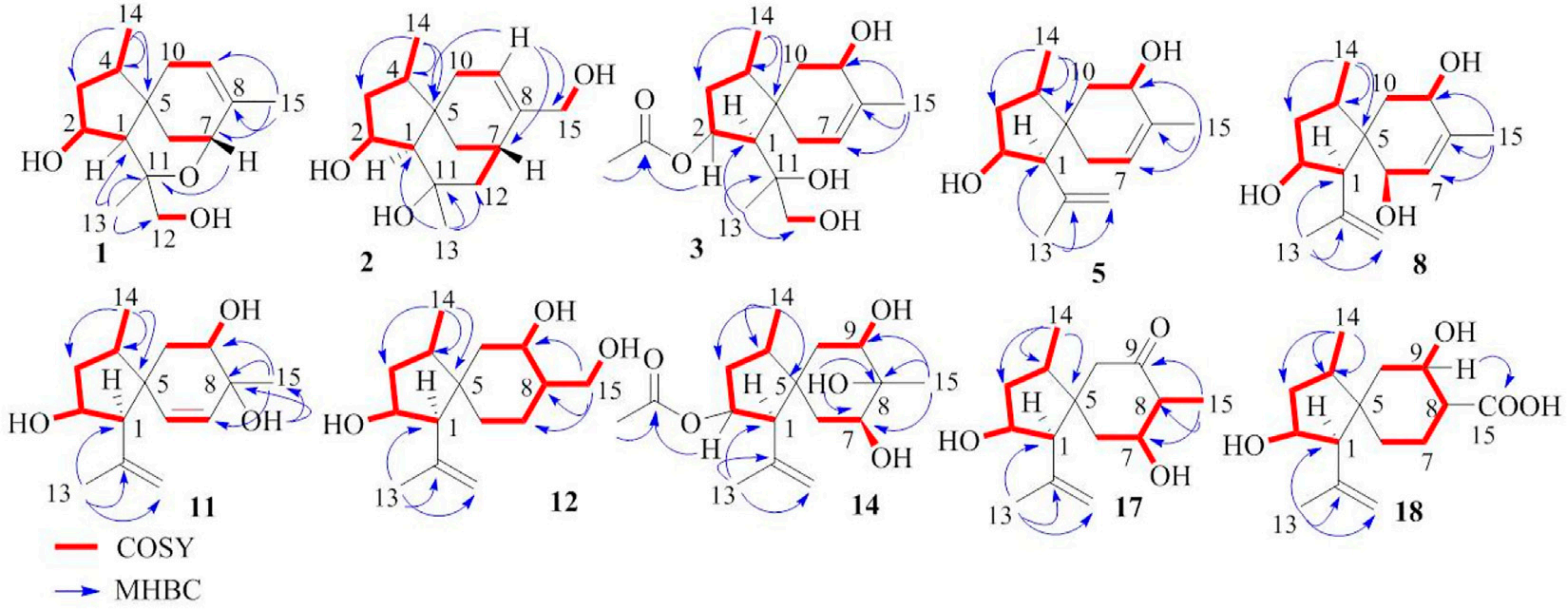
Key COSY and HMBC correlations of **1–3**, **5**, **8**, **11–12**, **14**, and **17–18**.

**FIGURE 4:**
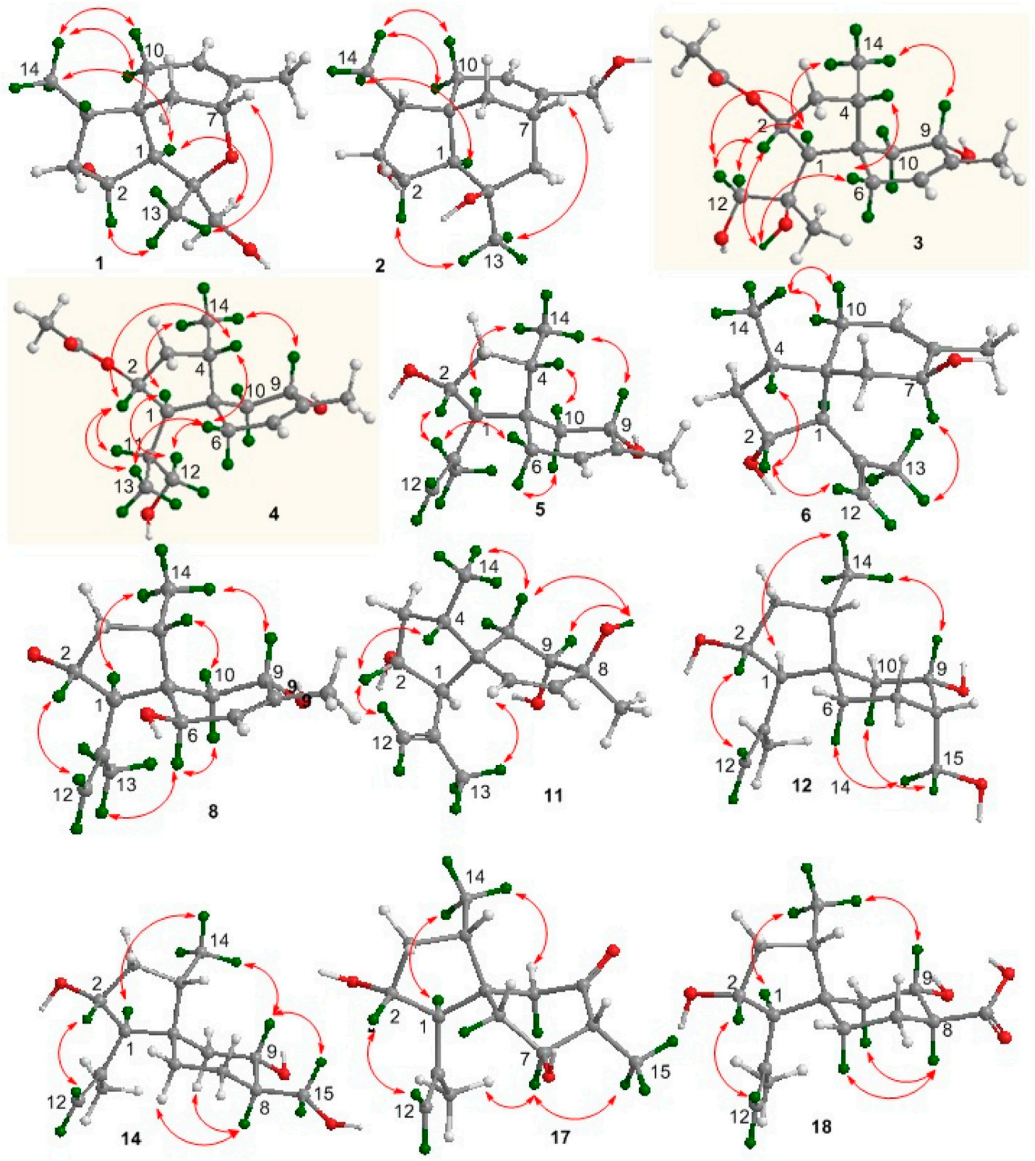
Key NOE correlations of **1–4**, **12**, **14**, and **17–18**.

**FIGURE 5:**
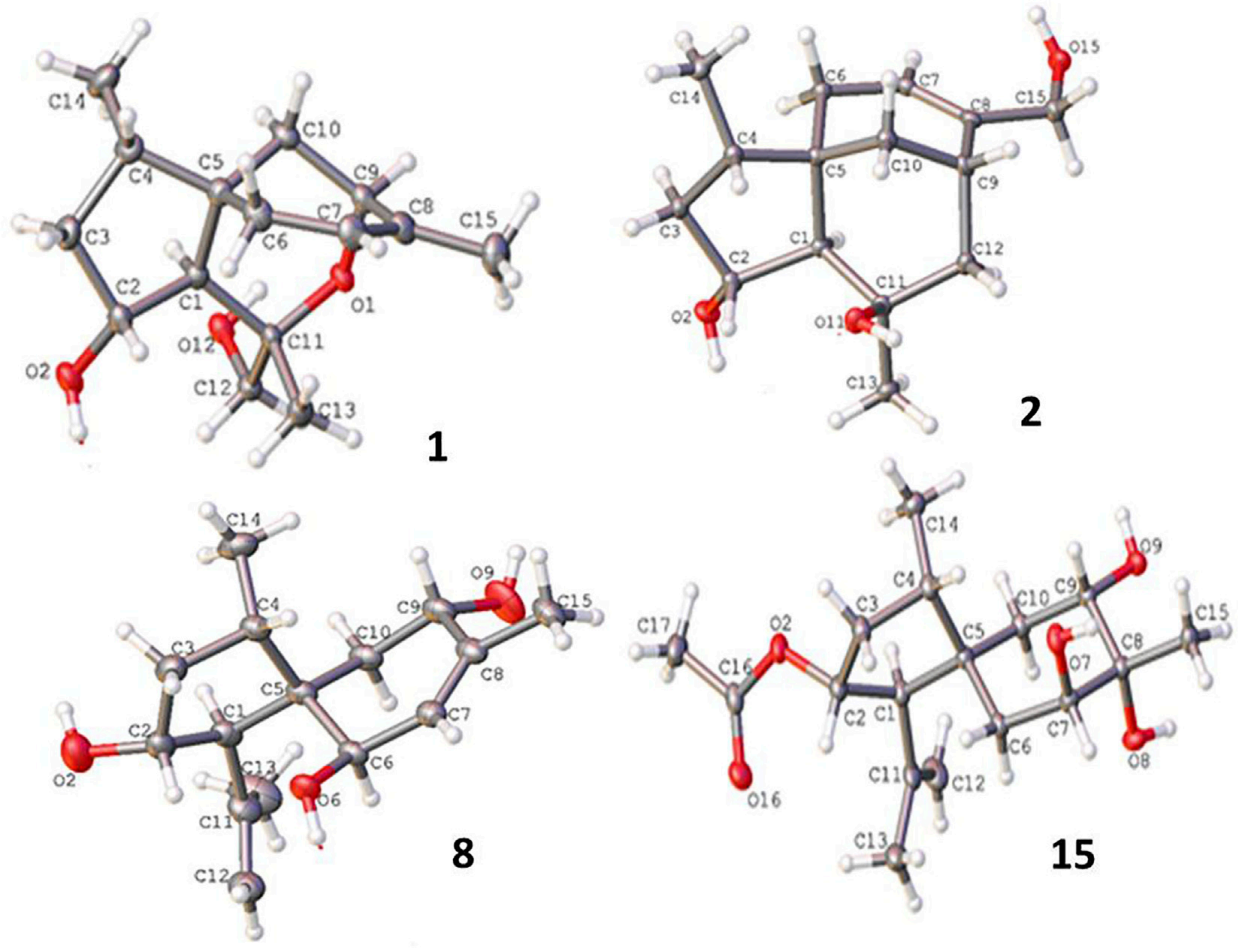
Structures of X-ray diffractions of **1–2**, **8**, and **15**.

Bilaiacorenol A (**1**), a colorless amorphous powder, has a molecular formula of C_15_H_24_O_3_, as established by HRESIMS and NMR data. Its ^13^C NMR data (Supplementary Table S5) showed 15 carbon resonances, which were classified into 13 sp3 and two sp2 carbons for a double bond by the APT and HSQC data. The sp3 resonances involved three methyl, four methylene, four methine, and two nonprotonated carbons. The COSY correlations from H-2 (*δ*_H_ 4.02, ddt, *J* = 2.0, 4.8, and 10.0 Hz) to H-1 (*δ*_H_ 1.76, d, *J* = 10.0 Hz), OH-2 (*δ*_H_ 5.12, d, *J* = 4.8 Hz), and H_2_-3 (*δ*_H_ 1.00, 2.43), and from H-4 (*δ*_H_ 1.73, ddq, *J* = 4.8, 7.2, and 8.0 Hz) to H_2_-3 and H_3_-14 (*δ*_H_ 0.92, d, *J* = 7.2 Hz) along with the HMBC correlations from C-5 (*δ*_C_ 40.0) to H-1, H-2, H_2_-3, and H-4, established a 4-methyl-2-hydroxycyclopentane unit. In addition, a cyclohexene unit was elucidated by the COSY relationships between H_2_-6 (*δ*_H_ 1.42, 1.57)/H-7 (*δ*_H_ 3.90, *J* = 2.0, 3.2 Hz) and H-9 (*δ*_H_ 5.38 br)/H_2_-10 (*δ*_H_ 1.87, 2.31) in association with the HMBC correlations from H_3_-15 (*δ*_H_ 1.72 brs) to C-7 (*δ*_C_ 69.2), C-8 (*δ*_C_ 135.8), and C-9 (*δ*_C_ 125.4) and from H_2_-6 to C-5 and C-10. These findings demonstrated an acorane core in which a *spiro*-fusion of the two moieties at C-5 with a methyl location at C-8 was characterized. The substitutions of the dioxygenated isopropyl group at C-1 (*δ*_C_ 53.1) were deduced by the COSY relationship between H_2_-12 (*δ*_H_ 3.16, 3.18) and OH-12 (*δ*_H_ 4.83, t, *J* = 5.0 Hz) together with the HMBC correlations from H_3_-13 (*δ*_H_ 1.12, s) to C-1, C-11 (*δ*_C_ 77.2) and C-12 (*δ*_C_ 72.5). The formation of an ether bond across C-7 (*δ*_C_ 69.2) and C-11 was evident from the HMBC correlation between H-7 and C-11 (Figure 3). The NOE correlations between H-1/H_3_-14 and H-2/H_3_-13 suggested a cofacial relationship of H-2 with H-4, which was in the opposite face toward H-1. The NOE correlations between H_3_-14 and H_2_-10 established the *spiro*-chirality center C-5, for which H_2_-10 was spatially approximated to H_3_-14. Additional NOE correlations between H_2_-12/H-1 and H_3_-13/H-7 (Figure 4) established the relative configurations of C-7 and C-11, in which H_2_-12 was spatially approximated to H-1. The X-ray diffraction data for the single crystal of **1** using the Flack parameter [0.00 (6)] assigned the absolute configurations as 1*R*, 2*R*, 4*S*, 5*S*, 7*R*, and 11*S* (Figure 5).

Bilaiacorenol B (**2**) was obtained as a colorless amorphous crystal, and its molecular formula (C_15_H_24_O_3_) was determined on the basis of the HRESIMS data. The NMR data on compound **2** (Supplementary Tables S3, S5) resembled those of **1**, and the 2D NMR data established a corane-type nucleus. The distinction was observed in the NMR data for the cyclohexene ring and the side chain at C-1 (*δ*_C_ 60.0). The connection of C-7 to C-11 (*δ*_C_ 69.4) *via* a methylene unit instead of an ether bond was demonstrated by the COSY relationship between H-7 (*δ*_H_ 2.28, ddt, *J* = 2.0, 4.0, and 9.0 Hz) and H_2_-12 (*δ*_H_ 1.34, 1.80), and hydroxylation at C-11 was clarified by the HMBC correlations from OH-11 (*δ*_H_ 3.91, s) to C-1, C-11 (*δ*_C_ 69.4), C-12 (*δ*_C_ 41.4), and C-13 (*δ*_C_ 30.3). In addition, a hydroxymethyl group at C-8 (*δ*_C_ 144.0) was deduced by the COSY coupling between H_2_-15 (*δ*_H_ 3.78, 3.79) and OH-15 (*δ*_H_ 4.57, t, *J* = 5.0 Hz) together with the HMBC correlations of H_2_-15 to C-7 (*δ*_C_ 29.3), C-8, and C-9 (*δ*_C_ 118.3). Similar to the NOE data on **1**, the correlations from H_3_-14 to H-1 and H_2_-10 and between H-2 and H_3_-13 suggested the same relative configurations at C-2 and C-4 and the *spiro*-chirality center C-5 of both compounds. The NOE correlations between OH-11/H-2 and H_2_-12/H-1 (Figure 4) established the spatial closeness among these groups. Based on the data on a single crystal of the Cu/Kα X-ray diffraction experiment, a Flack parameter of –0.05 (9) allowed an unequivocal assignment of the 1*R*, 2*R*, 4*S*, 5*R*, 7*S*, and 11*R* configurations (Figure 5).

Bilaiacorenol C (**3**) was found to have a molecular formula of C_17_H_28_O_5_, according to the HRESIMS data. Its NMR data (Supplementary Tables S3, S5) were characteristic of a corane-type sesquiterpene, related to those of compound **1**. The COSY and HMBC correlations established a planar structure, which was closely related to co-isolated adametacorenol A (Liu et al., 2015). However, the side chain at C-1 (*δ*_C_ 54.7) was assigned to a 1,2-dihydroxyisopropane moiety on the basis of the COSY relationship between H_2_-12 (*δ*_H_ 2.99, 3.07) and OH-12 (*δ*_H_ 4.57, t, *J* = 5.0 Hz) in association with the HMBC correlations from H_3_-13 (*δ*_H_ 1.21 s) and OH-11 (*δ*_H_ 4.09, s) to C-11 (*δ*_C_ 73.3) and C-12 (*δ*_C_ 69.6), and the correlations of C-1 to H_3_-12 and OH-11. The NOE correlations between H-1 and H_3_-14 and between H-2 and H_2_-12 were indicative of the same relative configuration for the cyclopentane ring in both compounds **1** and **3**. Additional NOE correlations between H_3_-14 (*δ*_H_ 0.97, d, *J* = 7.2 Hz) and H-9 (*δ*_H_ 3.90 ddd, *J* = 4.4, 5.0, 6.5 Hz) and between H-4 and H-6b fixed the *spiro*-form of the cyclohexene ring, in which H-9 was spatially approximated to H_3_-14. If H-1 is arbitrarily assigned to R* configuration, the NOE correlations between H-1 and H_2_-12, from OH-11 to H-2 and H_2_-6, and between H_2_-12 and H-2 (Figure 4) suggested an irrotational C_1_-C_11_ bond and 11R* configuration. The experimental ECD data were similar to those calculated for (1*R*, 2*R*, 4*S*, 5*S*, 9*S*, 11*R*)-3 (Supplementary Figure S163), suggesting the *R* configurations for C-1, C-2, and C-11, and the *S* configurations for C-4, C-5, and C-9.

The NMR and MS data (Supplementary Tables S3, S5) revealed bilaiacorenol D (**4**) to be a homolog of compound 3 with the distinction for the side chain at C-1 (*δ*_C_ 49.4). The COSY correlations from H-11 (*δ*_H_ 1.62, m) to H_3_-13 (*δ*_H_ 0.86, d, *J* = 7.0 Hz), H-1 (*δ*_H_ 2.00, dd, *J* = 3.2, 9.0), and H_2_-12 (*δ*_H_ 3.16, 3.23), and the extension of coupling between H_2_-12 and OH-12 (*δ*_H_ 4.54, t, *J* = 5.0 Hz) identified a hydroxylated isopropane unit at C-1. The HMBC correlations from H_3_-13 and H_2_-12 to C-1 and C-11 (*δ*_C_ 33.0) supported compound **4** as a 11-dehydroxylated analog of **3**. The similar NOE data on compounds **3** and **4**, such as the correlations between H-2/H-4, H_3_-14/H-9, and H_3_-14/H-1, suggested the same relative configurations in the backbone. The *J*_H-1/H-11_ value (3.2 Hz) in association with the NOE correlations from H_3_-13 to H-2 and H-6b, from H_2_-12 to H-1 and H_2_-6b, and between H-11 and H-2, also suggested the unrotational C-1/C-11 bond. The similar ECD data (Supplementary Figure S163) suggested that the absolute configuration of compounds **3** and **4** was identical, with the exception of C-11, which was suggested to be the *S* configuration with the help of NOE data.

Bilaiacorenol E (**5**) was found to have a molecular formula C_15_H_24_O_2_ on the basis of the HRESIMS data. Interpretation of the 2D NMR data clarified the planar structure of compound **5** to be identical to a 2-deacetylated adametacorenol A. The similar NOE data between compound 5 and adametacorenol A in association with the comparable experimental ECD data to those calculated for a model molecule of (1*R*, 2*R*, 4*S*, 5*S*, 9*S*)-**5** agreed compound 5 possessing the same absolute configuration as the known homolog. Alkaline hydrolysis of adametacorenol A derived a product whose NMR data (Supplementary Figure S172) and optical rotation were consistent with those of compound **5**, supporting the structural assignment.

Analyses of the 2D NMR and HRESIMS data assigned the planar structure of bilaiacorenol F (**6**) and compound **5** to be identical. The NOE correlations between H-1 (*δ*_H_ 2.17, d, *J* = 5.6 Hz)/H_3_-14 (*δ*_H_ 0.85, d, *J* = 6.8 Hz) and H_3_-13 (*δ*_H_ 1.64, s)/H-2 (*δ*_H_ 4.00, ddt, *J* = 4.8, 5.6, and 10.8 Hz) suggested the same relative configuration of ring A in both compounds **5** and **6**. The distinction was attributed to the NOE interactions between rings A and B, where the NOE correlation between H_3_-14 and H_2_-10 (*δ*_H_ 1.87, br) and the latter protons coupling to olefinic proton H-9 (*δ*_H_ 5.34, brs) suggested the double bond shifted from C-7/C-8 of **5** to C-8/C-9 of **6**. Additional NOE correlation between H_3_-13 and H-7 (*δ*_H_ 3.95) supported the structural assignment.

The 1D and 2D NMR data in association with the HRESIMS data identified bilaiacorenol G (**7**) as a 2-deacetylated adametacorenol B, and it was supported by the chemical conversion of adametacorenol B to compound **7** under alkaline catalysis.

Bilaiacorenol H (**8**) has a molecular formula of C_15_H_24_O_3_, as established by the HRESIMS data, containing an oxygen atom more than that of compound **5**. Comparison of the NMR data (Supplementary Tables S4, S5) revealed the structure of compound **8** closely related to compound **5**, and the cyclopentane moiety of both compounds was identical. With regard to the cyclohexene ring, two hydroxyl groups resided at C-6 and C-9, respectively, were recognized by the COSY correlations between H-6 (*δ*_H_ 3.83, brd, *J* = 6.0 Hz)/OH-6 (*δ*_H_ 4.50, d, *J* = 6.0 Hz) and H-9 (*δ*_H_ 3.97, ddd, *J* = 6.0, 6.8, and 10.0 Hz)/OH-9 (*δ*_H_ 4.56, d, *J* = 6.8 Hz) along with the HMBC correlations from OH-6 to C-5 (*δ*_C_ 52.5), C-6 (*δ*_C_ 68.9), and C-7 (*δ*_C_ 129.1) and from OH-9 to C-8 (*δ*_C_ 136.4), C-9 (*δ*_C_ 66.6), and C-10 (*δ*_C_ 39.5). These data allowed the location of a double bond at C-7/C-8. The similar NOE relationships in ring A of compounds **5** and **8** suggested the same relative configuration for the relevant protons of both compounds. Additional NOE correlations between H_3_-14/H-9 and H_3_-13/H-6 (Figure 4) reflected a *trans*-orientation between H-6 and H-9. The comparable experimental ECD data to those calculated for the model molecule of (1*R*, 2*R*, 4*S*, 5*R*, 6*R*, 9*S*)-**8** (Supplementary Figure S163) clarified the absolute configuration of compound **8**.

Bilaiacorenol I (**9**) was determined as a 2-acetylated analog of compound **8** based on the comparable NMR data, except for the presence of an acetyl group in compound **9**. The location of the acetyoxy group at C-2 was evident from the HMBC correlation between H-2 and the acetyl carbonyl carbon. The similar NOE correlations suggested that both compounds have the same relative configuration. The absolute configuration of compound **9** was the same as that of compound **8** based on the alkaline hydrolysis of compound **9** to produce a hydrolyzed product, whose ^1^H NMR data (Supplementary Figure S169) and specific rotation ([α]^20^_D_ -21) were almost identical to those of compound **8**.

The molecular formula (C_17_H_26_O_4_) of bilaiacorenol J (**10**) was determined by the HRESIMS data, containing an oxygen atom more than that of adametacorenol A. Its NMR data (Supplementary Tables S4, S5) resembled those of adametacorenol A, with the only difference for the substitution at C-13. A hydroxymethylene unit to replace a methyl group of the latter was recognized by the COSY correlation between H_2_-13 (*δ*_H_ 3.78, 3.86) and OH-13 (*δ*_H_ 4.90, t, *J* = 5.6 Hz) together with the HMBC correlations from H_2_-13 to the olefinic carbons C-11 (*δ*_C_ 147.4) and C-12 (*δ*_C_ 109.7), as well as C-1 (*δ*_C_ 52.7). The similar NOE interactions suggested the same relative configuration for both compound **10** and adametacorenol A. The comparable experimental ECD data with those calculated for (1*S*, 2*R*, 4*S*, 5*S*, 7*S*)-**10** reflected the same absolute configuration of both compound **10** and adametacorenol A (Supplementary Figure S163).

The molecular formula of bilaiacorenol K (**11**) was the same as that of compound **8**, as established by the HRESIMS data. A comparison of the NMR data revealed both compounds **8** and **11** share the partial structure of the cyclopentane unit. In regard to the cyclohexene unit, the olefinic coupling between H-6 (*δ*_H_ 5.28, d, *J* = 10.0 Hz) and H-7 (*δ*_H_ 5.27, d, *J* = 10.0 Hz) resided a double bond at C-6 (*δ*_C_ 131.6)/C-7 (*δ*_C_ 133.3), and the HMBC correlations of both H_3_-15 (*δ*_H_ 0.96, s) and OH-8 (*δ*_H_ 4.33, s) to C-7, C-8 (*δ*_C_ 71.7), and C-9 (*δ*_C_ 71.8) along with the COSY correlations from H-9 (*δ*_H_ 3.57, ddd, *J* = 2.4, 6.0, and 9.2 Hz) to H_2_-10 (*δ*_H_ 1.43, 1.64) and OH-9 (*δ*_H_ 4.47, d, *J* = 6.0 Hz) located the hydroxyl groups at C-8 and C-9 and a methyl substitution at C-8. Thus, compound **11** is likely derived from compound **8** by hydroxyl migration from C-6 to C-8, following olefinic transformation. The NOE correlations between H-1/H_3_-14 and H-2/H_3_-13 suggested the same relative configuration of the cyclopentane moiety for both compounds **8** and **11**. The *J*_H-9/H-10a_ value (9.2 Hz) was indicative of an axial orientation of H-9. The *spiro*-chirality center C-5 as the case of compound **8** was evident from the NOE correlations between H_3_-14 and H-9 and between H_3_-13 and H-6. The *cis*-orientation of H-9 with OH-8 was identified by their NOE interaction.

Corane-type sesquiterpenes **12–18** are structurally characteristic of a *spiro*-fusion of cyclopentane with a cyclohexane unit instead of a cyclohexene unit. The distinction was attributed to the different substitution at the backbone.

The 2D NMR data established a corane core of bilaiacorenol L (**12**). Apart from ring A, which was identical to that of compound **11**, the NMR data (Supplementary Table S6) showed two hydroxyl groups in the cyclohexane unit. The location of hydroxyl groups at C-9 (*δ*_C_ 67.5) and C-15 (*δ*_C_ 57.4) was evident from the COSY relationships between H-9 (*δ*_H_ 3.70, dt, *J* = 4.0, 10.0 Hz)/OH-9 (*δ*_H_ 5.00, d, *J* = 4.0 Hz) and H_2_-15 (*δ*_H_ 3.29, 3.60)/OH-15 (*δ*_H_ 4.22, t, *J* = 5.0 Hz) along with the COSY correlations from H-8 (*δ*_H_ 1.81, m) to H-9 and H_2_-15. The same relative configuration of the cyclopentane unit as that of compound 11 was suggested by the similar NOE correlations of the relevant protons. A chair conformer of the cyclohexane was recognized by the *J* values of the protons in cyclohexane. The NOE interactions from H_2_-15 to H-6a and H-10a suggested an axial orientation of the hydroxymethylene unit. As in the case in compound **11**, the NOE correlation between H_3_-14 and H-9 fixed the relative configuration of the *spiro*-center C-5, and H-9 was spatially approximated to H_3_-14.

The planar structure of bilaiacorenol M (**13**) was identified as a 2-acetylated analog of compound **12** on the basis of the diagnostic 2D NMR data. The NOE data suggested the relative configuration of the cyclopentane unit to be consistent with that of compound **12**. Like the case of **12**, the NOE interaction between H_3_-14 and H-9 identified the same *spiro*-configuration of both compounds **12** and **13**. The *J*_H-7/H-8_ (10.0 Hz) value and the NOE correlation between H_2_-15 and H-9 suggested a *trans* axial–axial relationship between H-8 and H-9, reflecting an equatorial orientation of H_2_-15. This resulted in an unshielded C-15 (*δ*_C_ 63.2) of compound **13** comparing that of compound **12** (*δ*_C_ 57.4).

Diagnostic 2D NMR (Supplementary Table S6) and MS data identified bilaiacorenol N (**14**) to be a 2-deacetylated **13**, and this was confirmed by the chemical conversion of compound **13** to **14** under alkaline catalysis.

The molecular formula of bilaiacorenol O (**15**) was determined to have an oxygen atom of more than **13**, as provided by the HRESIMS data. The NMR data revealed the cyclopentane moiety of both compounds **13** and **15** to be identical. The distinction was attributed to the substitution at the cyclohexane moiety, where three hydroxyl protons were observed at OH-7 (*δ*_H_ 4.56, d, *J* = 2.4 Hz), OH-8 (*δ*_H_ 3.78, s), and OH-9 (*δ*_H_ 4.08, d, *J* = 4.4 Hz), which were clarified by the COSY relationships between H-7 (*δ*_H_ 3.53, ddd, *J* = 2.4, 3.0, and 3.6 Hz)/OH-7 and H-9 (*δ*_H_ 3.49, ddd, *J* = 2.0, 4.4, and 10.0 Hz)/OH-9. The HMBC correlations of H_3_-15 (*δ*_H_ 1.09, s) and OH-8 to C-7 (*δ*_C_ 73.9), C-8 (*δ*_C_ 74.0), and C-9 (*δ*_C_ 70.5) further supported the hydroxyl locations. The *J*_H-9/H-10a_ (10 Hz) value and the *J*_H7-H6_ values (3.0, 3.6 Hz) reflected an axial H-9 and an equatorial H-7. The NOE correlation between H-9 and H_3_-15 suggested the cofacial relationships of these groups. The remaining NOE data were similar to those of compound **13**. The single-crystal X-ray diffraction using Cu-Kα radiation (Figure 5) clarified the absolute configurations of compound 15 to be 1R, 2R, 4S, 5R, 7S, 8R, and 9S.

Bilaiacorenol P (**16**) was determined as a 2-deacetylated **15** on the basis of the NMR and MS data. Alkaline hydrolysis of compound **15** to derive compound **16** supported the structure assignment.

Bilaiacorenol Q (**17**) has a molecular formula of C_15_H_24_O_3_, as established by the HRESIMS data. The 2D NMR data provided the partial structure regarding the cyclopentane unit to be identical to that of compound **16**. The distinction was found in the cyclohexane moiety, where a ketone group at C-9 (*δ*_C_ 211.2) was evident from the HMBC correlations from H_3_-15 (*δ*_H_ 0.93, d, *J* = 6.0 Hz) to C-7 (*δ*_C_ 71.4), C-8 (*δ*_C_ 52.5), and C-9. The *J*_H-7/H-6a_ and *J*_H-7/H-8_ values (10 Hz) were characteristic of a chair conformation of the cyclohexane ring. The NOE data suggested that both compounds **17** and **16** have the same relative configuration for ring A. The NOE interaction between H_3_-14 (*δ*_H_ 0.89, d, *J* = 7.2 Hz) and H_2_-10 (*δ*_H_ 2.22, 2.28) fixed the *spiro*-orientation, and the correlations of H-7 (*δ*_H_ 3.37, ddt, *J* = 4.8, 6.0, 10.0 Hz) with H_3_-13 (*δ*_H_ 1.69, s) and H_3_-15 (*δ*_H_ 0.93, d, *J* = 6.0 Hz) assigned the same face of H-7 and H_3_-15 (Figure 4), and the former was spatially approximated to H_3_-13.

Bilaiacorenol R (**18**) has a molecular formula of C_15_H_24_O_4_, as determined by the HRESIMS data. The NMR data on compound **18** (Supplementary Table S7) resembled those of compound **14**, indicating structure similarity. The difference was attributed to the substituent at C-8, where a carboxylic group of compound **18** was found for C-15 (*δ*_C_ 176.3) due to the HMBC correlations of C-15 to H-8, H-9 and H_2_-7. The *J*_H-7/H-8_ (12 Hz) value and the similar NOE data suggested that both compounds **14** and **18** have the same relative configuration.

Compounds **19** and **20** were identical to adametacorenols A and B by the comparison of their spectroscopic data and the specific rotations with those reported in the literature (Liu et al., 2015). Based on the configurational assignments, the stereogenic centers in ring A regarding ring A of all analogs are conserved. This can be explained by the analogs derived from the same acorane precursor. Thus, the comparison of experimental and calculated ECD data (Supplementary Figure S163) in association with the NOE data enables to assign the absolute configurations of the amorphous analogs.

### Biogenetic postulation

**SCHEME 1:**
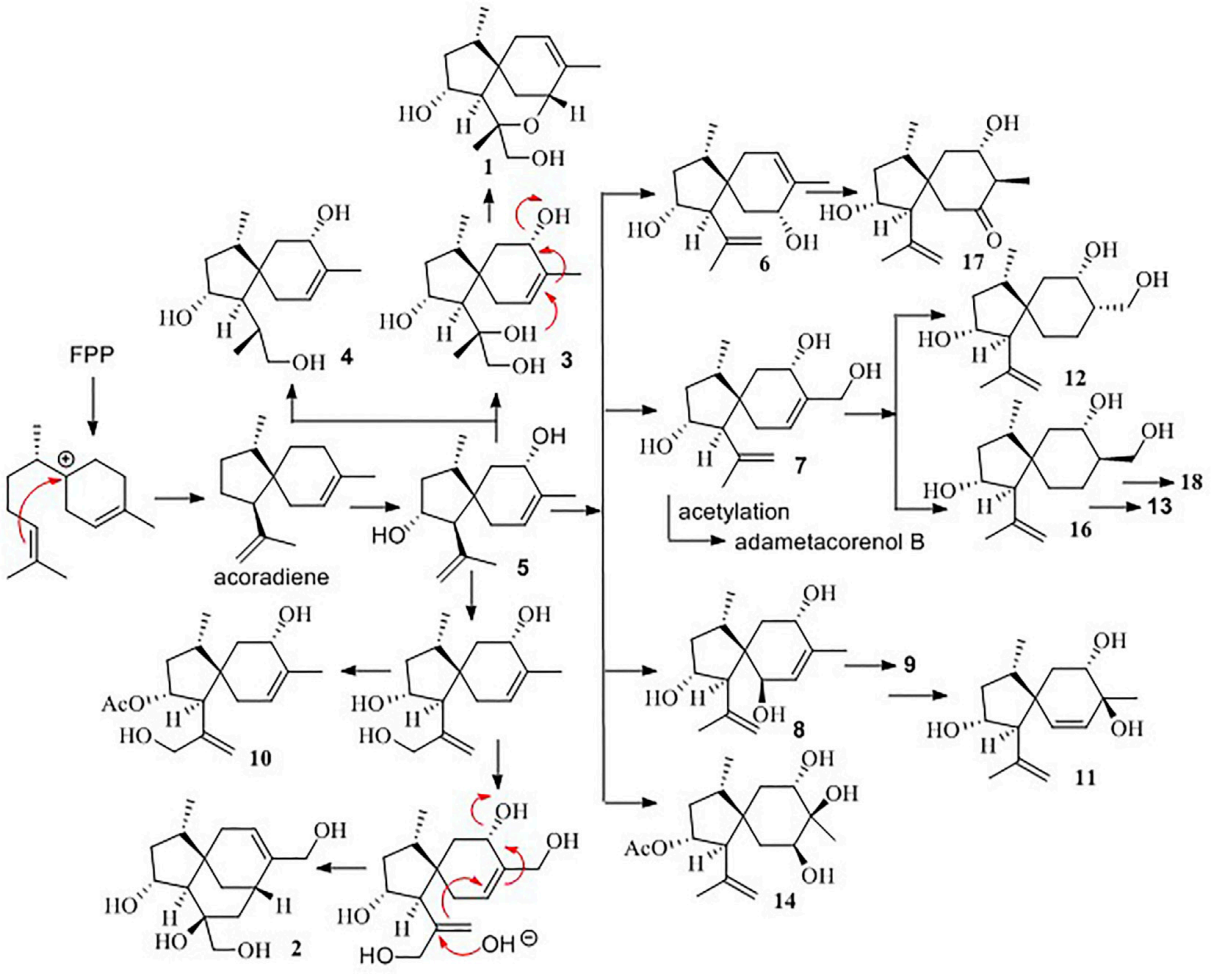
Biogenetic relationships of bilaiaeacorenols.

Biogenetically, the bisabolyl cation, as derived from farnesyl diphosphate (FPP), is an intermediate to generate acoradiene (Citron et al., 2011), which is considered the principal component to derive an array of acorane-type sesquiterpenes *via* various oxidation and rearrangement mechanisms. 2,9-Dihydroxylation of acoradiene generates compound **5**, and further hydroxylation of compound **5** derives compounds **7** and **8**. Acetylation of compounds **5**, **7**, and **8** affords adametacorenols A and B, and compound **9**. 13-Hydroxylation of adametacorenol A derives compound **10**, but analog **6** is likely derived from 5 *via* hydroxyl migration and olefinic transformation. A similar pathway occurs for the conversion of compound **8** to **11**. Reduction of the double bond in compound 7 and adametacorenol B affords compounds **12**, **13**, and **14**. Analogs **15** and **16** are assumed to be derived from adametacorenols A and B *via* epoxidation and hydrolysis, but analog **17** is likely derived from epoxidated 5, following oxidative epoxide cleavage. Oxidation of hydroxymethylene C-15 in compound **13** converts to **18**. Epoxidation of adametacorenol A at the side chain of ring A, following epoxide cleavage, derives 3 and 4. Analogs 1 and 2 are depicted to be derived from 11,12-epoxided 5, followed by ring fusion (Scheme 1). Since acoradiene is a fungal product isolated from our fungal strain and other organisms, it is an intermediate to derive diverse acorane analogs. Hydroxylation or oxidation at ring B is depicted to occur after the formation of the bicyclic core. The different C-5 configuration in **5** and **6** is thus raised by the hydroxylation at C-7 or C-9, respectively, rather than the induction by different fusion of the bicyclic core. The putative biogenetic relationships suggested that all isolates maintain the conserved configurations in ring A due to compound **5** as the sole precursor.

**SCHEME 2:**
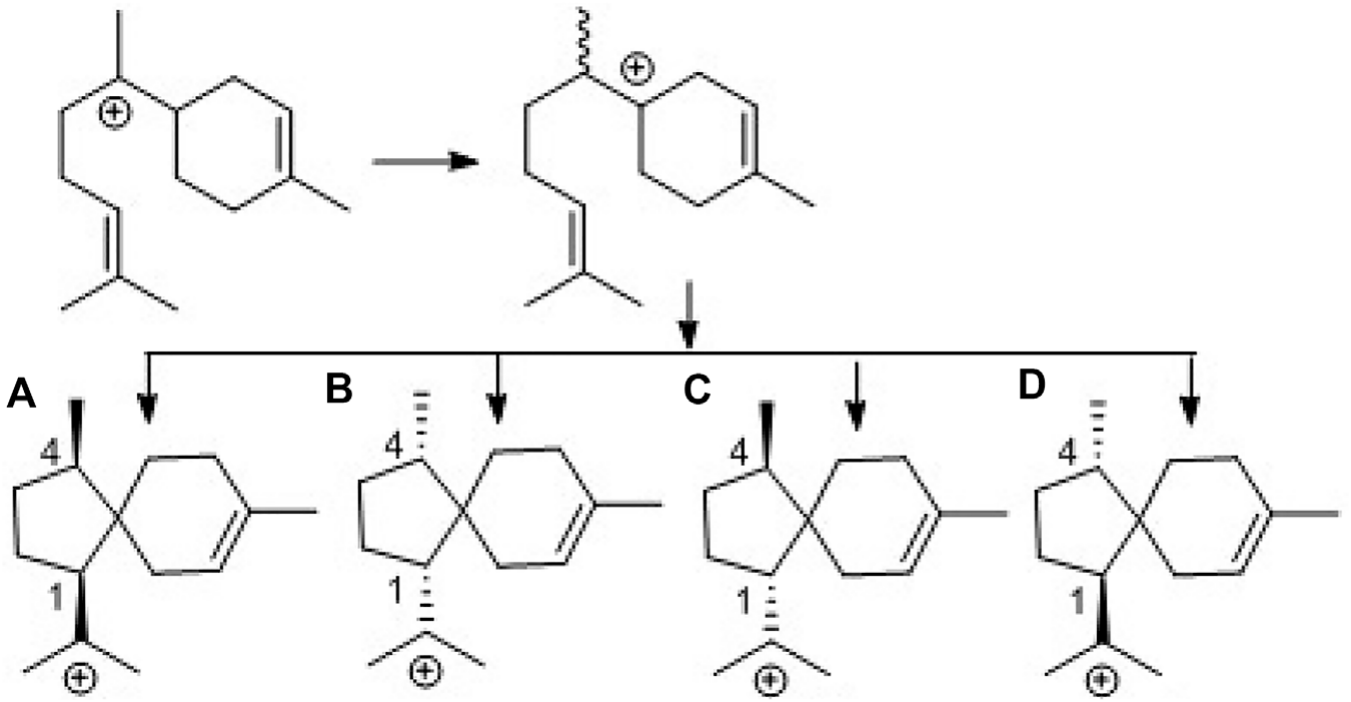
Biogenetic formation of the stereospecific centers of acorane cores. **(A)**: 1R,4R-form, **(B)**: 1S,4S-form, **(C)**: 1S,4R-form, **(D)**: 1R,4S-form.

To provide evidence for the biosynthetic process of these sesquiterpenes, genome sequencing was conducted, and nine putative terpenoid synthases (TS) in different locations were annotated by anti-SMASH analysis (Supplementary Table S2). Among them, the gene *g10525* showed a high identity to *Ffsc6*, a terpene cyclase used for the synthesis of acorenols in ascomycete *Fusarium fujikuroi* (Brock et al., 2013). Using heterologous hosts to express *g10525* in *Aspergillus nidulans* A1145, a number of sesquiterpenes were detected by LC-MS/MS spectra, and a molecular ion at *m/z* 204 was consistent with that of acoradiene. These data supported that the acorane-type derivatives synthesized in *P. bilaiae* followed the similar pathways as other fungal origins reported in the literature. Notably, corane-type sesquiterpenes from different fungal species display distinct stereogenic centers regarding the cyclopentane ring. Theoretically, the cyclization of the homobisabolyl cation derives four diastereomeric acorenyl cations (Scheme 2). In *Trichoderma* strains, the intermediate B derives tricho-acorenol and relevant analogs as the main components, which are characteristic of *cis*-orientation of the substituents at C-1 and C-4 (Aoyagi et al., 2008; Citron et al., 2011; Li et al., 2011; Wu et al., 2011; Zhang et al., 2017). Eupho-acorenols from a plant are diastereoisomers of tricho-acorenol with *trans*-orientation of 1,4-substituents, as catalyzed by the sesquiterpene synthase EfTPS12 (Zhu et al., 2021). The stereogenic assembly pattern of acorane sesquiterpene from the plant *Lysionotus pauciflorus* coincides with those from *Trichoderma* fungi but in a different manner from that in the plant *Daphne genkwa* (Guo et al., 2020), which assembles the acorane skeleton through the intermediate C. A basidiomycete (mushroom)-derived acorane-type sesquiterpenoid possesses the scaffold (Sandargo et al., 2019) which is likely constructed by the intermediate A. In this work, bilaiacorenols are obviously produced from the intermediate D and are characteristic of the 1,4-*trans*-substituted spiro [4,5]decane core. These findings suggest that the terpene cyclases from different origins play similar rules to assemble the acorane core but with a stereospecific selection of precursors, implying g10525 as a new sesquiterpene synthase. The detailed functions and catalysis mechanism require further investigation.

### Anti-neuroinflammation effects

In preliminary *in vitro* bioassay, the inhibition of lipopolysaccharide (LPS)-reduced nitric oxide (NO) production in murine BV-2 microglial cells was detected (Cheng et al., 2011; Mendes et al., 2012). Prior to the detection, the MTT method was used to test the analogs for their cytotoxic effects of analogs by counting and analyzing cell viability. All tested compounds showed no to weak cytotoxicity due to their IC_50_ values more than 100 μM (Supplementary Table S2). At non-toxic concentrations (10 μM), six acorane-type analogs exhibited potent effects for the reduction of the LPS-induced NO production (Supplementary Table S2), showing higher activities than the positive control NG-monomethyl-L-arginine (L-NMMA), a nitric oxide synthase (NOS) inhibitor. Analyses of the structures related to activities suggested analogs with 2-acetylation increasing the activity in comparison with that for 2-hydroxylated counterparts, such as **8** vs. **9**, **13** vs. **14**, and **15** vs. **16**, indicating the substitution at C-2 directly affected the activity. Hydroxylation at the cyclohexane ring also affects the bioactivity, such as analogs with the triol unit (**15** and **16**) showed higher effects than those with diol and mono-hydroxylation. The most active analog **18** with a carboxylic group at C-8 showed more effects than those with the hydroxymethylene unit at C-8 (Table 1).

**TABLE 1:** Inhibitory effects of **1–20** against NO production in LPS-induced BV-2 cells.

No	IC_50_ (μM)	CC_50_ (μM)
1	>10	>100
2	6.1 ± 2.3	>100
3	5.3 ± 1.1	>100
4	7.6 ± 1.2	>100
5	>10	>100
6	>10	>100
7	>10	>100
8	>10	>100
9	3.5 ± 0.1	>100
10	>10	>100
11	8.7 ± 2.1	>100
12	>10	>100
13	0.53 ± 0.47	>100
14	>10	>100
15	3.7 ± 0.1	>100
16	7.5 ± 0.2	>100
17	>10	>100
18	0.5 ± 1.2	>100
19	>10	>100
20	>10	>100
L-NMMA	6.8 ± 4.2	>100

L-NMMA, NG-monomethyl-L-arginine; CC, cell cytotoxicity.

**FIGURE 6:**
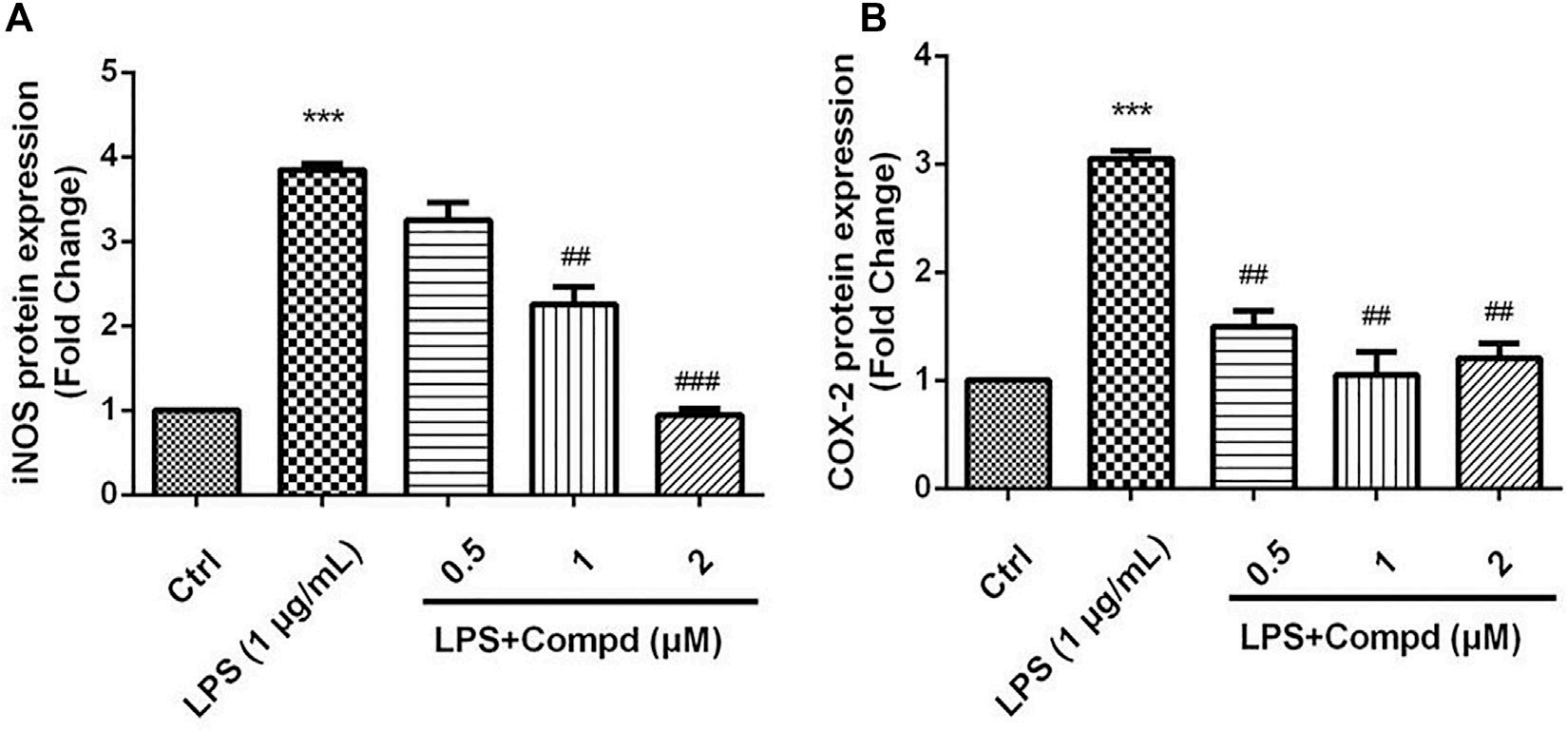
Analog **18** inhibited the expression of iNOS and COX-2 in LPS-induced BV-2 cells. Cells were stimulated by 1 μg/mL LPS with or without 18 for 24 h. **(A)** The protein expressions o f iNOS treated by different concentrations of 18 were determined by Western blot assay, **(B)** the expressions of COX-2 t reated by different concentrations of 18 were determined by Western blot assay. The data are represented as a mean ± S.D. from independent experiments performed in triplicate (*compared with the control, #compared with LPS, */^#^ *p* < 0.05, **/^##^ *p* < 0.01, and ***p < 0.001).

**FIGURE 7:**
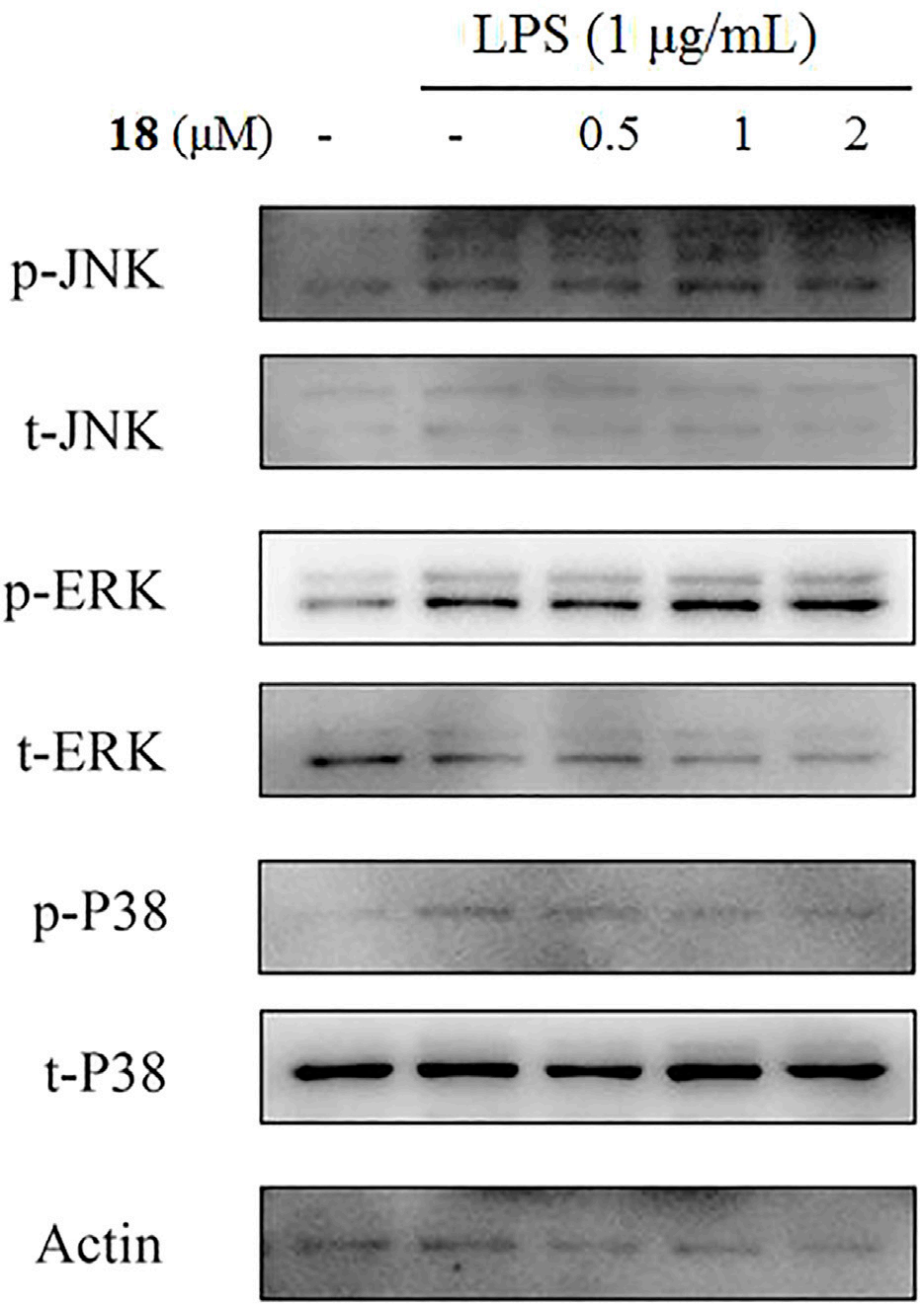
Ineffective analog **18** to regulate MAPK phosphorylation in LPS-stimulated BV-2 cells.

**FIGURE 8:**
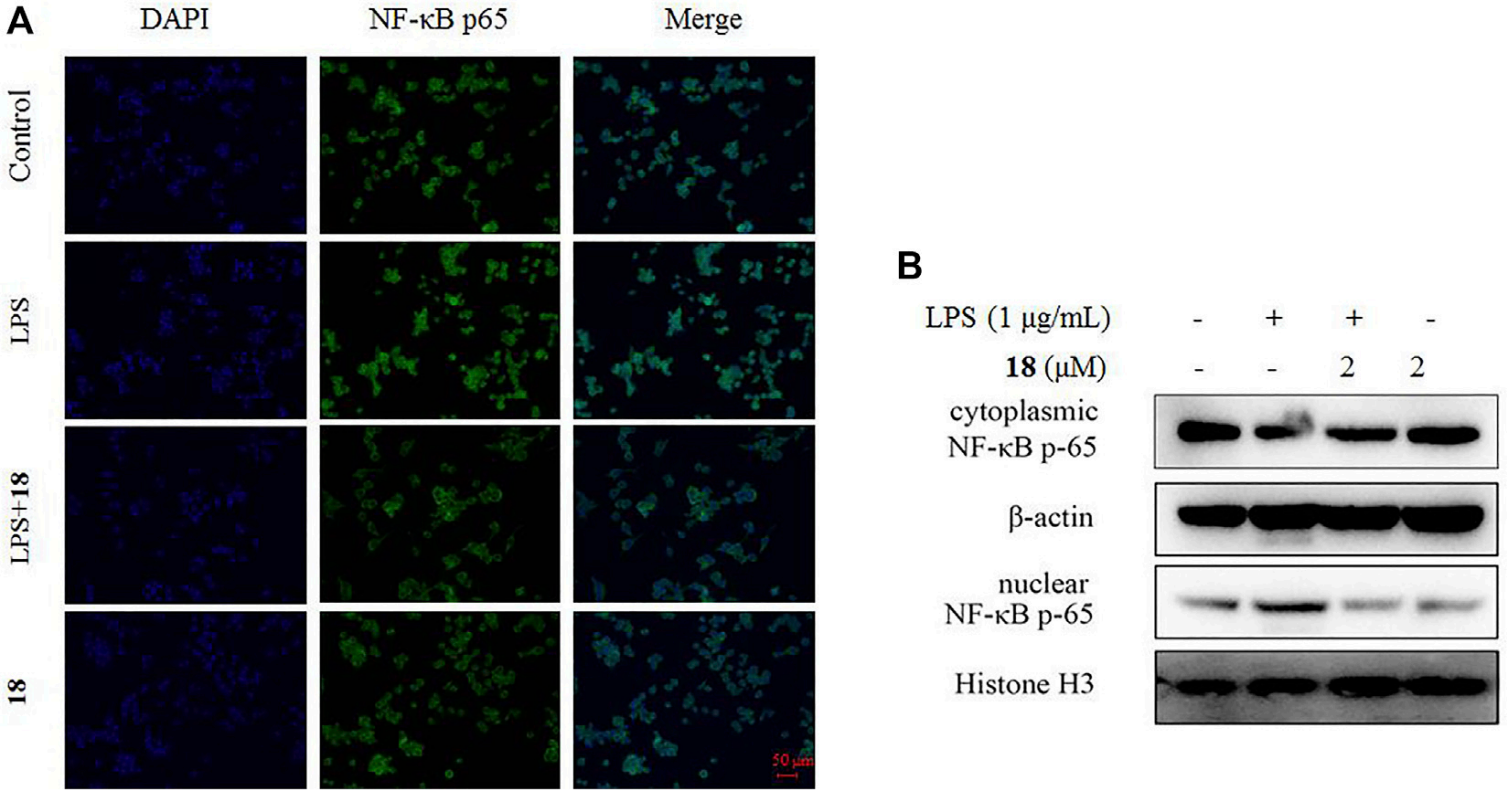
Effect of analog **18** on the nuclear translocation of NF-κB p65 in LPS-stimulated BV-2 cells. **(A)** BV-2 cells were stimulated with LPS (1 μg/ml) in the absence or presence of analog **18** (2 μM) for 3 h, followed by detection of the NF-kB p65 subunit translocation by immunocytochemistry. NF-κB p65 is shown in green, and DNA (DAPI nuclear staining) is shown in blue. Bars: 50 μm. **(B)** BV-2 cells were stimulated with LPS (1 μg/ml) in the absence or presence of **18** (2 μM) for 3 h, and NF-kB p65 levels in the nucleus and cytoplasm were determined by Western blot. Histone H3 and β-actin were used as endogenous controls for nuclear and cytoplasmic proteins, respectively. Values represent the mean ± SD of three independent experiments (*compared with the control, ^#^compared with LPS, */^#^ *p* < 0.05, **/^##^ *p* < 0.01, and ***p < 0.001).

The inducible nitric oxide synthase (iNOS) produced the signaling molecule NO as an inflammatory factor related to neurodegenerative diseases, and iNOS regulates the NO level during neuroinflammation (Herbert et al., 2006). Western blot detection revealed that **18** decreased the iNOS and the other inflammatory mediator cyclooxygenase-2 (COX-2) levels in LPS-induced BV-2 cells (Figure 6). The MAPK and NF-kB signaling pathways are the critical transcription factors which mediate the expression of pro-inflammatory genes (Lawrence et al., 2009; DiDonato et al., 2012; Choi et al., 2019). In BV-2 microglial cells, analog **18** slightly affected the phosphorylation of c-Jun NH_2_-terminal protein kinase (JNK), extracellular regulated protein kinases (ERK), and p38, which play key roles in the MAPK signaling pathway (Figure 7). However, the immunofluorescence and WB results revealed that **18** significantly downregulated the expression of the p65 level in the nucleus of LPS-stimulated BV-2 cells (Figure 8), suggesting the anti-neuroinflammatory effects of compound **18** related to the NF-kB signaling pathway.

## Conclusion

In this study, the bioinformatics approach in association with the molecular networking data provides an effective method to detect the metabolite patterns produced by marine-derived fungi, and a total of 18 new acorane-type sesquiterpenes are obtained from the deep-sea-derived fungus *P. bilaiae* F-28. Although the spiro[4.5]decane core of analogs from the F-28 strain is similar to that reported in the literature (Zhang et al., 2020), the distinct stereogenic centers of the analogs from this fungus to those derived from plants or the *Trichoderma* genus suggest the synthases with distinct stereospecific selections, implying a group of new synthases in this fungus. Bilaiacorenols A and B are structurally featured by the unique tricyclic acorane-type sesquiterpenes in nature. Analog **18** exhibits efficient reduction against the NO production in LPS-induced BV_2_ macrophages in a dose-dependent manner, and it abolished LPS-induced NF-κB in the nucleus of BV-2 microglial cells, along with the inhibition of iNOS and COX-2 at cellular levels. This study extends the chemical diversity of acorane-type sesquiterpenes and demonstrates that compound **18** shows potential for the development as an anti-neuroinflammation agent after structure optimization.

## Data Availability

The datasets presented in this study can be found in online repositories. The names of the repository/repositories and accession number(s) can be found in the article/Supplementary Material.

## References

[B1] Aoyagi A. Ito-Kobayashi M. Ono Y. Furukawa Y. Takahashi M. Muramatsu Y. (2008). Colletoic acid, a novel 11β-hydroxysteroid dehydrogenase type 1 inhibitor from *Colletotrichum gloeosporioides* SANK 21404. J. Antibiot. 61 (3), 136–141. 10.1038/ja.2008.122 18503191

[B2] Bian G. Hou A. Yuan Y. Hu B. Cheng S. Ye Z. (2018). Metabolic engineering-based rapid characterization of a sesquiterpene cyclase and the skeletons of fusariumdiene and fusagramineol from *Fusarium graminearum* . Org. Lett. 20, 1626–1629. 10.1021/acs.orglett.8b00366 29513542

[B3] Brock N. L. Huss K. Tudzynski B. Dickschat J. S. (2013). Genetic dissection of sesquiterpene biosynthesis by *Fusarium fujikuroi* . ChemBioChem 14, 311–315. 10.1002/cbic.201200695 23335243

[B4] Cheng X. Zeng Q. Ren J. Qin J. Zhang S. Shen Y. (2011). Sesquiterpene lactones from Inula falconeri, a plant endemic to the Himalayas, as potential anti-inflammatory agents. Eur. J. Med. Chem. 46, 5408–5415. 10.1016/j.ejmech.2011.08.047 21924800

[B5] Choi M. C. Jo J. Park J. Kang H. K. Park Y. (2019). NF-κB signaling pathways in osteoarthritic cartilage destruction. Cells 7, e734, 8, 10.3390/cells8070734 PMC6678954 31319599

[B6] Citron C. A. Riclea R. Brock N. L. Dickschat J. S. (2011). Biosynthesis of acorane sesquiterpenes by *Trichoderma* . RSC Adv. 1, 290–297. 10.1039/c1ra00212k

[B7] DiDonato J. A. Mercurio F. Karin M. (2012). NF-κB and the link between inflammation and cancer. Immunol. Rev. 246, 379–400. 10.1111/j.1600-065x.2012.01099.x 22435567

[B8] Guo R. Ren Q. Tang Y. Zhao F. Lin B. Huang X. (2020). Sesquiterpenoids from the roots of *Daphne genkwa* Siebold et Zucc. with potential anti-inflammatory activity. Phytochemistry 174, e112348. 10.1016/j.phytochem.2020.112348 32213358

[B9] Herbert T. Alexander R. M. (2006). Adipocytokines: Mediators linking adipose tissue, inflammation and immunity. Nat. Rev. Immunol. 6, 772–783. 10.1038/nri1937 16998510

[B10] Li G. Yang Z. Zhao P. Zheng X. Luo S. Sun R. (2011). Three new acorane sesquiterpenes from *Trichoderma* sp. YMF1.02647. Phytochem. Lett. 4, 86–88. 10.1016/j.phytol.2010.09.005

[B11] Lawrence T. (2009). The Nuclear Factor NF-κB Pathway in Inflammation, Cold Spring Harb. Perspect. Biol., 1, 6, e001651, 10.1101/cshperspect.a001651 PMC2882124 20457564

[B12] Liu Y. Li X. Meng L. Jiang W. Xu G. Huang C. (2015). Bisthiodiketopiperazines and acorane sesquiterpenes produced by the marine-derived fungus *Penicillium adametzioides* AS-53 on different culture media. J. Nat. Prod. 78, 1294–1299. 10.1021/acs.jnatprod.5b00102 26039736

[B13] Mendes S. A. C. Mansoor T. A. Rodrigues A. Armas J. B. Ferreira M. U. (2012). Anti-inflammatory guaiane-type sesquiterpenes from the fruits of Pittosporum undulatum. Phytochemistry 95, 308–314. 10.1016/j.phytochem.2013.06.019 23899690

[B14] Meng L. Li X. Li H. Wang B. (2020). Chermebilaenes A and B, new bioactive meroterpenoids from co-cultures of marine-derived isolates of Penicillium bilaiae MA-267 and P. chermesinum EN-480. Mar. Drugs 18, e339. 10.3390/md18070339 PMC7401264 32605151

[B15] Sandargo B. Michehl M. Praditya D. Steinmann E. Stadler M. Surup F. (2019). Antiviral meroterpenoid rhodatin and sesquiterpenoids rhodocoranes A–E from the wrinkled peach mushroom, *Rhodotus palmatus* . Org. Lett. 21 (9), 3286–3289. 10.1021/acs.orglett.9b01017 31008606

[B16] Wu S. Zhao L. Chen Y. Huang R. Miao C. Wang J. (2011). Sesquiterpenoids from the endophytic fungus *Trichoderma* sp. PR-35 of *Paeonia delavayi* . Chem. Biodivers. 8, 1717–1723. 10.1002/cbdv.201000236 21922660

[B17] Zhang J. Yi P. Xiong Y. Du C. Zhang Y. Yuan C. (2020). A new acorane sesquiterpenes of *Lysionotus pauciflorus* maxim. Biochem. Syst. Ecol. 93, e104165. 10.1016/j.bse.2020.104165

[B18] Zhang M. Zhao J. Liu J. Chen R. Xie K. Chen D. (2017). Neural anti-inflammatory sesquiterpenoids from the endophytic fungus *Trichoderma* sp. Xy24. J. Asian Nat. Prod. Res., 19, 651–658. 10.1080/10286020.2016.1251908 27835936

[B19] Zhu J. Liu L. Wu M. Xia G. Lin P. Zi J. (2021). Characterization of a sesquiterpene synthase catalyzing formation of cedrol and two diastereoisomers of tricho-acorenol from *Euphorbia fischeriana* . J. Nat. Prod. 84, 1780–1786. 10.1021/acs.jnatprod.1c00126 34014675

