# Supplementary material for: Acorane sesquiterpenes from the deep-sea derived Penicillium bilaiae fungus with anti-neuroinflammatory effects
Source: Front Chem. 2022 Nov 23;10:1036212. doi: 10.3389/fchem.2022.1036212 (PMC9727179; doi:10.3389/fchem.2022.1036212)

Acorane Sesquiterpenes from Deep-sea Derived *Penicillium bilaiae* Fungus with Anti-neuroinflammatory Effects

Supplementary Material

Table S1. Primer sequences of nine genes

primer	primer sequences (5'-3')
g1385-F	CATAGCTCACCACCGCACT
g1385-R	ATACACCTCTTGCGGGCAT
g4352-F	TCACCTTCAATCGTCACCGAG
g4352-R	AGCCCTGTGGTAAACATATCCG
g4713-F	CGAGGGCAAATTCTCATTTCCG
g4713-R	CCATTGACATTTGGCGAAGCA
g5873-F	TGTATCCCCGCTATTTGCCTT
g5873-R	ACCATGTTATGATTCACGCCCTC
g7166-F	TTCAACTTTTGCGCTATCCCT
g7166-R	AATCCTGCCACATTCGATGC
g8034-F	ATGTACCACTGCCTCGGTCA
g8034-R	GAACTCACAGCCACGACGAGA
g8904-F	ATGTCTTTCCAATCGCCACT
g8904-R	TTCTCAGCAAGCCATGAGGT
g3027-F	AGTTCCCCATCTATCCTCGAC
g3027-R	AGTACGGCCATTTTGTTTCCGAA
g10525-F	F CCGCTCGACTGATATTGCCAT
g10525-R	ATGACACCGATAAAGTCCGTTC

Table S2. Proposed functions of the proteins and their amino acid identity

**F-28_**	**Amino acids**	**Protein homologue**	**Putative function**	**Coverage%**	**Identity%**
g1385	525	KAE8378301.1	terpenoid cyclases	**86**	58.08
g4352	400	XP_025578872.1	terpenoid synthase	27	67.27
g4713	371	KGO72645.1	terpenoid synthase	98	79.4
g5873	300	XP_001265719.1	terpene synthase metal binding domain protei	87	54.2
g7166	485	XP_033424753.1	terpene cyclase	96	77.4
g8034	734	KAF7716703.1	terpene cyclase/mutase family member	99	84.86
g8904	188	KAF7715187.1	terpenoid cyclases	100	64.02
g3027	384	XP_016599181.1	terpenoid synthase	100	89.35
g10525	381	XP_024710217.1	terpenoid synthase	99	63.2

Table S3. ^{1}H NMR data of **1-7** (DMSO-d6)

	**1**	**2**	**3**	**4**	**5**	**6**	**7**
1	1.76 d (10.0)	1.21 d (4.8)	2.06 d (5.6)	2.00 dd (3.2,9.0)	2.30 d (9.2)	2.17 d (5.6)	2.31 d (9.6)
2	4.02 ddt (2.0,4.8, 10.0)	4.23, dt(4.8,6.0)	5.27 dd (5.6,7.6)	4.99 dt (3.6,9.0)	4.18 ddt (4.8,6.0,9.2)	4.00,ddt (4.8,5.6,10.8)	4.18 ddt (4.8, 9.0, 9.6)
3	1.00ddd (2.0,4.8,14.0)	1.32ddd (6.0,8.0,12.0)	1.13 d (14.5)	1.06ddd (2.4,3.6,14.8)	1.08ddd (2.8,4.8,16.0)	1.17 m	1.08ddd(2.8,4.8,16.0)
	2.43 dt (8.0,14.0)	1.89 dt (6.0,12.0)	2.19 dt (6.8,7.6,14.5)	2.41 dt (9.0,14.8)	2.33 td (9.2,16.0)	2.14 m	2.31ddd(8.4,9.0,16.0)
4	1.73 ddq (4.8,7.2,8.0)	1.61ddq (6.0,6.6,12.0)	1.81 dq (6.8,7.2)	1.80 ddq (2.4,7.2,9.0)	1.77 ddq (2.8,7.0,9.2)	2.04 m	1.77 ddq (2.8,7.2,8.4)
6	1.42 dd (2.0,12.0)	1.18 dd (3.6,12.0)	1.63 brd (12.0)	1.58 dd (3.0,12.0)	1.52 brdd (4.8,16.0)	1.47 dd (6.4, 13.2)	1.60 dd (4.4,16.0)
	1.57 dd (3.2,12.0)	1.83 dd (2.0,12.0)	2.63 brd (12.0)	1.89 dd (2.0,12.0)	1.87 ddq (2.4,16.0)	1.53 dd (5.6, 13.2)	1.89 ddt (2.4,16.0)
7	3.90 dd (2.0,3.2)	2.28 ddt (2.0,4.0,9.0)	5.24 brs	5.25 dd (2.5,3.0)	5.21 br	3.95 ddd (5.6,6.0,6.4)	5.46 br
9	5.38 br	5.42 dd (2.0,3.5)	3.90 ddd (4.4,5.0,6.5)	3.90 ddd (5.0,6.0,9.6)	4.00ddd (2.4,4.8,10.0)	5.34 brs	3.92ddd(2.4,4.8,10.0)
10	1.87 dq (2.5,14.0)	1.73 dd (3.5,14.0)	1.83 dd (5.0,12.0)	1.52 dd (9.6,13.2)	1.44 dd (10.0,12.8)	1.87 brs	1.45 dd (10.0,12.8)
	2.31 brd (14.0)	1.94 dd (2.0,14.0)	1.88 dd (4.4, 12.)	1.72 dd (5.0,13.2)	1.76 dd (2.4,12.8)		1.74 dd (2.4,12.8)
11				1.62 m			
12	3.16 dd (5.0,12.0)	1.34 dd (4.0,12.0)	2.99 dd (5.0, 12.0)	3.16 dt (5.0, 12.0)	4.68 br	4.60 br; 4.82 br	4.94 br, 4.98 br
	3.18 dd (5.0,12.0)	1.80 dd (9.0,12.0)	3.07 dd (5.0,12.0)	3.23 dt (5.0,12.0)	4.97 br		
13	1.12 s	1.10 s	1.21 s	0.86 d (7.0)	1.62 brs	1.64, s	1.75, s
14	0.92 d (7.2)	0.83 d (6.6)	0.97 d (7.2)	0.96 d (7.2)	1.00 d (7.0)	0.85 d (6.8)	1.00 d (7.2)
15	1.72 brs	3.78 dd (5.0,12.0)	1.63 s	1.62 s	1.74 s	1.72, s	3.89 brdd (5.2,13.2)
		3.79 dd (5.0,12.0)					3.92 brdd (5.2,13.2)
OH-2	5.12 d (4.8)	4.23 d (6.0)			4.49 d (6.0)	4.60 d (4.8)	4.52 d (6.0)
OH-9			4.55 d (6.5)	4.62 d (6.0)	4.15 d (4.8)	4.51 d (6.0)	4.51 d (4.8)
OH-11		3.91 s	4.09 s				
OH-12	4.83 t (5.0)		4.55 t (5.0)	4.54 t (5.0)			
OH-15		4.57 t (5.0)					4.50 t (5.2)
Ac			1.94 s	1.97 s			

Table S4. ^{1}H NMR data of **8-11** and **15-16** (DMSO-d6)

	**8**	**9**	**10**	**11**	**15**	**16**
1	2.19 d (9.6)	2.57 d (10.0)	2.67 d (9.2)	2.06 d (6.0)	2.59 d (9.0)	2.24 d (8.4)
2	4.35 ddt (6.0,8.4,9.6)	5.45ddd(4.4,8.8,10.0)	5.12 dt (4.0, 9.2)	4.13 ddt (4.8.5.2,6.0)	5.13 ddd (4.4,8.4,9.0)	4.12 tt (5.2,8.4)
3	0.92ddd(4.8,6.0,12.8)	0.92ddd(4.4,4.8,13.4)	1.17 dt (4.0, 14.0)	1.16 dt (4.8, 12.0)	1.08ddd(4.4,4.8,12.0)	1.05ddd (5.2,6.0,12.0)
	2.36 td (8.4, 12.8)	2.64 td (8.8,13.4)	2.48 dt (9.2, 14.0)	2.19 dt (5.2, 12.0)	2.50 td (8.4, 12.0)	2.29 td (8.4,12.0)
4	1.99 ddq (4.8,7.2,8.4)	2.05 ddq (4.8,7.2,8.8)	1.88 ddq (4.0,7.2,9.2)	1.76 ddq (4.8,5.2,7.2)	2.72 ddq (4.8,7.2,8.4)	2.29 ddq (6.0,7.2,8.4)
6	3.83 brd (6.0)	3.88 brd (6.0)	1.60 brd (12.0)	5.28 d (10.0)	1.17 dd (3.0,13.2)	1.13 dd (2.0,14.0)
			1.89 brd (12.0)		1.51 dd (3.6,13.2)	1.52 dd (4.0,14.0)
7	5.14 br	5.15 br	5.21 brs	5.27 d (10.0)	3.53 ddd (2.4,3.0,3.6)	3.51 dt (2.0,4.0)
9	3.97ddd(6.0,6.8,10.0)	4.01ddd(6.0,6.4,10.0)	3.94 ddd (4.0,6.4,8.8)	3.57 ddd (2.4,6.0,9.2)	3.49ddd(2.0,4.4,10.0)	3.50ddd (3.2,6.8,10.0)
10	1.39 dd (10.0,13.2)	1.42 dd (10.0,13.6)	1.46 dd (8.8,13.2)	1.43 t (9.2)	1.33 dd (2.0,10.0)	1.30 dd (3.2,12.0)
	1.76 dd (6.0,13.2)	1.82 dd (6.0,13.6)	1.73 dd (4.0,13.2)	1.64 dd (2.4,9.2)	1.74 t (10.0)	1.71 dd (10.0,12.0)
12	4.94 br, 4.69 br	4.78 br, 4.99 br	4.79 br, 5.21 br	4.64 br, 4.81 br	4.65 br, 4.94 br	4.62 br, 4.92 br
13	1.79 s	1.77 s	3.78 dd (5.6, 12.0)	1.66 s	1.72 s	1.73 s
			3.86 dd (5.6, 12.0)			
14	0.96 d (7.2)	1.00 d (7.2)	0.97 d (7.2)	0.96 d (7.2)	0.93 d (7.2)	0.93 d (7.2)
15	1.61 s	1.64 s	1.63 s	0.96 s	1.09 s	1.09 s
OH-2	4.27 d (6.0)			4.54 d (6.0)		4.40 d (5.2)
OH-6	4.50 d (6.0)	4.62 d (6.4)				
OH-7					4.56 d (2.4)	4.50 d (4.0)
OH-8				4.33 s	3.78 s	3.69 s
OH-9	4.56 d (6.8)	4.72 d (6.0)	4.63 d (6.4)	4.47 d (6.0)	4.08 d (4.4)	4.00 d (6.8)
OH-13			4.90 t (5.6)			
Ac-2		1.92 s	1.94, s		1.94 s	

Table S5. ^{13}C NMR data of **1-11** and **15-16** (DMSO-d6)

	**1**	**2**	**3**	**4**	**5**	**6**	**7**	**8**	**9**	**10**	**11**	**15**	**16**
1	53.1	60.0	54.7	49.4	60.4	63.5	60.5	62.9	58.2	52.7	64.0	58.8	62.6
2	71.3	71.2	76.7	76.0	72.6	74.4	72.6	72.7	77.3	77.4	71.8	77.4	73.3
3	39.6	41.8	40.0	38.6	40.7	41.9	40.7	43.7	40.5	38.2	41.3	38.3	41.5
4	41.9	41.0	38.4	37.1	37.2	39.5	37.2	32.4	33.0	37.5	40.3	37.6	37.6
5	40.0	41.4	48.1	46.7	47.3	45.8	47.0	52.5	52.0	46.1	49.1	48.0	47.7
6	35.0	31.1	31.4	30.7	31.4	40.0	31.0	68.9	69.2	30.8	131.6	33.2	33.7
7	69.2	29.3	121.9	121.0	121.3	66.3	120.0	129.1	128.4	121.3	133.3	73.9	74.7
8	135.8	144.0	136.4	136.9	136.7	136.2	140.7	136.4	136.6	136.5	71.7	74.0	73.3
9	125.4	118.3	66.9	66.4	66.5	121.7	64.1	66.6	66.5	66.4	71.8	70.5	69.5
10	36.7	33.5	41.3	38.5	40.0	31.2	37.2	39.5	40.0	37.9	36.1	35.0	35.6
11	77.2	69.4	73.3	33.0	143.4	146.0	143.2	143.7	142.1	147.4	143.0	142.9	144.3
12	72.5	41.4	69.6	66.7	113.2	113.4	113.6	114.0	114.8	109.7	113.4	115.1	114.4
13	17.7	30.3	24.4	15.0	24.3	23.1	24.3	22.5	22.4	64.6	22.5	23.8	24.5
14	18.4	14.0	16.8	17.7	18.7	15.6	18.7	20.2	19.9	17.4	17.5	18.0	18.8
15	21.2	63.2	19.2	19.3	19.3	19.9	61.5	18.9	18.6	19.4	21.9	21.6	23.6
Ac-2			21.3	21.1					20.9	21.0		21.4	
			170.0	170.0					170.0	173.0		170.1	

Table S6. ^{1}H and ^{13}C NMR data of **12-14** (DMSO-d6)

		**12**		**13**		**14**
	δ_C	δ_H	δ_C	δ_H	δ_C	δ_H
1	61.7	2.24 d (8.8)	58.0	2.50 d (9.0)	61.5	2.17 d (9.0)
2	72.7	4.12 ddt (4.0,6.0,8.8)	76.2	5.19dt(3.6, 9.0)	72.2	4.16 ddt (3.2,6.0,9.0)
3	41.0	1.07 dt (4.0,14.0)	37.7	1.17dt(3.6,12.0)	40.8	1.03ddd(3.2,4.8,12.0)
		2.30 dt (8.4,14.0)		2.40dt(9.0,12.0)		2.30 dt (9.0,12.0)
4	36.1	1.94 ddq (4.0,7.2,8.4)	35.3	2.02ddq(3.6,7.2,9.0)	35.5	1.90 ddq (4.8,7.2,9.0)
5	47.4		47.9		47.5	
6	25.4	1.25 m, 1.68 m	30.1	1.20 m, 1.62 m	30.7	1.15 dd (10.0,12.0)
						1.56 dd (4.0,12.0)
7	21.2	1.20, m; 1.35, m	23.5	1.12 m, 1.25 m	23.5	3.22ddt(4.0,6.0,12.0)
8	41.5	1.81 m	46.8	1.12ddt(4.0,6.0,10.0)	46.9	1.04 m
9	67.5	3.70 dt (4.0,10.0)	67.4	3.23 dt (3.0, 10.0)	67.6	1.53 m, 1.56 m
10	35.8	0.90 dt (3.0, 12.0)	41.2	1.23 dd (3.0, 12.5)	41.5	0.96 dd (10.0,12.0)
		1.17ddd(4.0,10.0,12.0)		1.52 dd (10.0,12.0)		1.19 dt (4.0, 12.0)
11	143.8		141.5		143.2	
12	114.1	4.62 brs, 4.96 brs	115.0	4.68 brs, 4.99 brs	114.2	4.63 brs, 5.00 brs
13	23.8	1.74 s	23.4	1.72 s	23.6	1.74 s
14	18.6	0.95 d (7.2)	18.1	0.98 d (7.2)	18.9	0.98 d (7.2)
15	57.4	3.29 dt (5.0, 12.0)	63.2	3.30 dt (6.0,12.0)	63.4	3.55 dt (5.0, 12.0)
		3.60 dt (5.0,12.0)		3.56 dt (6.0, 12.0)		3.56 dt (5.0, 12.0)
			20.9	1.95		
			170.1			
OH-2		4.48 d (6.0)				4.45 d (6.0)
OH-9		5.00 d (4.0)				4.40 d (6.0)
OH-15		4.22 t (5.0)		4.30 t (6.0)		4.29 t (5.0)

Table S7. ^{1}H and ^{13}C NMR data of **17** and **18** (DMSO-d6)

		**17**		**18**
1	62.1	2.03 d (4.2)	61.5	2.17 d (9.6)
2	75.2	3.96 ddt (4.2,6.0,12.6)	72.2	4.16 ddt (2.0,6.0,9.6)
3	41.6	1.23 dt (6.6, 12.6)	40.8	1.07ddd (2.0,4.8,12.0)
		2.10 dt (6.6,12.6)		2.27 dt (9.0,12.0)
4	41.8	1.89ddq(6.6,7.2,12.6)	35.6	1.91ddq(4.0,7.2,9.0)
5	47.0		47.4	
6	40.9	1.64 dd (10.0,13.0)	30.2	1.01 dt (4.0,12.0)
		1.74 dd (4.8,13.0)		1.19 dt (4.0,12.0)
7	71.4	3.37ddt(4.8,6.0,10.0)	24.3	1.43ddd(2.4,7.8,12.0)
				1.66 dt (4.0,12.0)
8	52.5	2.31 dq (6.0)	51.7	1.81 dt (2.4,12.0)
9	211.2		67.3	3.57ddt(3.0,6.0,12.0)
10	47.5	2.22 d (13.0)	40.7	1.15 t (12.0)
		2.28 d (13.0)		1.60 dd (3.0,12.0)
11	143.7		143.0	
12	113.9	4.61 brs, 4.91 brs	114.5	4.63 brs, 4.98 brs
13	22.6	1.69 s	23.7	1.73 s
14	13.9	0.89 d (7.2)	18.8	1.00 d (7.2)
15	11.0	0.93 d (6.0)	176.3	
OH-2		4.73 d (6.0)		4.47 d (6.0)
OH-7		4.91 d (6.0)		
OH-9				4.63 d (6.0)

Fig. S1. ^{1}H NMR spectrum of **1** (400 MHz, DMSO-d6)

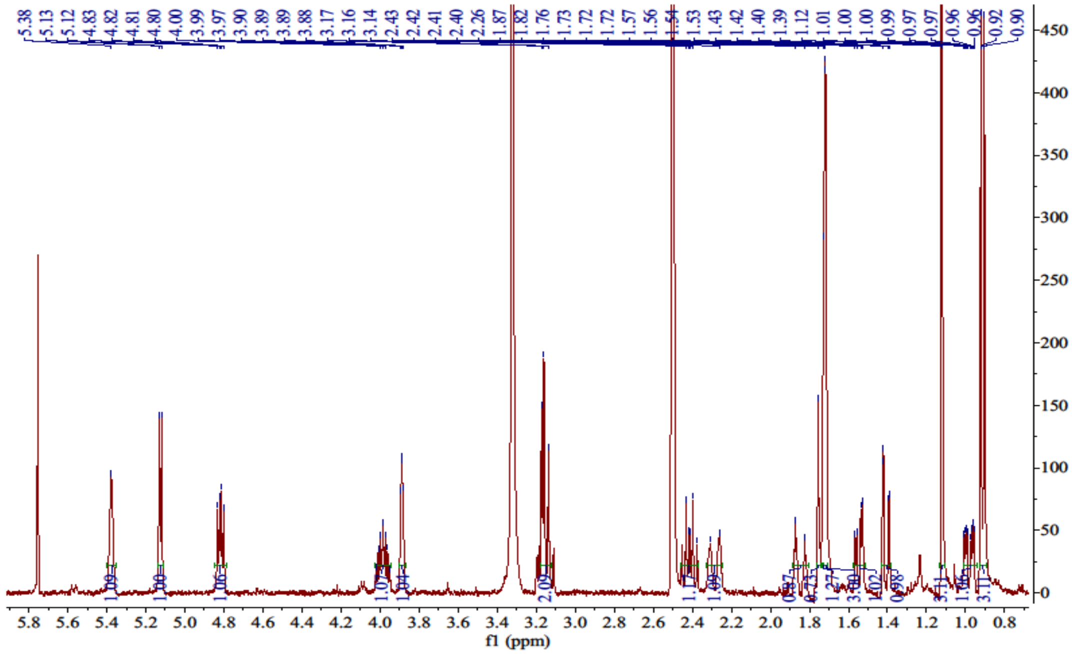

Fig. S2. ^{13}C NMR (APT) spectrum of **1** (100 MHz, DMSO-d6)

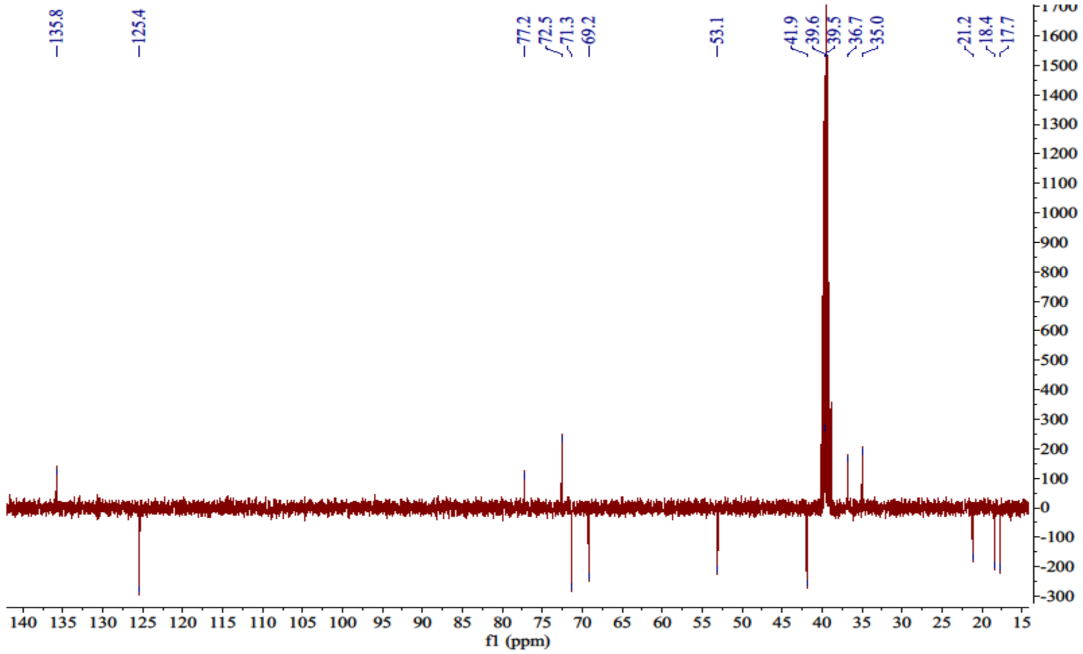

Fig. S3. ^{1}H-^{1}H COSY spectrum of **1**

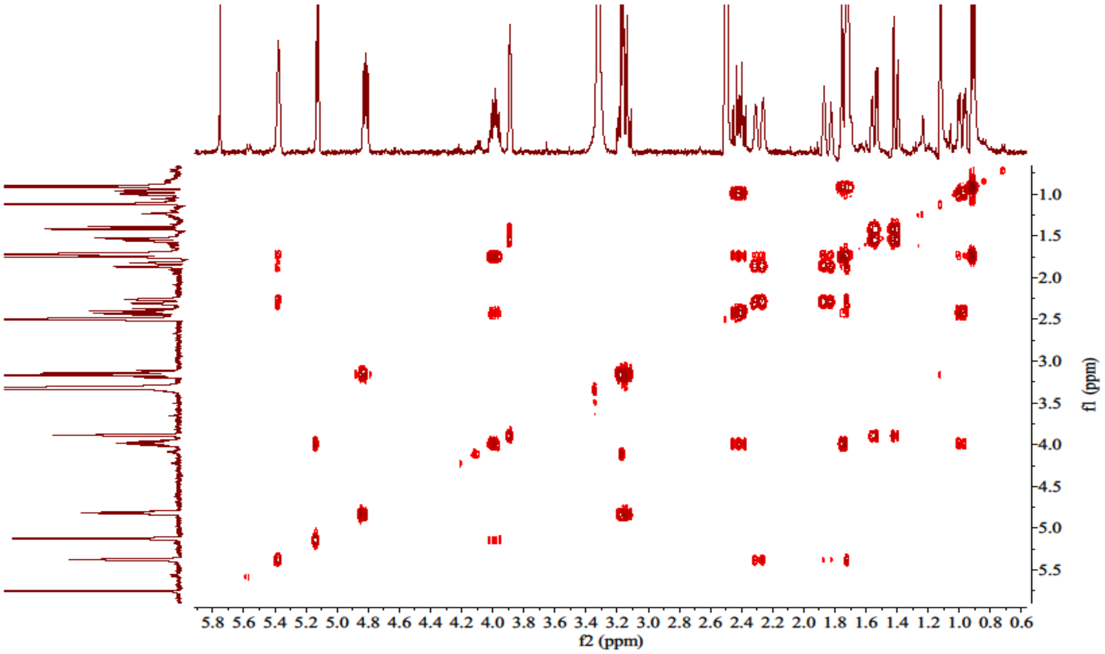

Fig. S4. HSQC spectrum of **1**

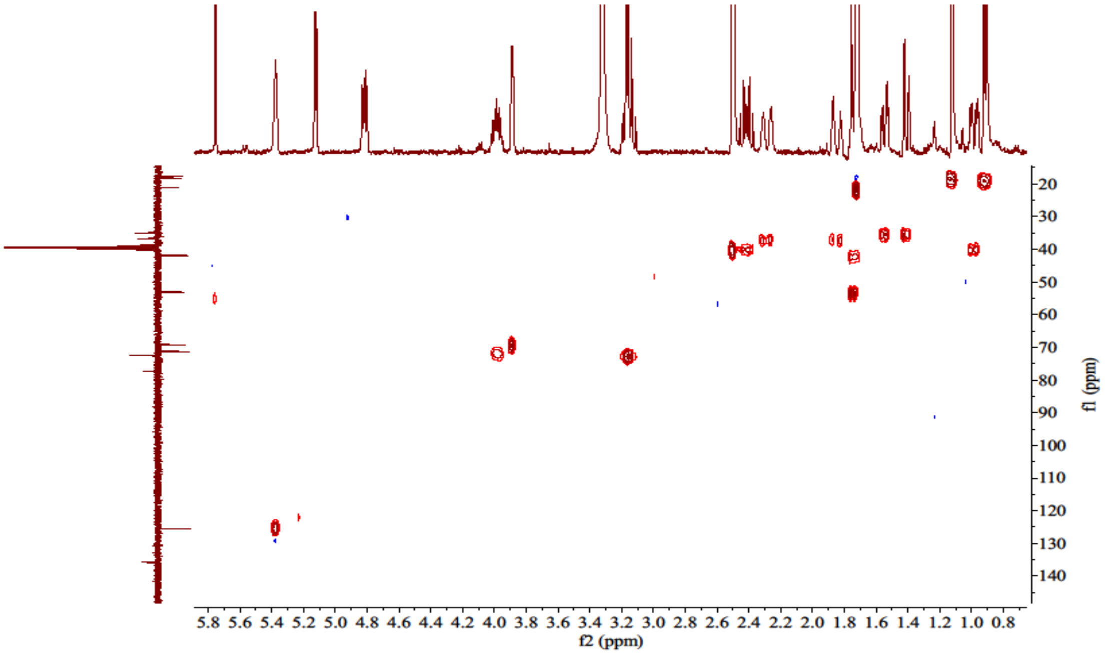

Fig. S5. HMBC spectrum of **1**

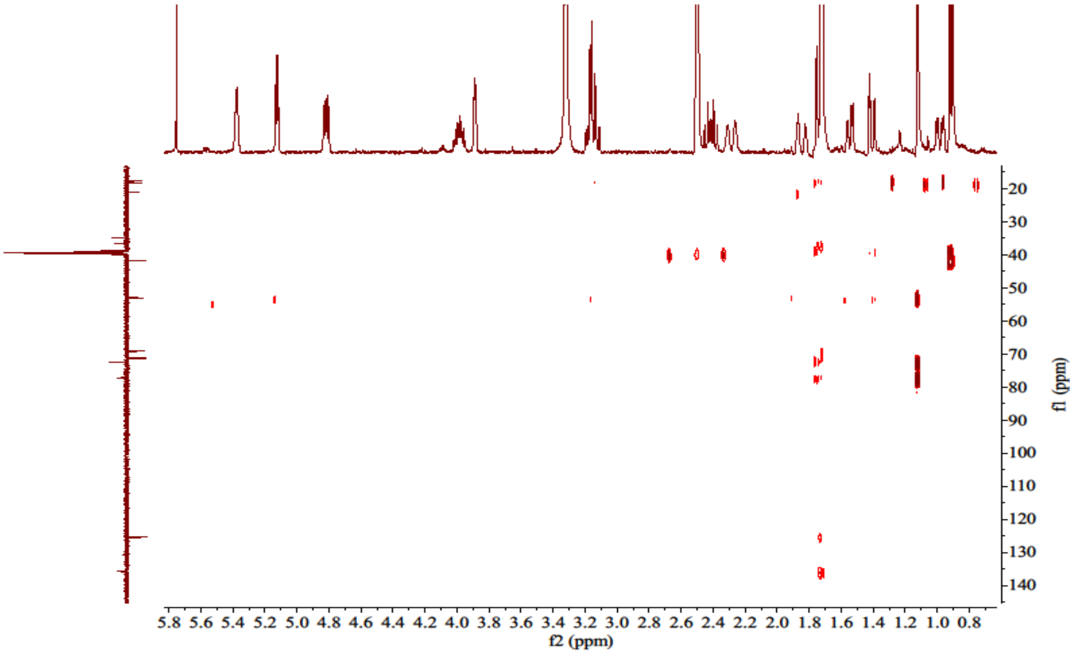

Fig. S6. NOESY spectrum of **1**

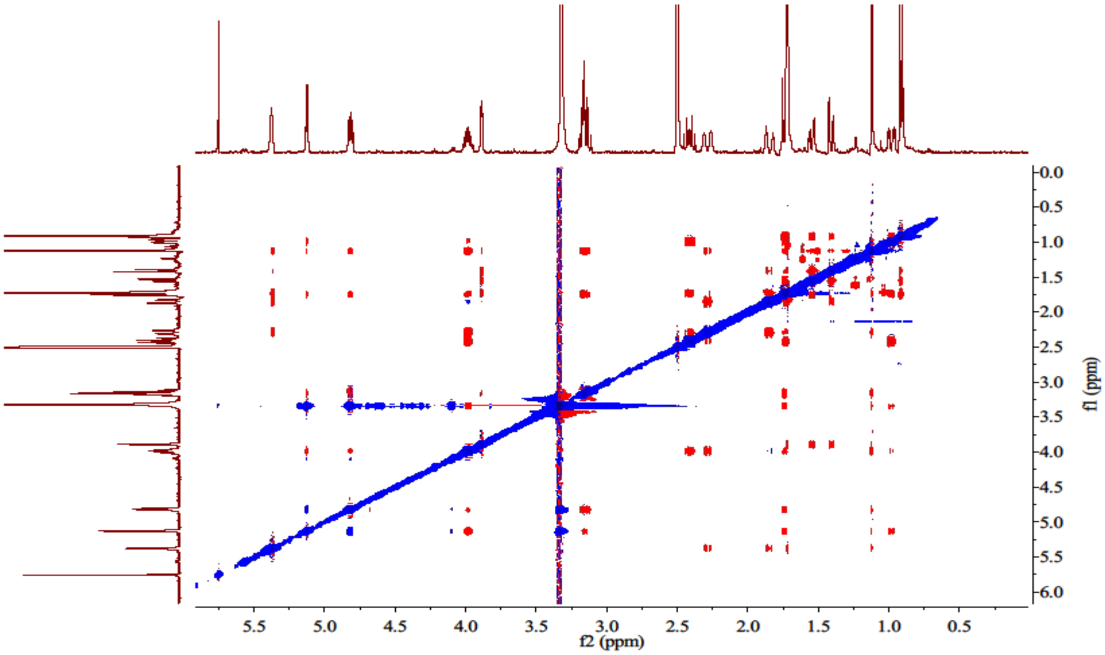

Fig. S7. HRESIMS spectrum of **1**

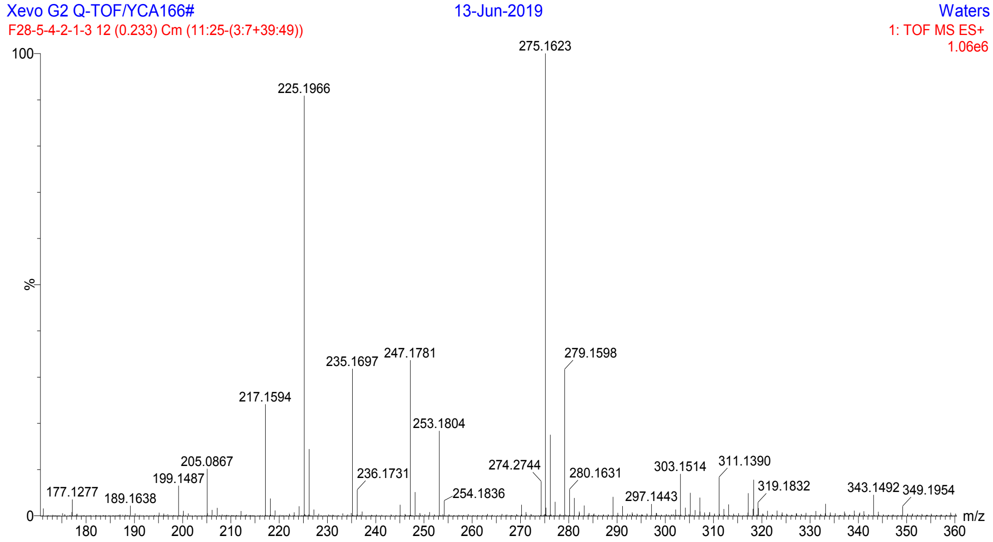

Mass	Calc. Mass	mDa	PPM	DBE	i-FIT	Norm	Conf(%)	Formula
275.1623	275.1623	0.0	0.0	3.5	503.0	n/a	n/a	C15 H24 O3 Na

Fig. S8. IR spectrum of **1**

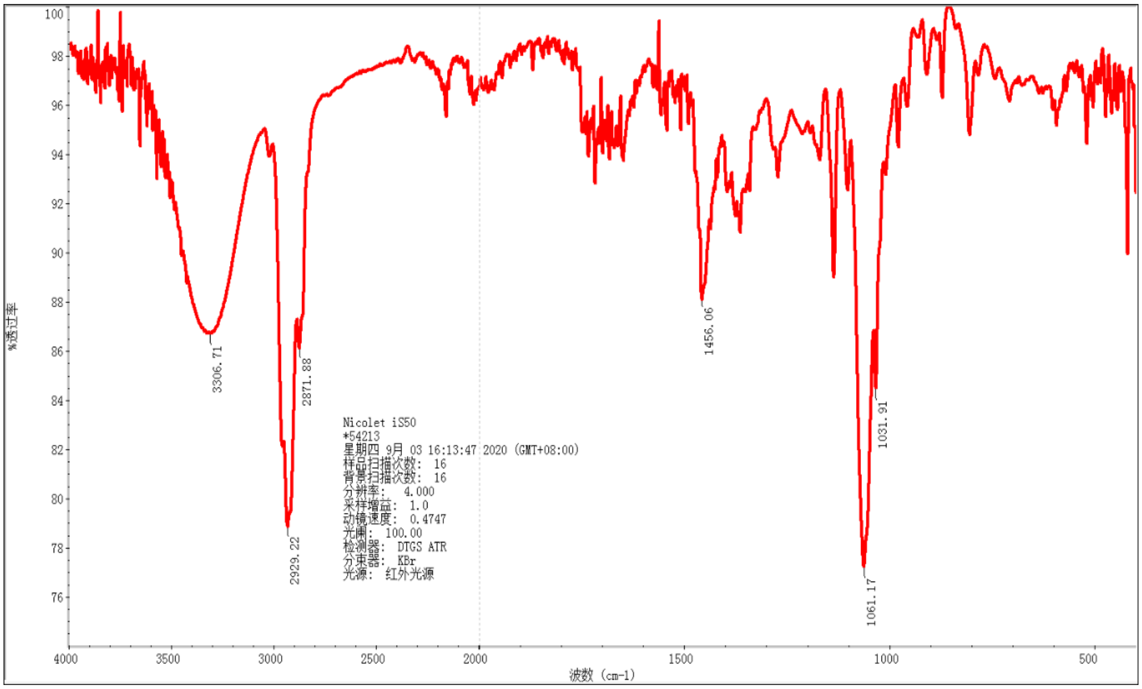

Fig. S9. UV spectrum of **1**

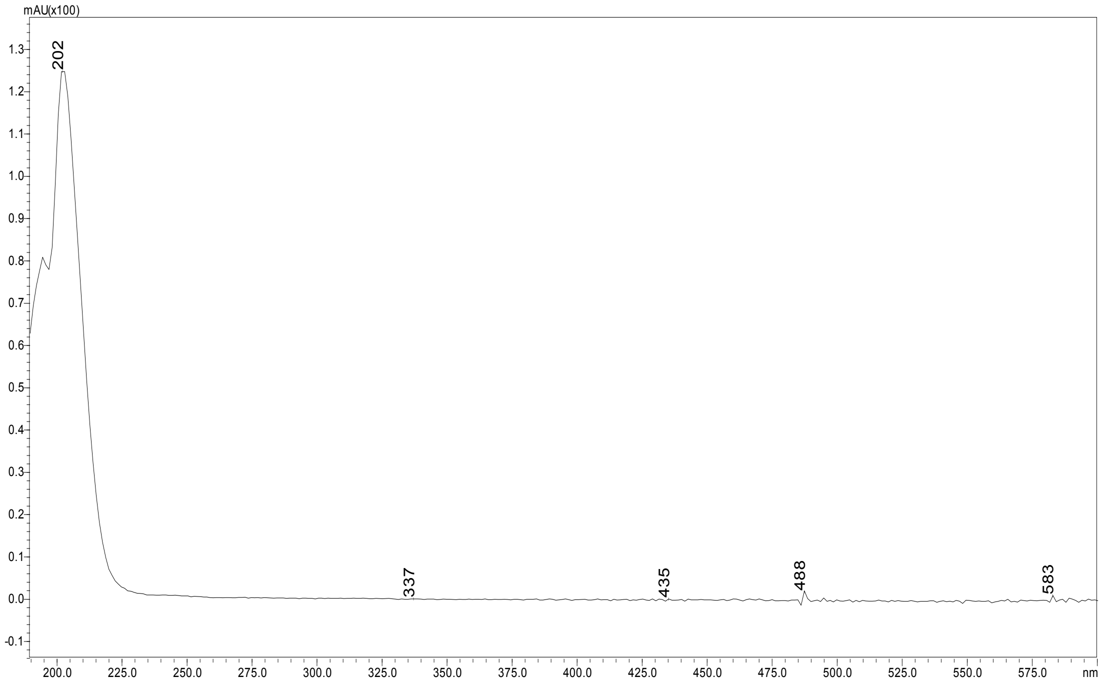

Fig. S10. ^{1}H NMR spectrum of **2** (400 MHz, DMSO-d6)

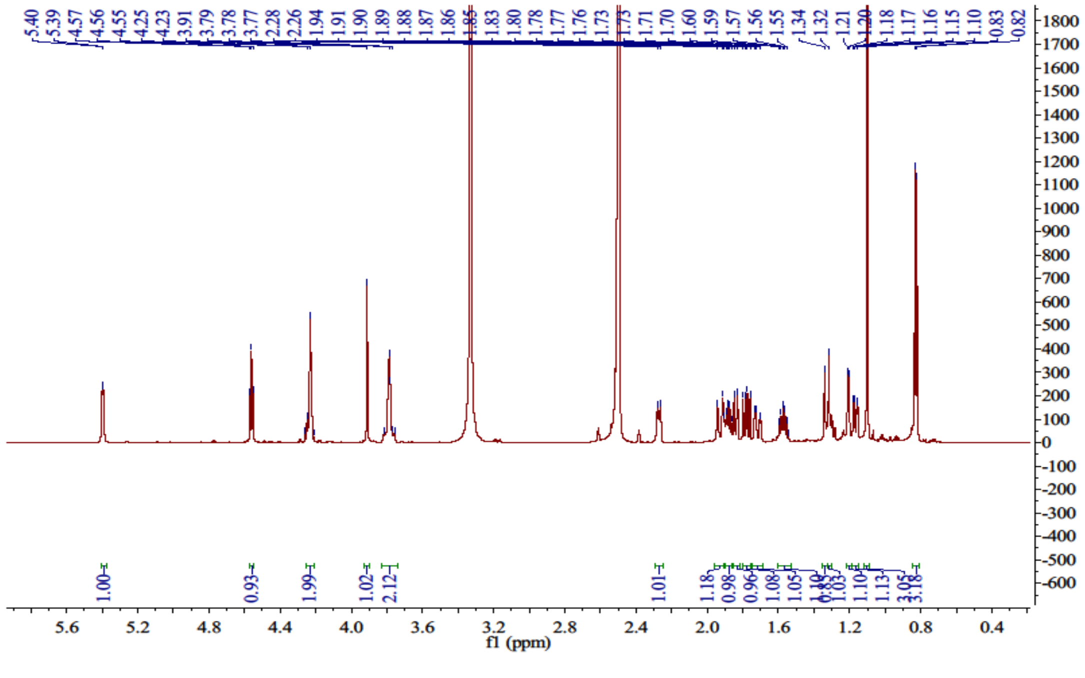

Fig. S11. ^{13}C NMR (APT) spectrum of **2** (100 MHz, DMSO-d6)

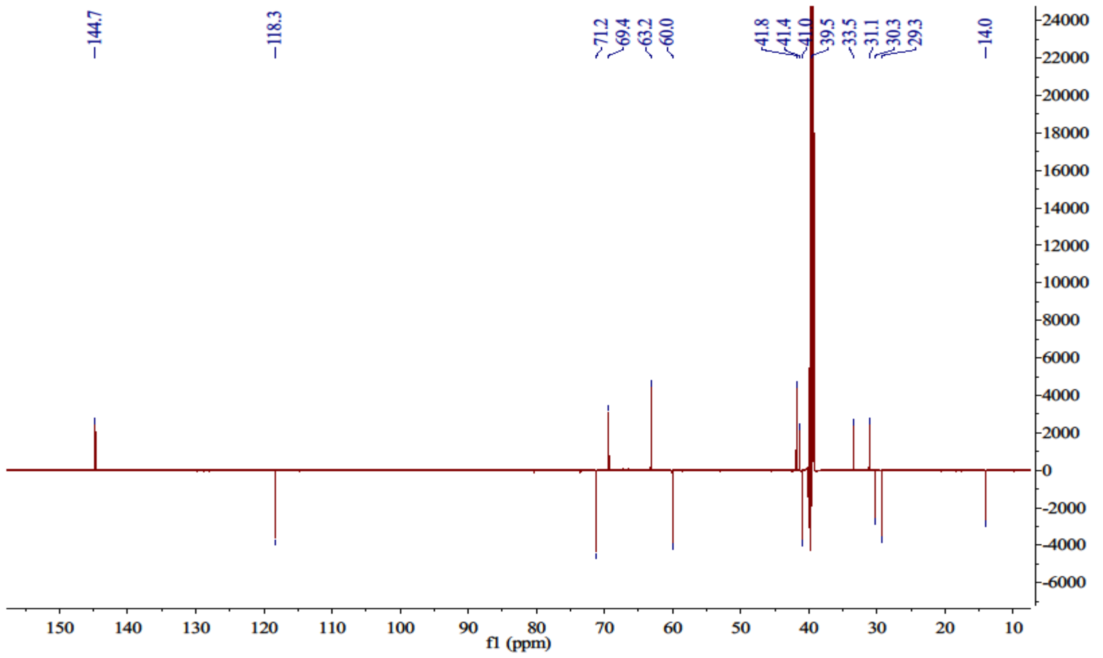

Figure S12. ^{1}H-^{1}H COSY spectrum of **2**

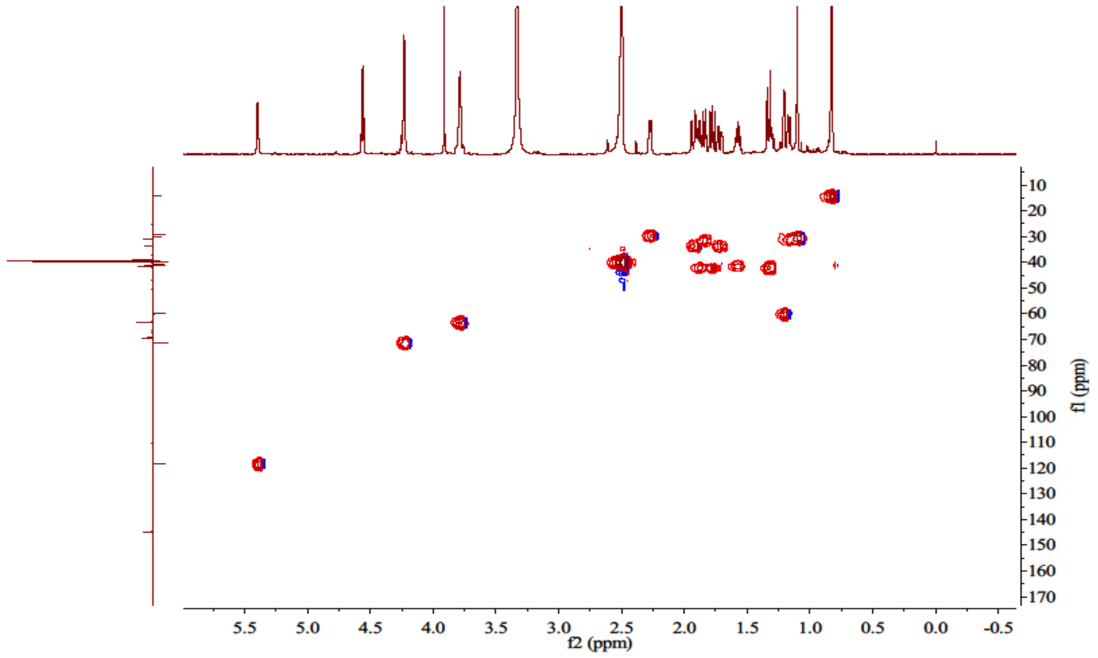

Fig. S13. HSQC spectrum of **2**

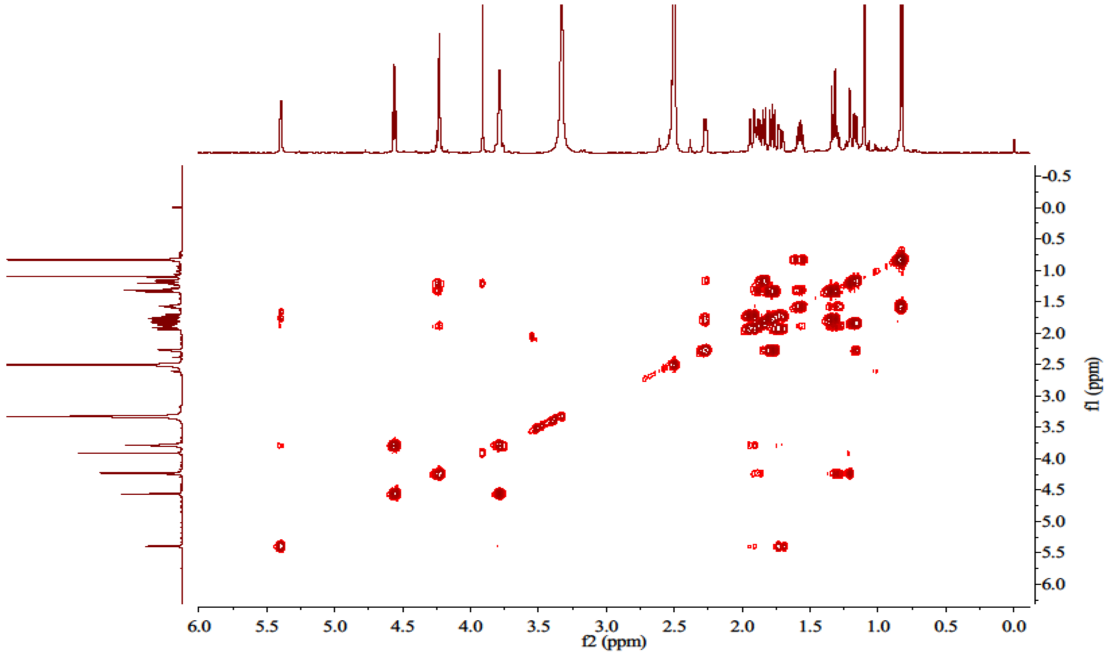

Fig. S14. HMBC spectrum of **2**

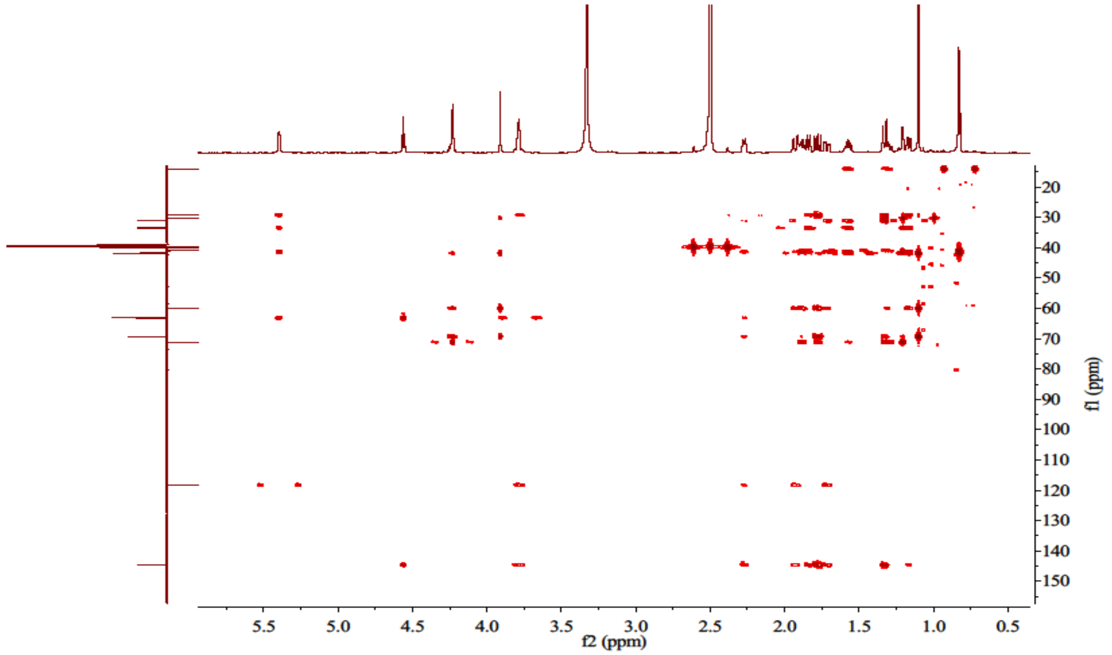

Fig. S15. NOESY spectrum of **2**

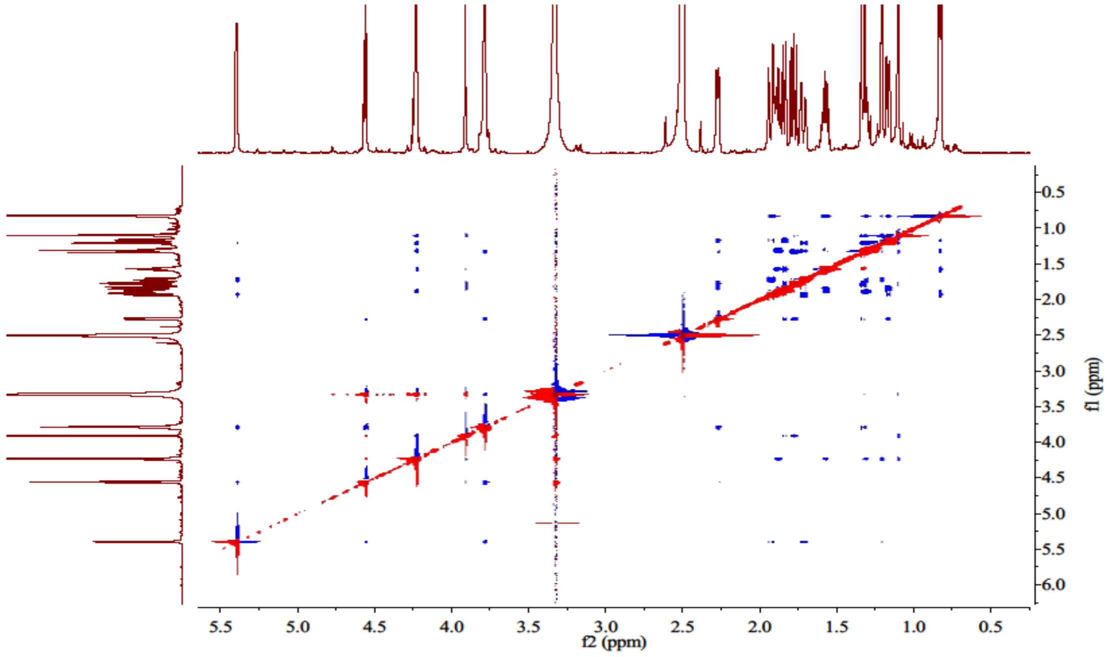

Fig. S16. HRESIMS spectrum of **2**

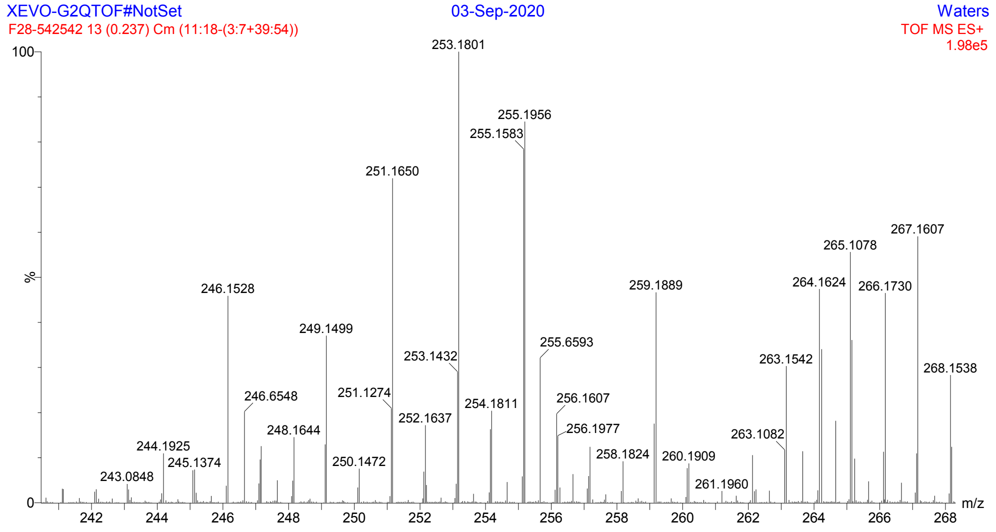

Mass	Calc. Mass	mDa	PPM	DBE	i-FIT	Norm	Conf(%)	Formula
253.1801	253.1804	-0.3	-1.2	3.5	607.4	n/a	n/a	C15 H25 O3

Fig. S17. IR spectrum of **2**

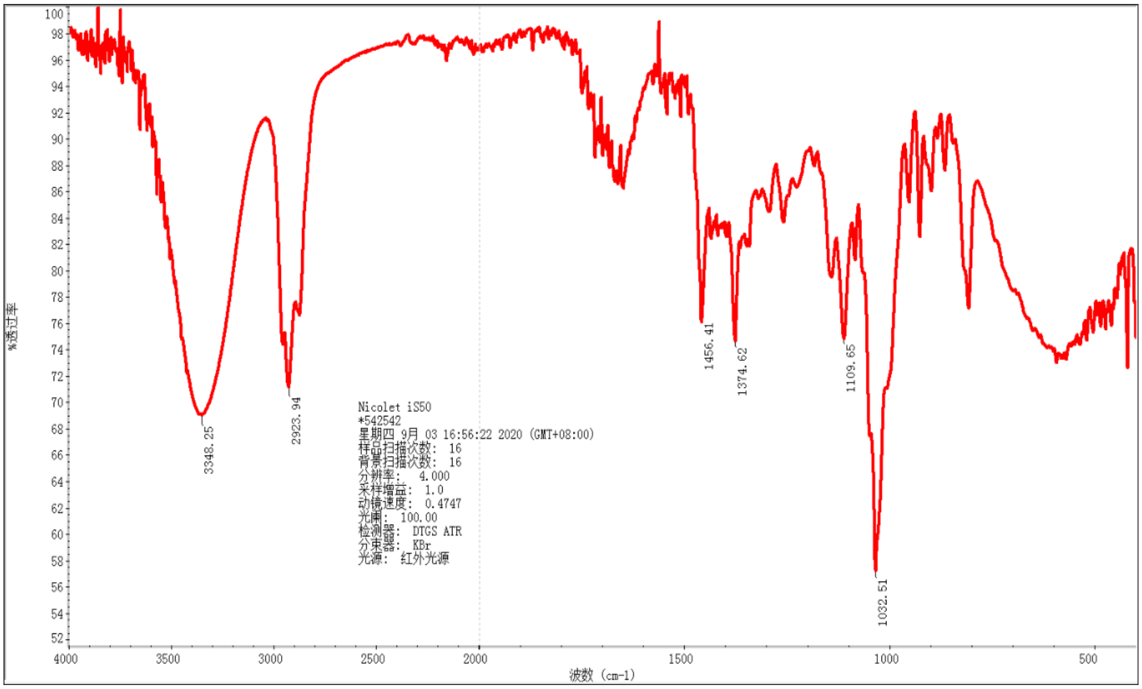

Fig. S18. UV spectrum of **2**

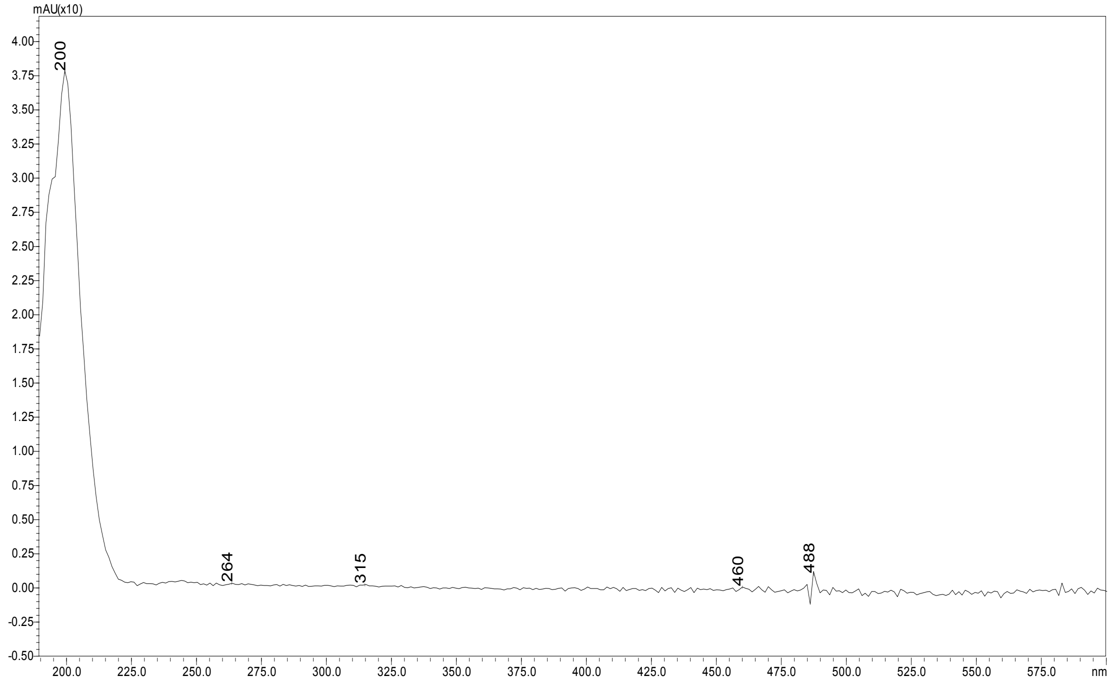

Fig. S19. ^{1}H NMR spectrum of **3** (400 MHz, DMSO-d6)

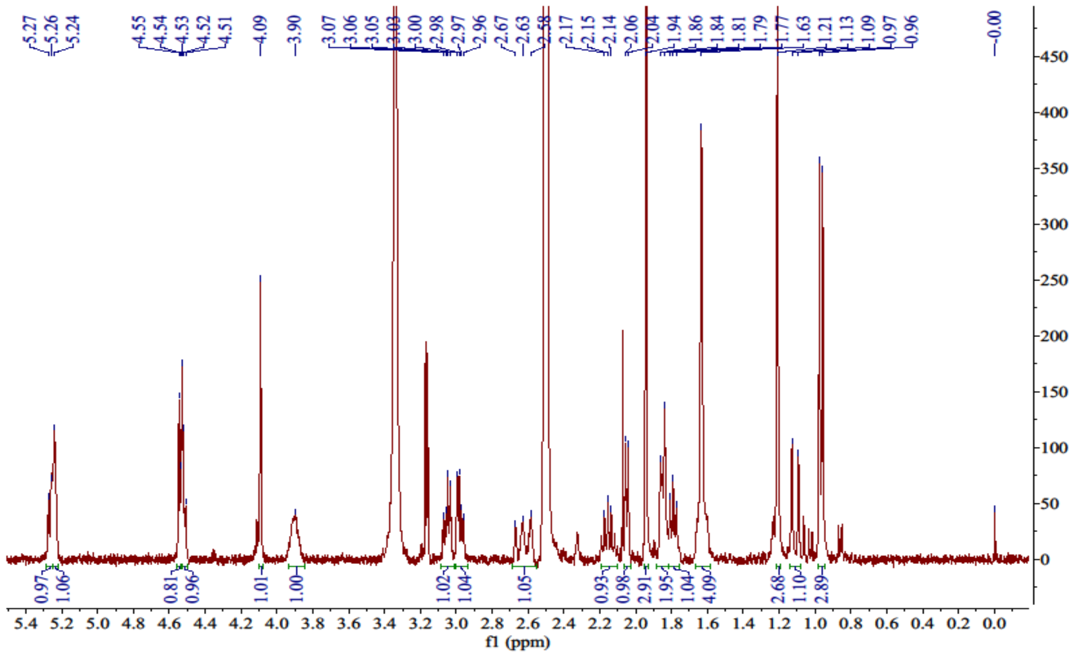

Fig. S20. ^{13}C NMR (APT) spectrum of **3** (100 MHz, DMSO)

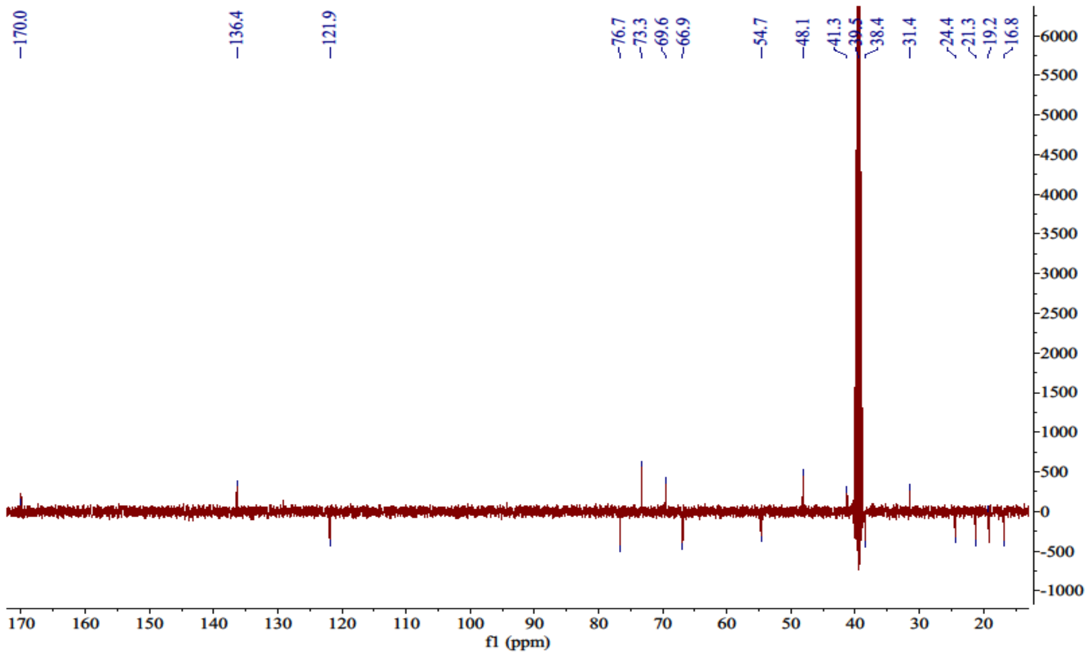

Fig. S21. ^{1}H-^{1}H COSY spectrum of **3**

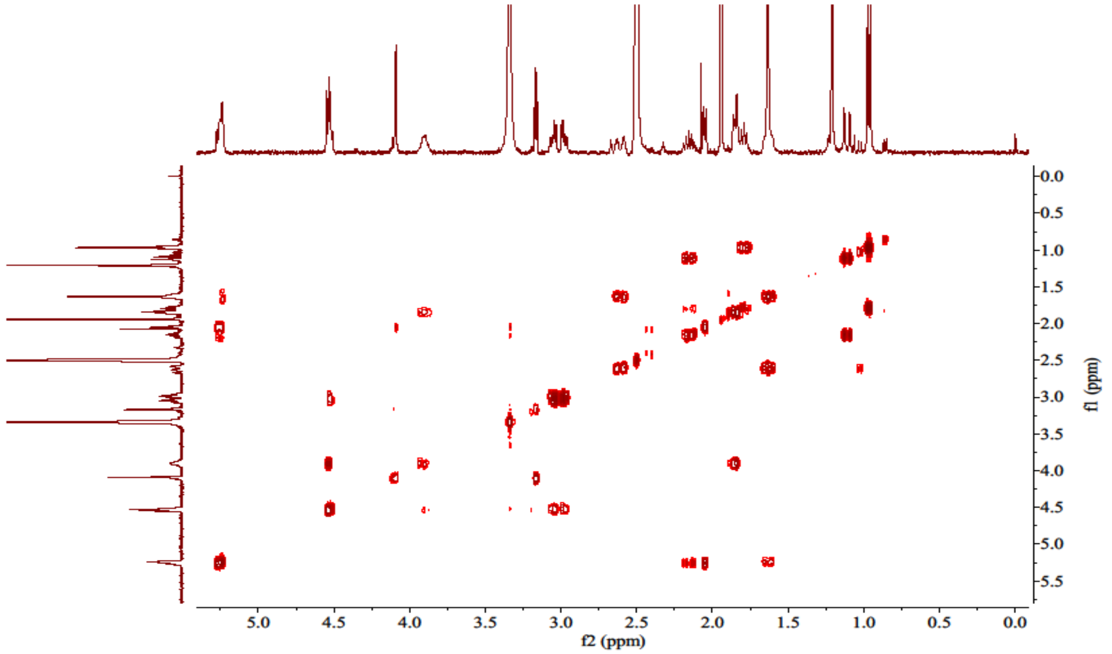

Fig. S22. HSQC spectrum of **3**

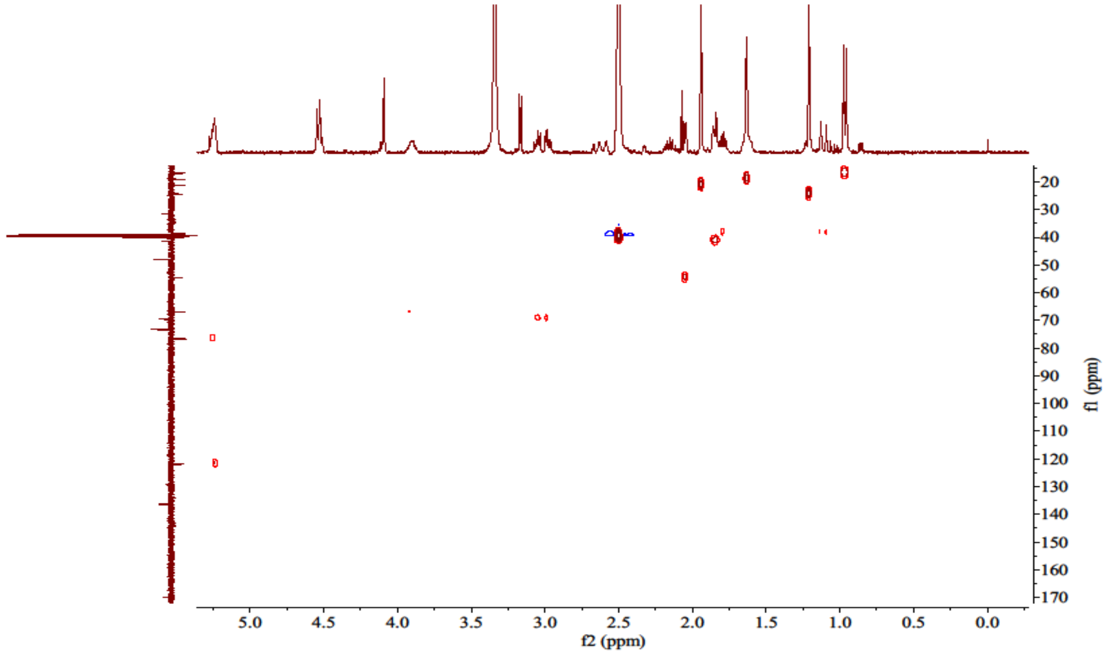

Fig. S23. HMBC spectrum of **3**

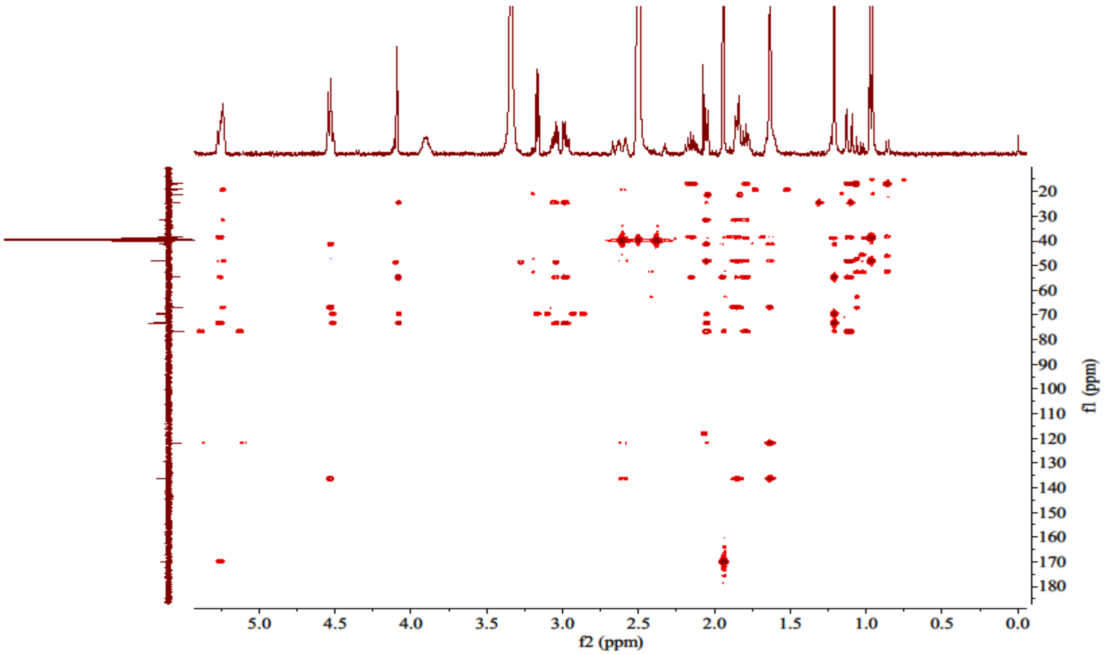

Fig. S24. NOESY spectrum of **3**

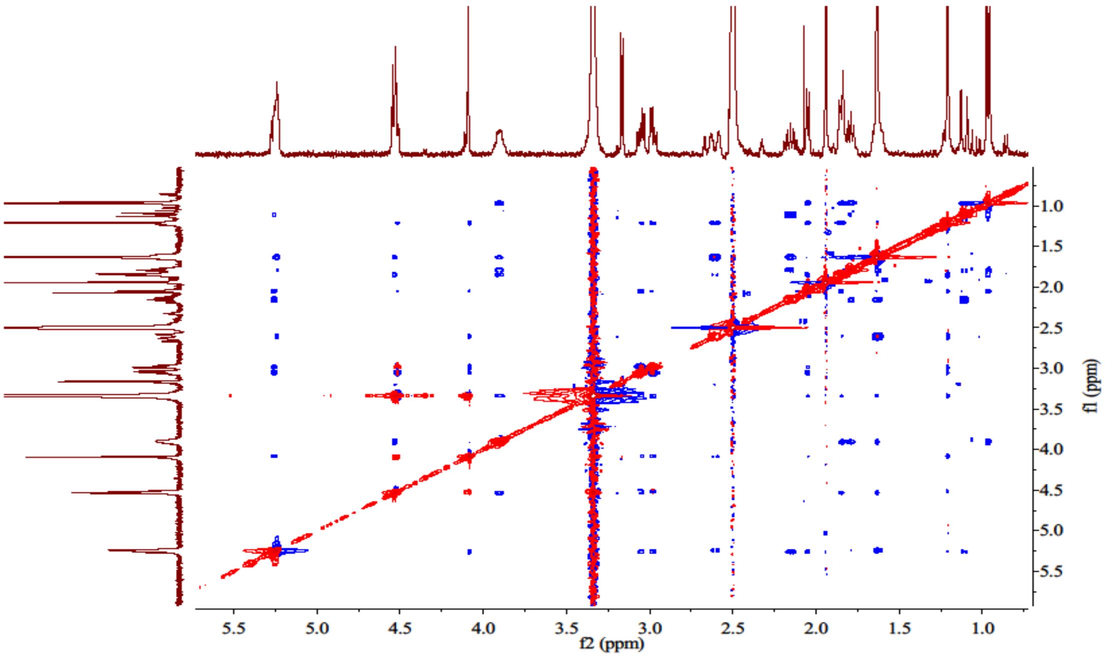

Fig. S25. HRESIMS spectrum of **3**

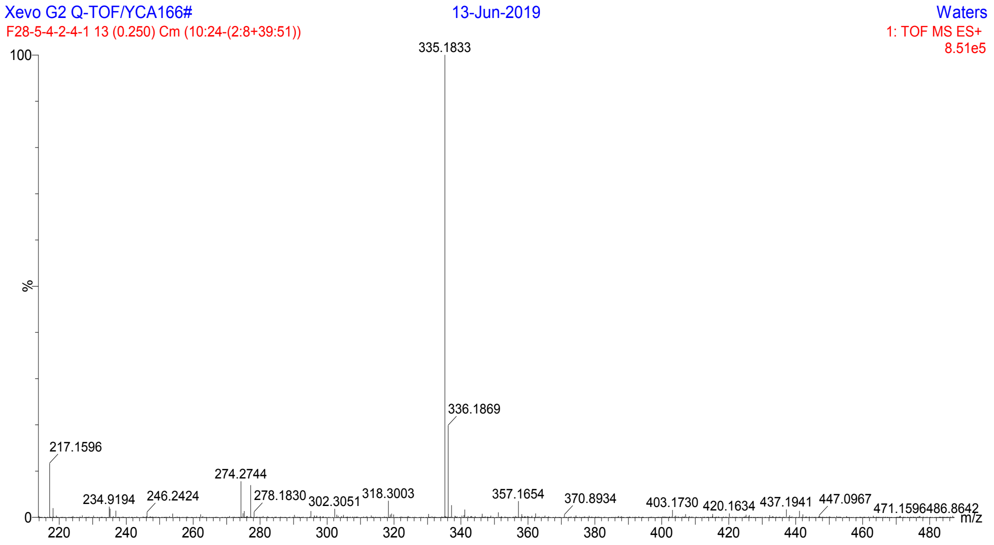

Mass	Calc. Mass	mDa	PPM	DBE	i-FIT	Norm	Conf(%)	Formula
335.1833	335.1834	-0.1	-0.3	3.5	322.4	0.050	95.13	C17 H28 O5 Na

Fig. S26. IR spectrum of **3**

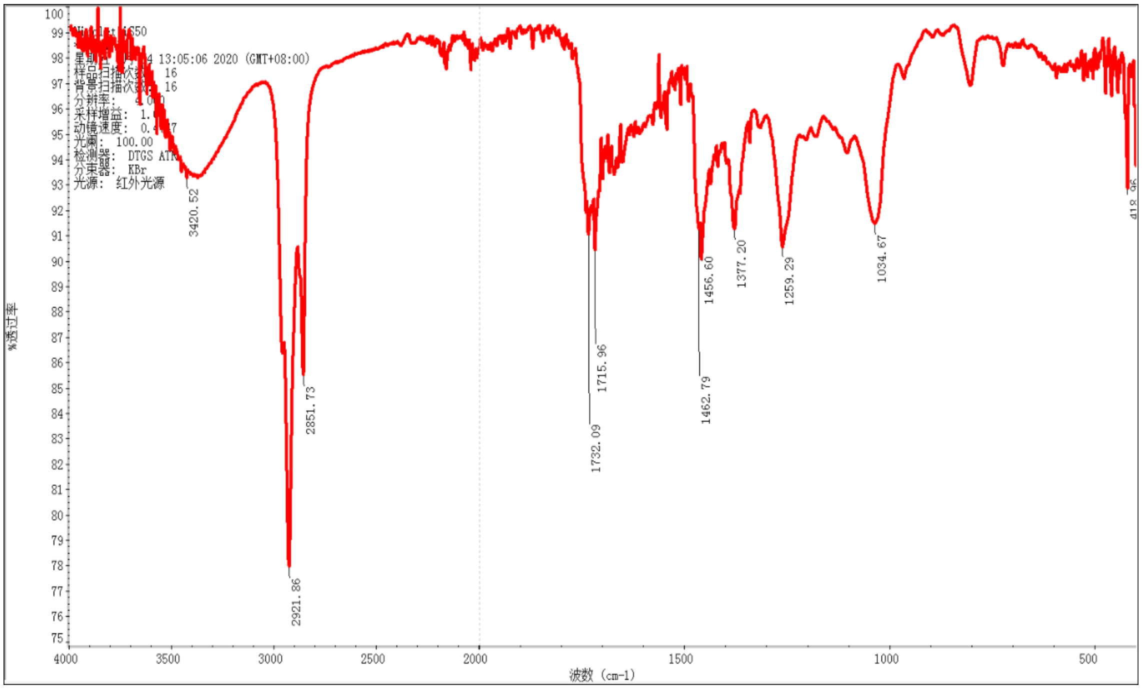

Fig. S27. UV spectrum of **3**

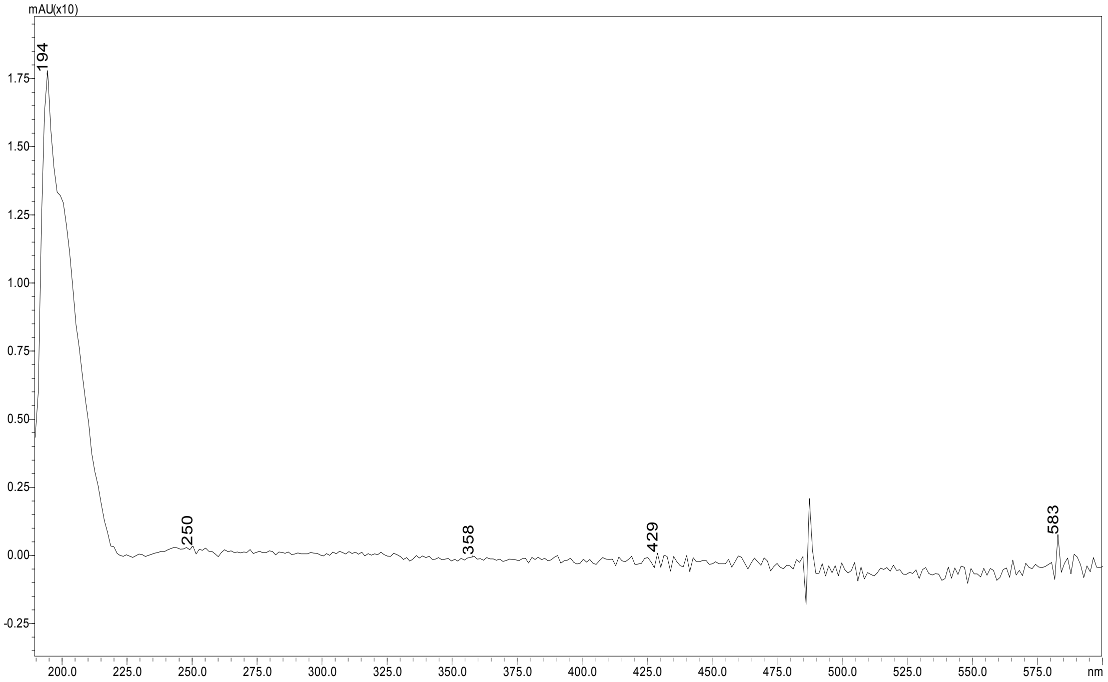

Fig. S28. ^{1}H NMR spectrum of **4** (400 MHz, DMSO-d6)

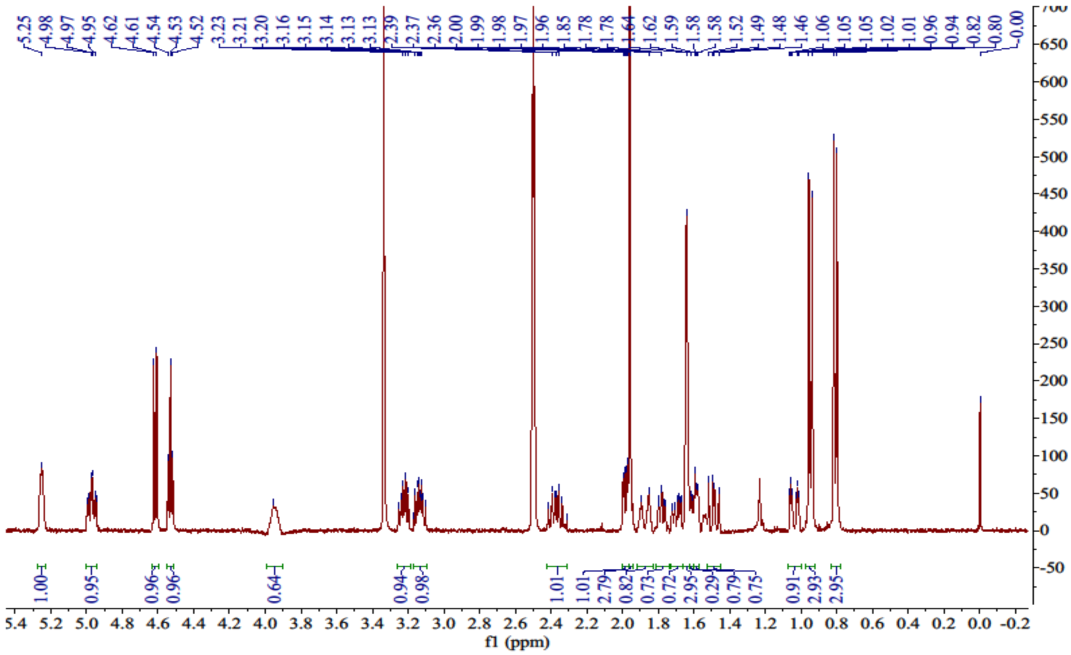

Fig. S29. ^{13}C NMR (APT) spectrum of **4** (100 MHz, DMSO-d6)

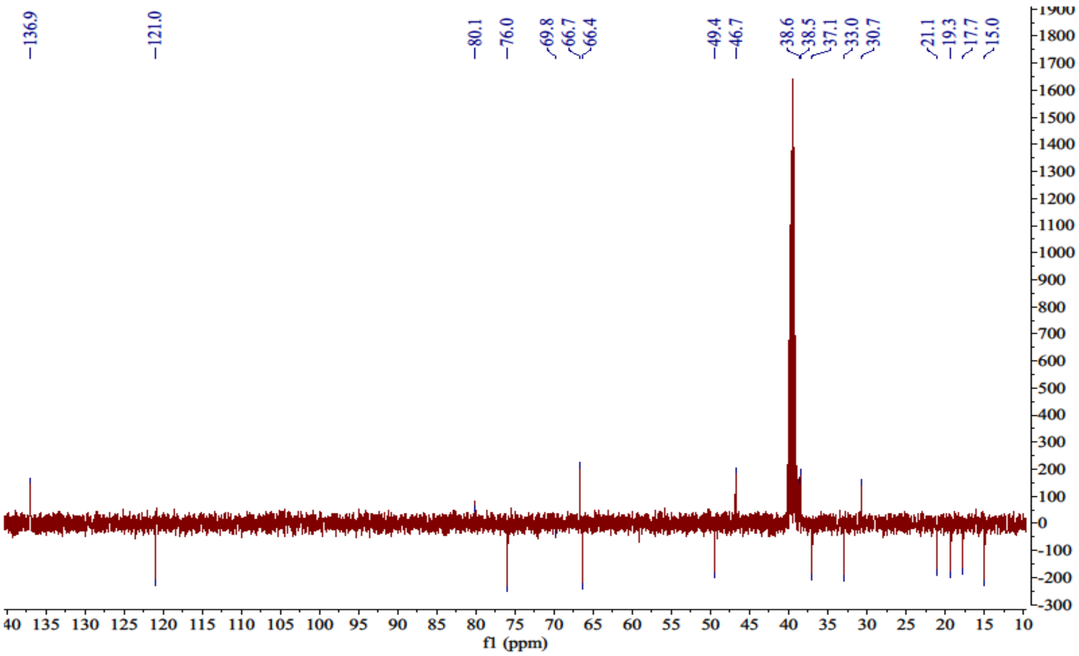

Fig. S30. ^{1}H-^{1}H COSY spectrum of **4**

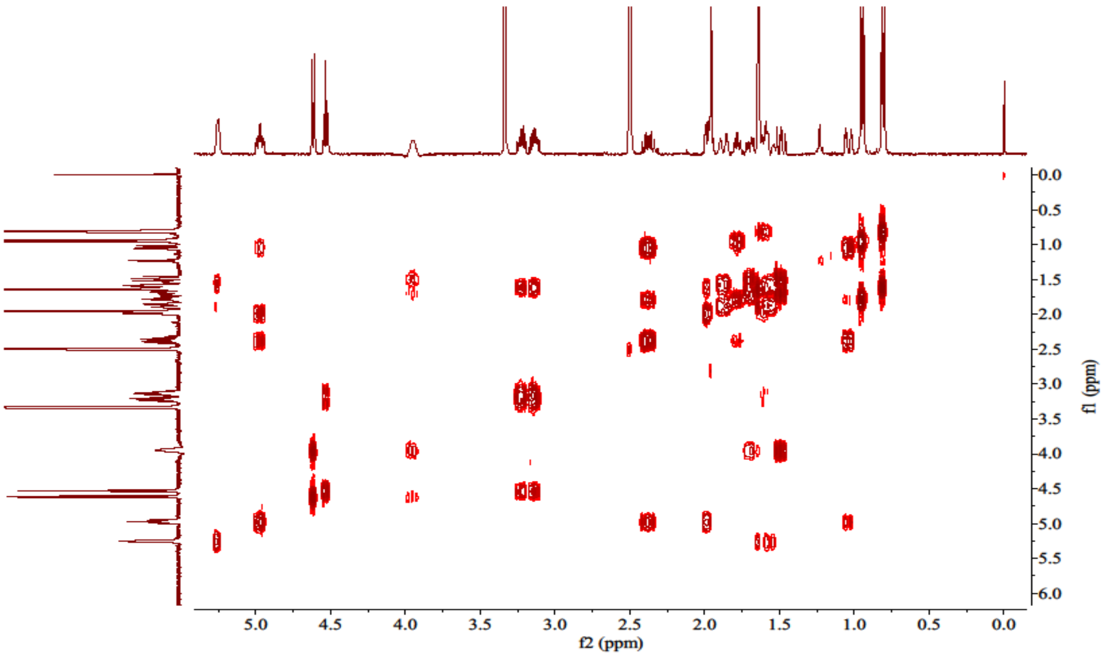

Fig. S31. HSQC spectrum of **4**

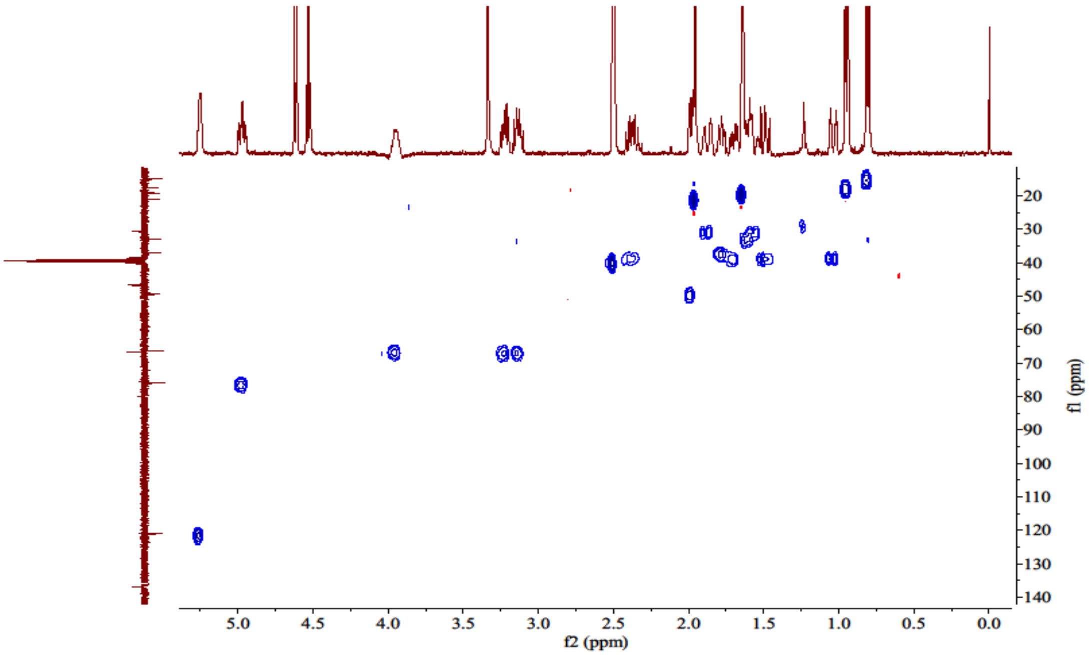

Fig. S32. HMBC spectrum of **4**

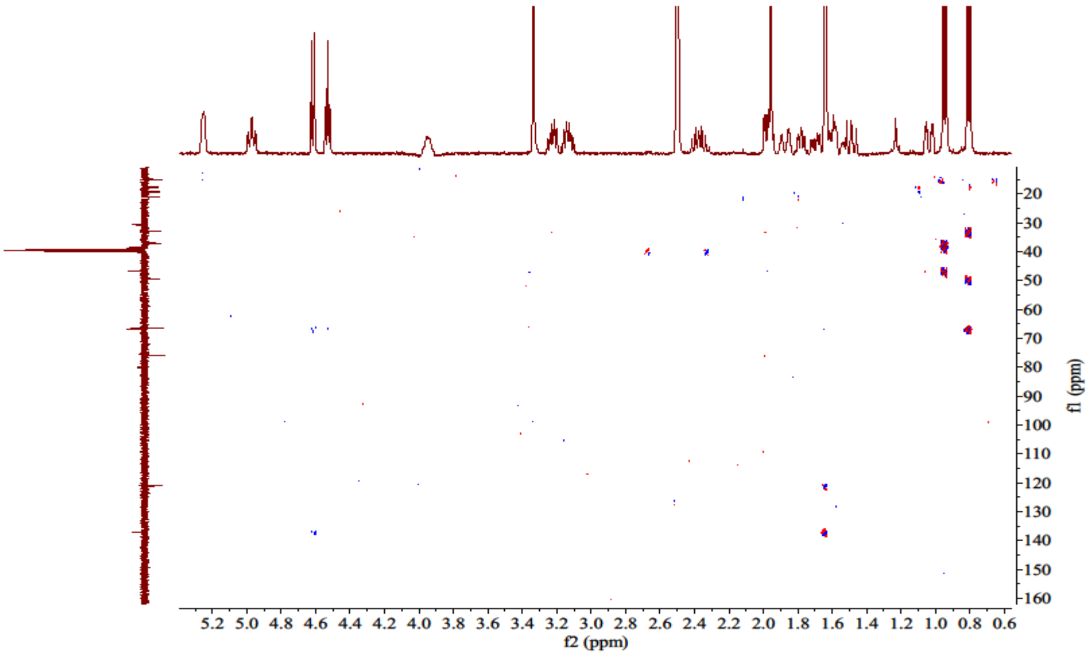

Fig. S33. NOESY spectrum of **4**

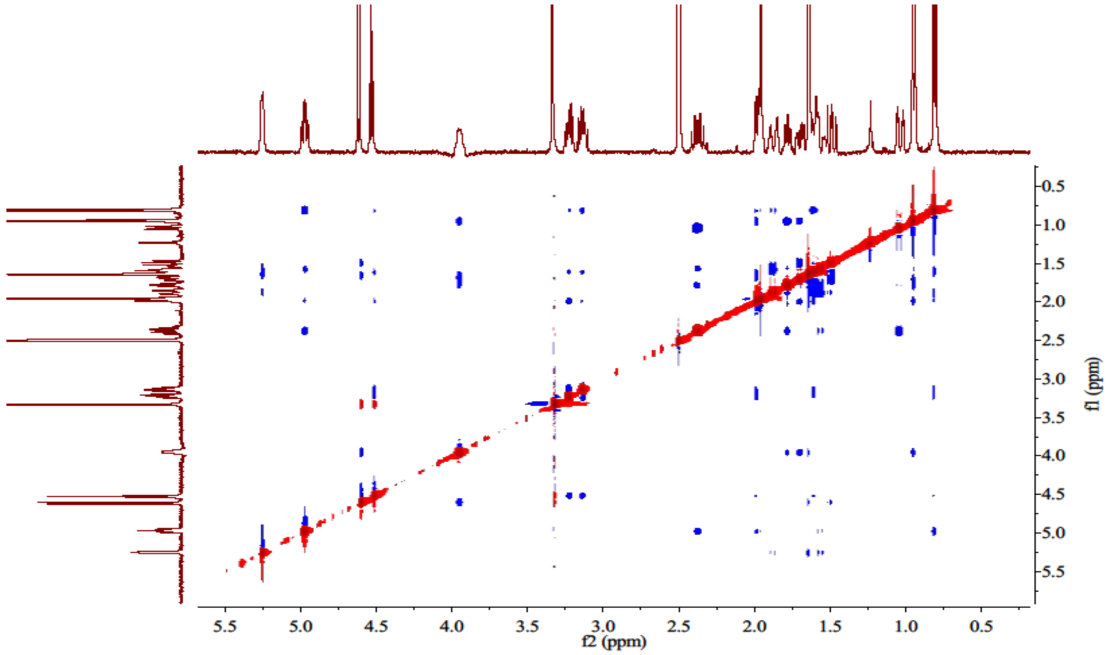

Fig. S34. HRESIMS spectrum of **4**

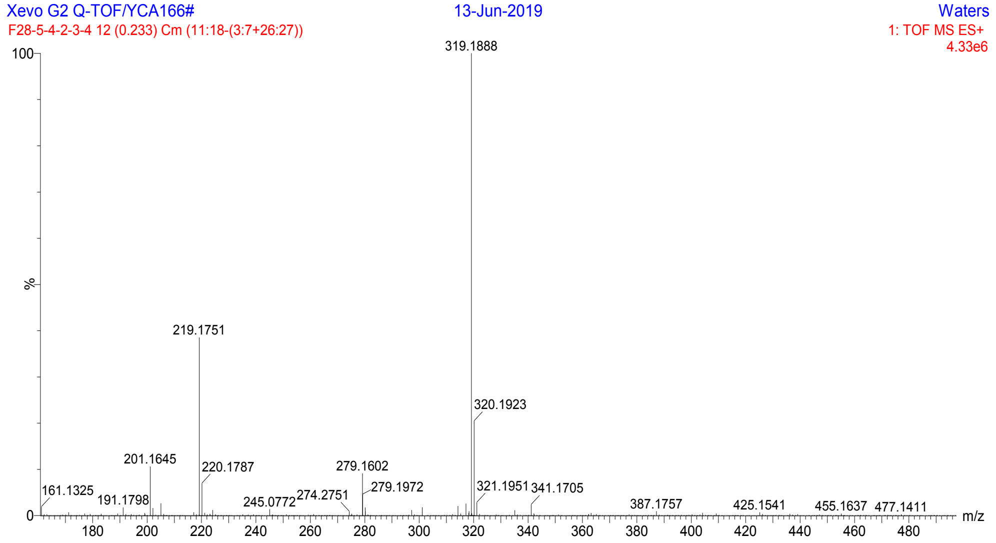

Mass	Calc. Mass	mDa	PPM	DBE	i-FIT	Norm	Conf(%)	Formula
319.1888	319.1885	0.3	0.9	3.5	685.6	0.120	88.70	C17 H28 O4 Na

Fig. S35. IR spectrum of **4**

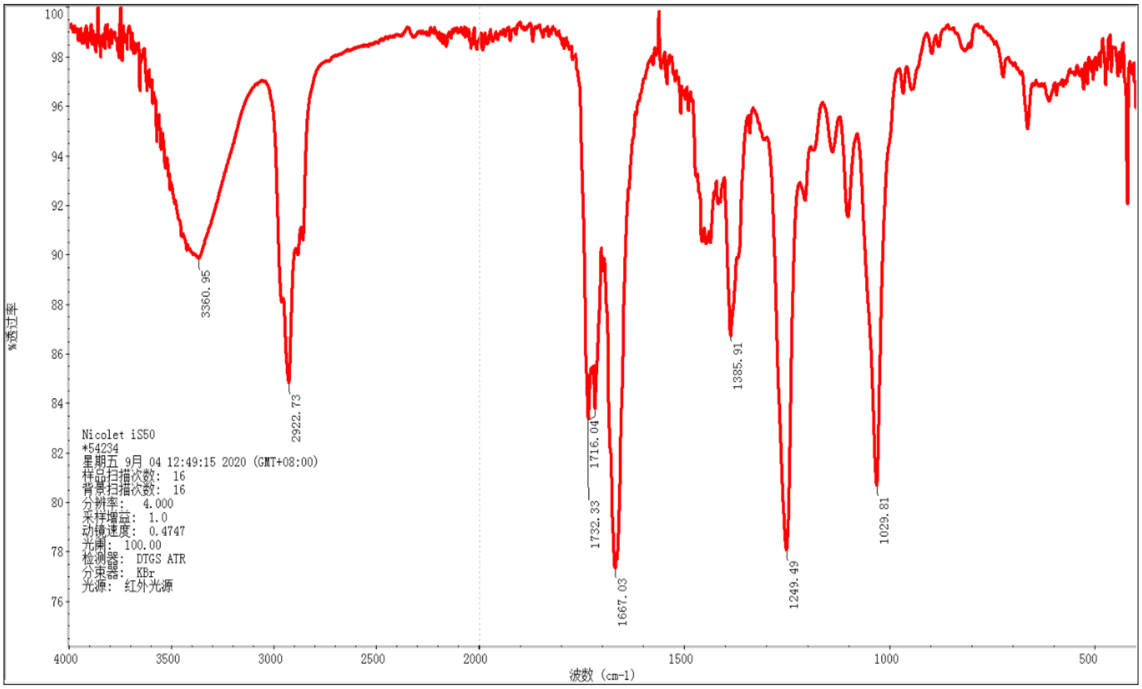

Fig. S36. UV spectrum of **4**

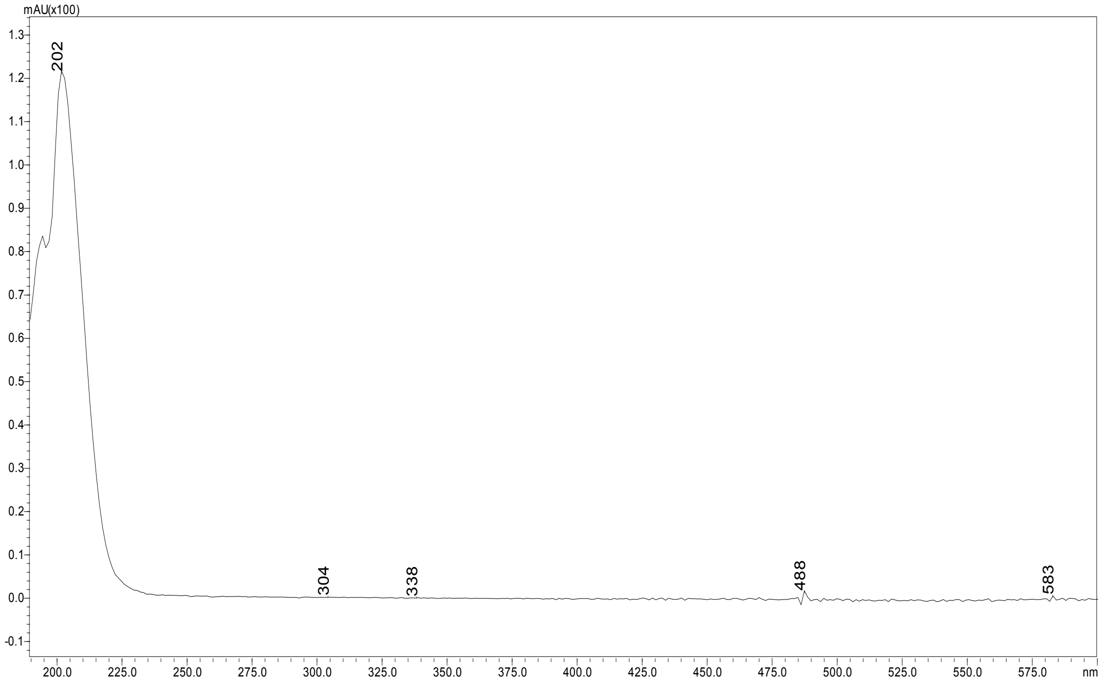

Fig. S37. ^{1}H NMR spectrum of **5** (400 MHz, DMSO-d6)

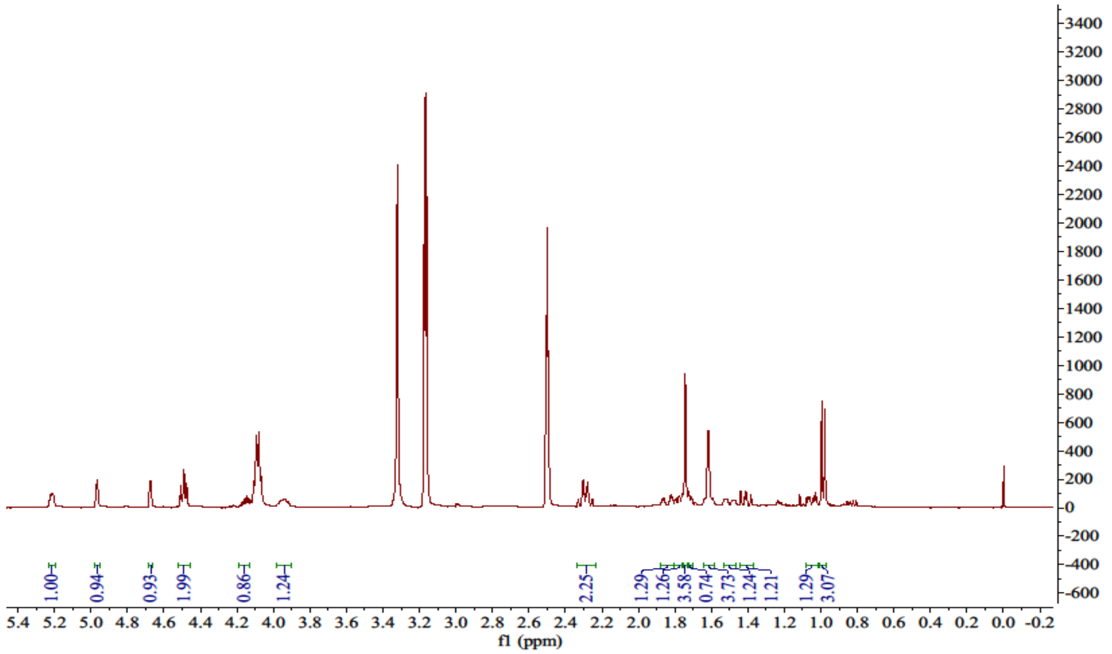

Fig. S38. ^{13}C NMR (APT) spectrum of **5** (100 MHz, DMSO-d6)

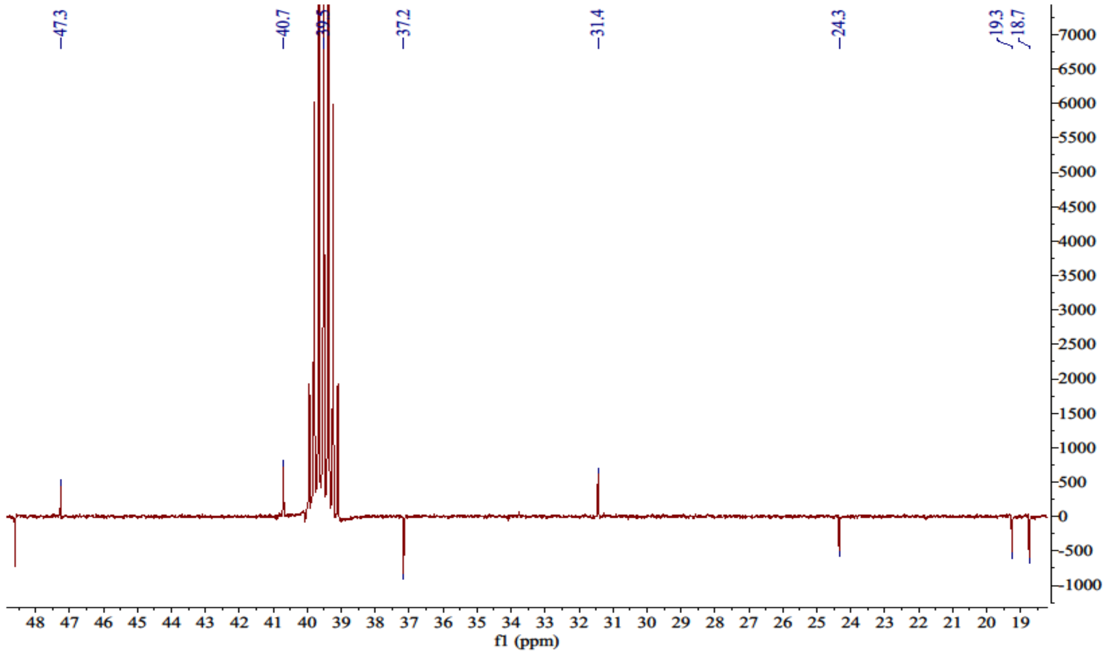

Fig. S39. ^{1}H-^{1}H COSY spectrum of **5**

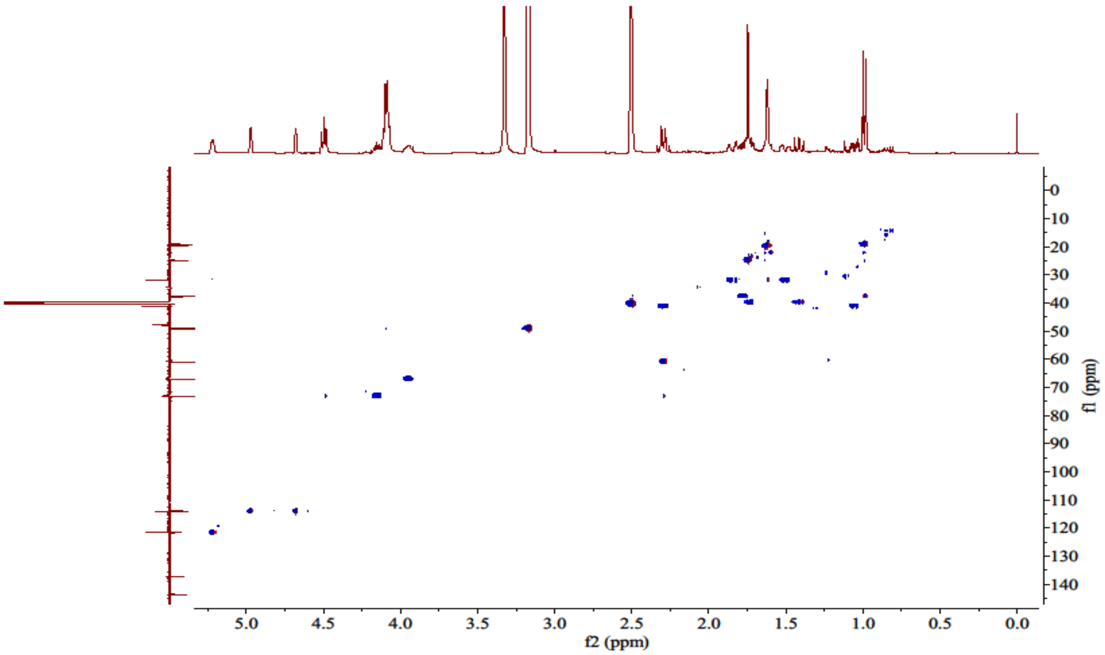

Fig. S40. HSQC spectrum of **5**

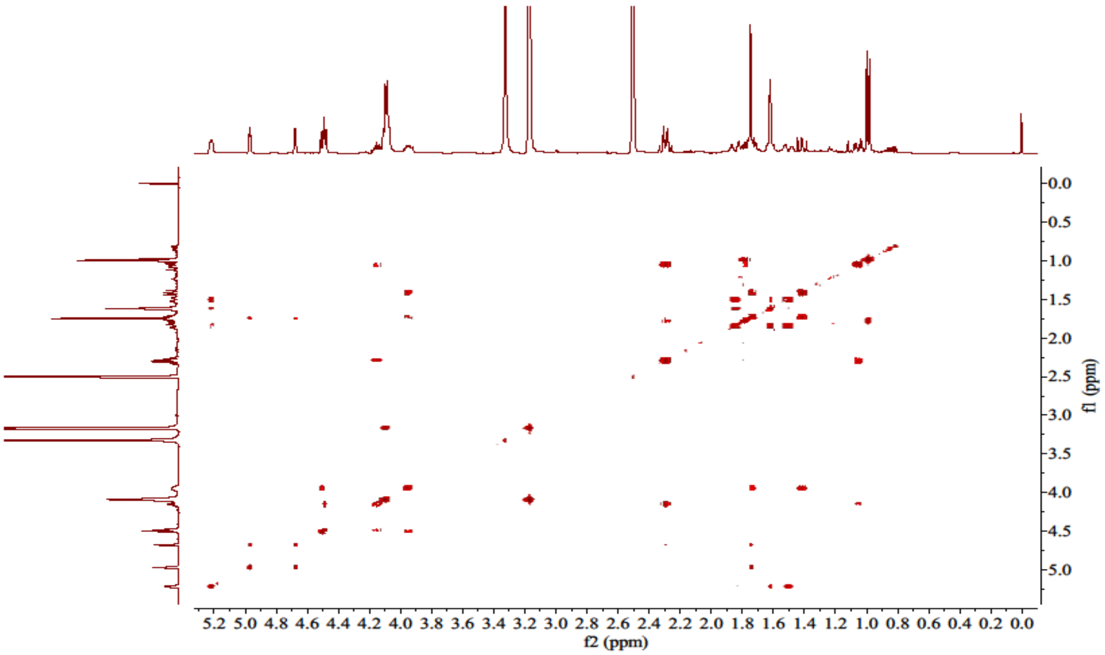

Fig. S41. HMBC spectrum of **5**

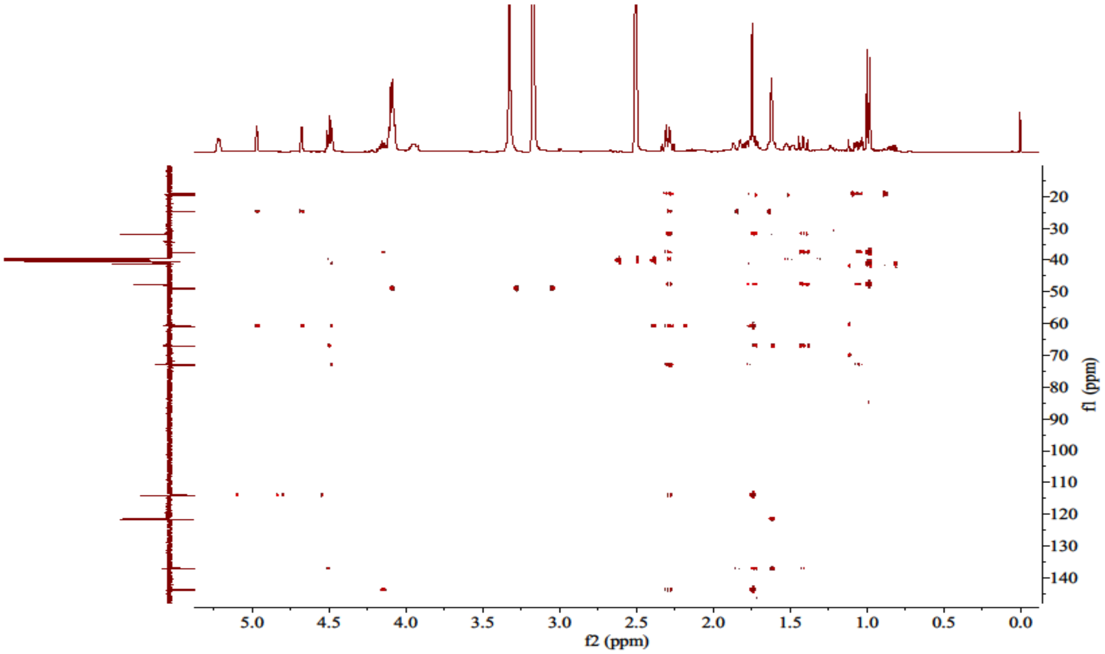

Fig. S42. NOESY spectrum of **5**

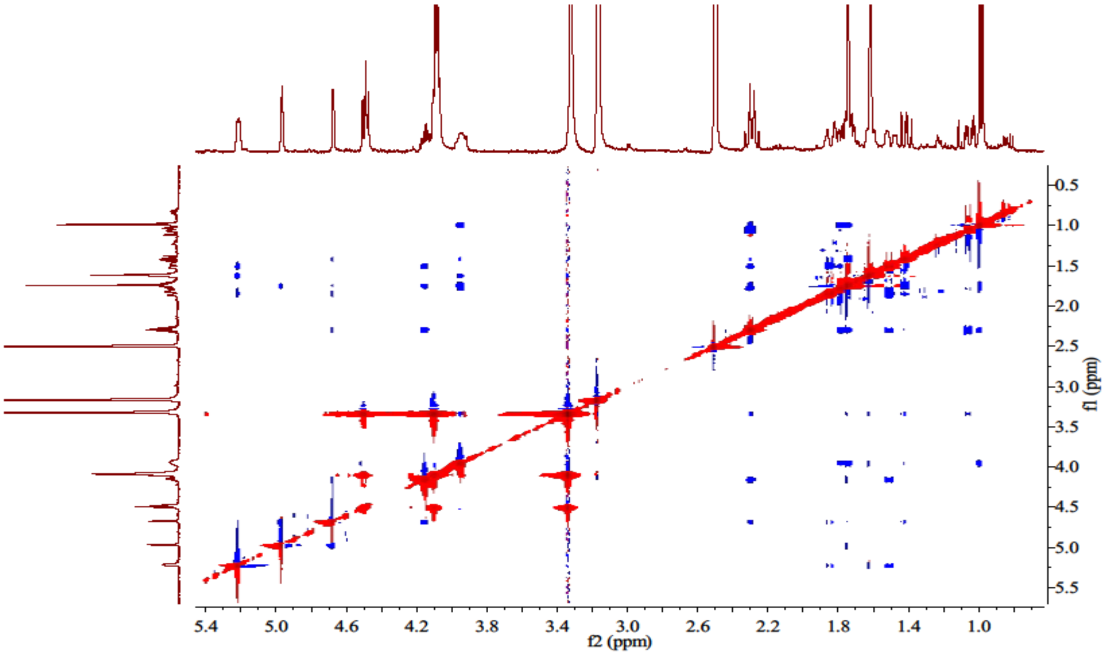

Fig. S43. HRESIMS spectrum of **5**

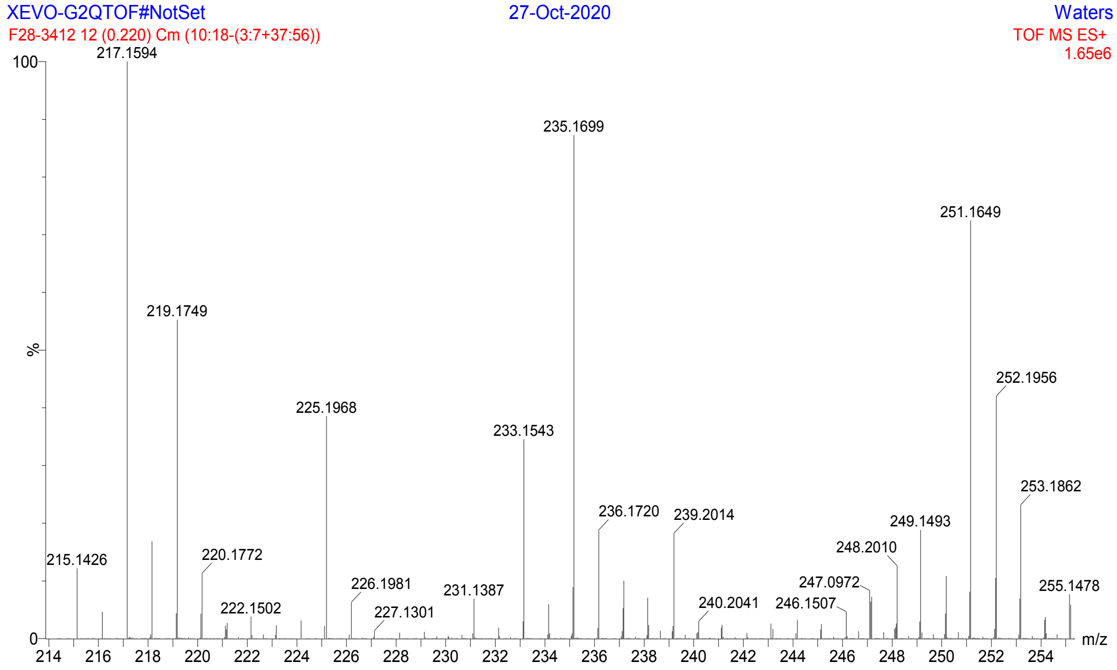

Fig. S44. IR spectrum of **5**

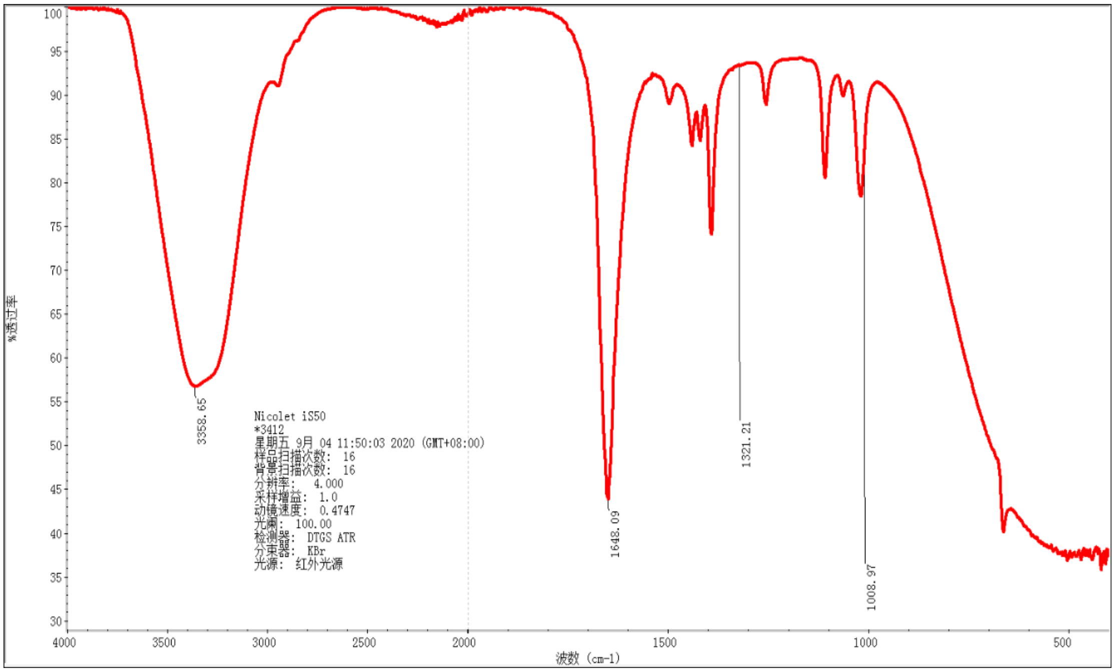

Fig. S45. UV spectrum of **5**

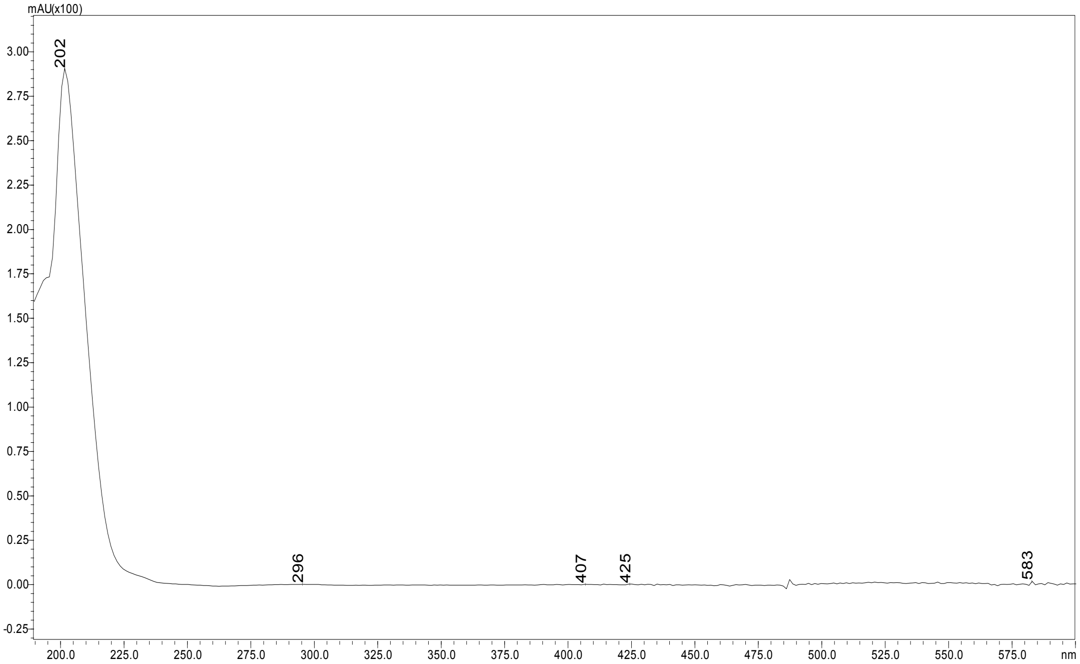

Fig. S46. ^{1}H NMR spectrum of **6** (400 MHz, DMSO-d6)

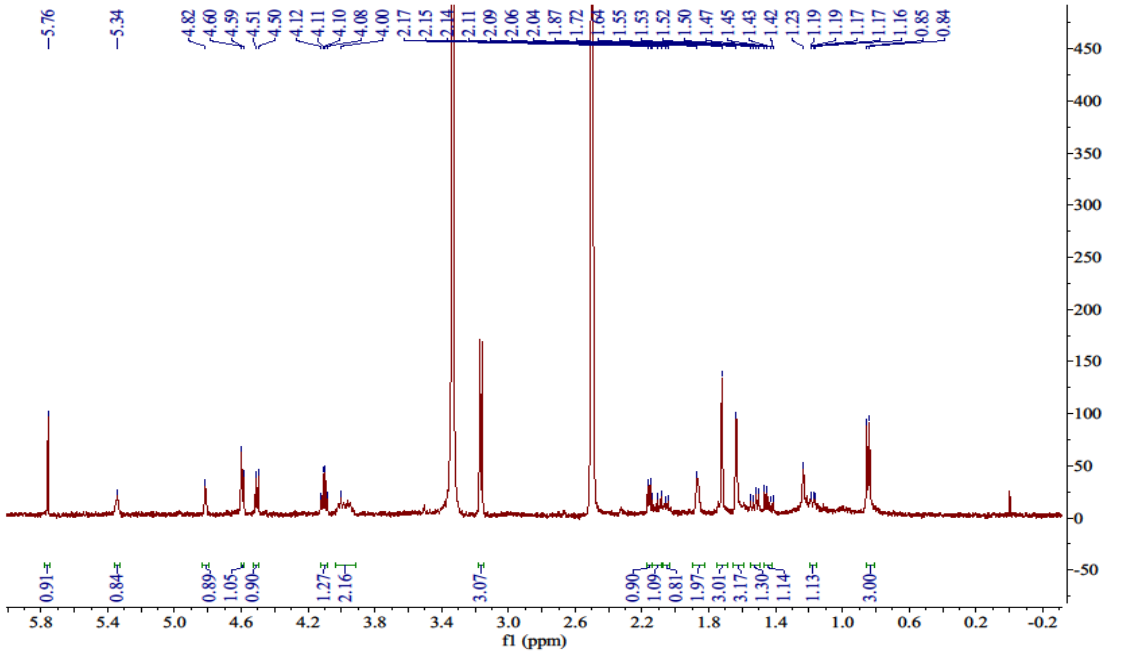

Fig. S47. ^{13}C NMR (APT) spectrum of **6** (100 MHz, DMSO-d6)

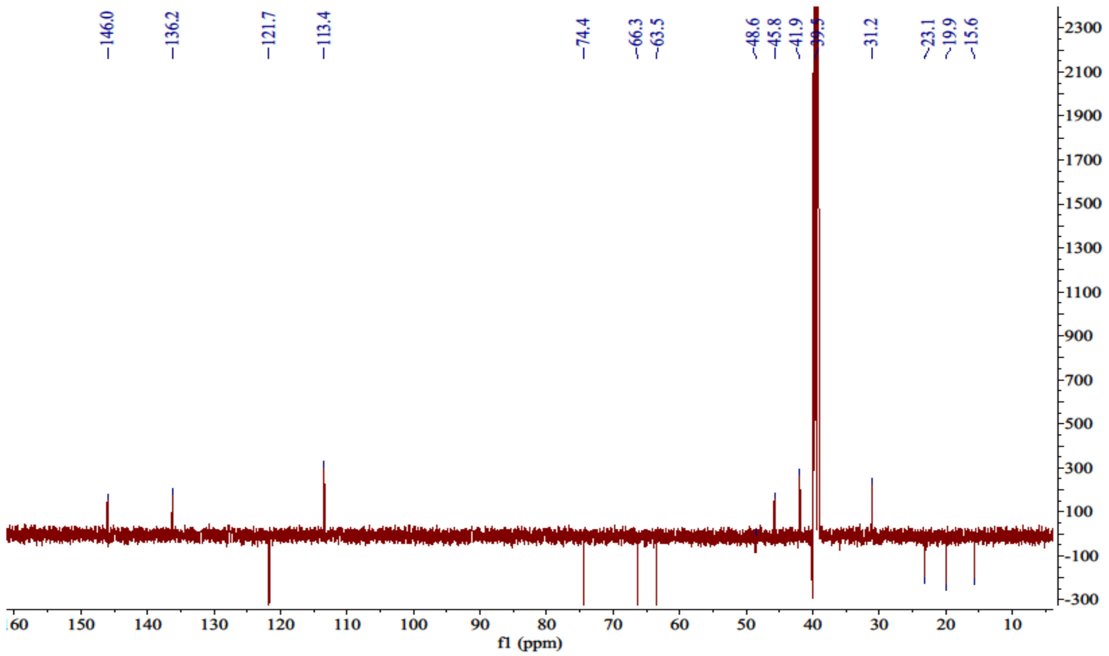

Fig. S48. ^{1}H-^{1}H COSY spectrum of **6**

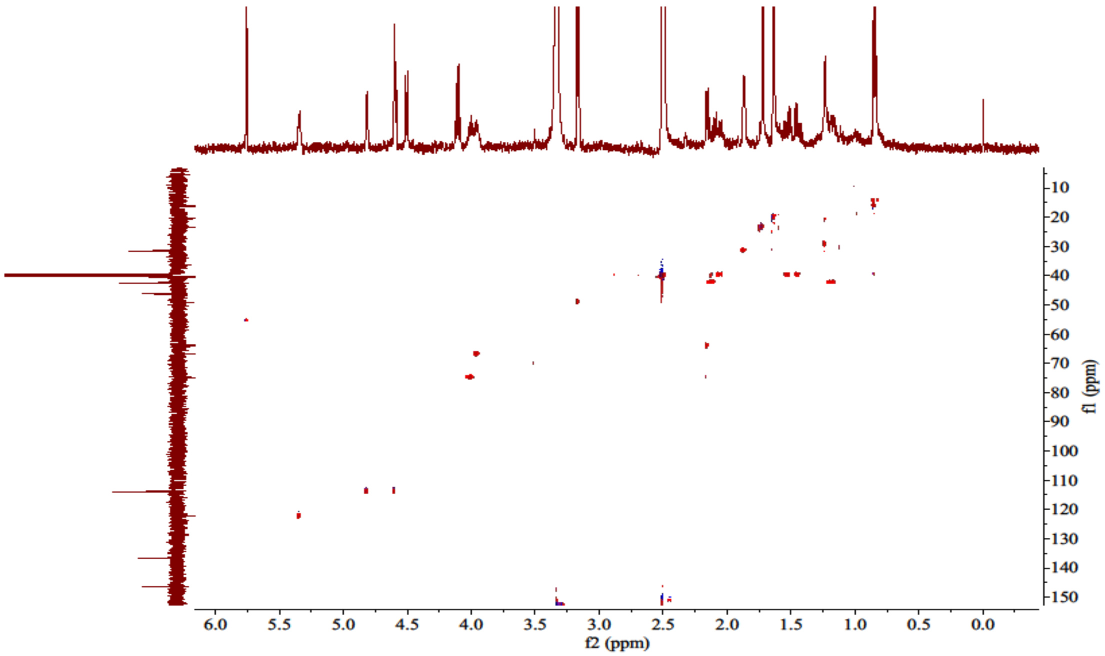

Fig. S49. HSQC spectrum of **6**

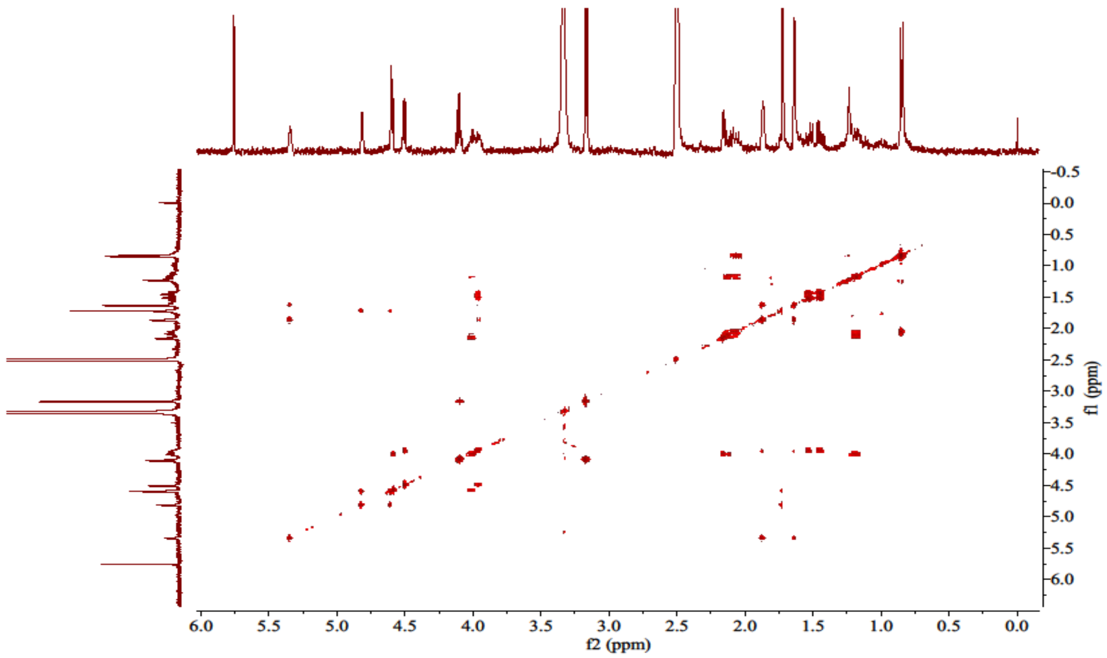

Fig. S50. HMBC spectrum of **6**

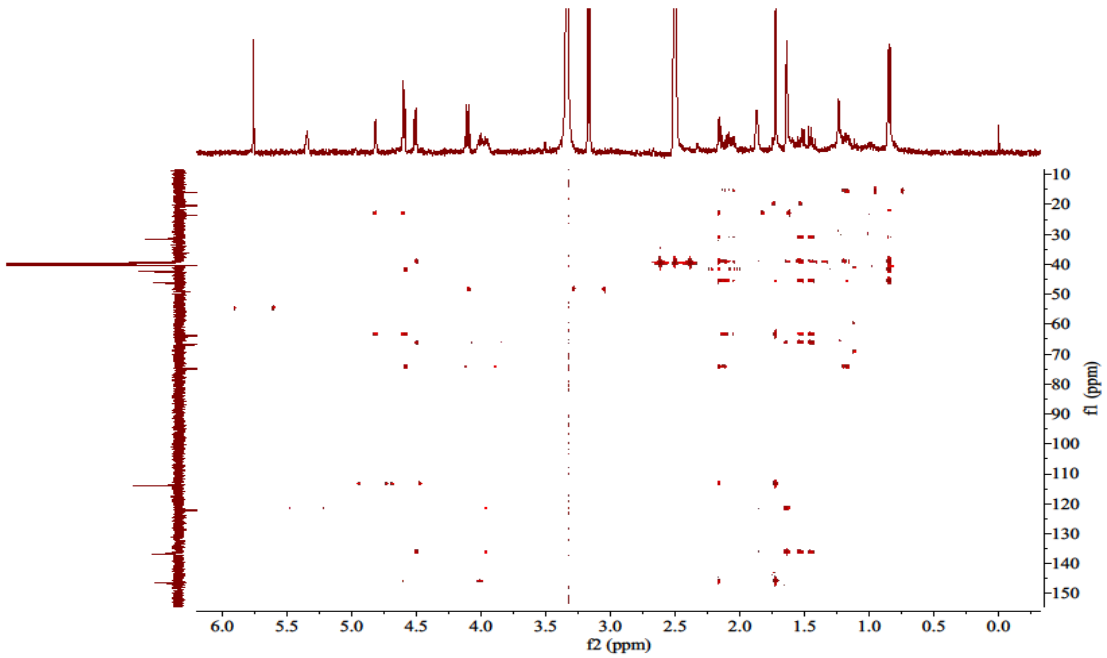

Fig. S51. NOESY spectrum of **6**

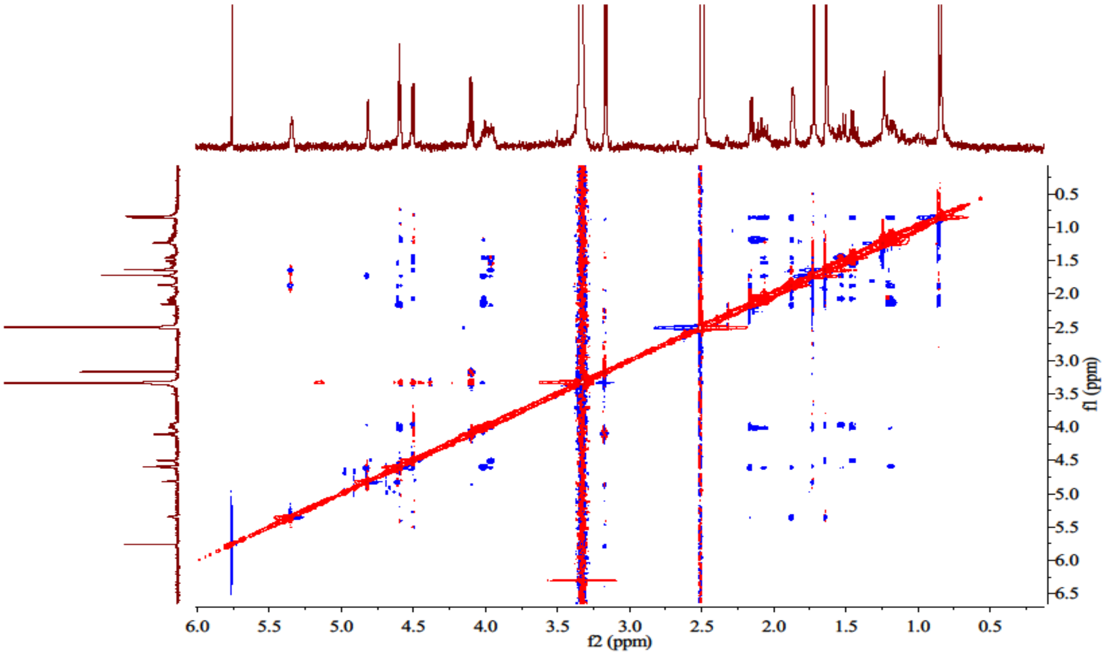

Fig. S52. HRESIMS spectrum of **6**

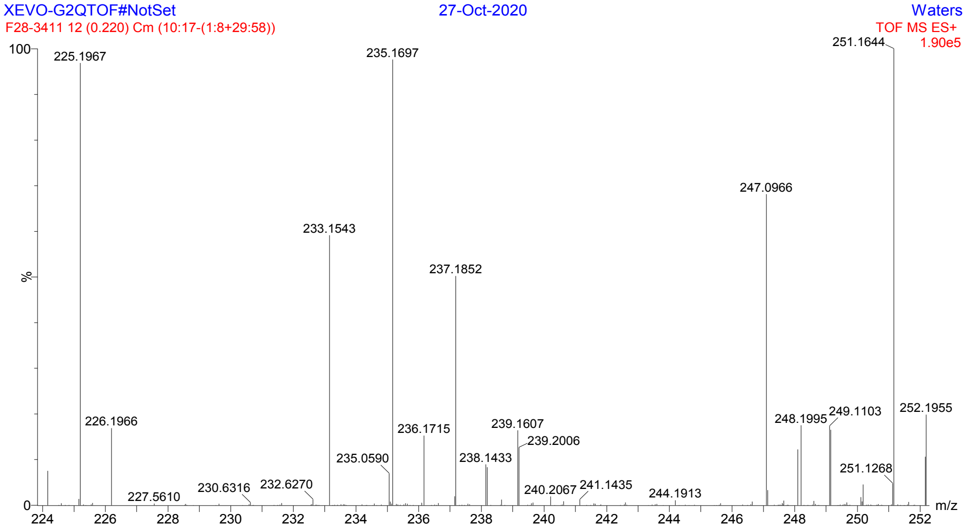

Mass	Calc. Mass	m Da	PPM	DBE	i-FIT	Norm	Conf (%)	Formula
237.1852	237.1855	-0.3	-1.3	3.5	219.9	n/a	n/a	C15H25 O2

Fig. S53. IR spectrum of **6**

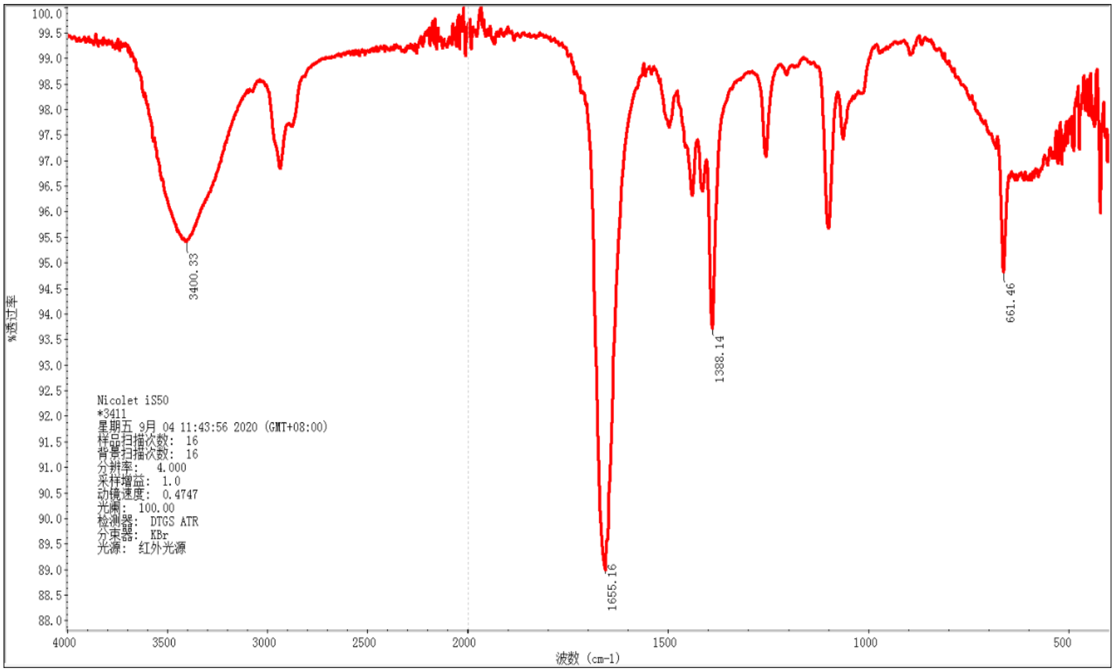

Fig. S54. UV spectrum of **6**

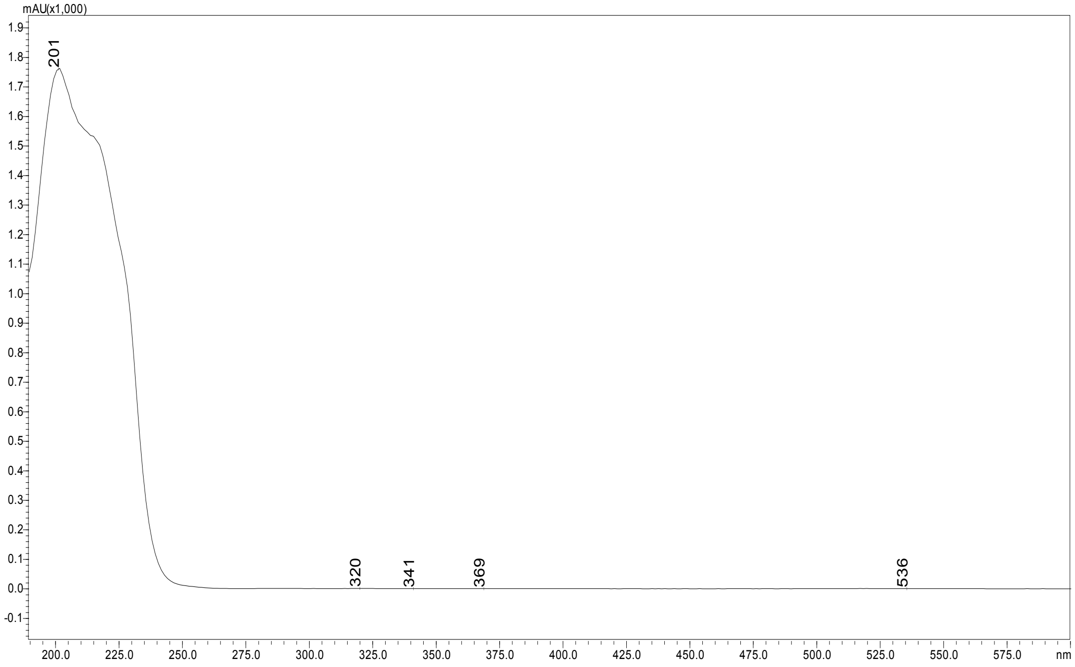

Fig. S55. ^{1}H NMR spectrum of **7** (400 MHz, DMSO-d6)

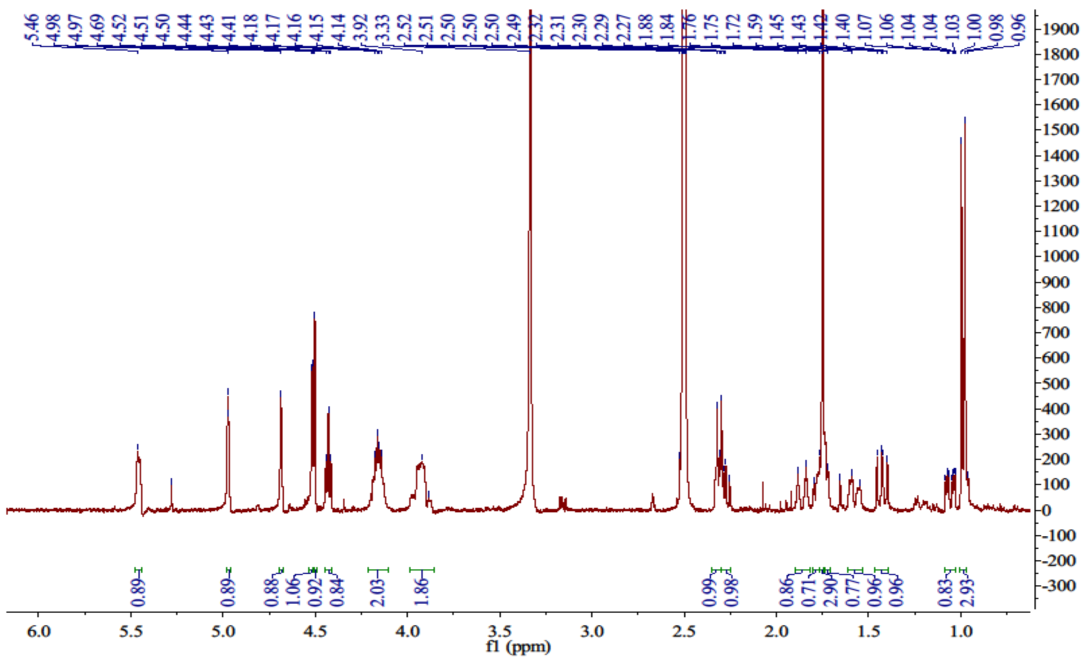

Fig. S56. ^{13}C NMR (APT) spectrum of **7** (100 MHz, DMSO-d6)

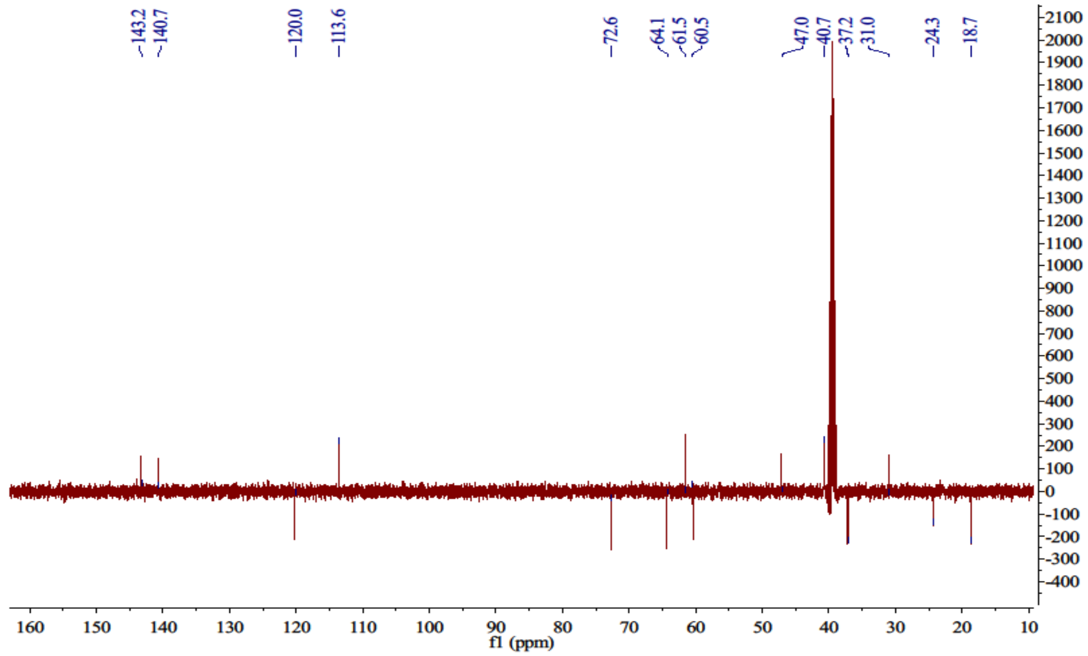

Fig. S57. ^{1}H-^{1}H COSY spectrum of **7**

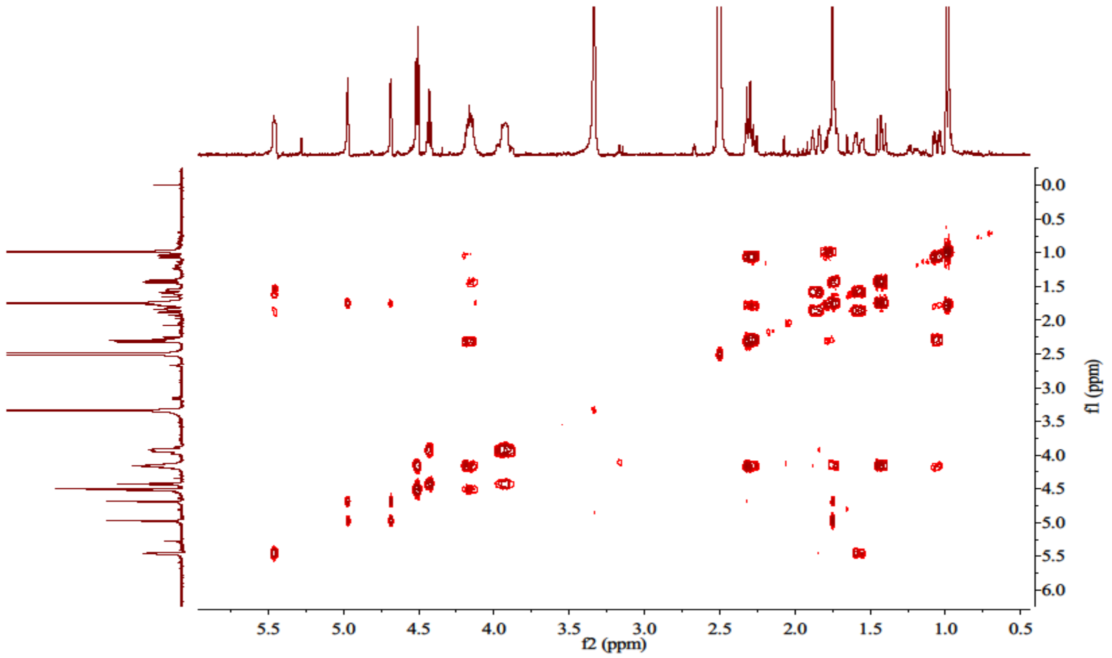

Fig. S58. HSQC spectrum of **7**

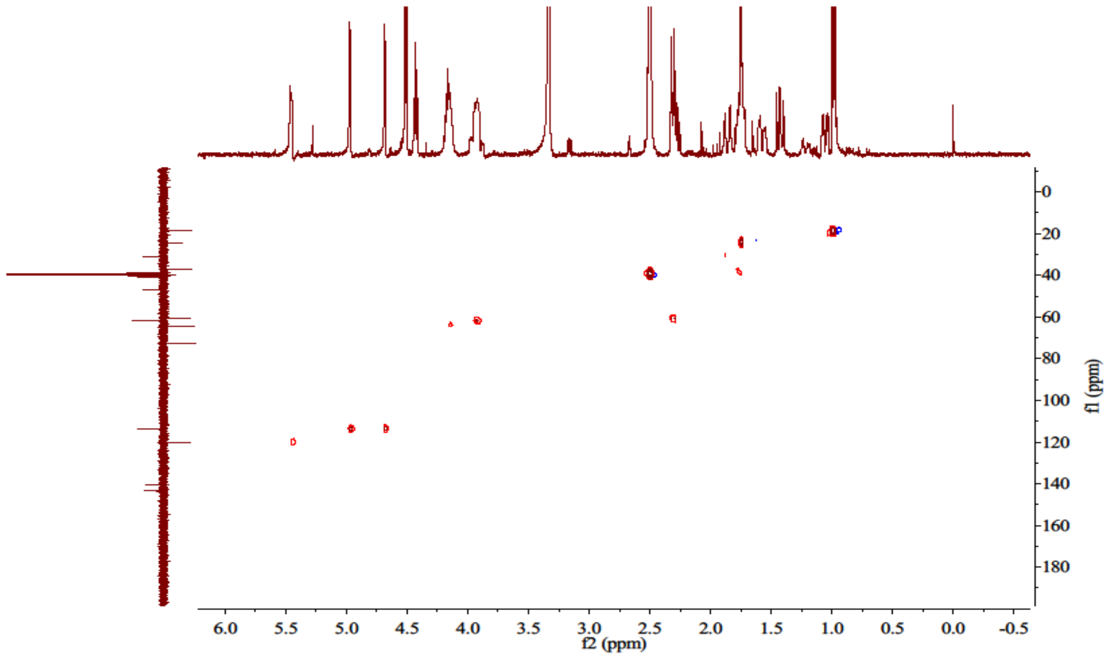

Fig. S59. HMBC spectrum of **7**

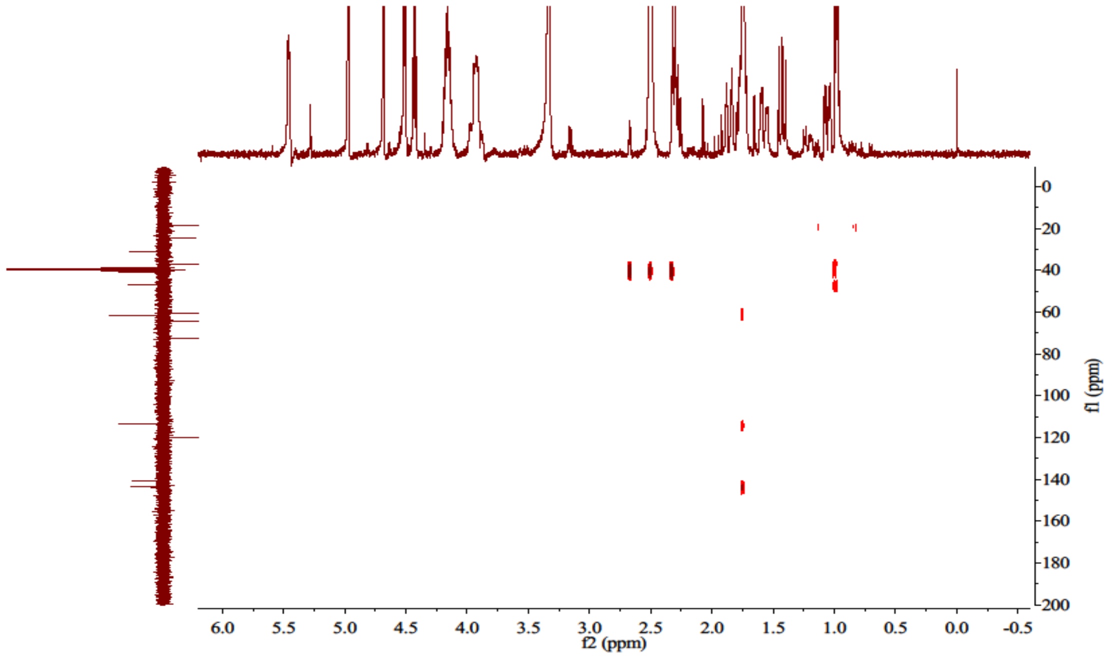

Fig. S60. NOESY spectrum of **7**

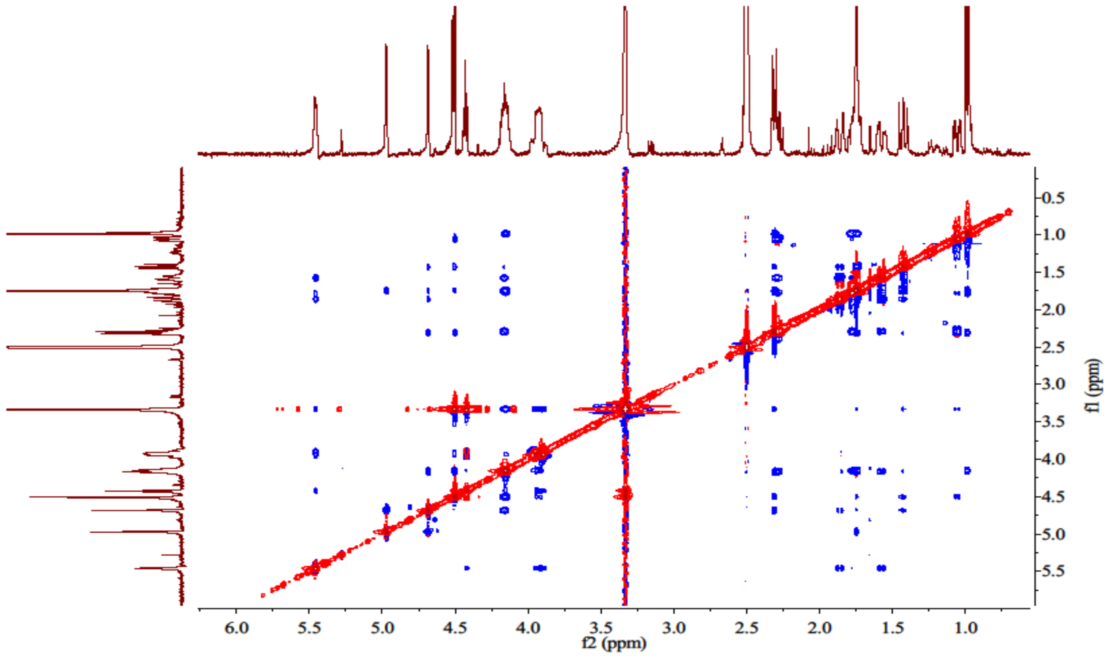

Fig. S61. HRESIMS spectrum of **7**

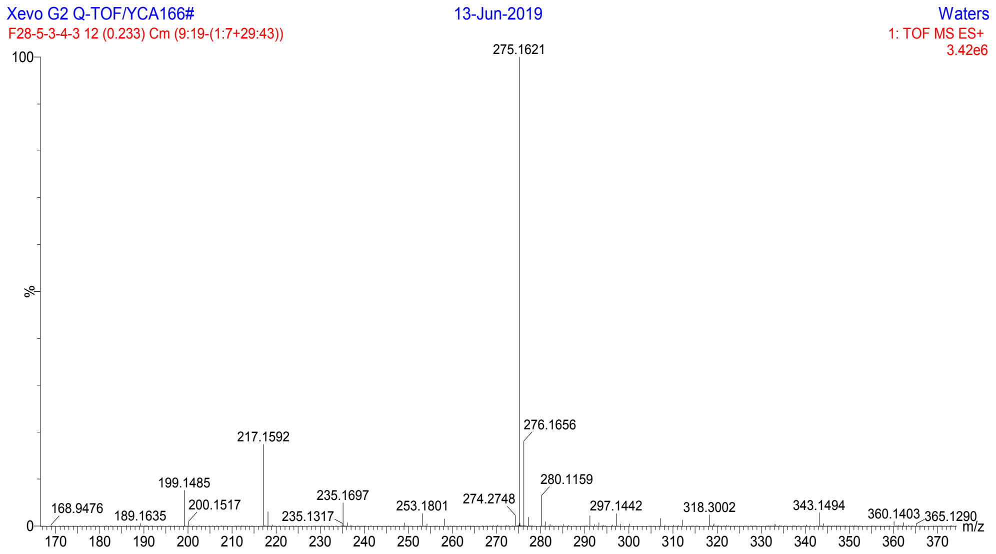

Mass	Calc. Mass	mDa	PPM	DBE	i-FIT	Norm	Conf(%)	Formula
275.1621	275.1623	-0.2	-0.7	3.5	480.5	n/a	n/a	$C_{15}H_{24}O_3Na$

Fig. S62. IR spectrum of **7**

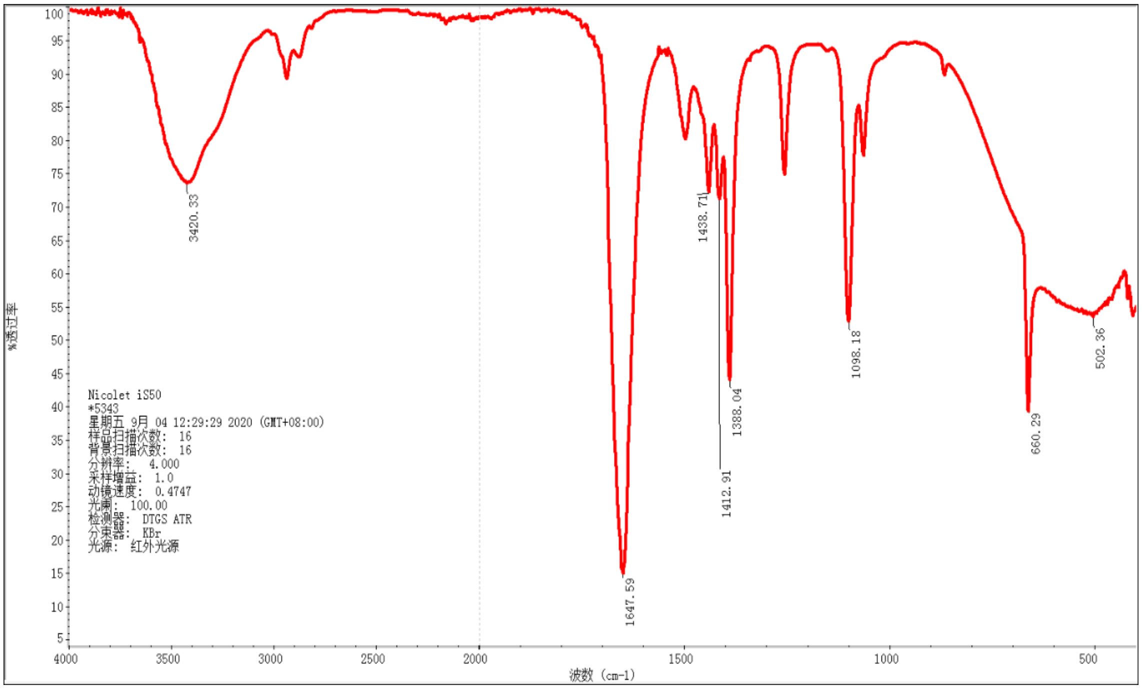

Fig. S63. UV spectrum of **7**

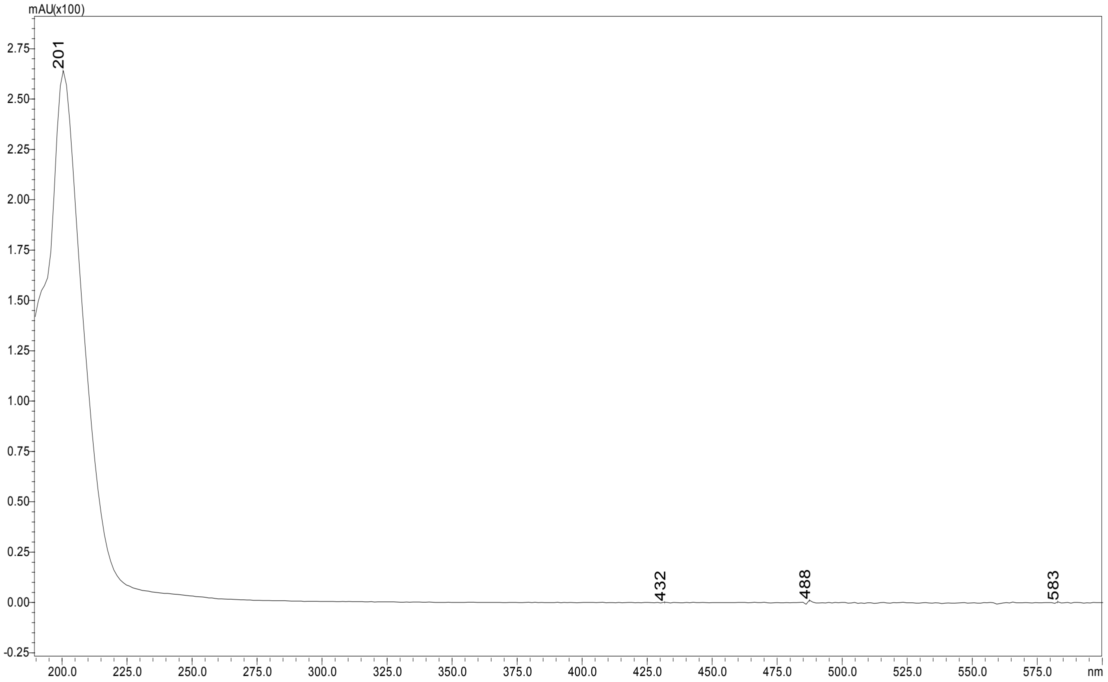

Fig. S64. ^{1}H NMR spectrum of **8** (400 MHz, DMSO-d6)

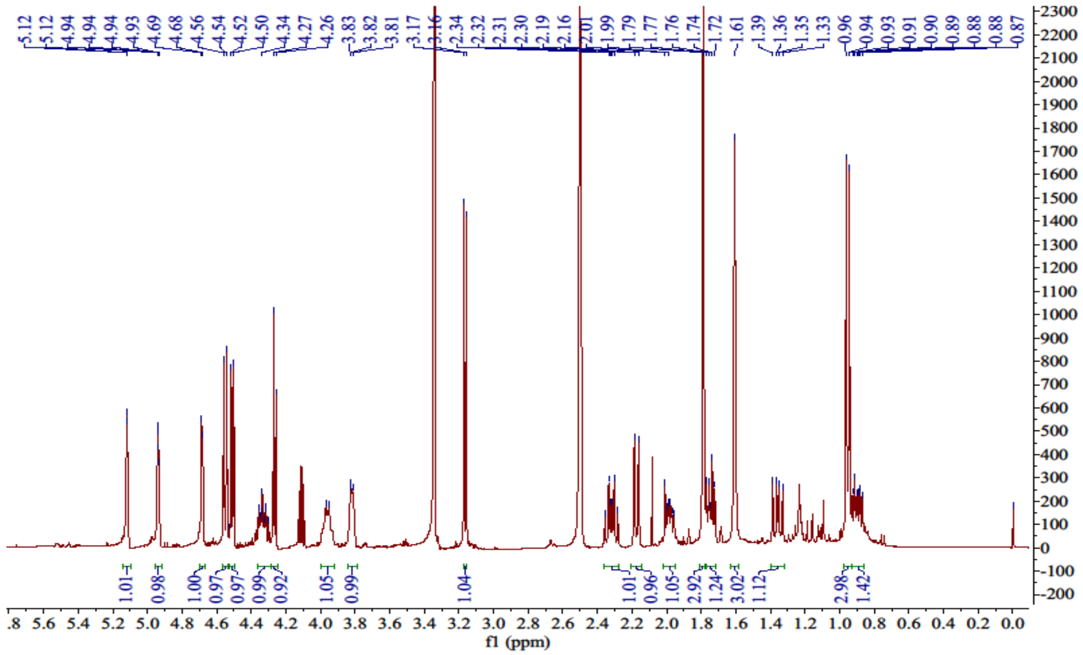

Fig. S65. ^{13}C NMR (APT) spectrum of **8** (100 MHz, DMSO-d6)

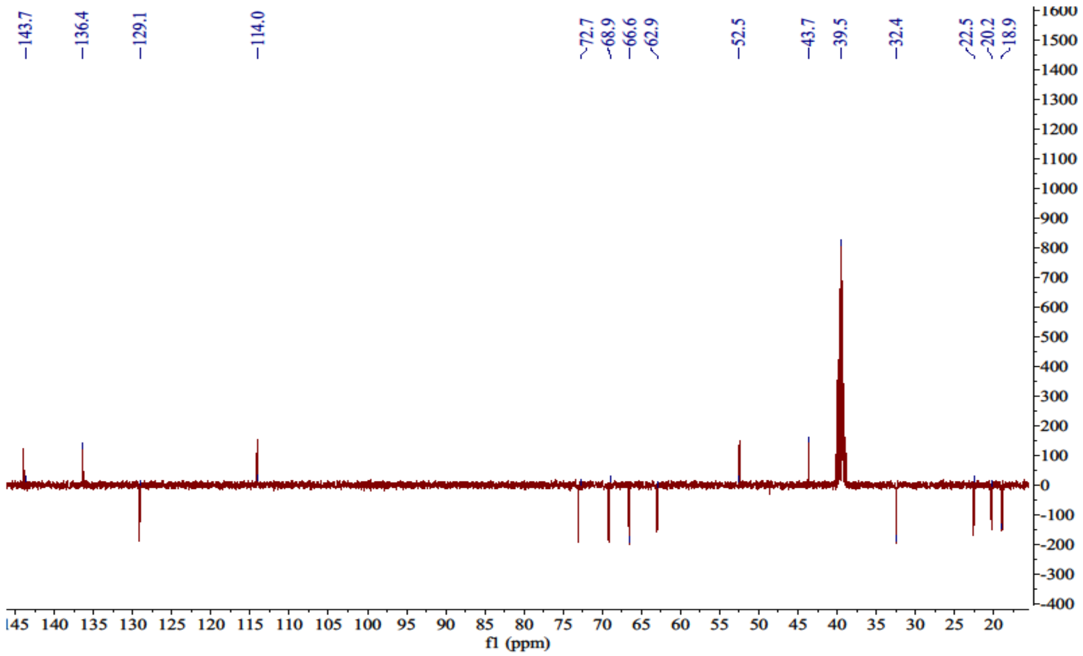

Fig. S66. ^{1}H-^{1}H COSY spectrum of **8**

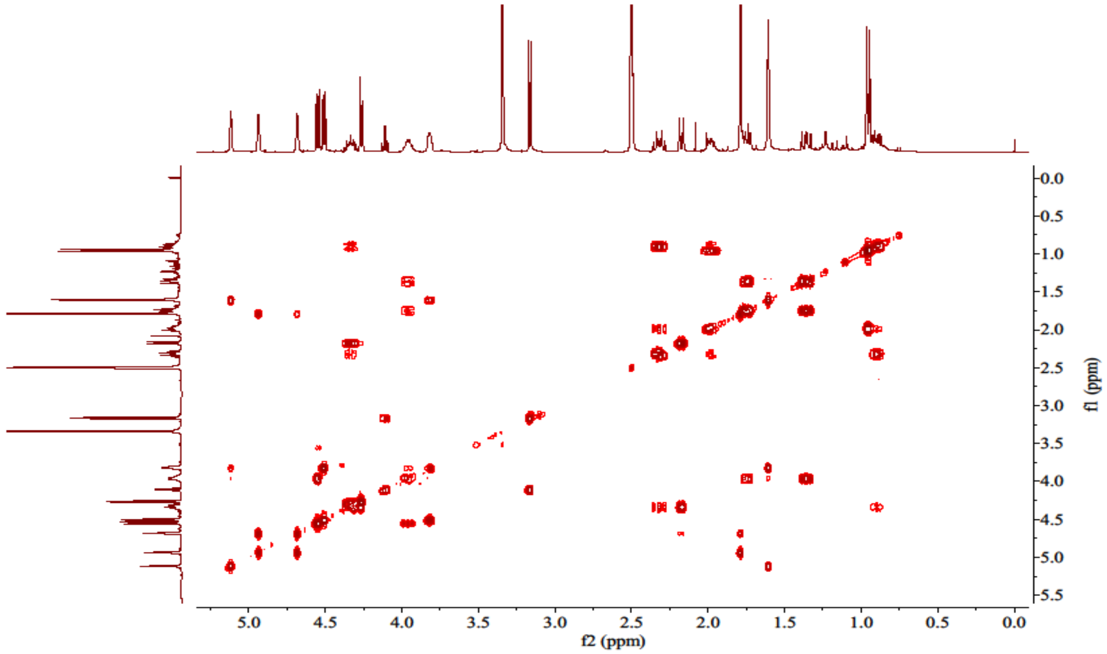

Fig. S67. HSQC spectrum of **8**

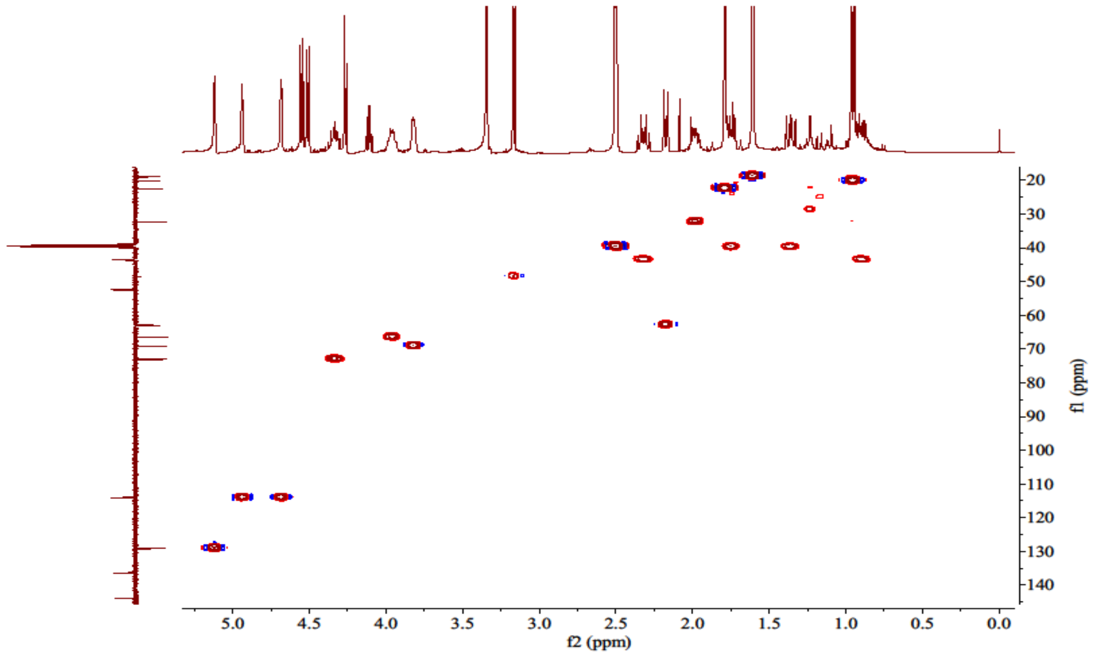

Fig. S68. HMBC spectrum of **8**

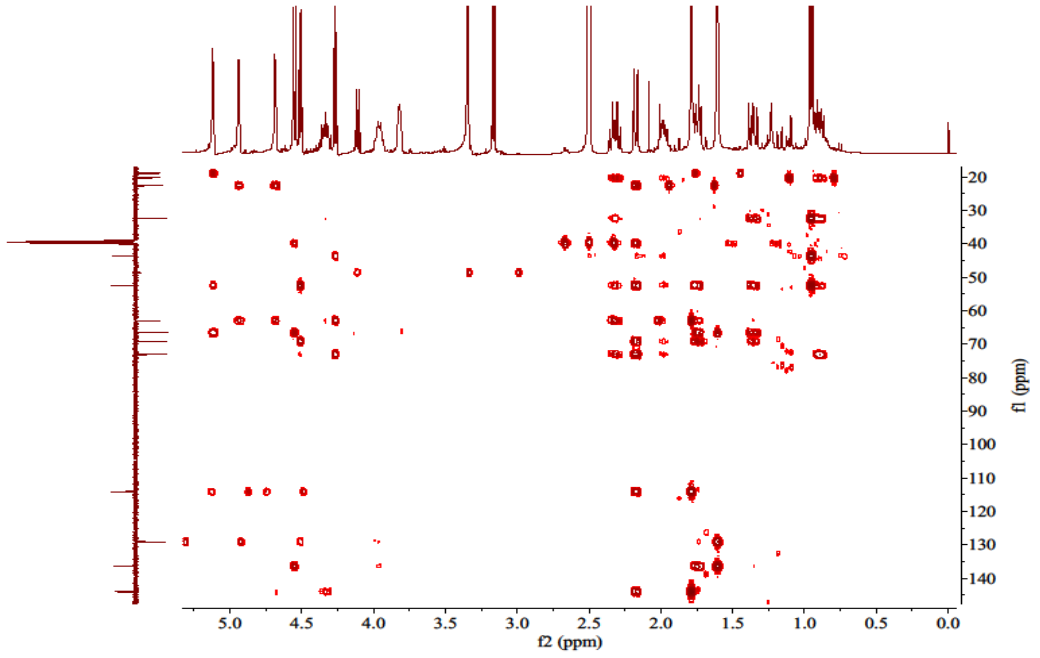

Fig. S69. NOESY spectrum of **8**

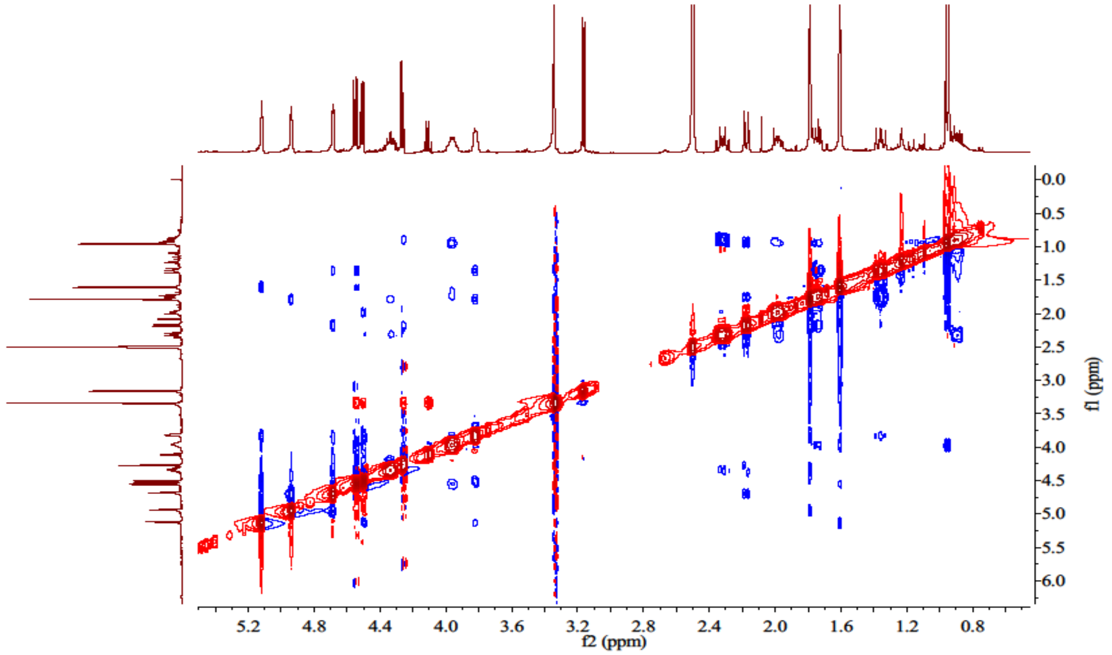

Fig. S70. HRESIMS spectrum of **8**

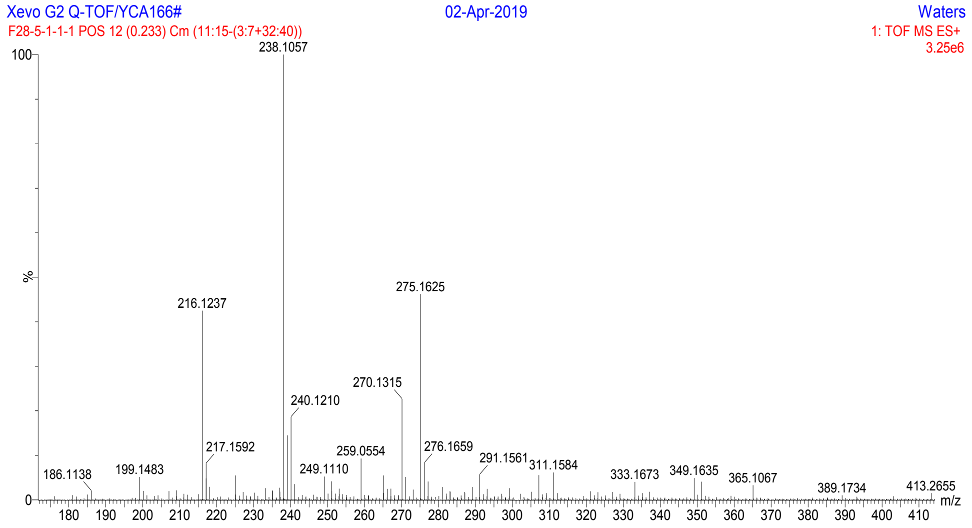

Mass	Calc. Mass	mDa	PPM	DBE	i-FIT	Norm	Conf(%)	Formula
275.1625	275.1623	0.2	0.7	3.5	531.7	n/a	n/a	C15 H24 O3 Na

Fig. S71. IR spectrum of **8**

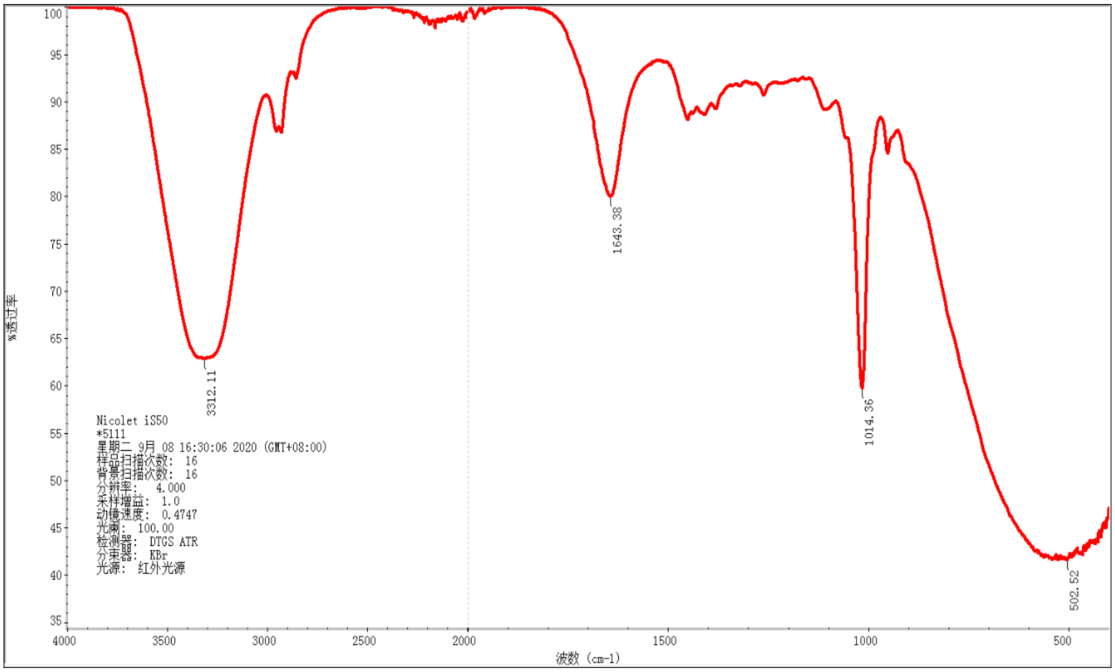

Fig. S72. UV spectrum of **8**

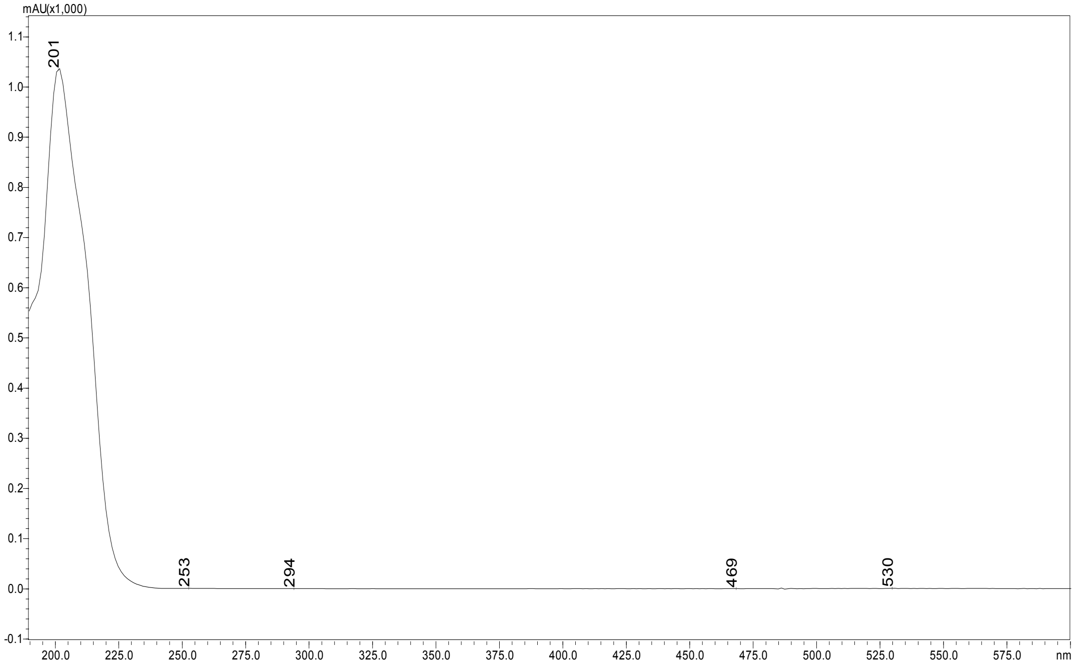

Fig. S73. ^{1}H NMR spectrum of **9** (400 MHz, DMSO-d6)

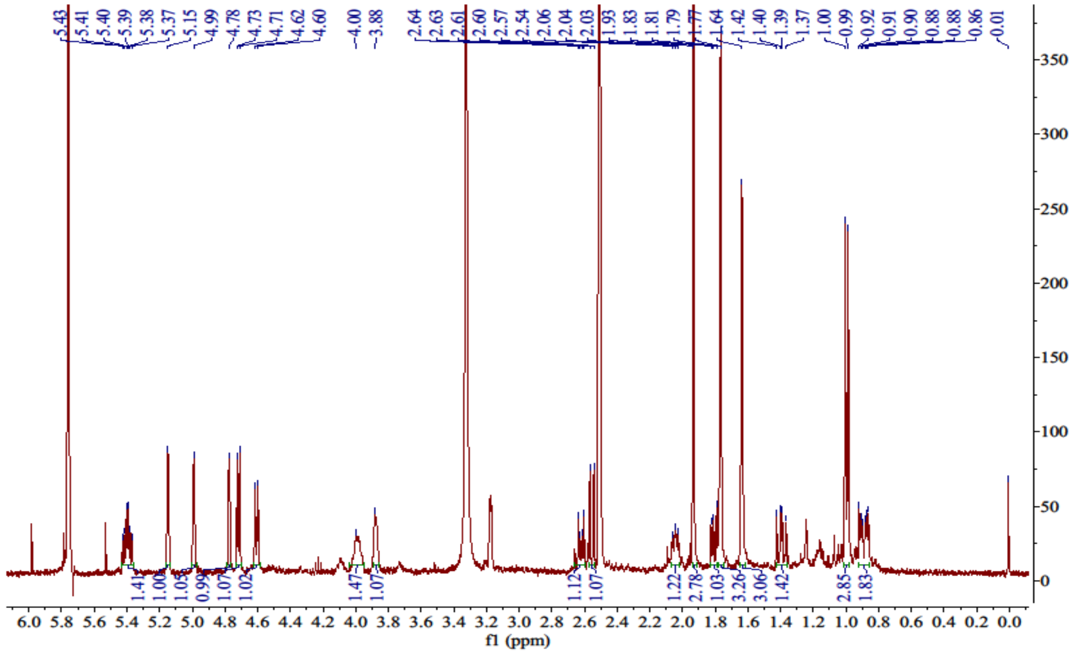

Fig. S74. ^{13}C NMR (APT) spectrum of **9** (100 MHz, DMSO-d6)

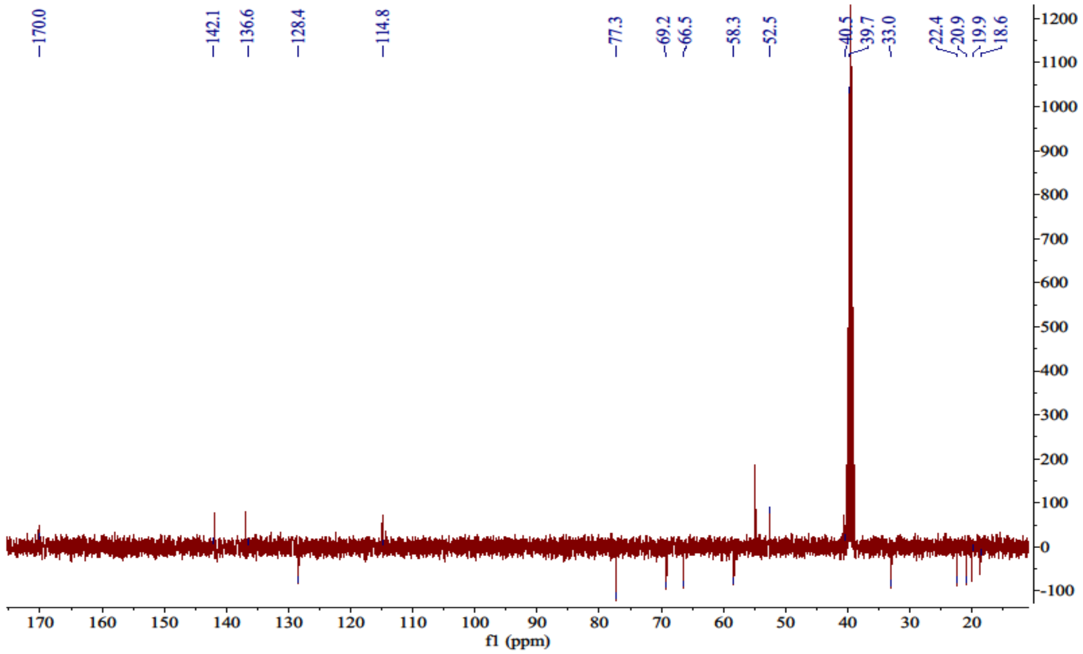

Fig. S75. ^1H-^1H COSY spectrum of **9**

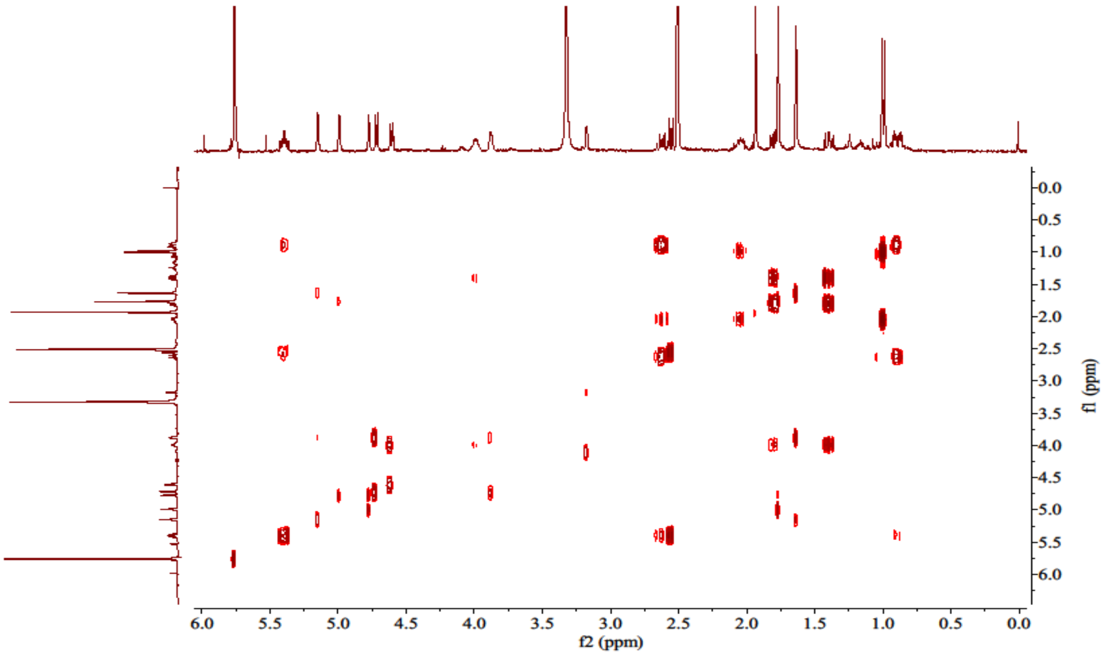

Fig. S76. HSQC spectrum of **9**

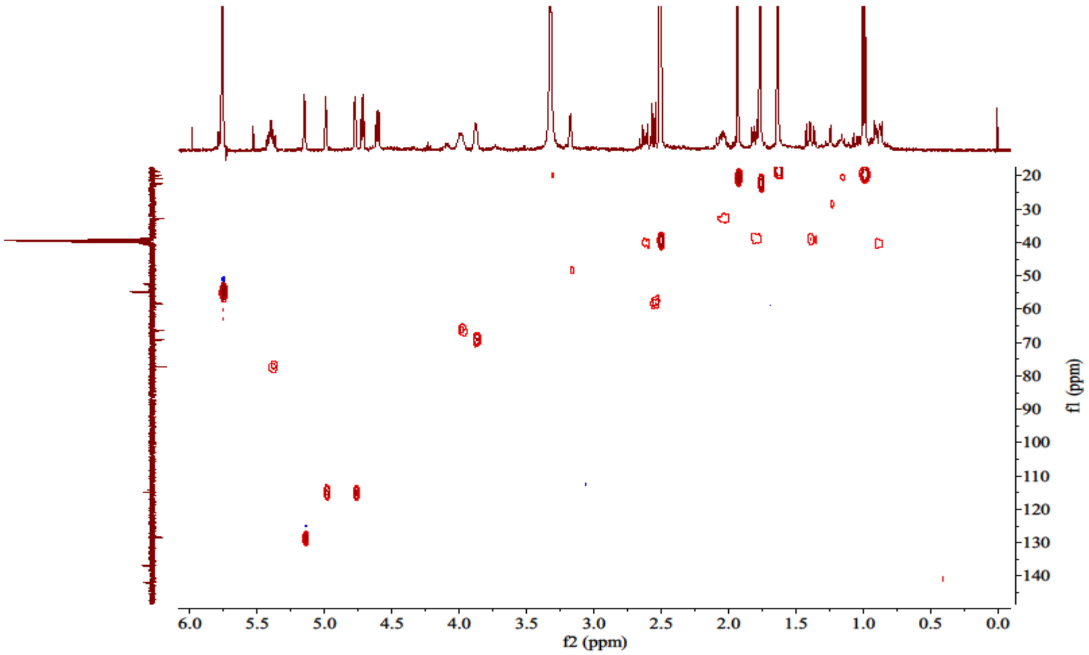

Fig. S77. HMBC spectrum of **9**

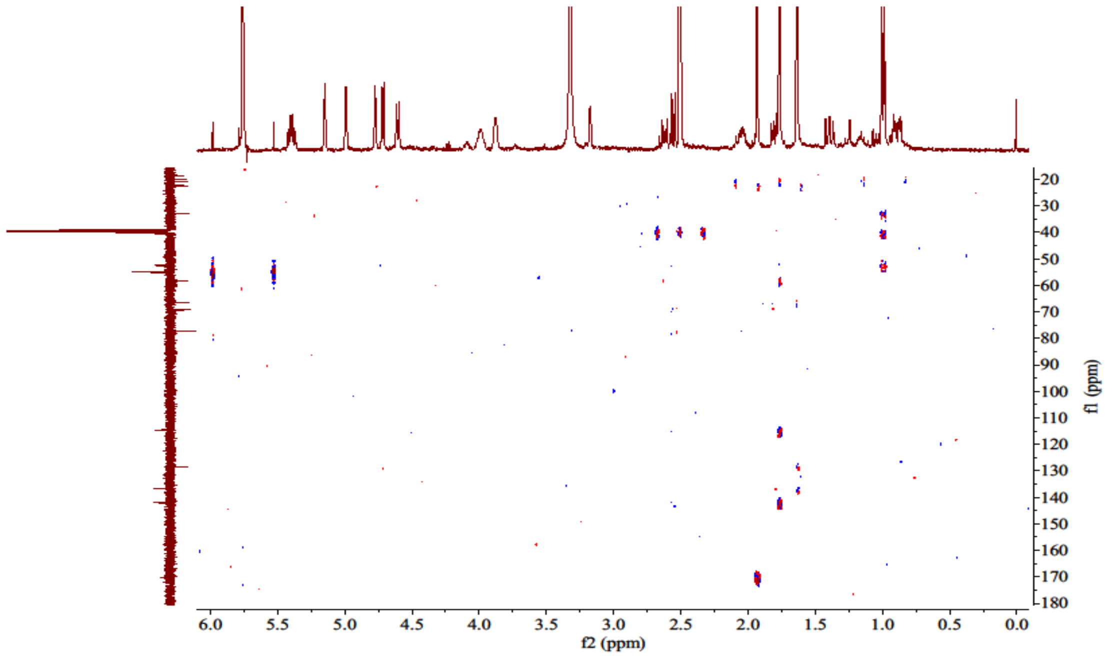

Fig. S78. NOESY spectrum of **9**

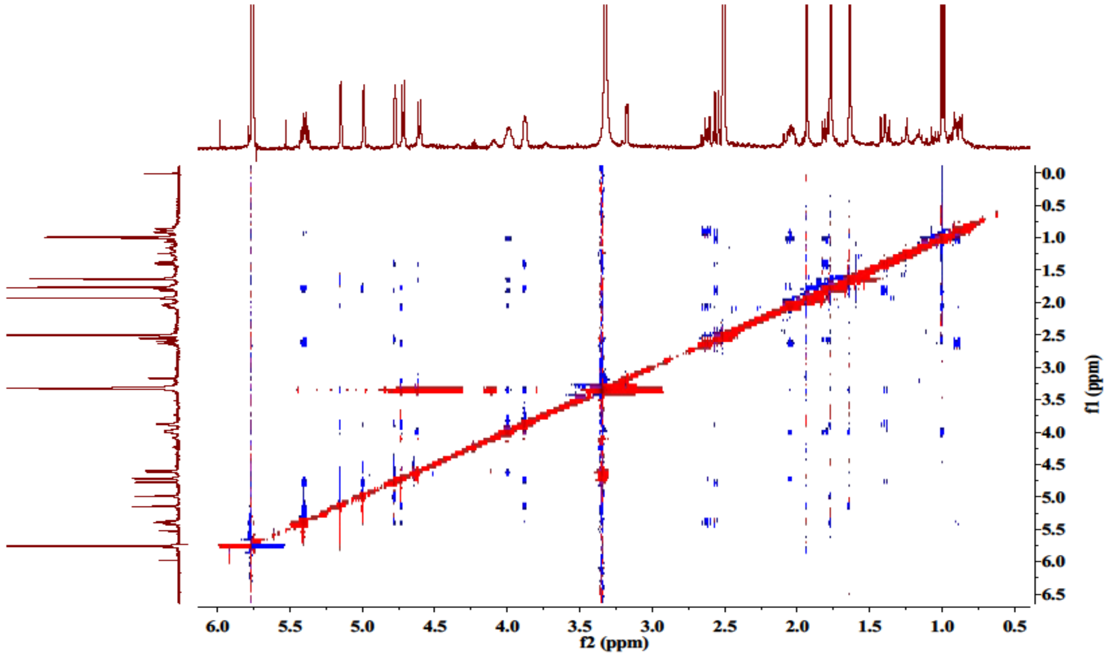

Fig. S79. HRESIMS spectrum of **9**

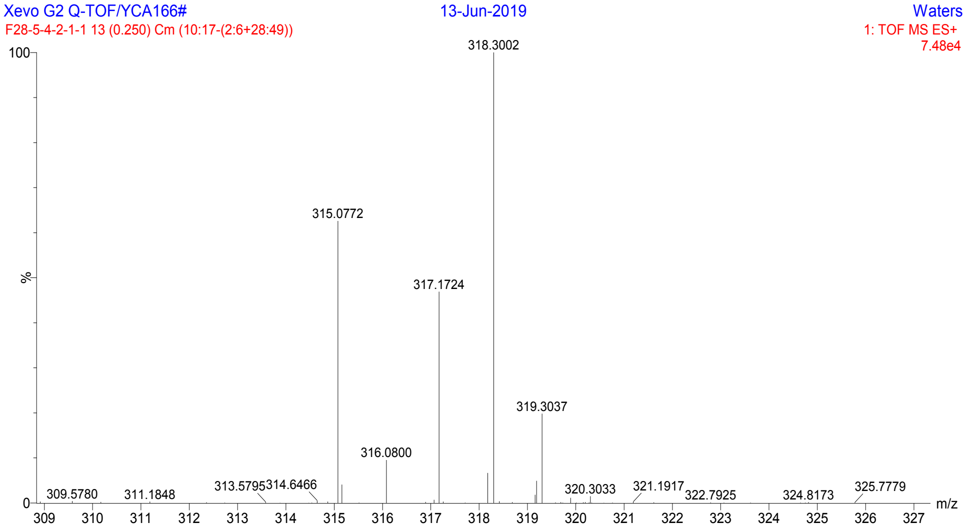

Mass	Calc. Mass	mDa	PPM	DBE	i-FIT	Norm	Conf(%)	Formula
317.1724	317.1729	-0.5	-1.6	4.5	131.3	1.071	34.25	C17 H26 O4Na

Fig. S80. IR spectrum of **9**

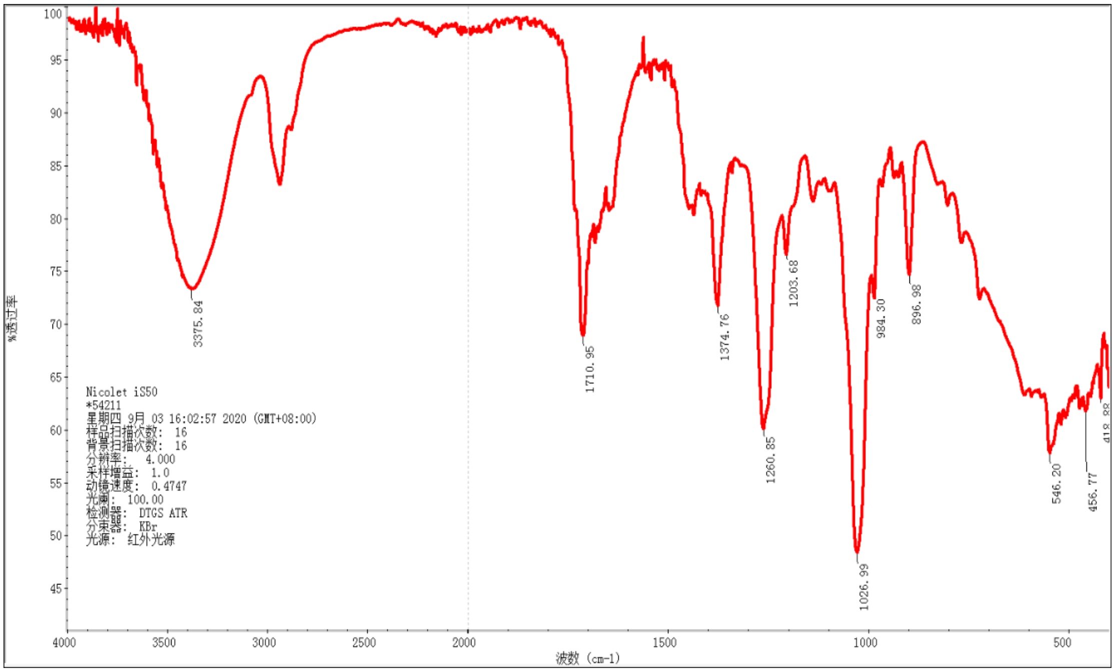

Fig. S81. UV spectrum of **9**

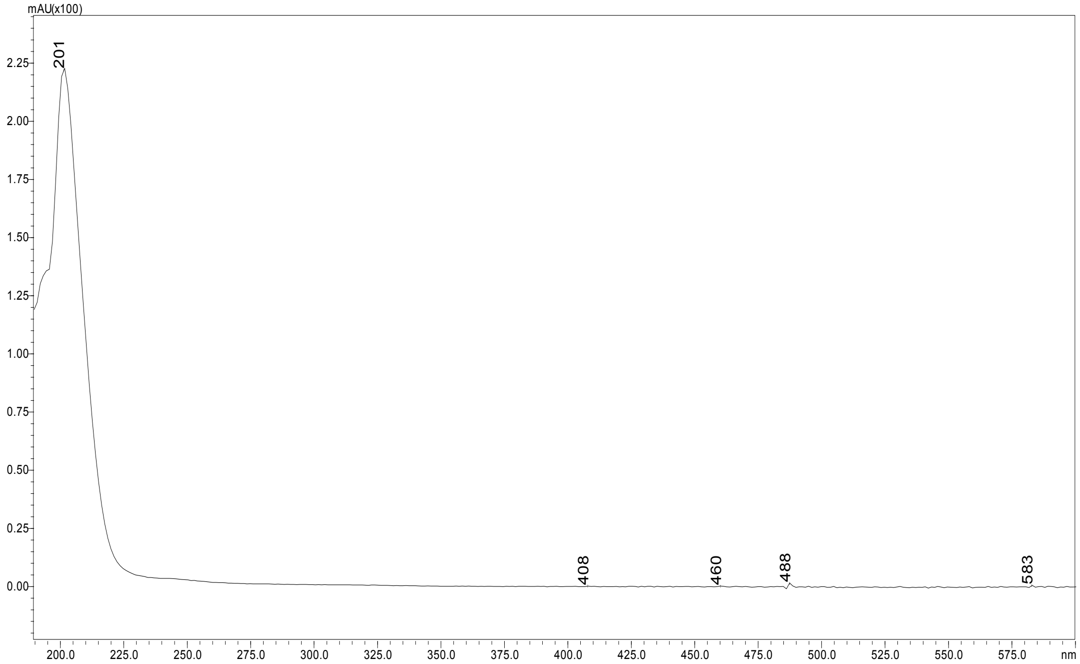

Fig. S82. ^{1}H NMR spectrum of **10** (400 MHz, DMSO-d6)

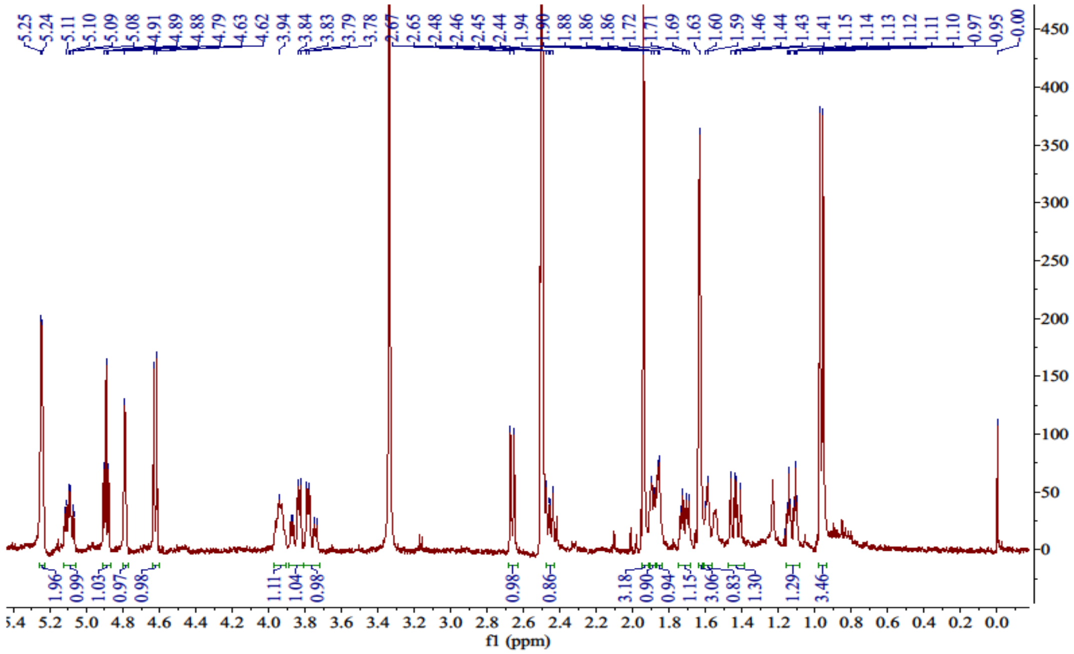

Fig. S83. ^{13}C NMR (APT) spectrum of **10** (100 MHz, DMSO-d6)

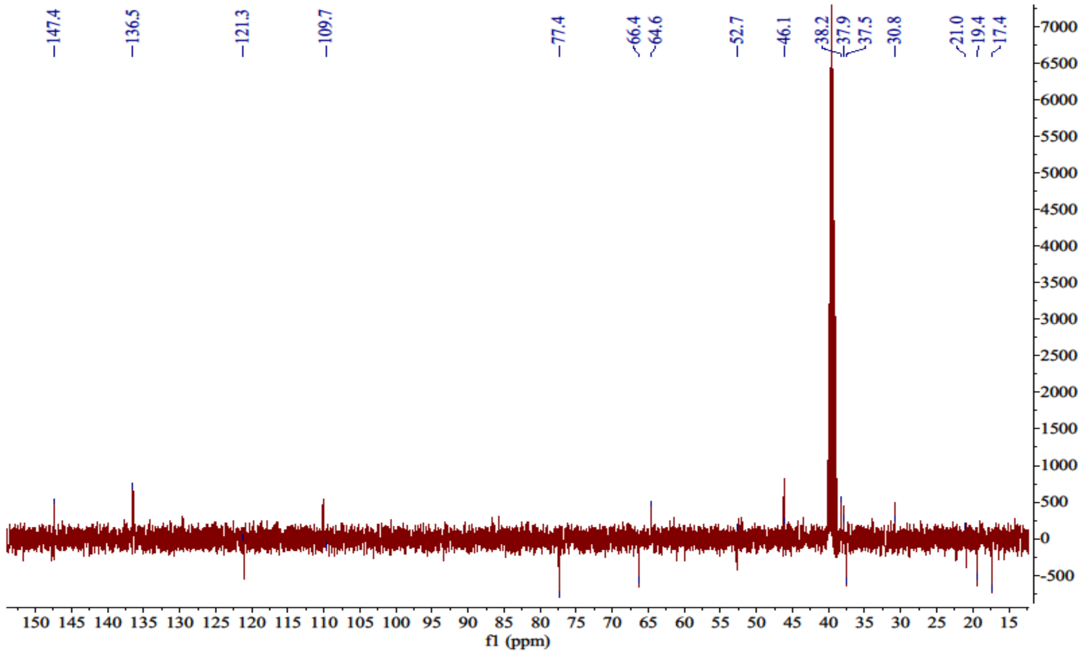

Fig. S84. ^{1}H-^{1}H COSY spectrum of **10**

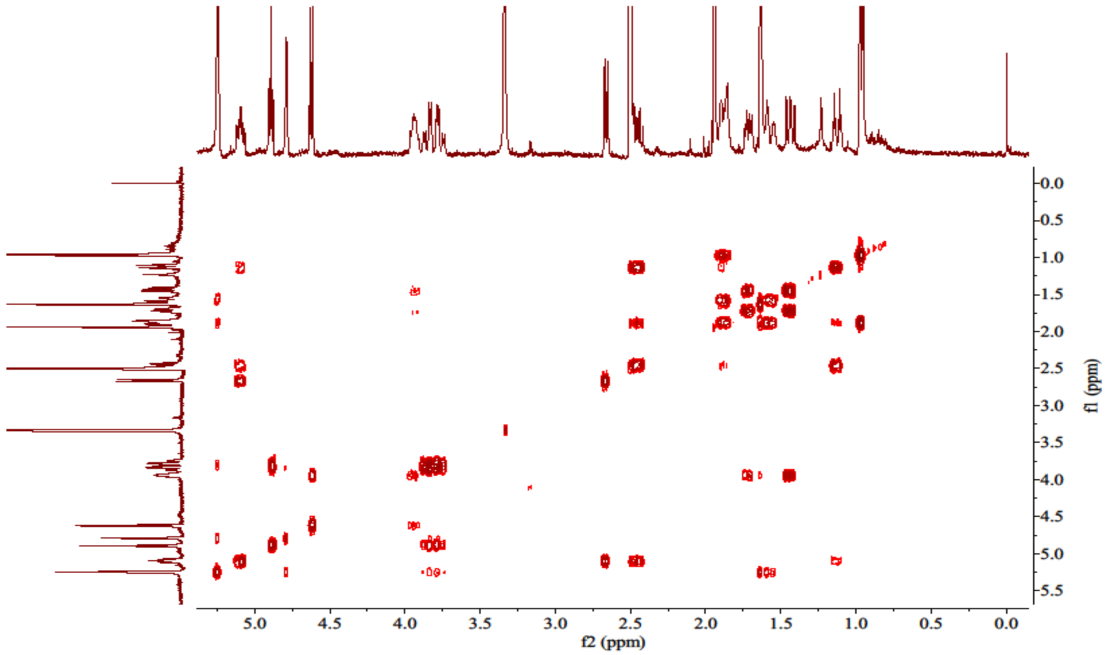

Fig. S85. HSQC spectrum of **10**

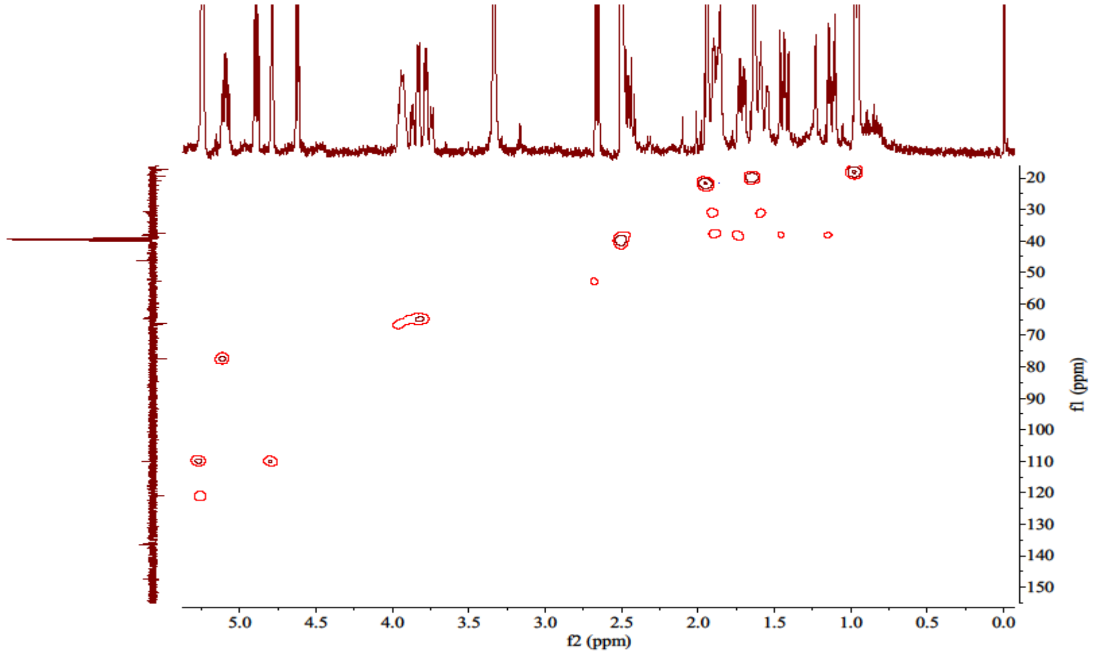

Fig. S86. HMBC spectrum of **10**

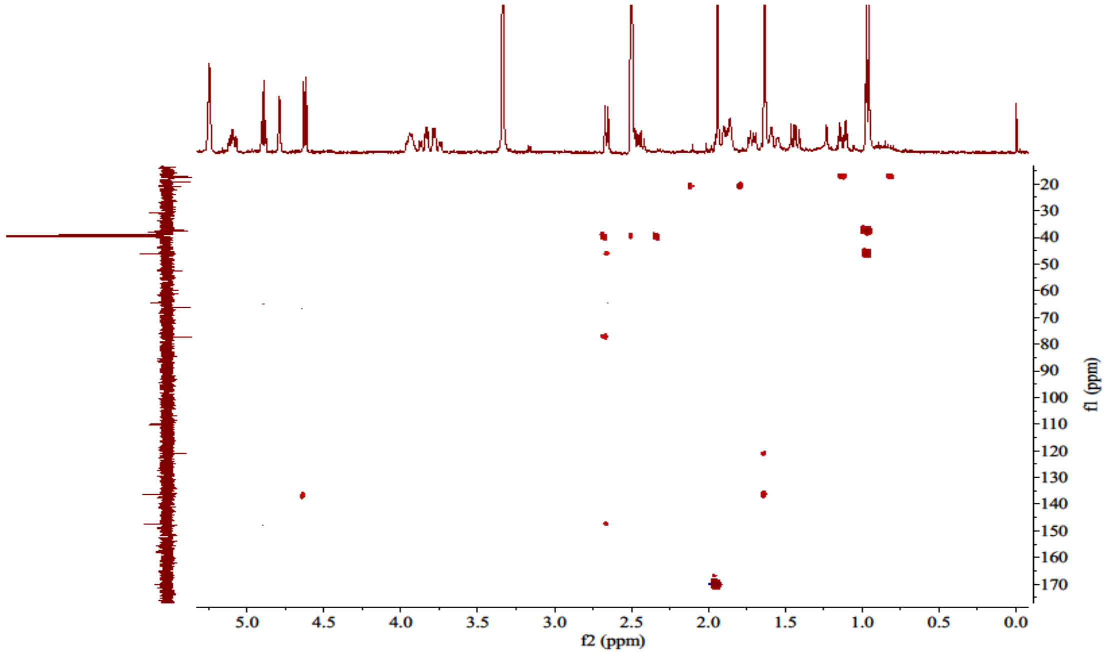

Fig. S87. NOESY spectrum of **10**

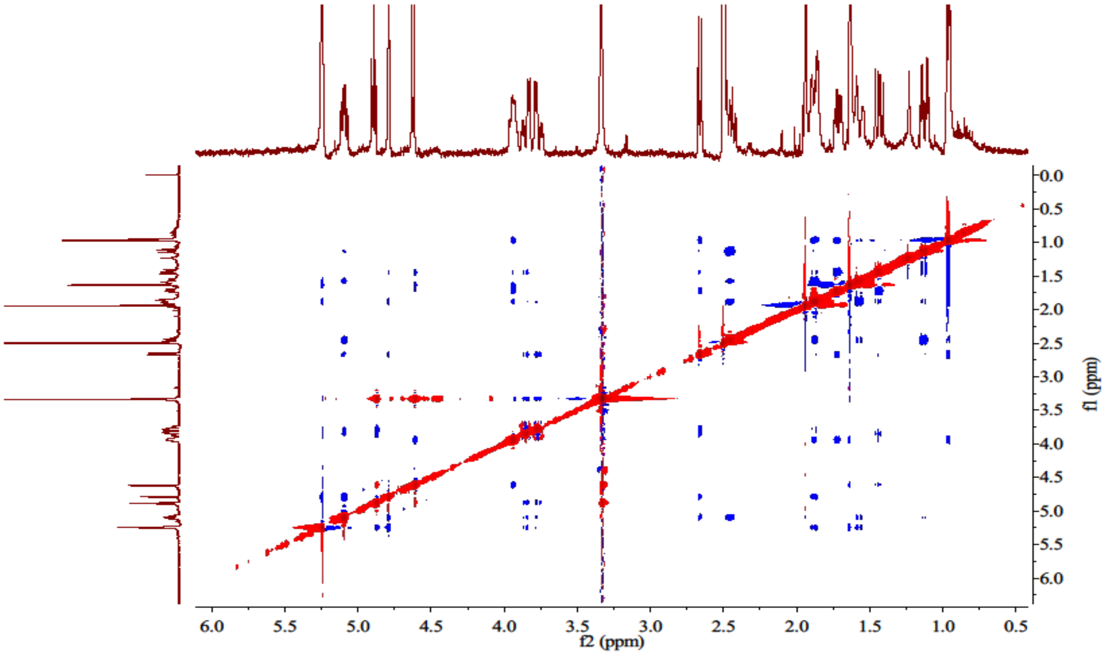

Fig. S88. HRESIMS spectrum of **10**

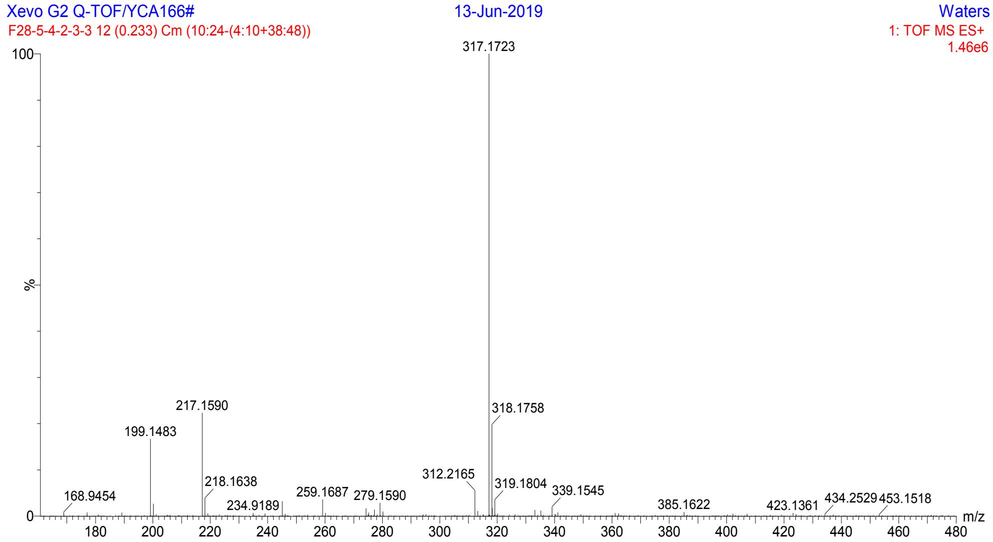

Mass	Calc. Mass	mDa	PPM	DBE	i-FIT	Norm	Conf(%)	Formula
317.1723	317.1729	-0.6	-1.9	4.5	469.8	0.039	96.21	C17 H26 O4 Na

Fig. S89. IR spectrum of **10**

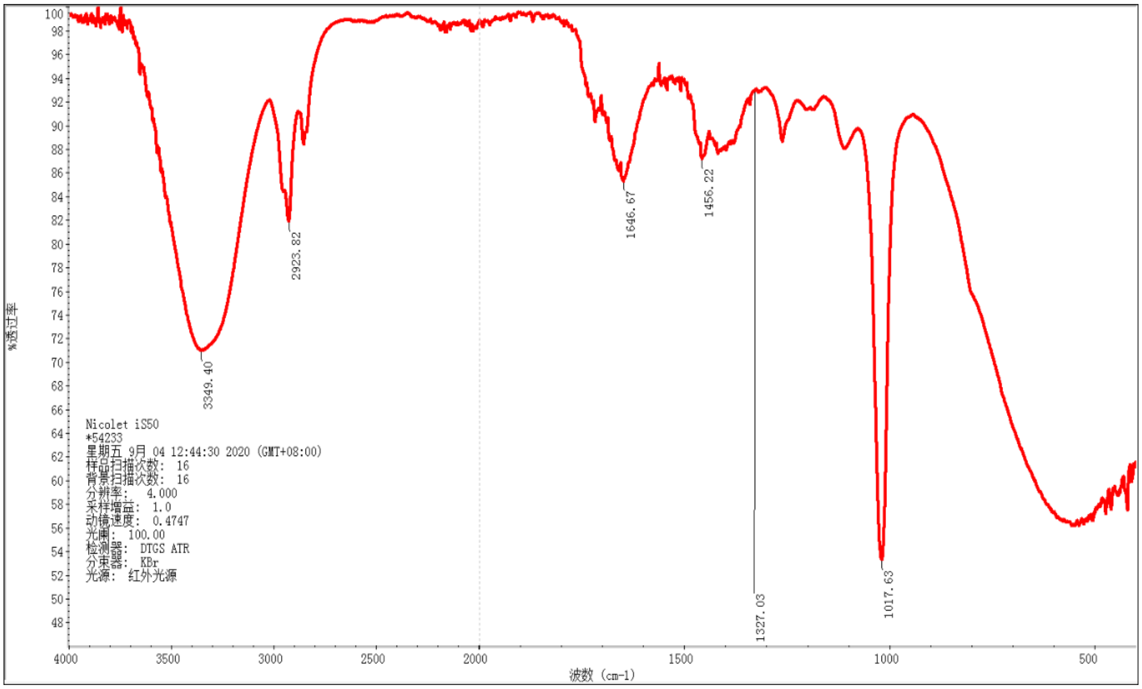

Fig. S90. UV spectrum of **10**

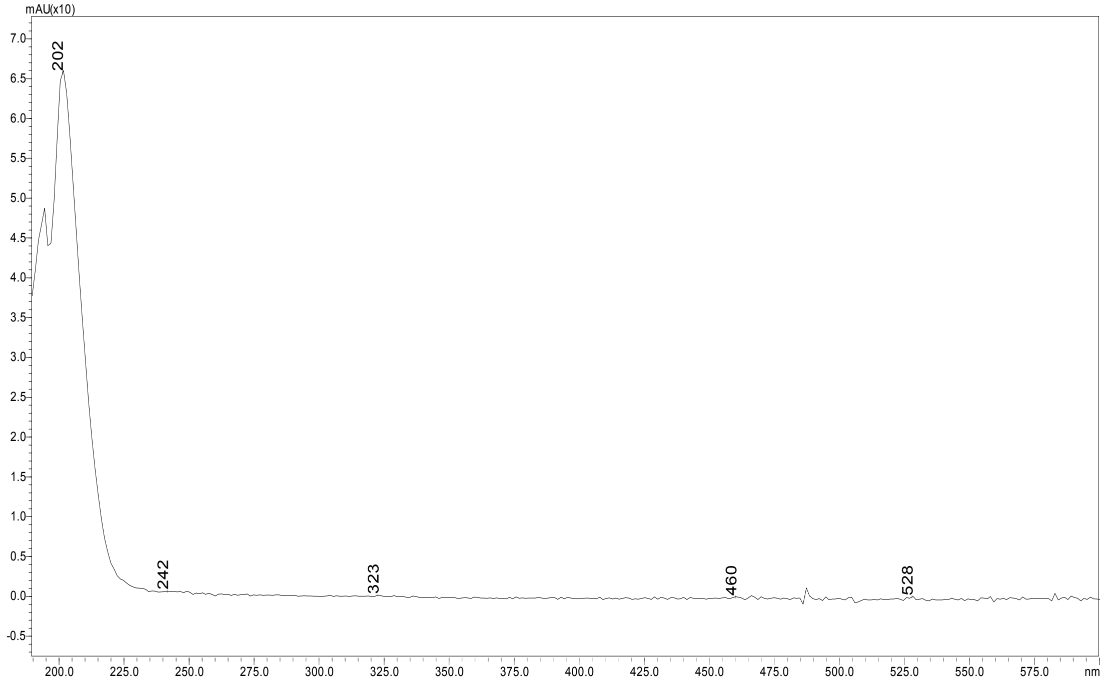

Fig. S91. ^{1}H NMR spectrum of **11** (400 MHz, DMSO-d6)

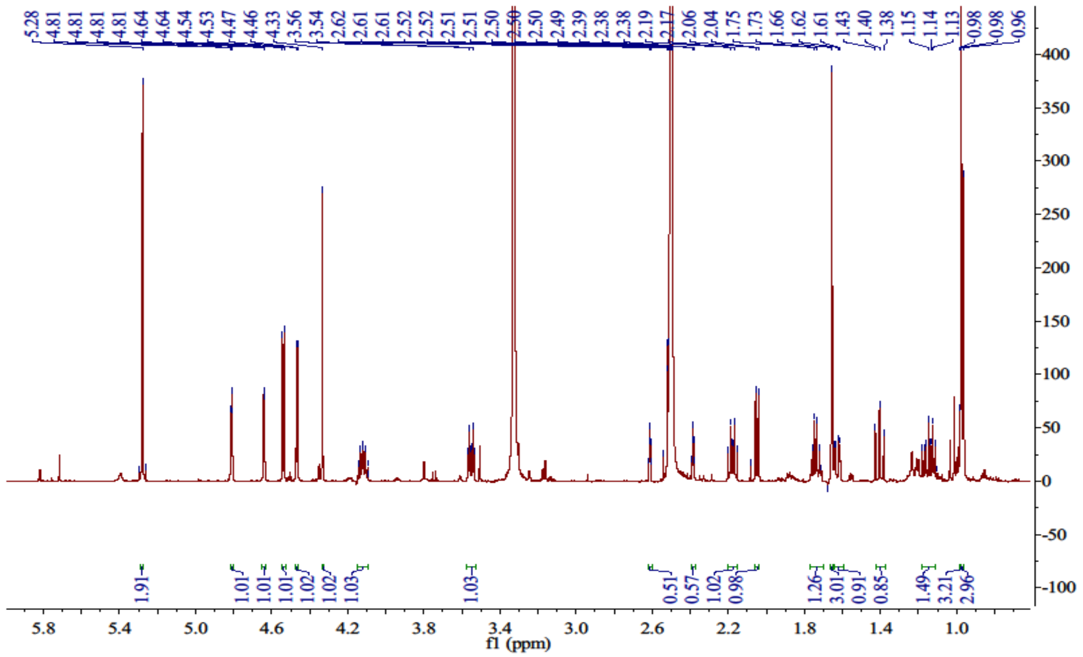

Fig. S92. ^{13}C NMR (APT) spectrum of **11** (100 MHz, DMSO-d6)

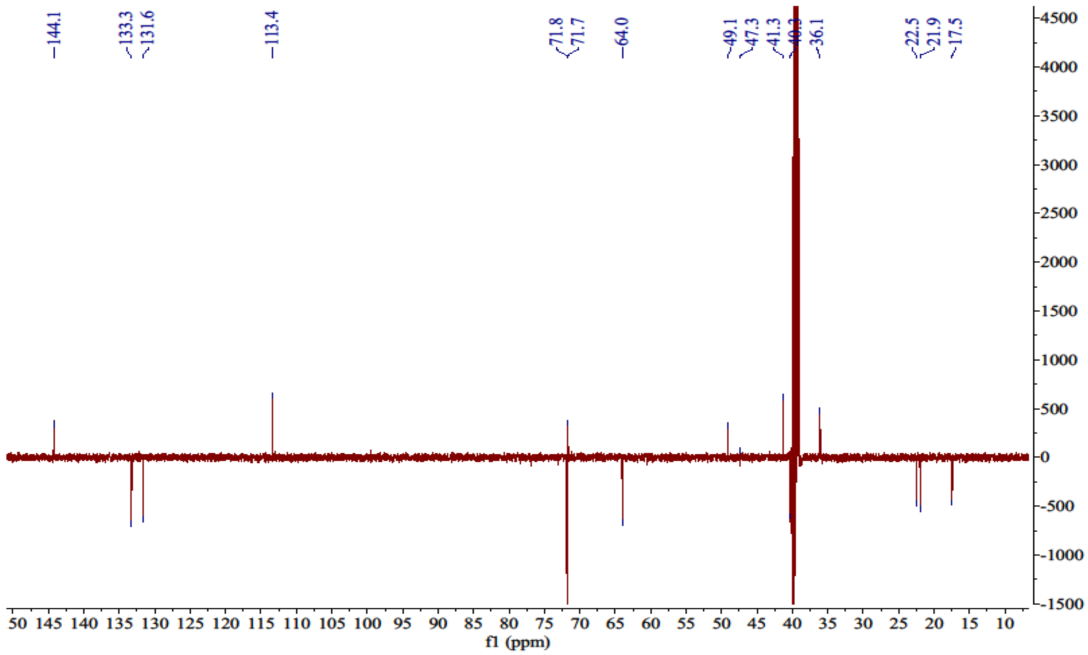

Fig. S93. ^1H-^1H COSY spectrum of **11**

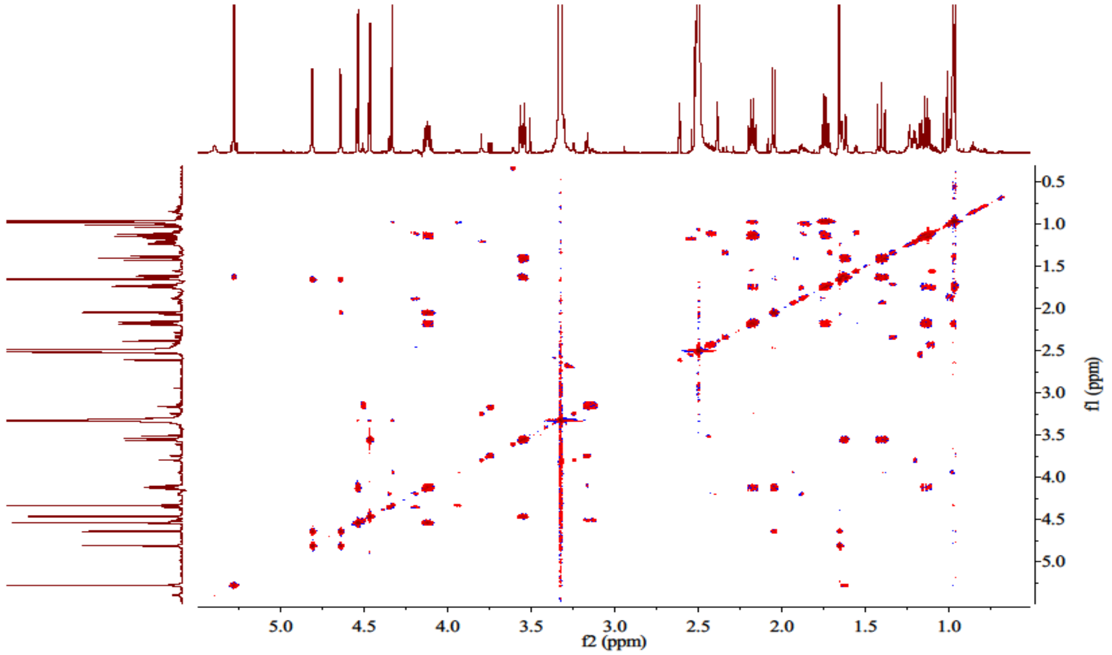

Fig. S94. HSQC spectrum of **11**

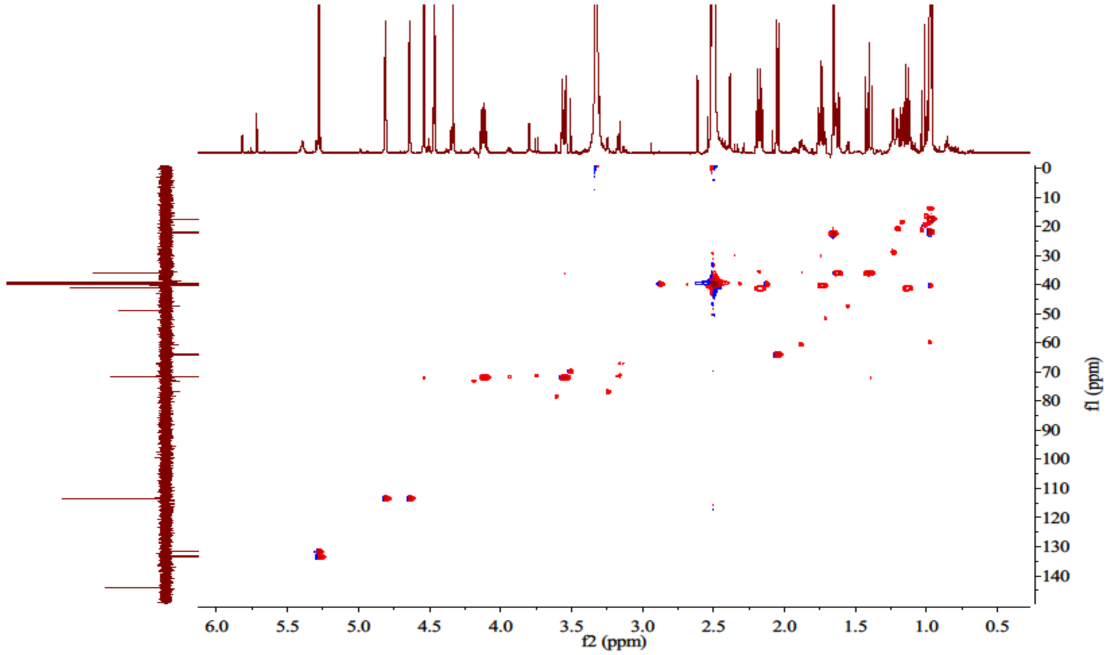

Fig. S95. HMBC spectrum of **11**

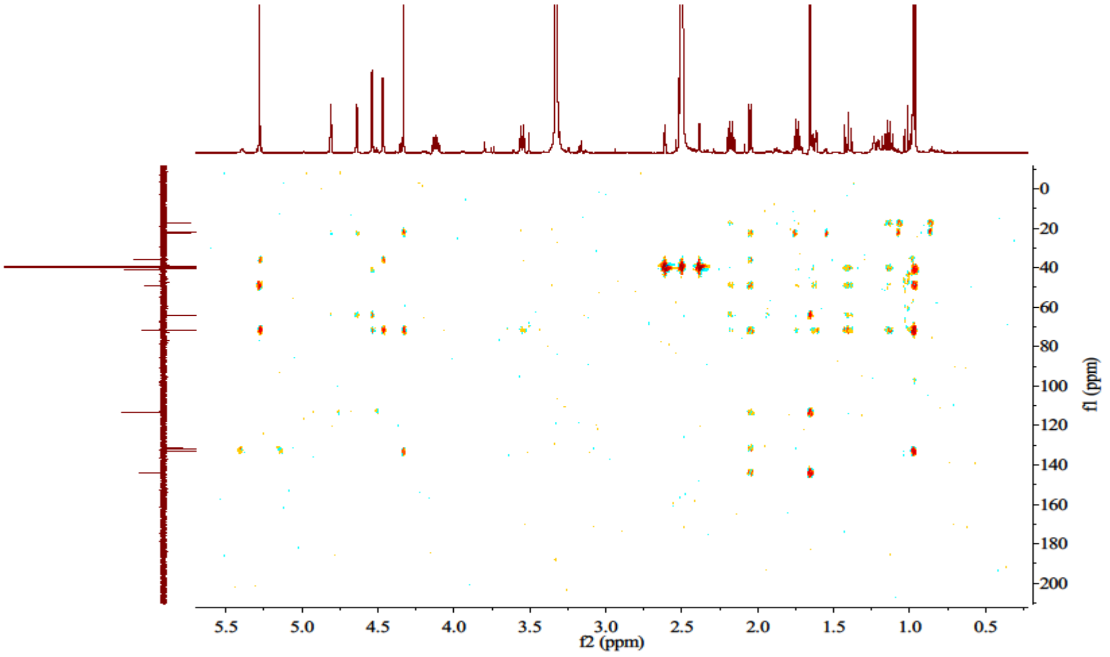

Fig. S96. NOESY spectrum of **11**

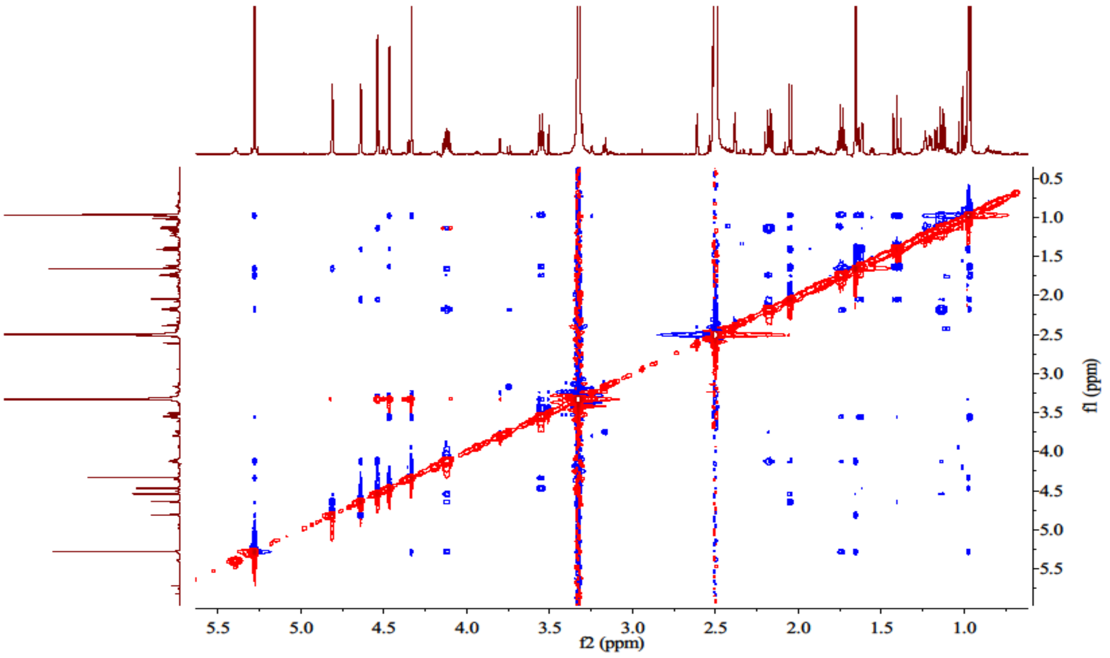

Fig. S97. HRESIMS spectrum of **11**

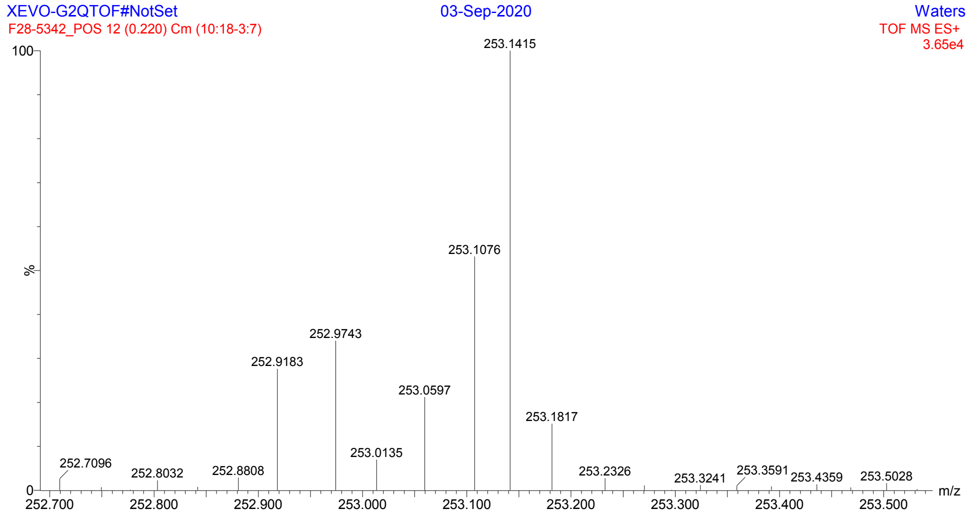

Mass	Calc. Mass	mDa	PPM	DBE	i-FIT	Norm	Conf(%)	Formula
253.1817	253.1804	1.3	5.1	3.5	95.7	n/a	n/a	C15 H25 O3

Fig. S98. IR spectrum of **11**

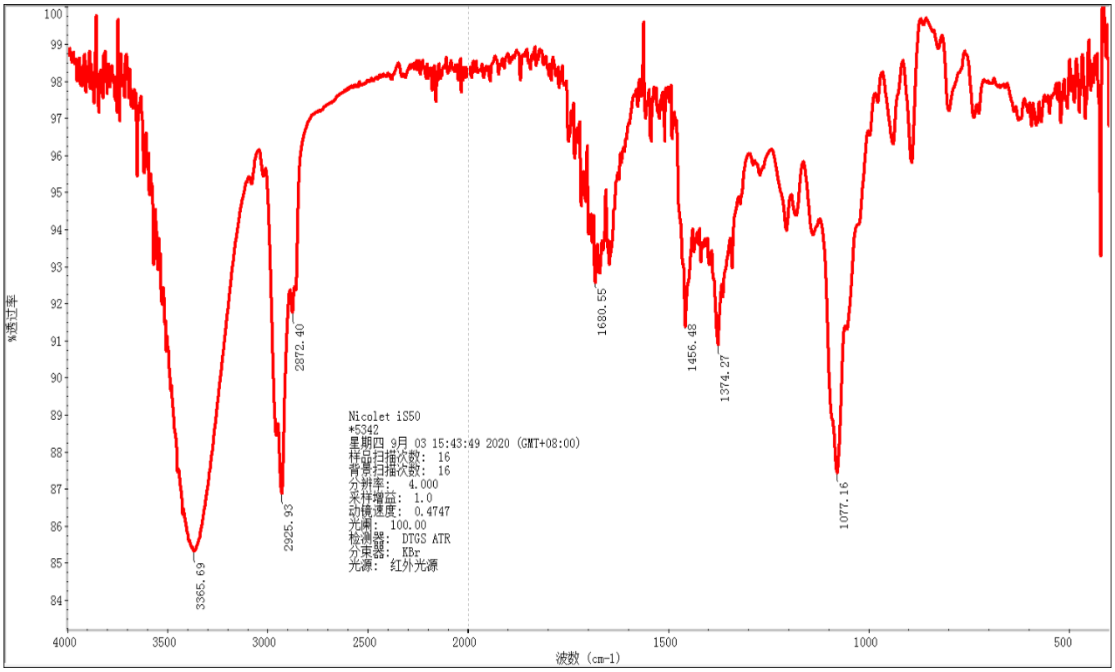

Fig. S99. UV spectrum of **11**

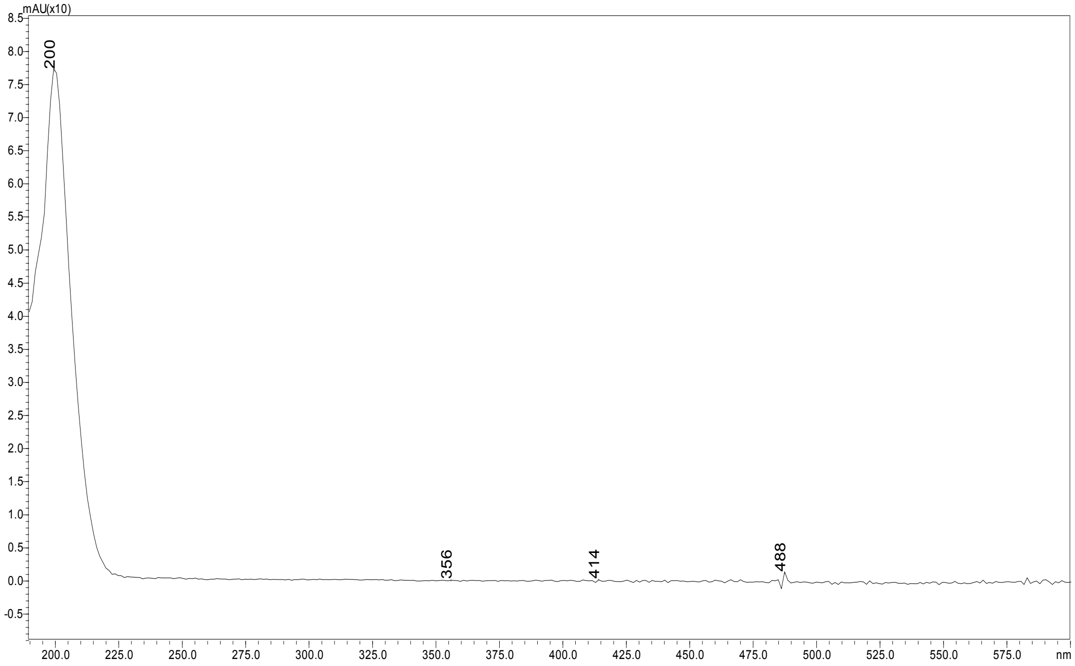

Fig. S100. ^{1}H NMR spectrum of **12** (400 MHz, DMSO-d6)

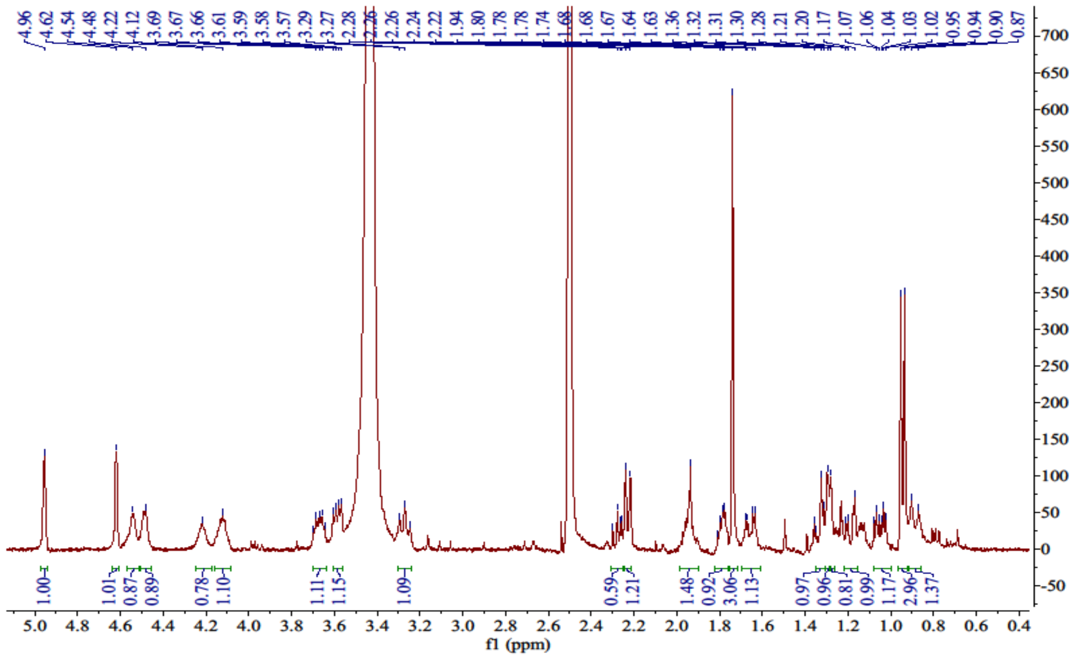

Fig. S101. ^{13}C NMR (APT) spectrum of **12** (100 MHz, DMSO-d6)

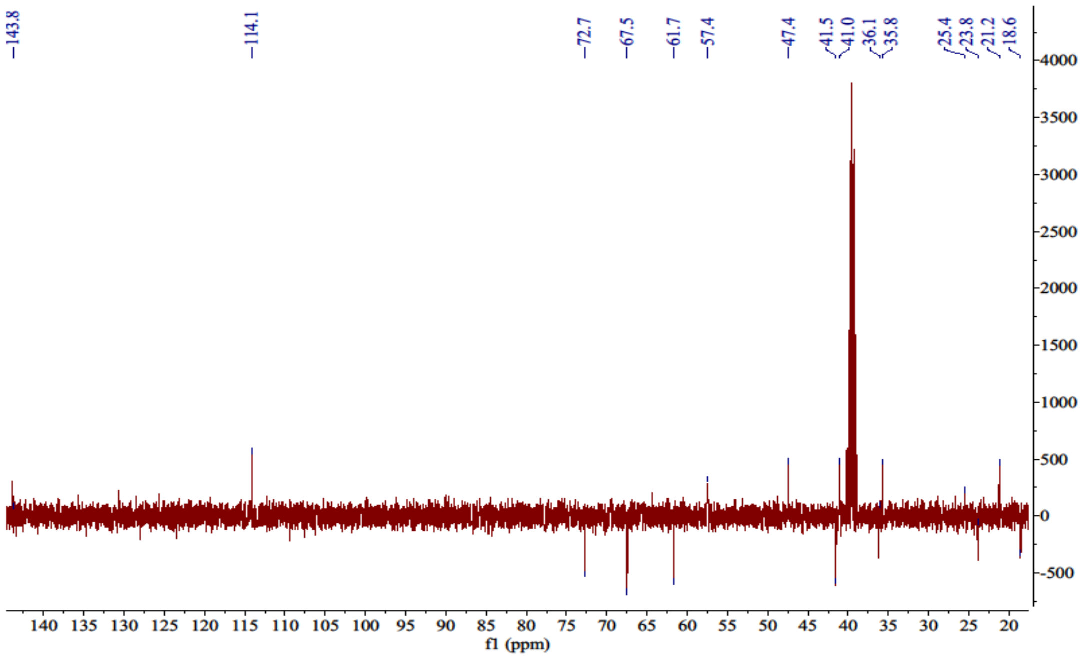

Fig. S102. ^{1}H-^{1}H COSY spectrum of **12**

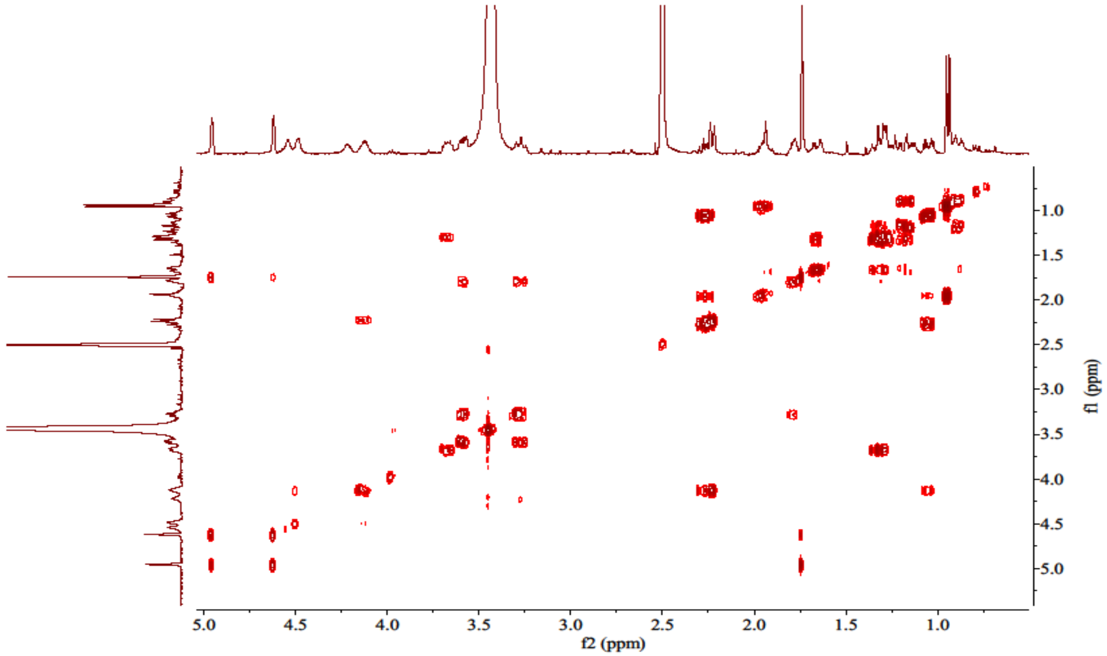

Fig. S103. HSQC spectrum of **12**

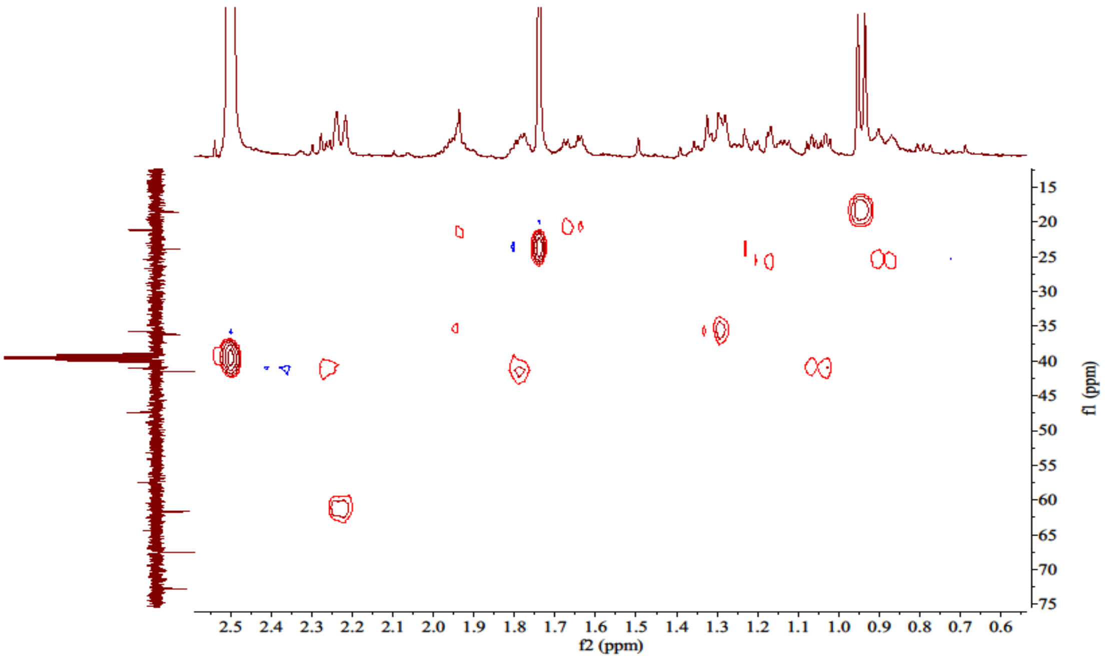

Fig. S104. HMBC spectrum of **12**

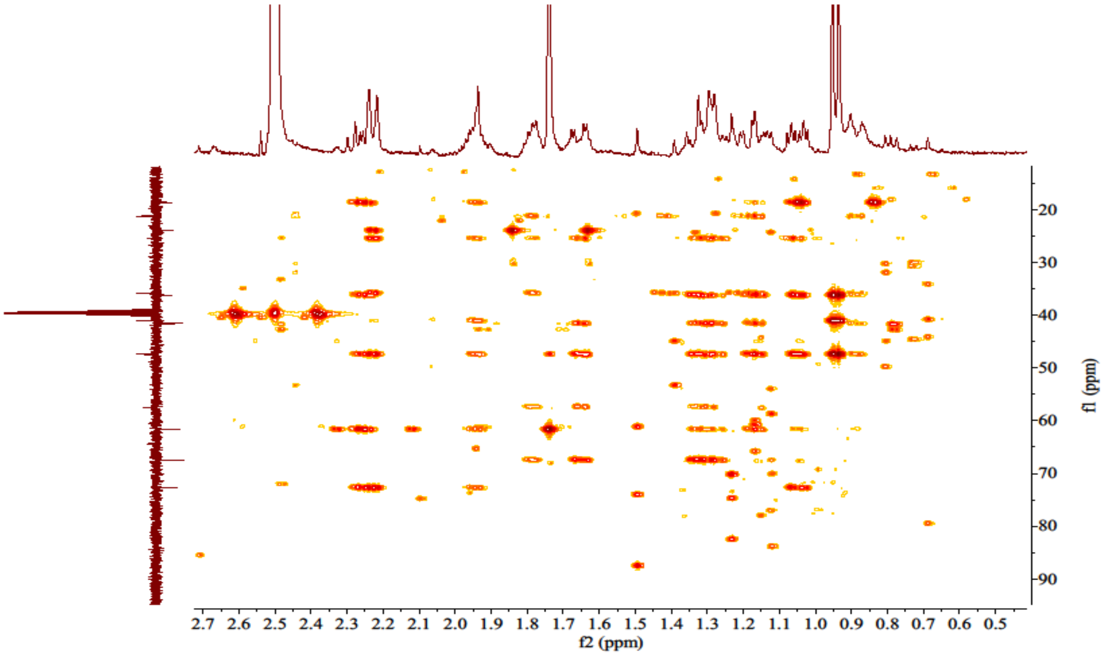

Fig. S105. NOESY spectrum of **12**

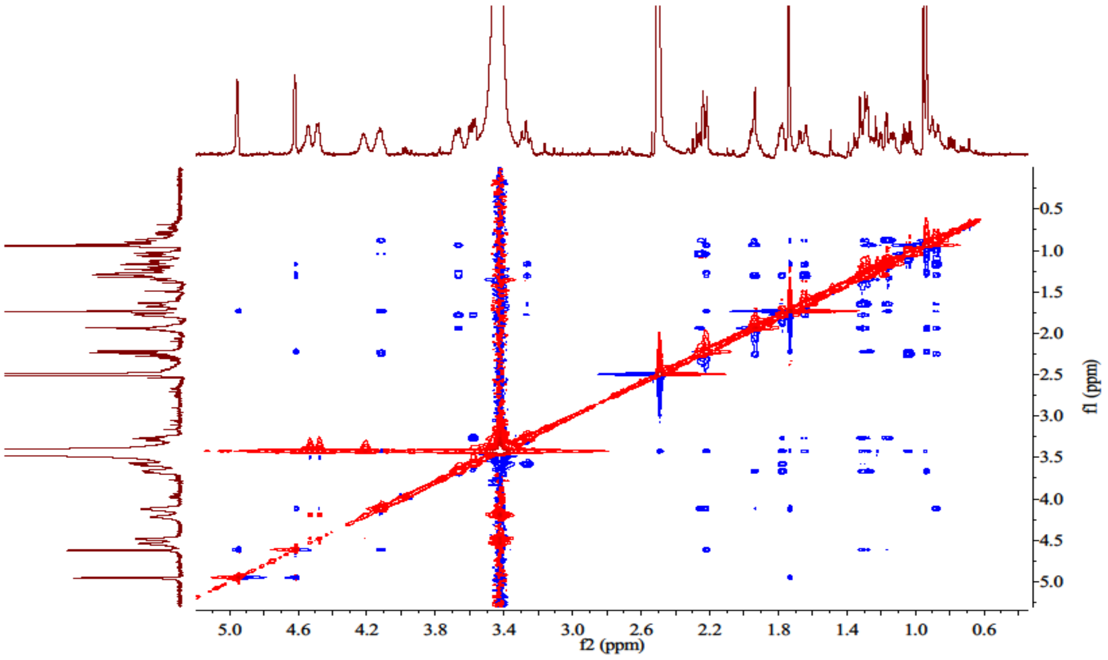

Fig. S106. HRESIMS spectrum of **12**

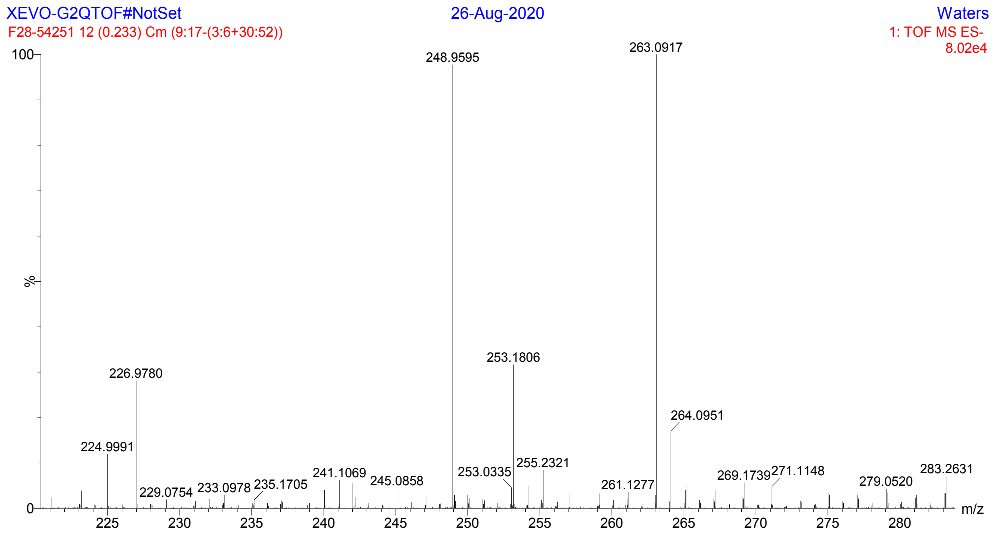

Mass	Calc. Mass	mDa	PPM	DBE	i-FIT	Norm	Conf(%)	Formula
253.1806	253.1804	0.2	0.8	3.5	390.9	n/a	n/a	C15 H25 O3

Fig. S107. IR spectrum of **12**

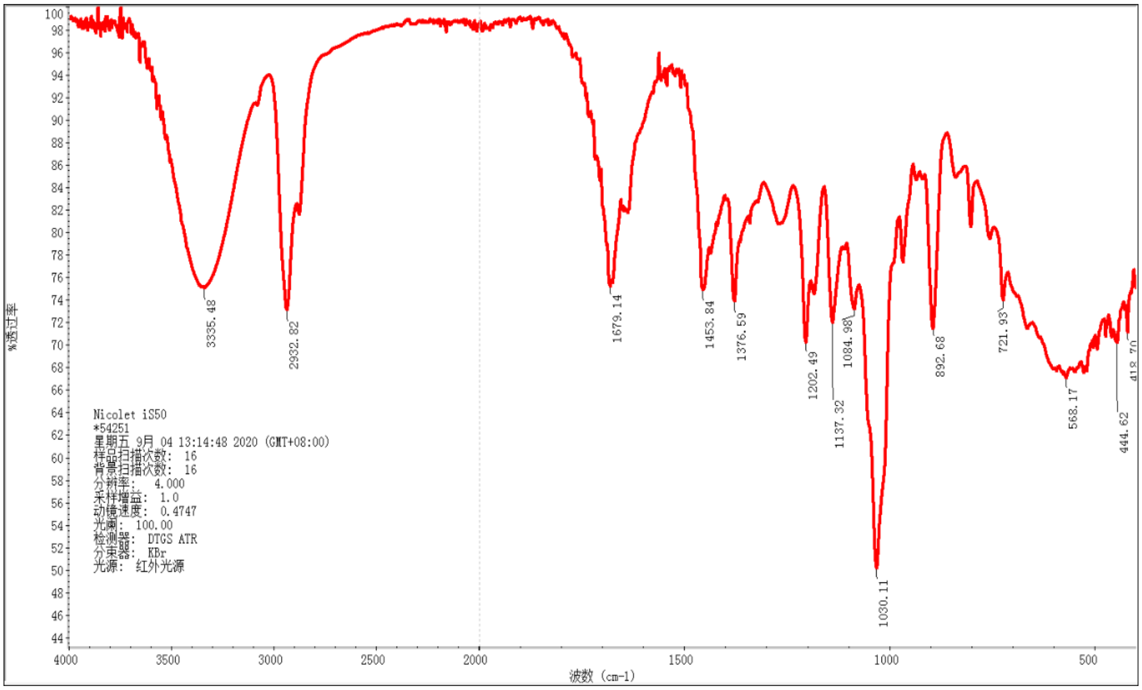

Fig. S108. UV spectrum of **12**

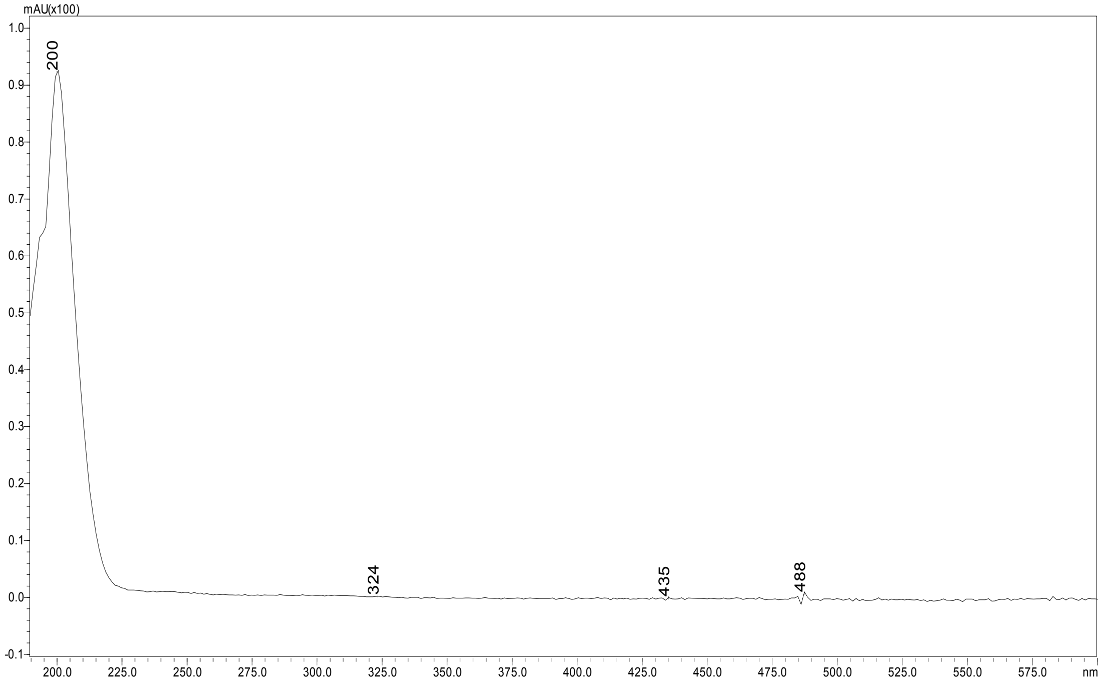

Fig. S109. ^{1}H NMR spectrum of **13** (400 MHz, DMSO-d6)

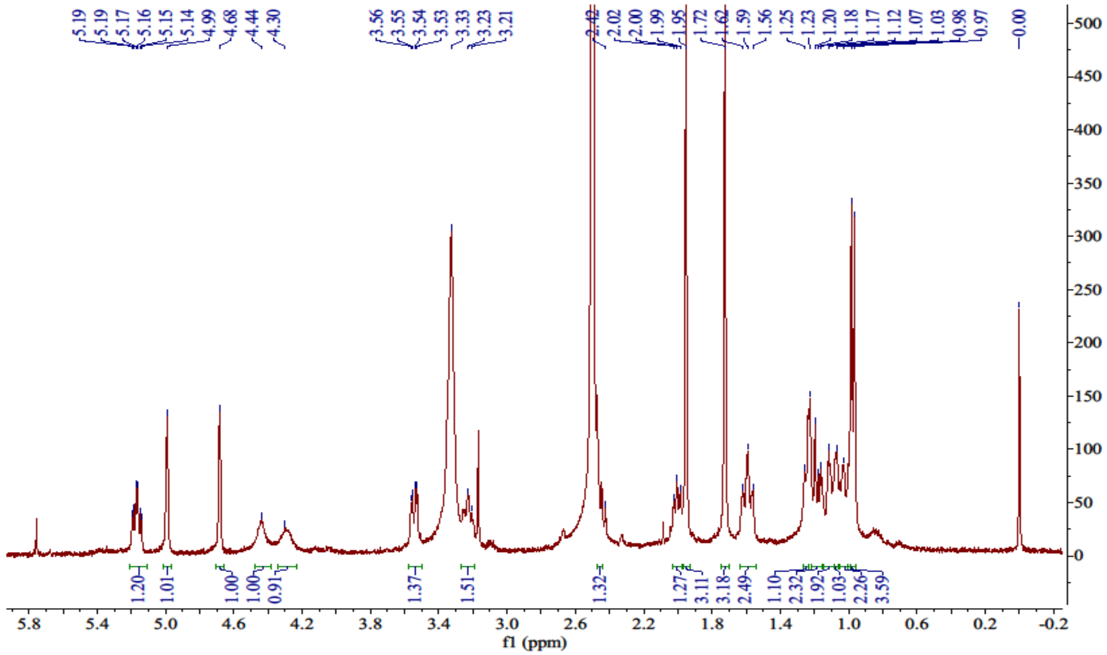

Fig. S110. ^{13}C NMR (APT) spectrum of **13** (100 MHz, DMSO-d6)

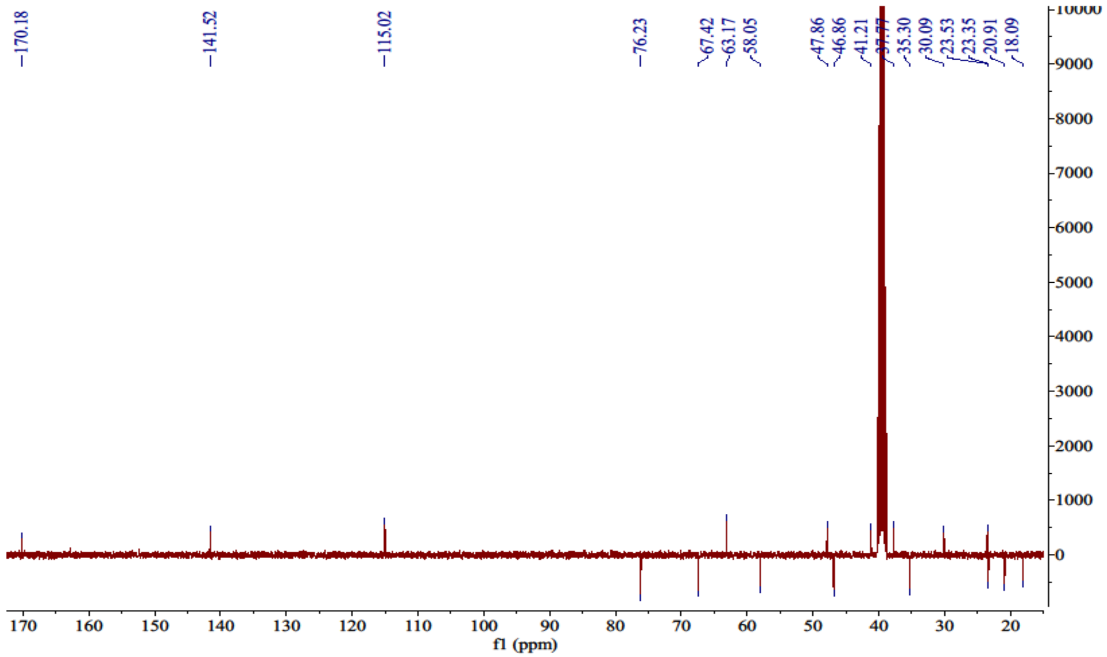

Fig. S111. ^{1}H-^{1}H COSY spectrum of **13**

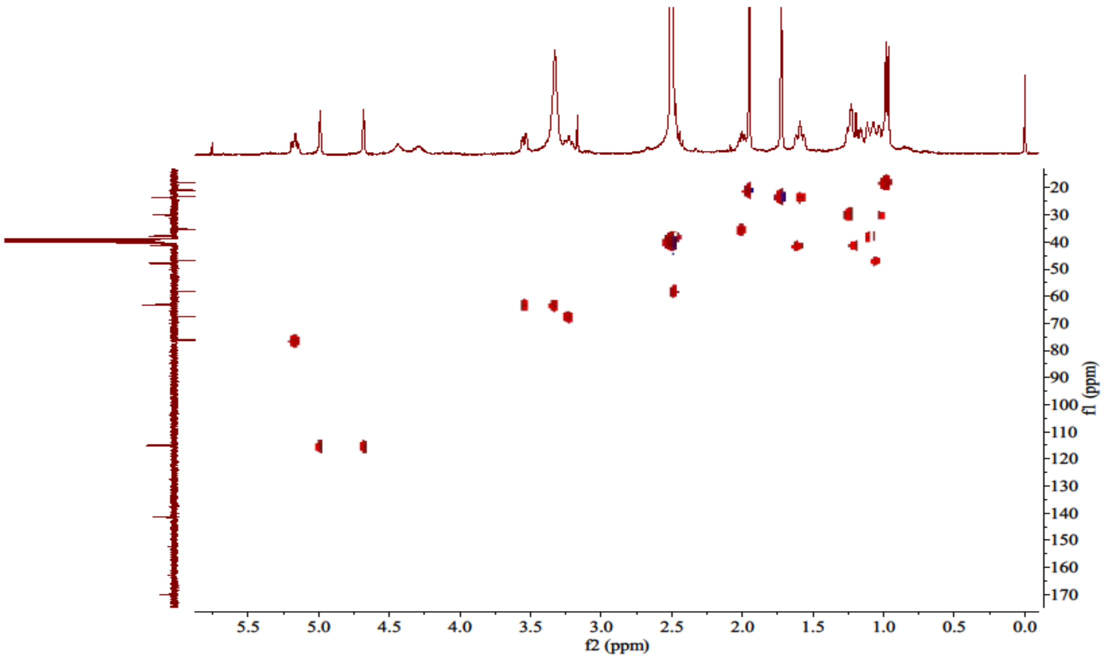

Fig. S112. HSQC spectrum of **13**

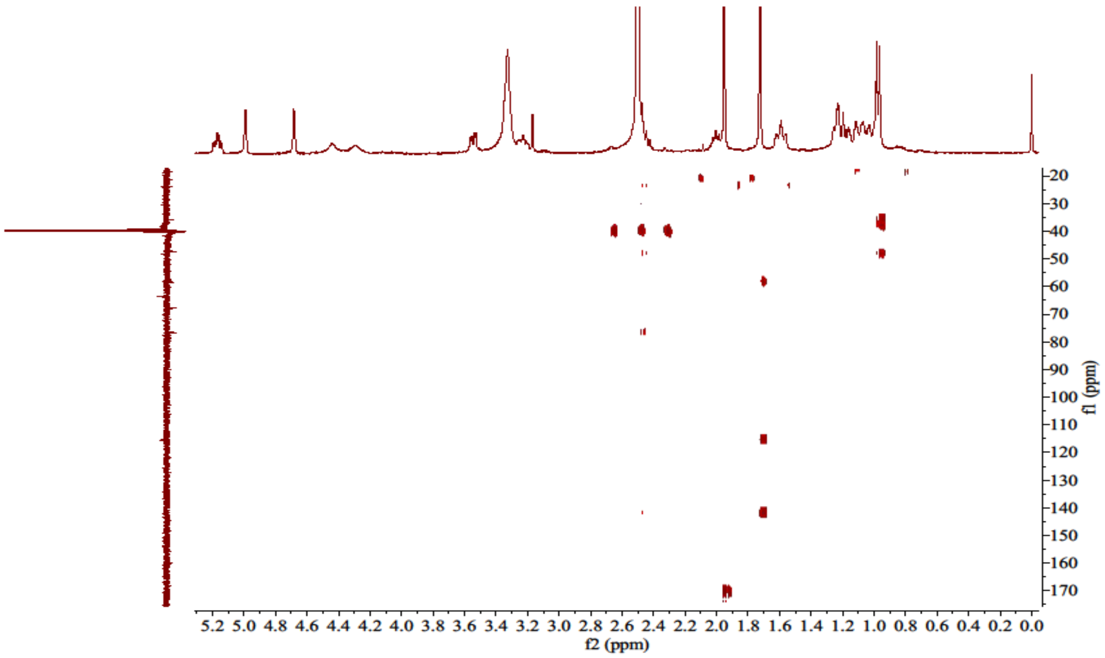

Fig. S113. HMBC spectrum of **13**

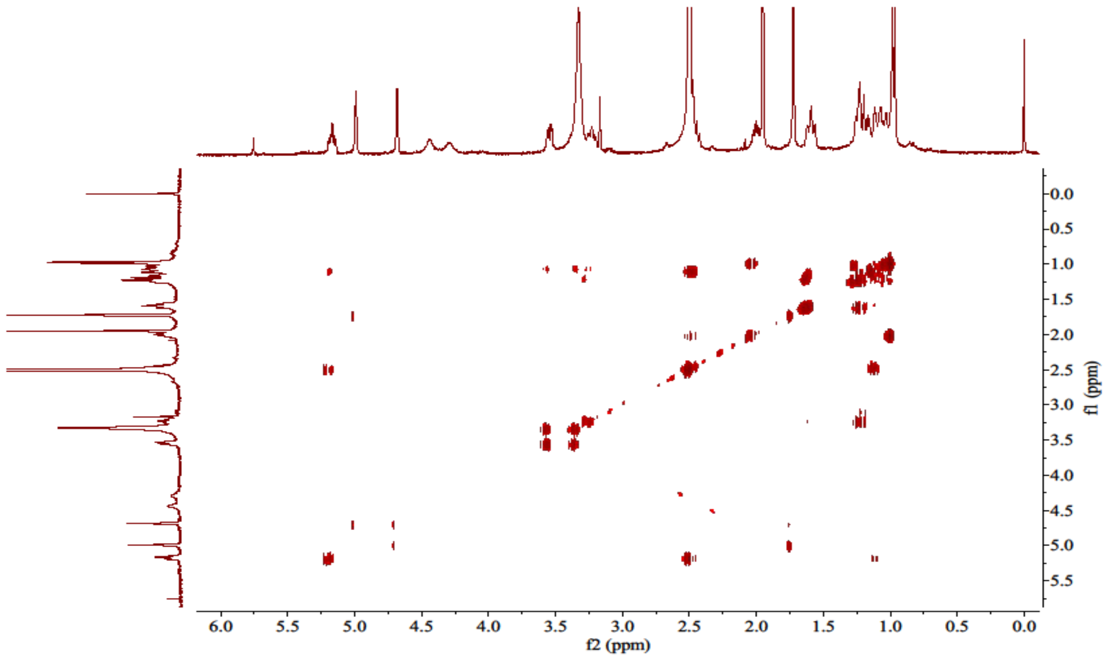

Fig. S114. NOESY spectrum of **13**

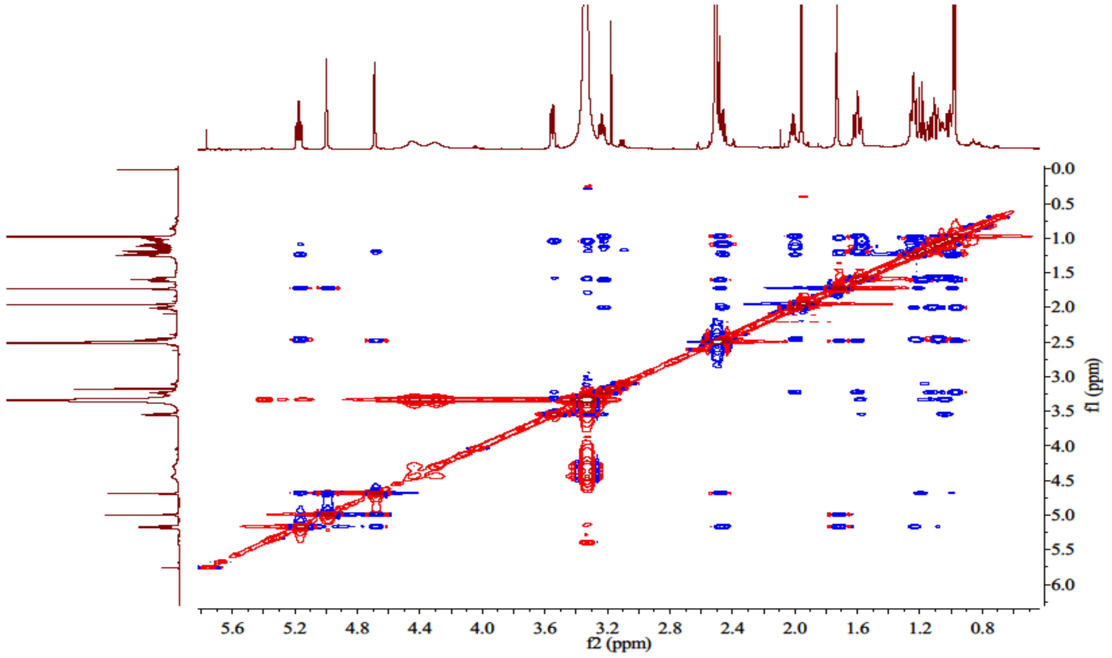

Fig. S115. HRESIMS spectrum of **13**

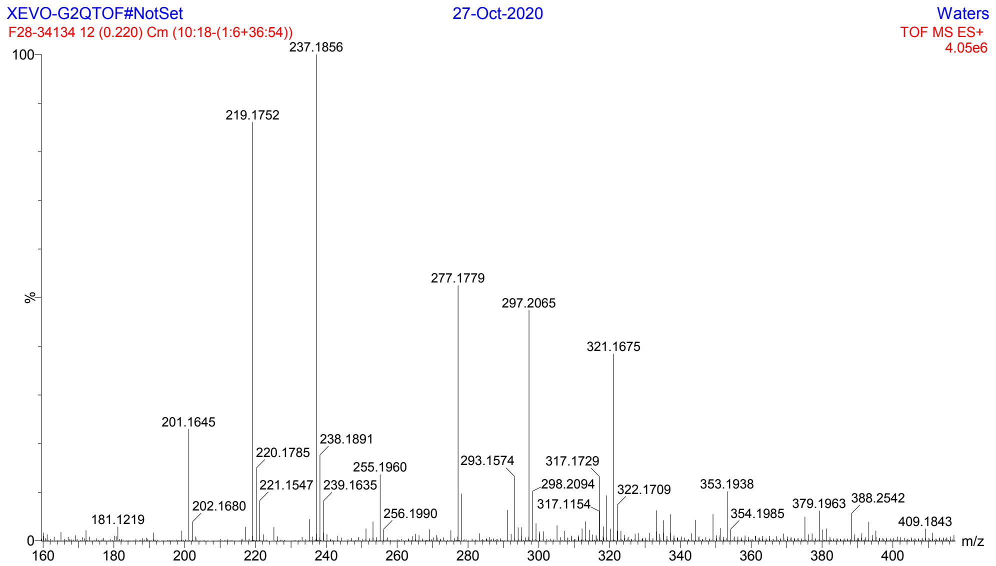

Mass	Calc. Mass	mDa	PPM	DBE	i-FIT	Norm	Conf(%)	Formula
297.2065	297.2066	-0.1	-0.3	3.5	451.8	0.581	55.93	C17 H29 O4

Fig. S116. IR spectrum of **13**

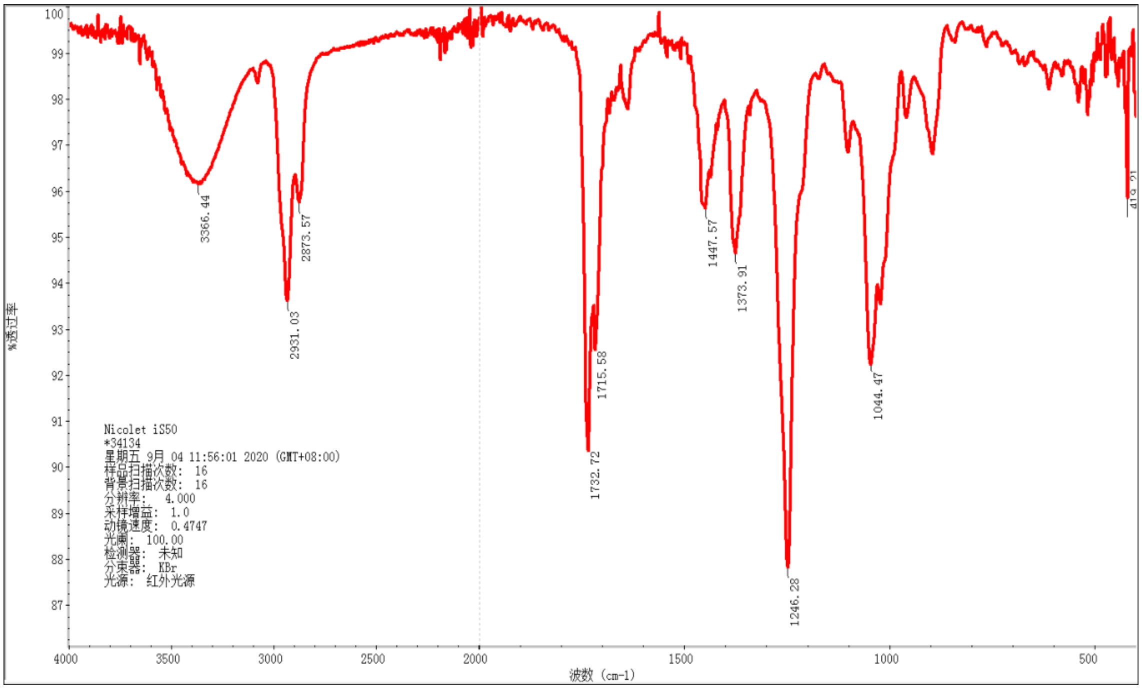

Fig. S117. UV spectrum of **13**

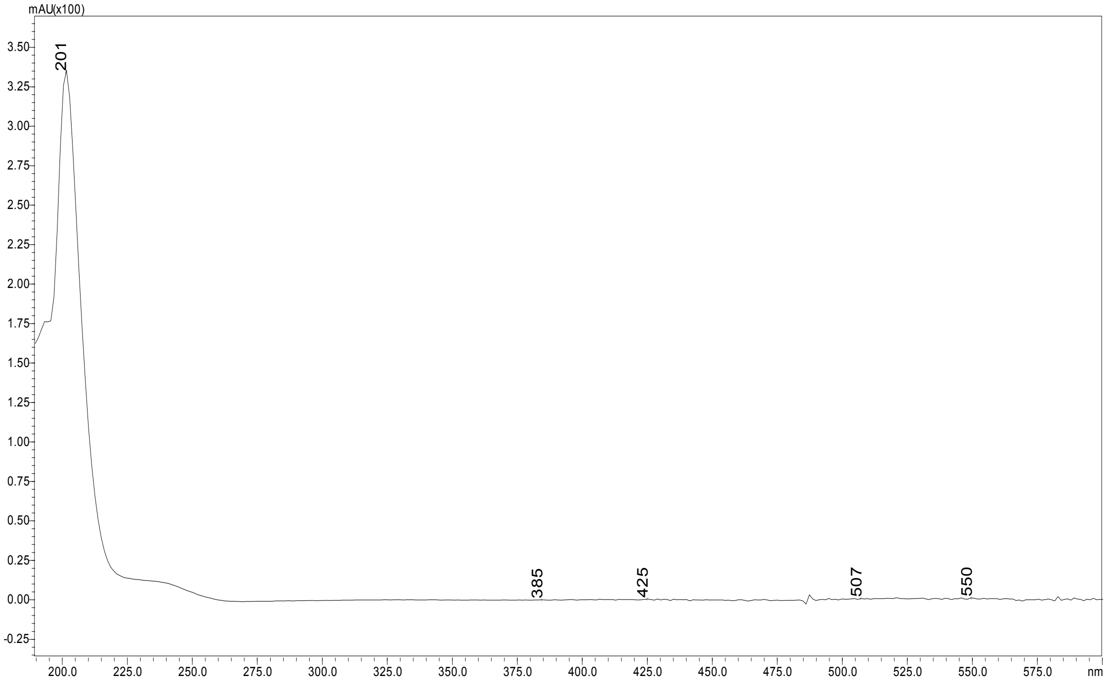

Fig. S118. ^{1}H NMR spectrum of **14** (400 MHz, DMSO-d6)

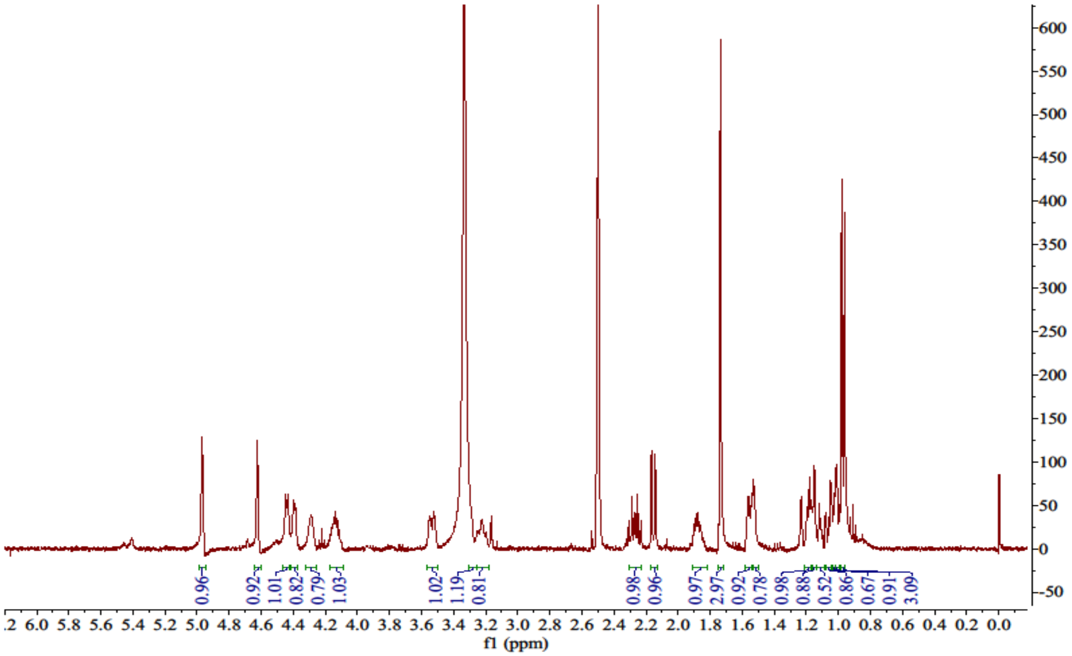

Fig. S119. ^{13}C NMR (APT) spectrum of **14** (100 MHz, DMSO-d6)

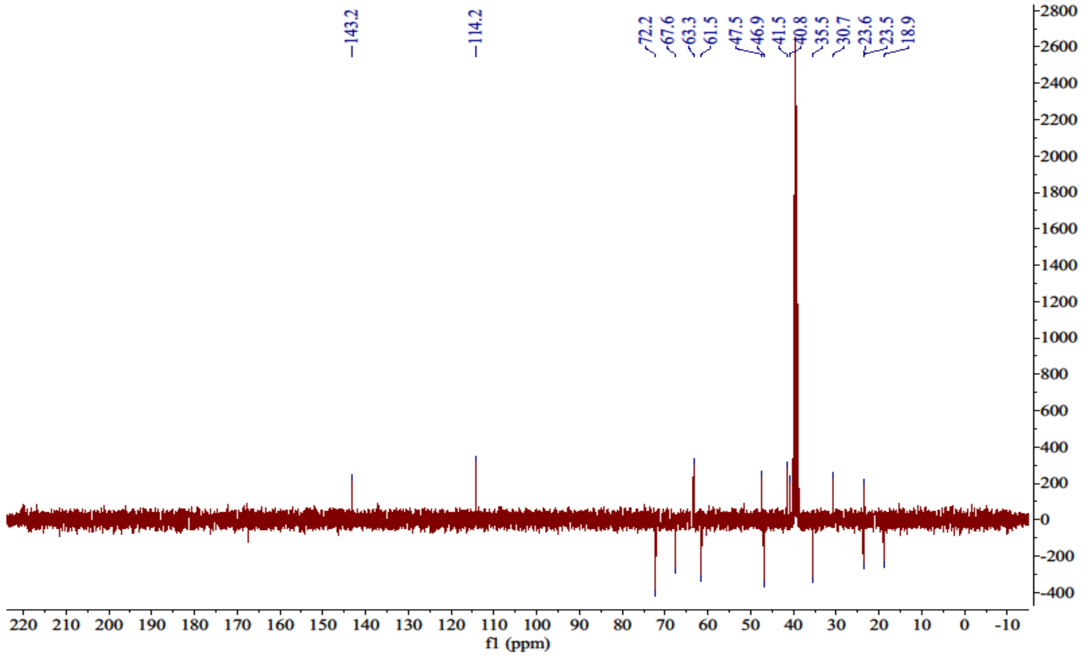

Fig. S120. ^{1}H-^{1}H COSY spectrum of **14**

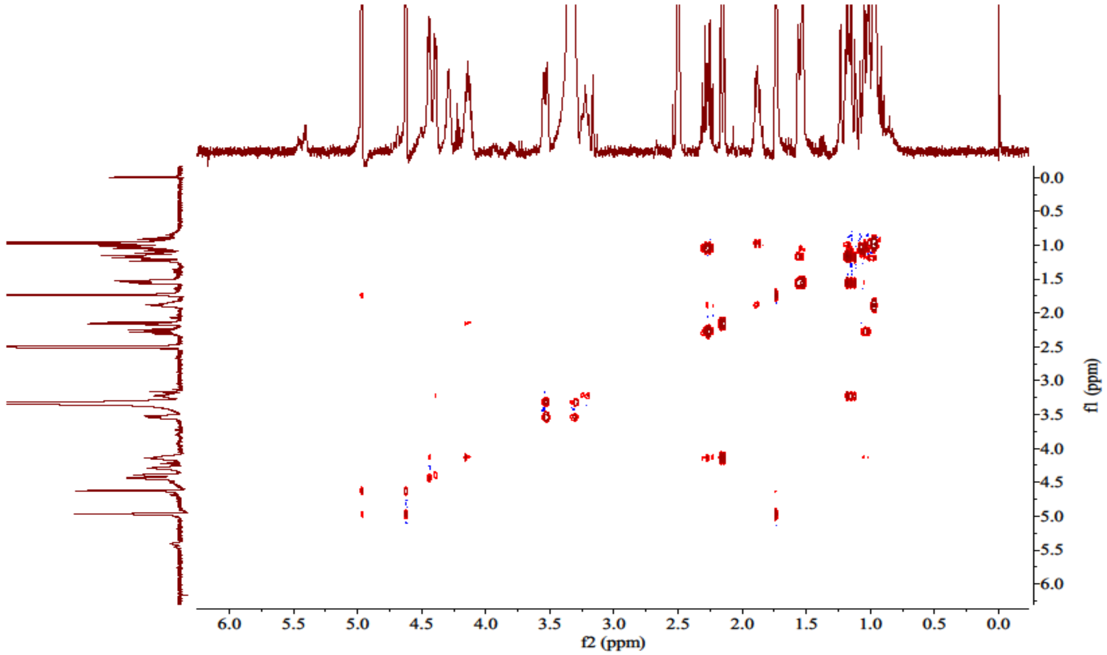

Fig. S121. HSQC spectrum of **14**

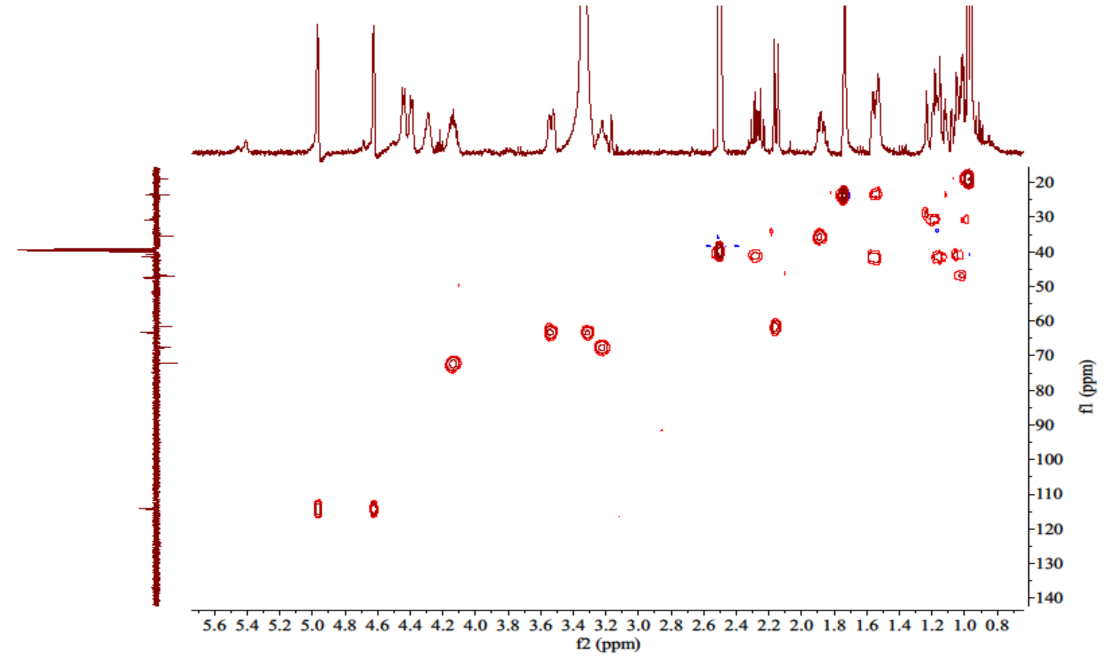

Fig. S122. HMBC spectrum of **14**

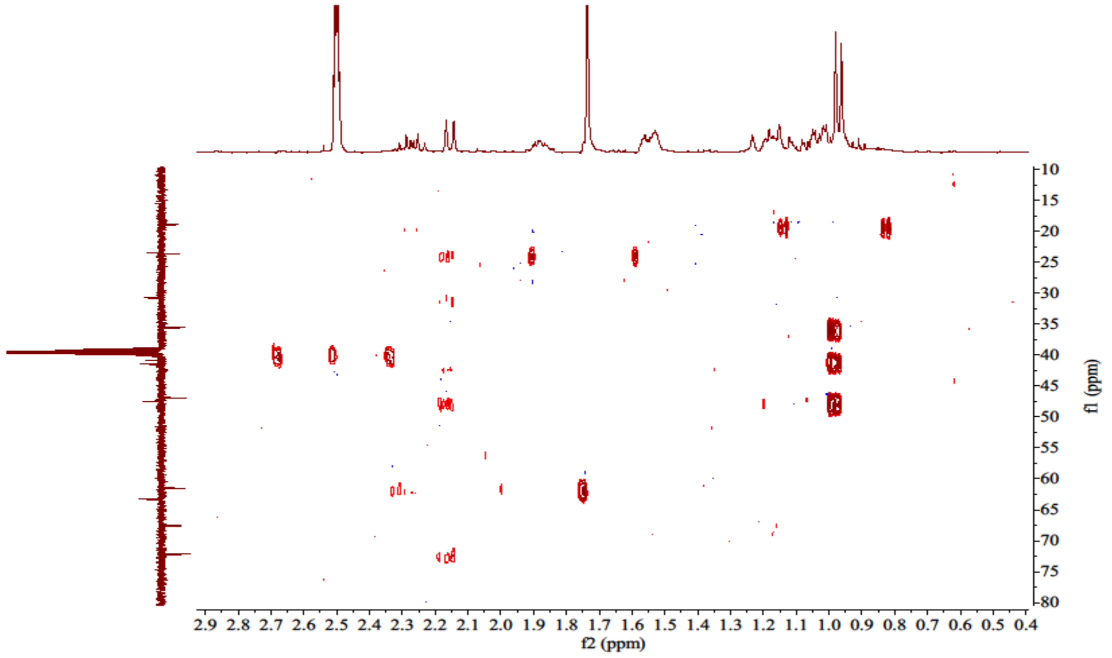

Fig. S123. NOESY spectrum of **14**

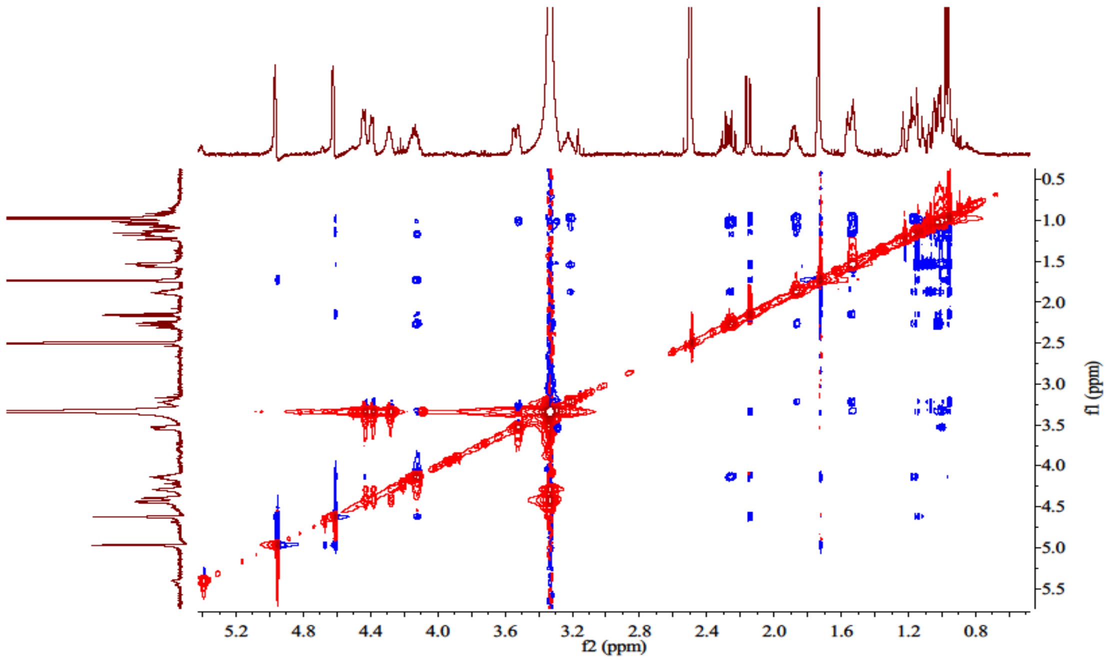

Fig. S124. HRESIMS spectrum of **14**

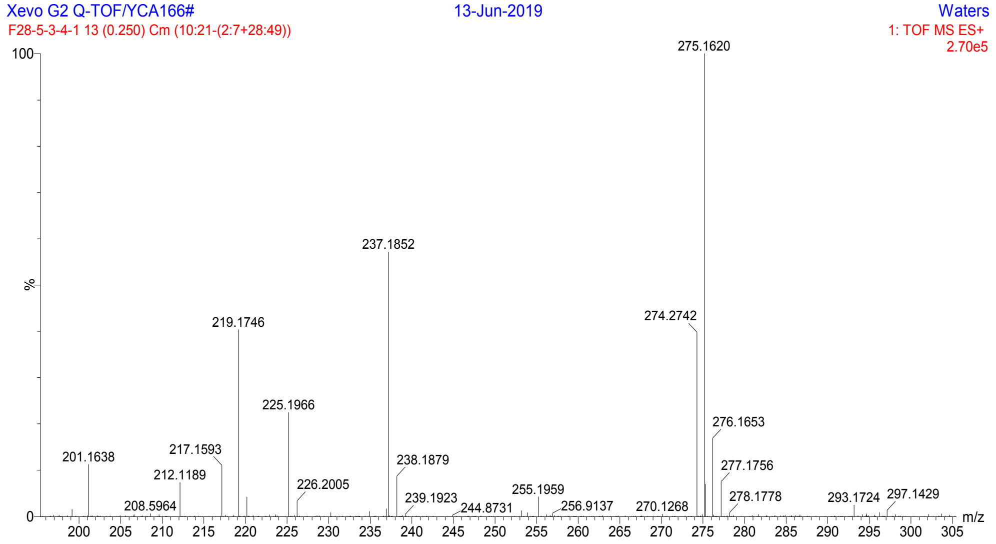

Mass	Calc. Mass	mDa	PPM	DBE	i-FIT	Norm	Conf(%)	Formula
255.1959	255.1960	-0.1	-0.4	2.5	126.3	0.007	99.33	C15 H27 O3

Fig. S125. IR spectrum of **14**

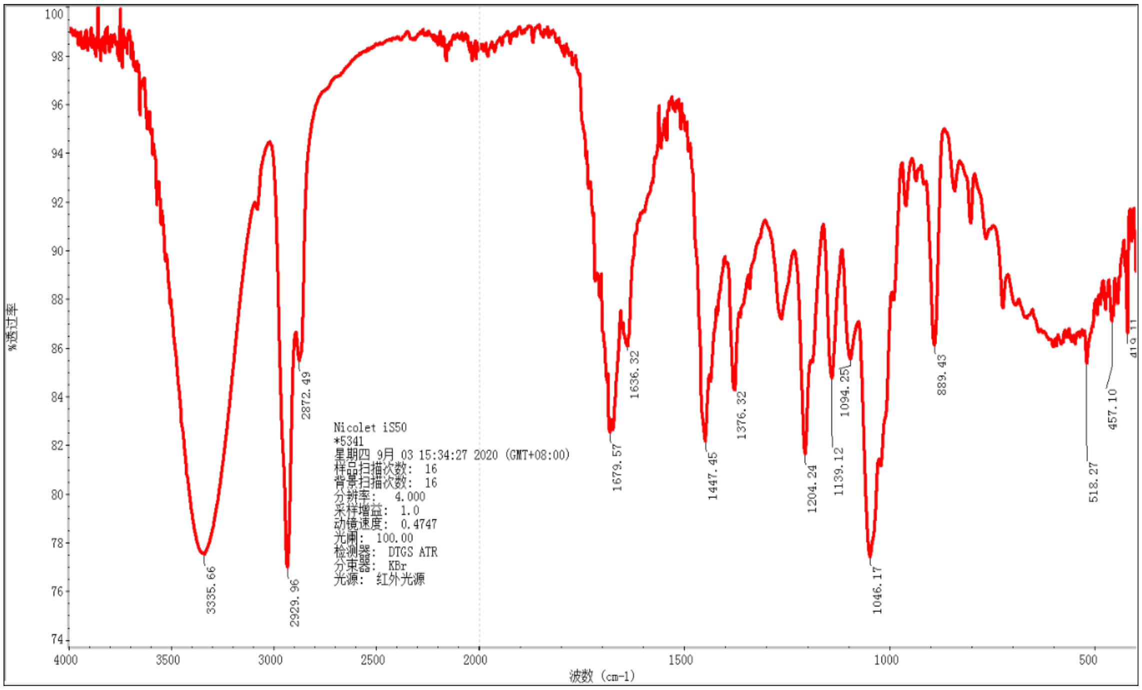

Fig. S126. UV spectrum of **14**

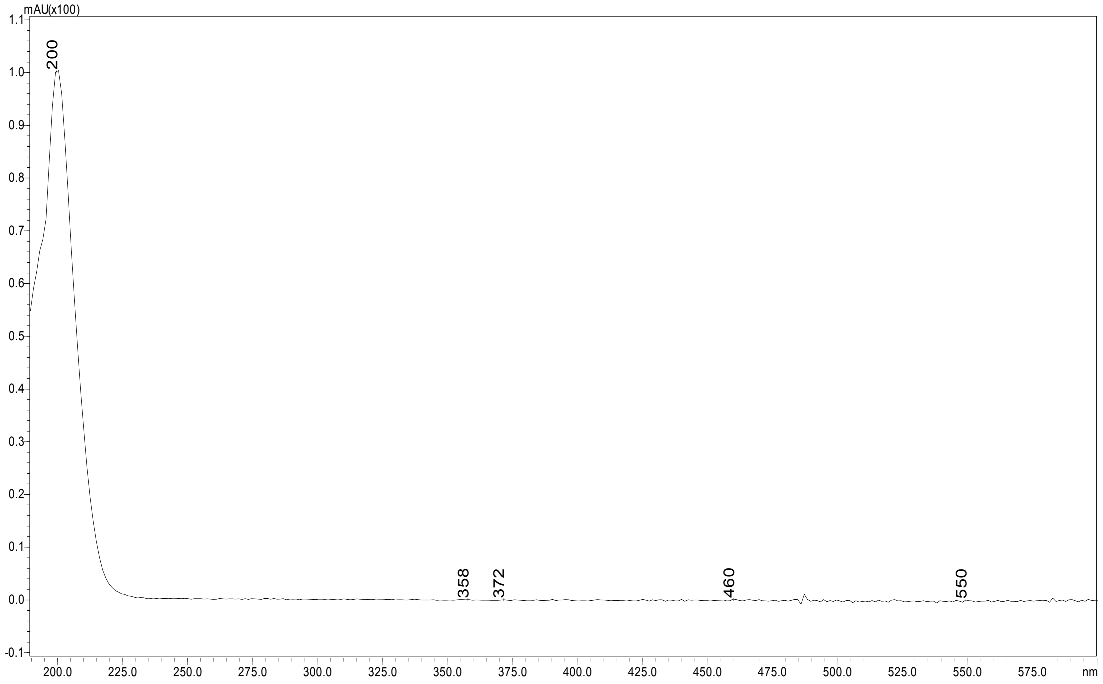

Fig. S127. ^{1}H NMR spectrum of **15** (400 MHz, DMSO-d6)

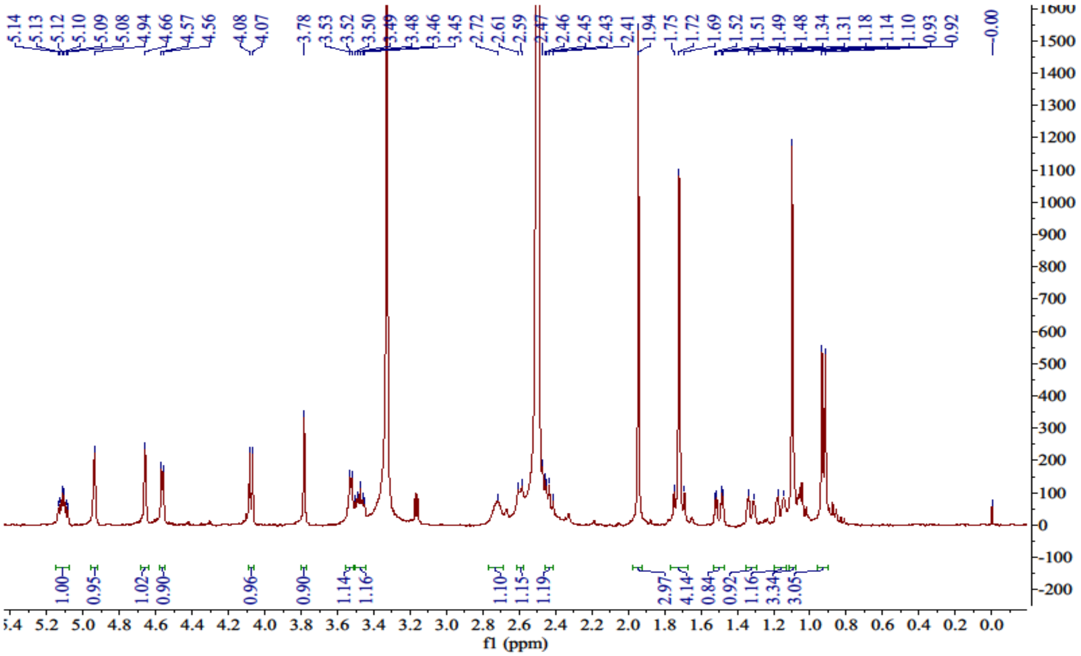

Fig. S128. ^{13}C NMR (APT) spectrum of **15** (100 MHz, DMSO-d6)

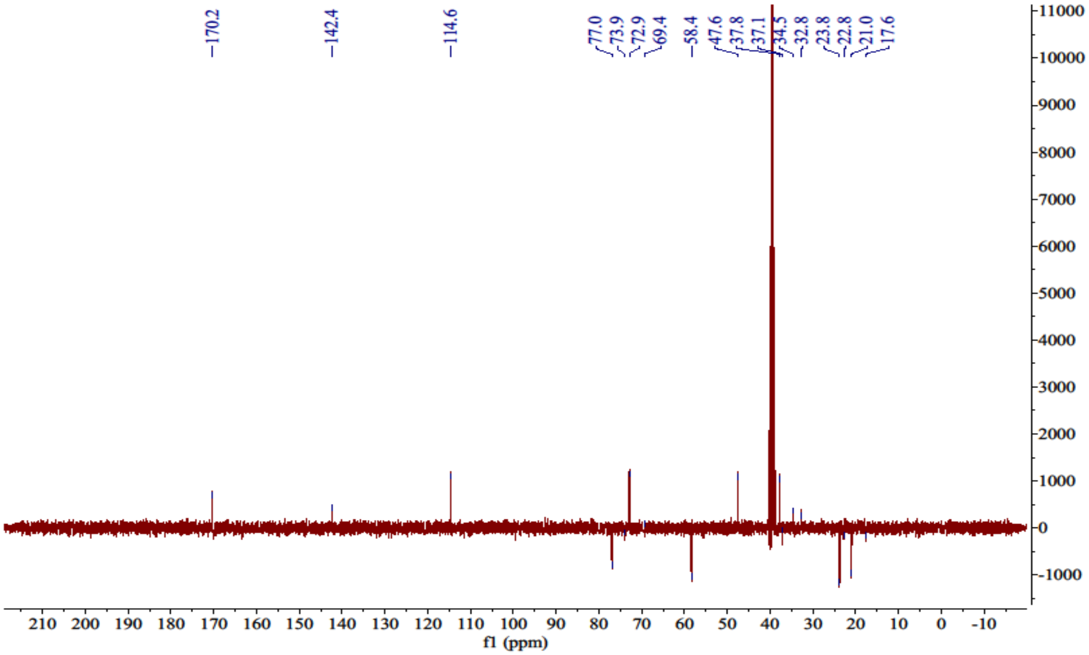

Fig. S129. ^{1}H-^{1}H COSY spectrum of **15**

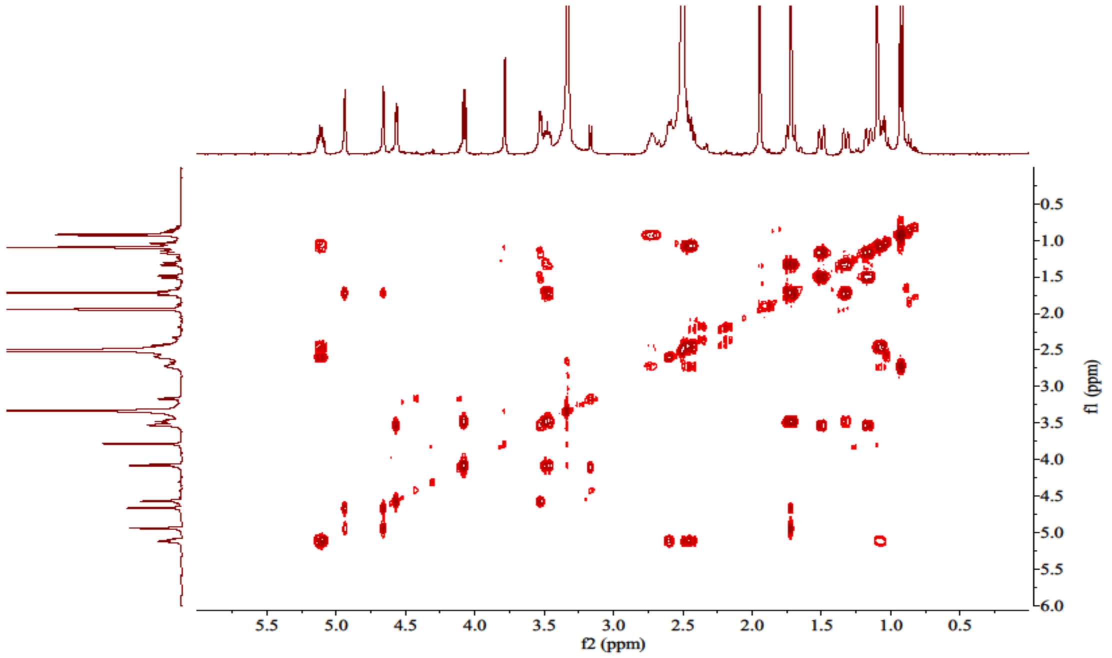

Fig. S130. HSQC spectrum of **15**

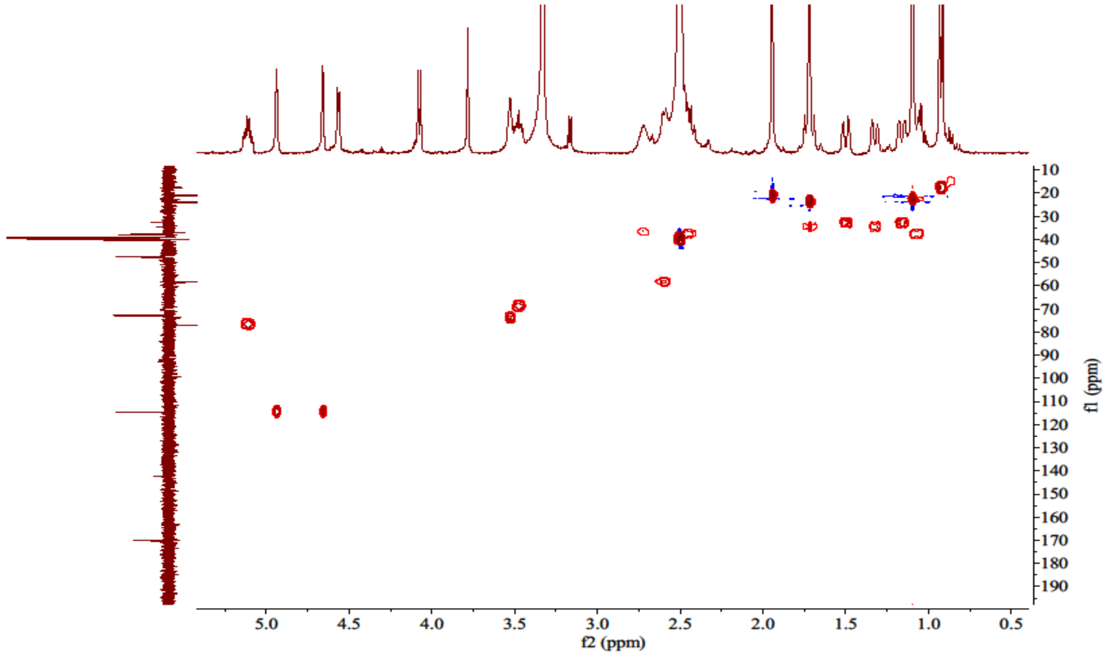

Fig. S131. HMBC spectrum of **15**

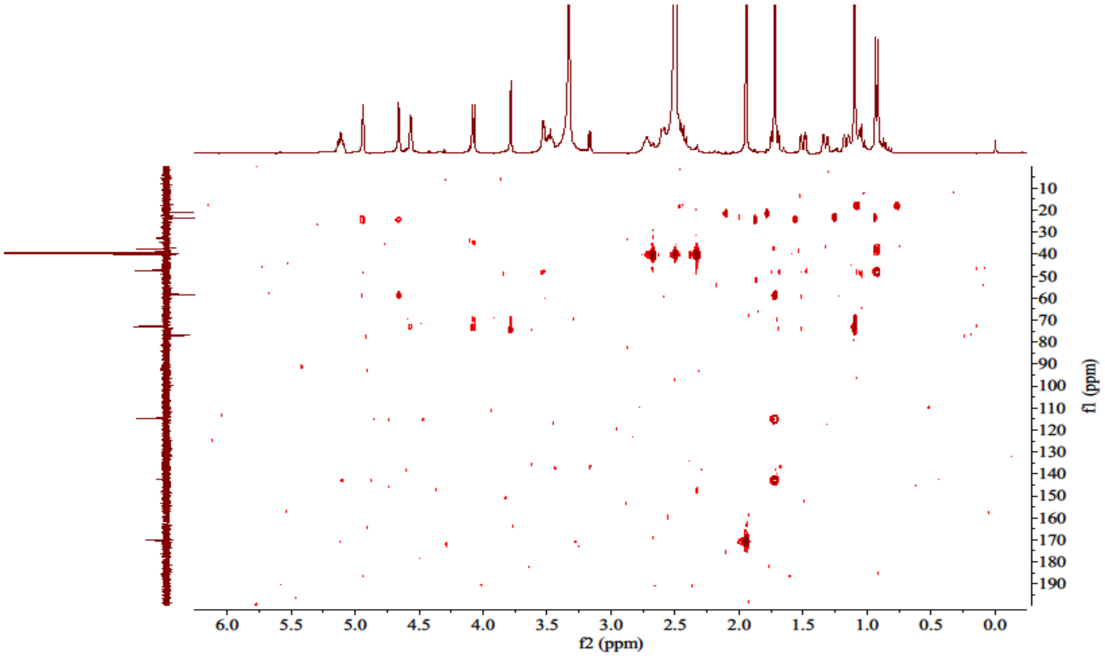

Fig. S132. NOESY spectrum of **15**

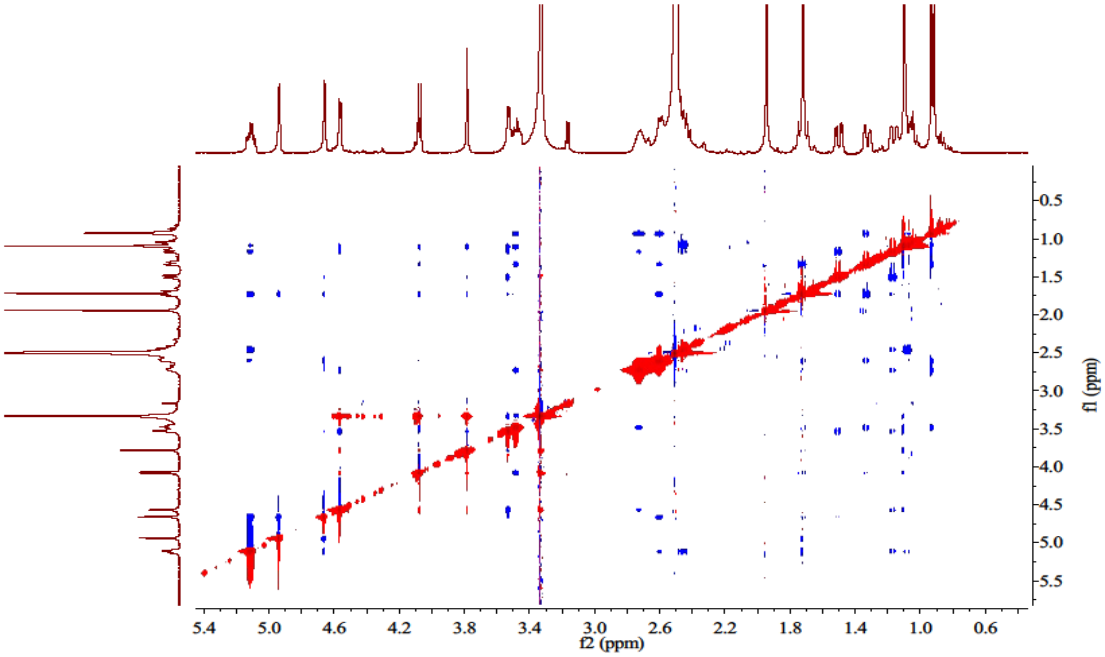

Fig. S133. HRESIMS spectrum of **15**

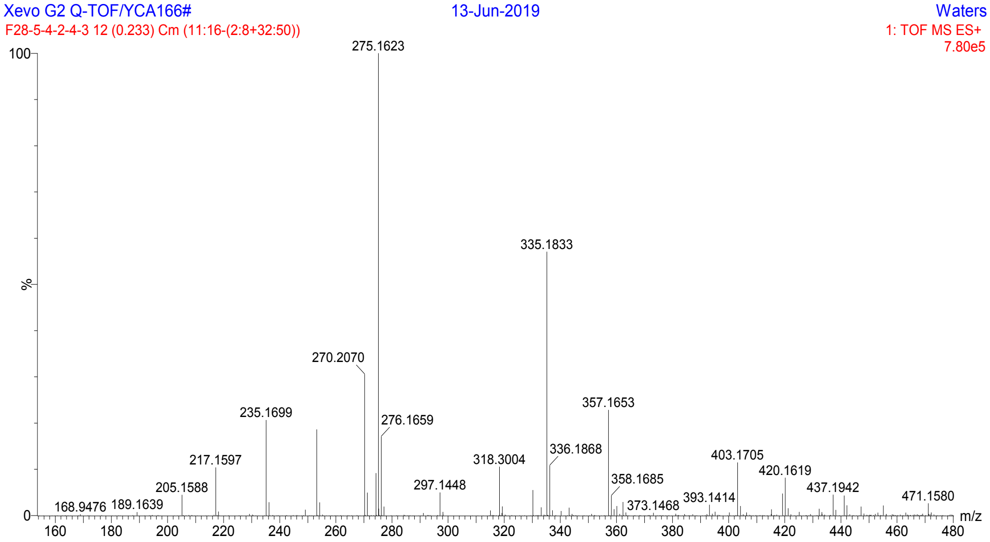

Mass	Calc. Mass	mDa	PPM	DBE	i-FIT	Norm	Conf(%)	Formula
335.1833	335.1834	-0.1	-0.3	3.5	135.3	0.067	93.52	C17 H28 O5 Na

Fig. S134. IR spectrum of **15**

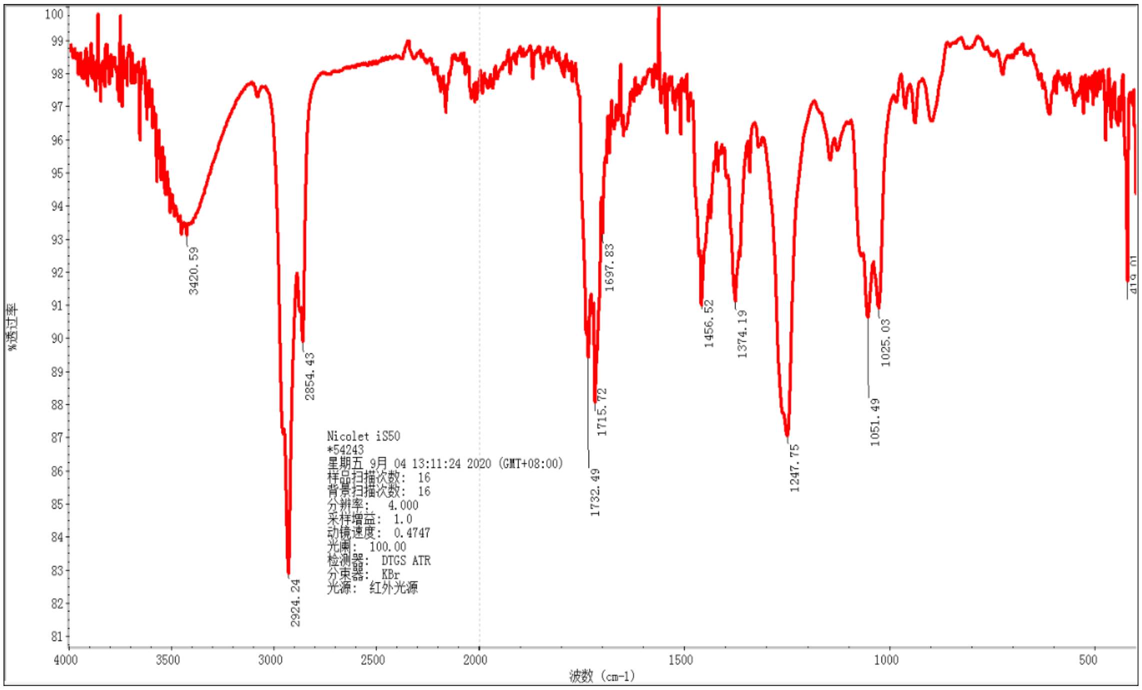

Fig. S135. UV spectrum of **15**

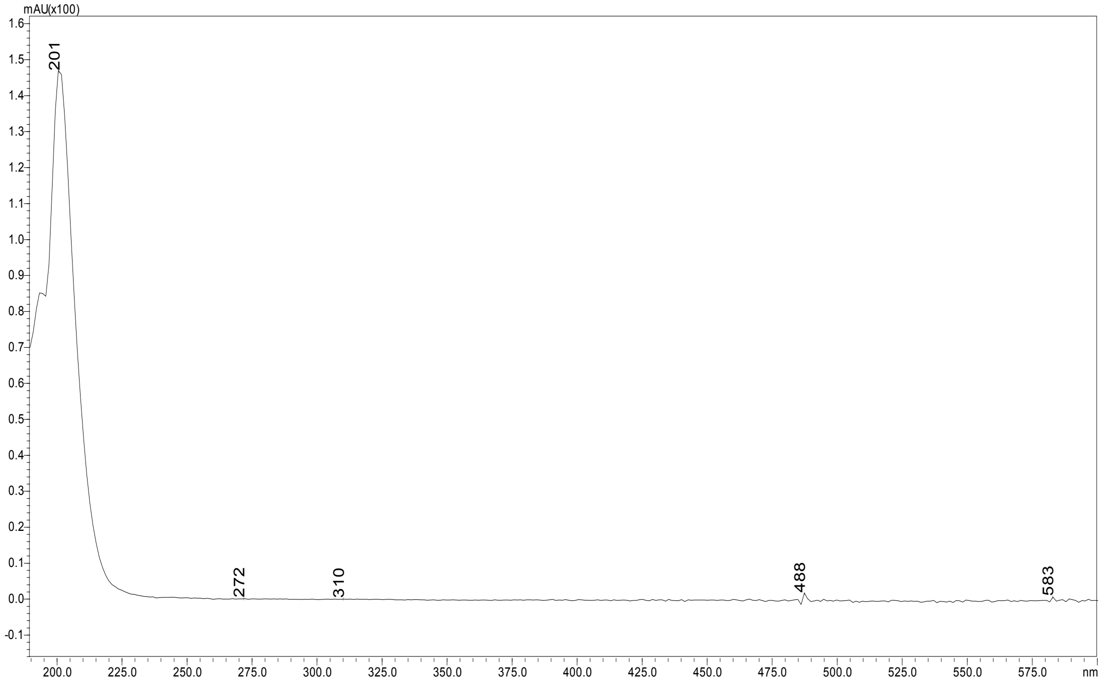

Fig. S136. ^{1}H NMR spectrum of **16** (400 MHz, DMSO-d6)

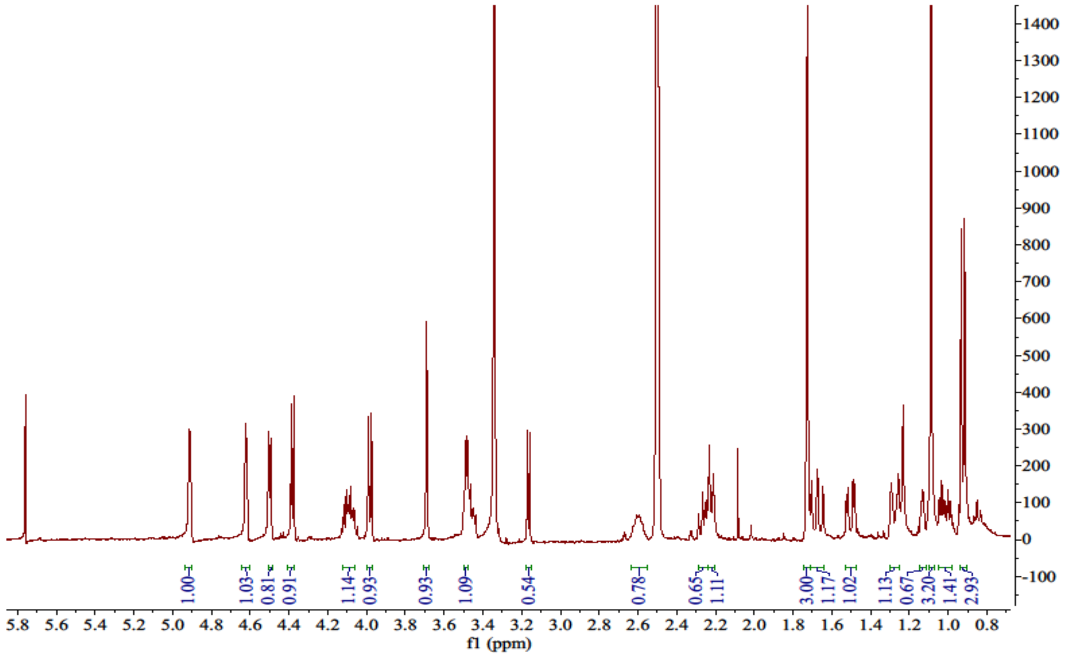

Fig. S137. ^{13}C NMR (APT) spectrum of **16** (100 MHz, DMSO-d6)

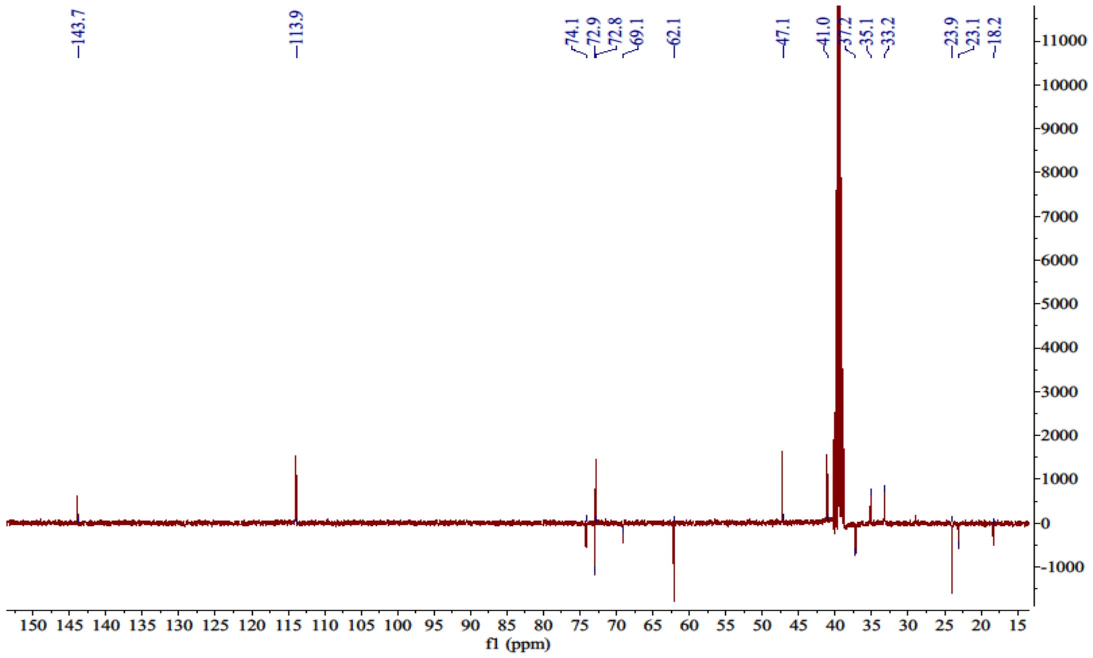

Fig. S138. ^{1}H-^{1}H COSY spectrum of **16**

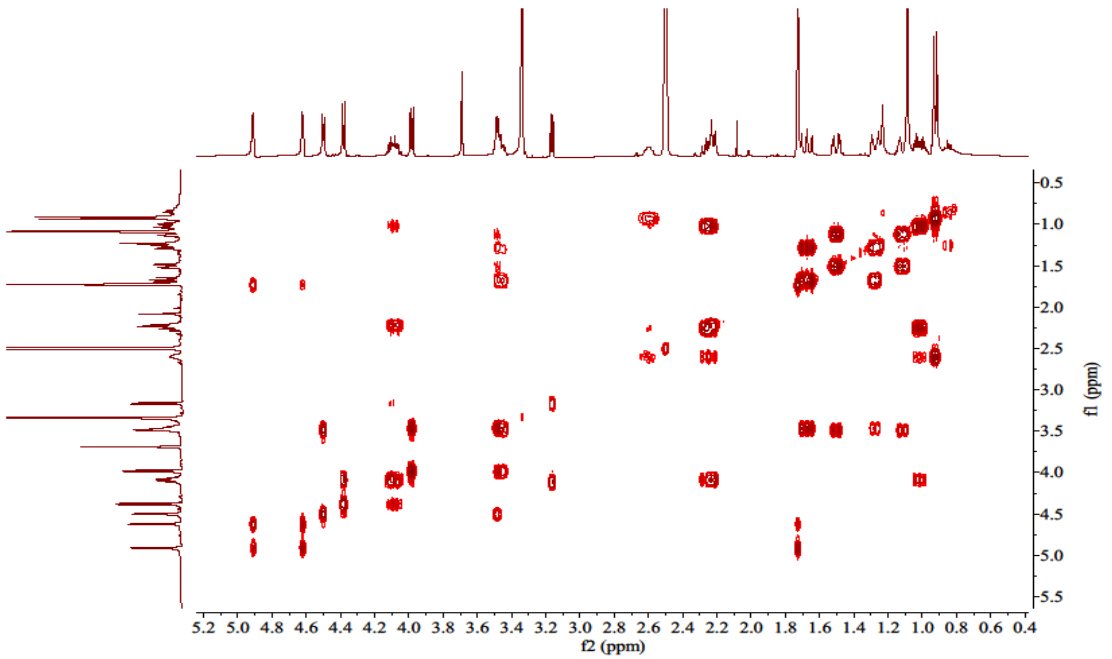

Fig. S139. HSQC spectrum of **16**

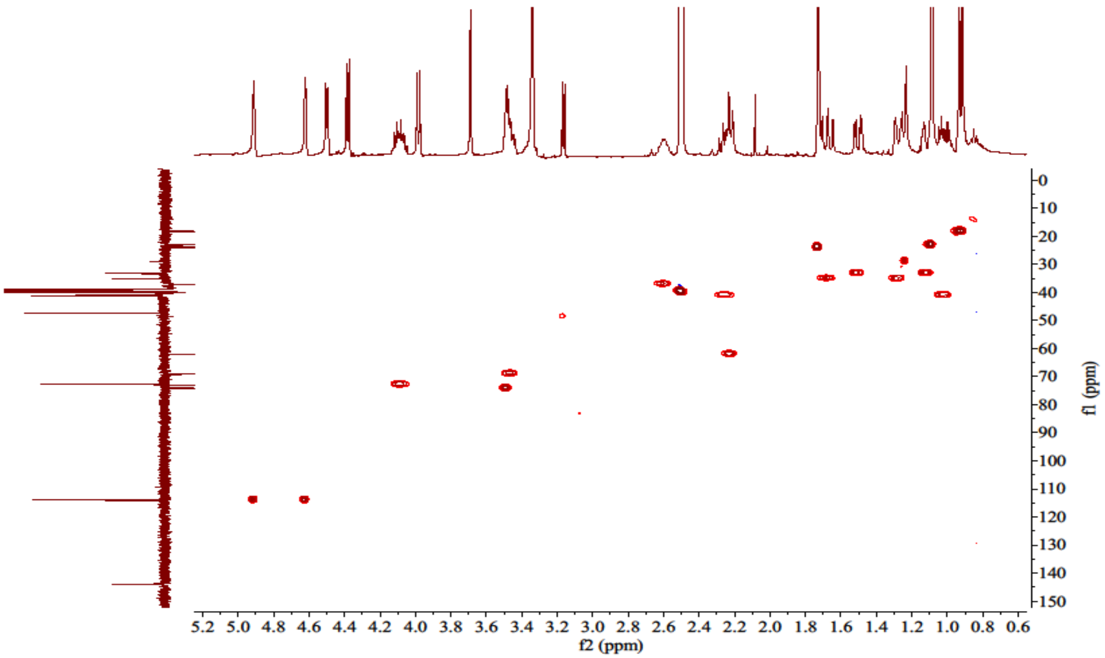

Fig. S140. HMBC spectrum of **16**

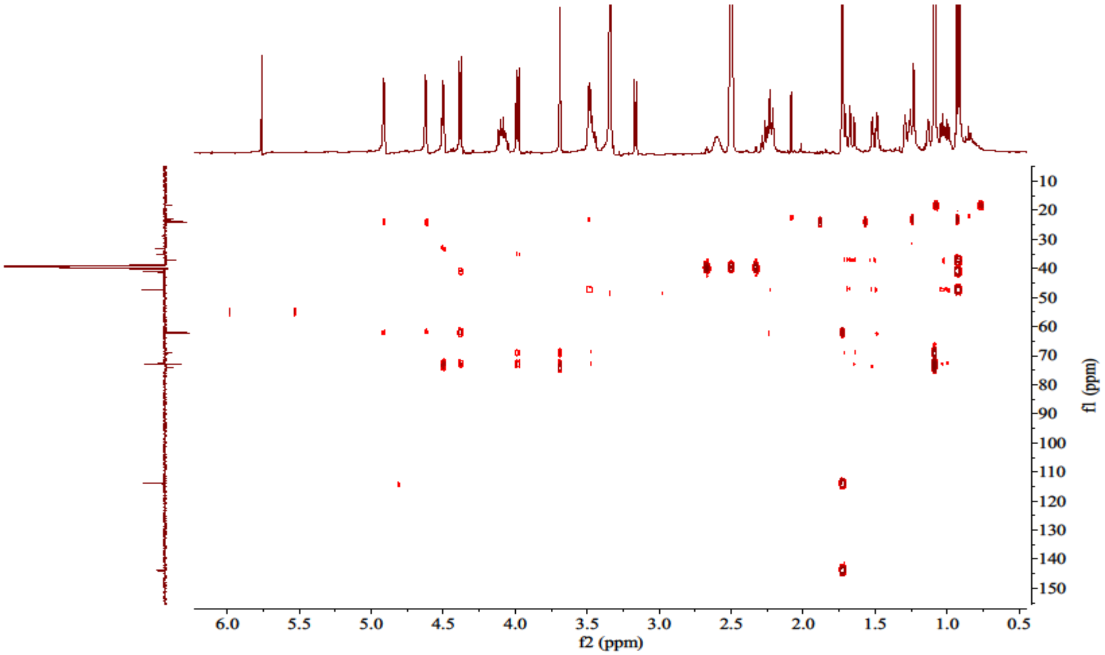

Fig. S141. NOESY spectrum of **16**

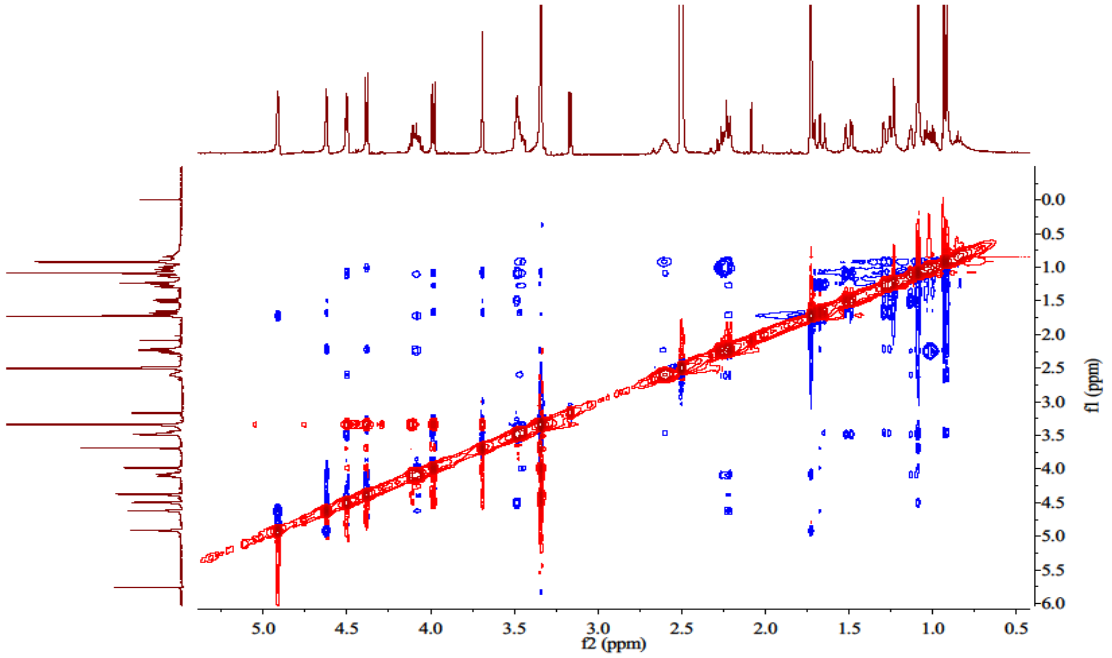

Fig. S142. HRESIMS spectrum of **16**

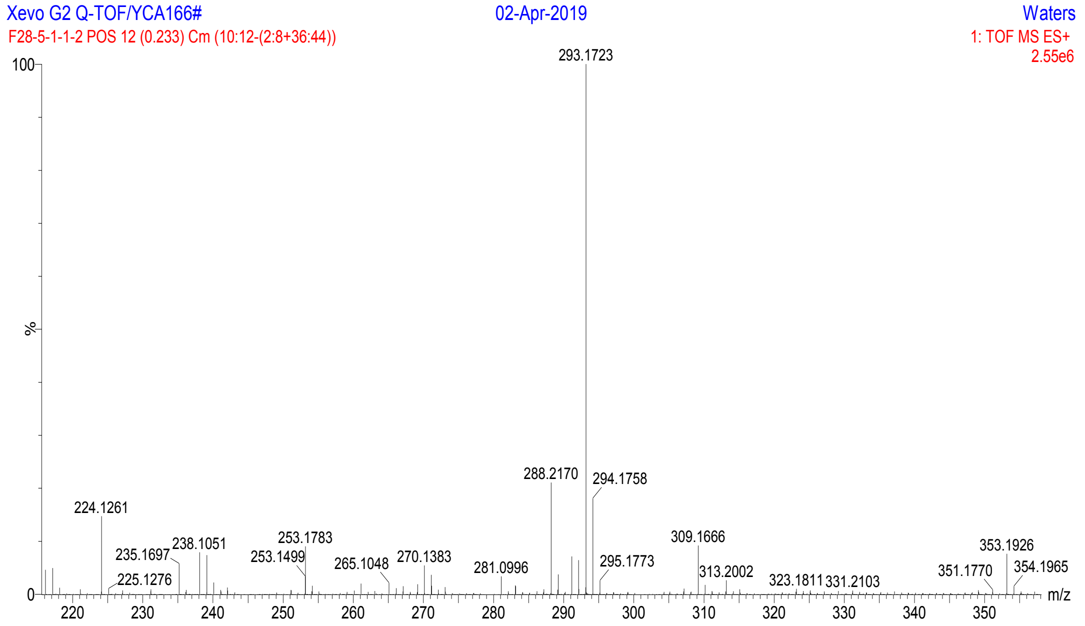

Mass	Calc. Mass	mDa	PPM	DBE	i-FIT	Norm	Conf(%)	Formula
293.1723	293.1729	-0.6	-2.0	2.5	441.4	n/a	n/a	C15 H26 O4 Na

Fig. S143. IR spectrum of **16**

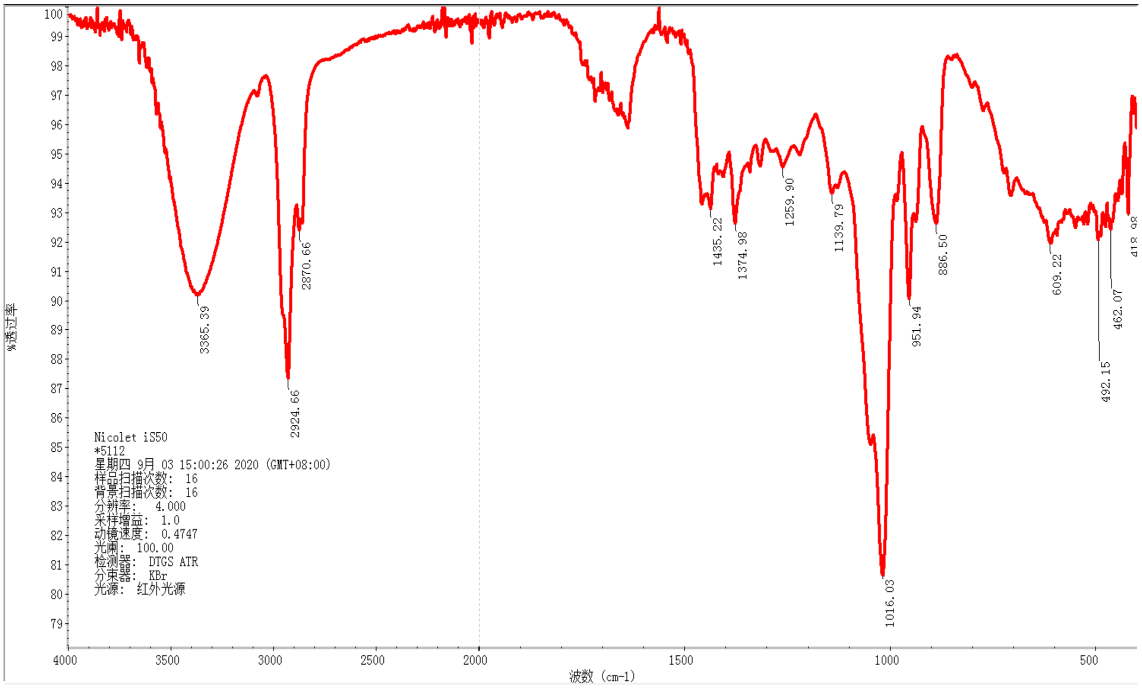

Fig. S144. UV spectrum of **16**

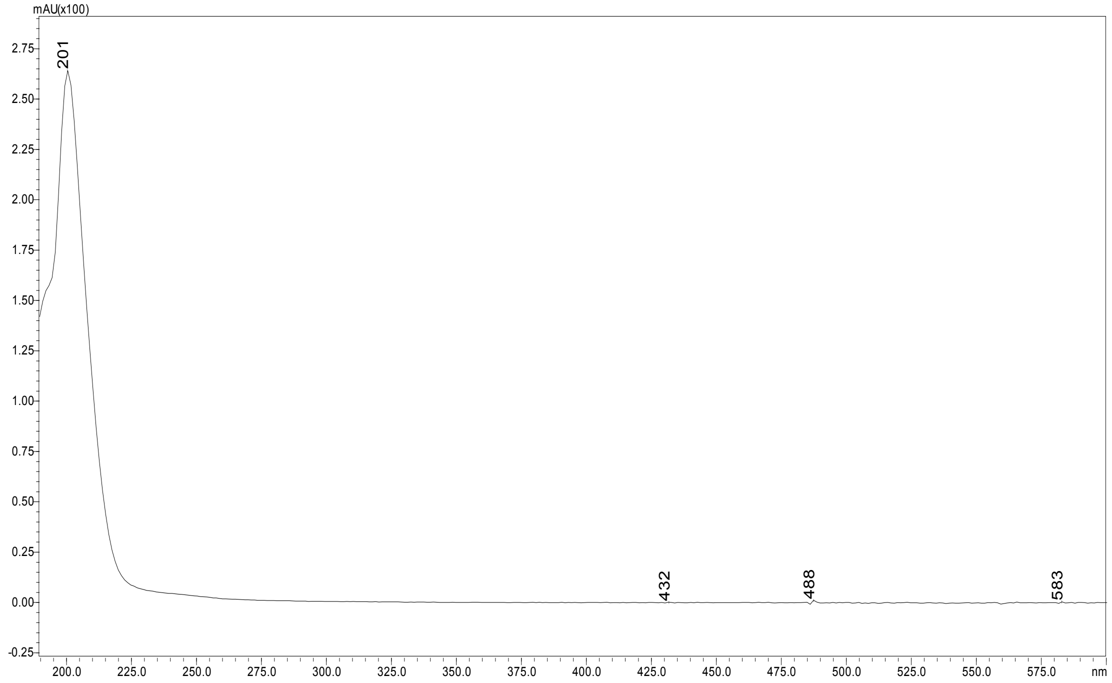

Fig. S145. ^{1}H NMR spectrum of **17** (400 MHz, DMSO-d6)

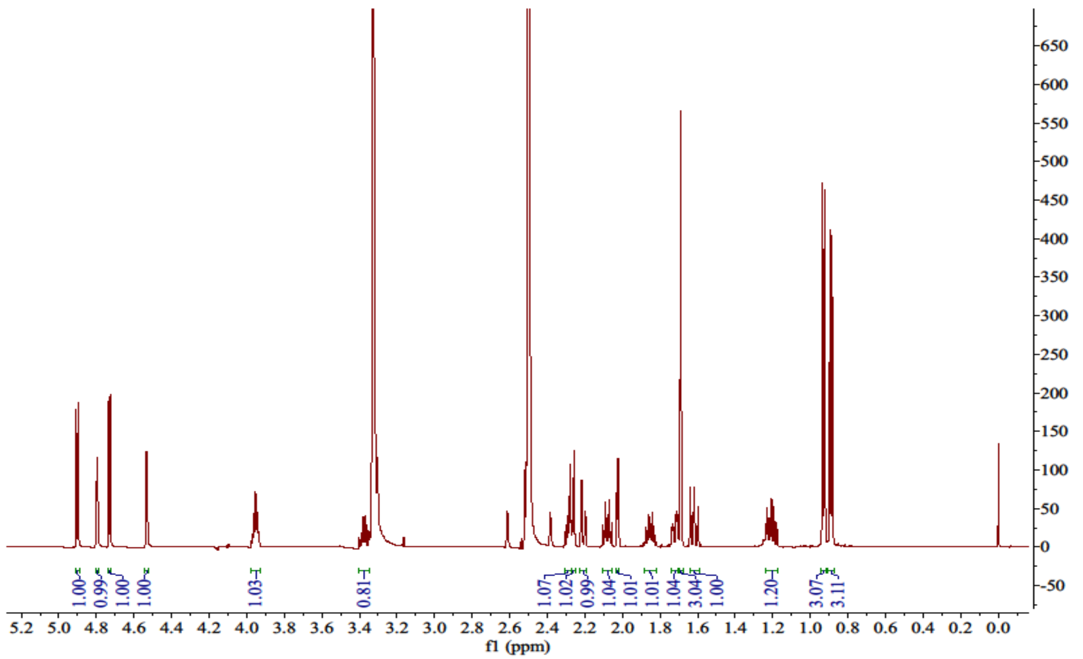

Fig. S146. ^{13}C NMR (APT) spectrum of **17** (100 MHz, DMSO-d6)

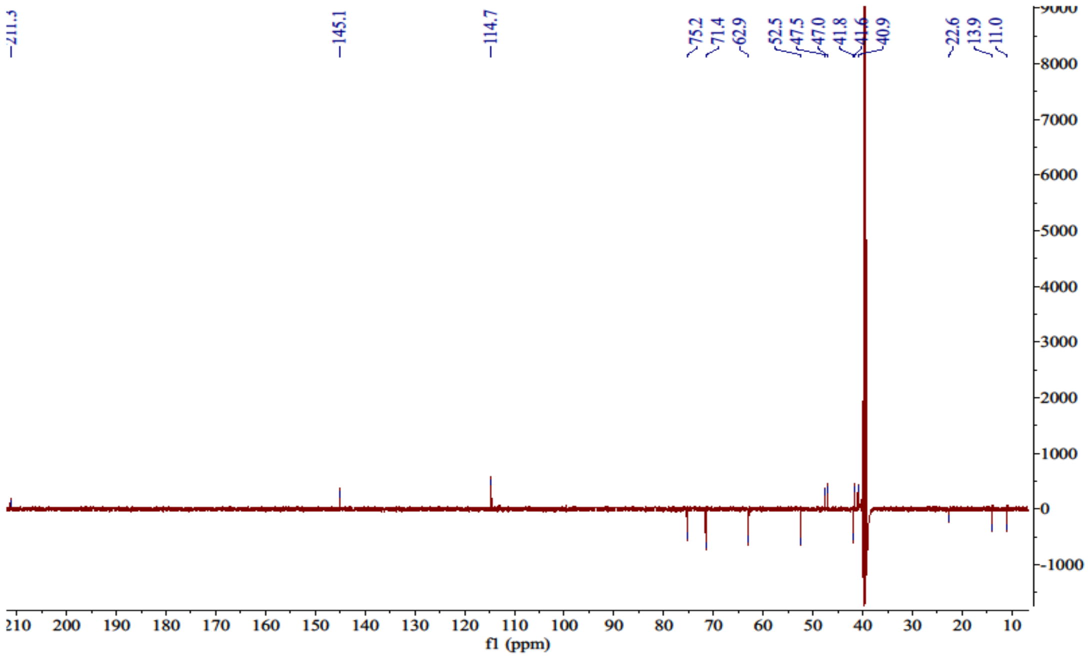

Fig. S147. ^{1}H-^{1}H COSY spectrum of **17**

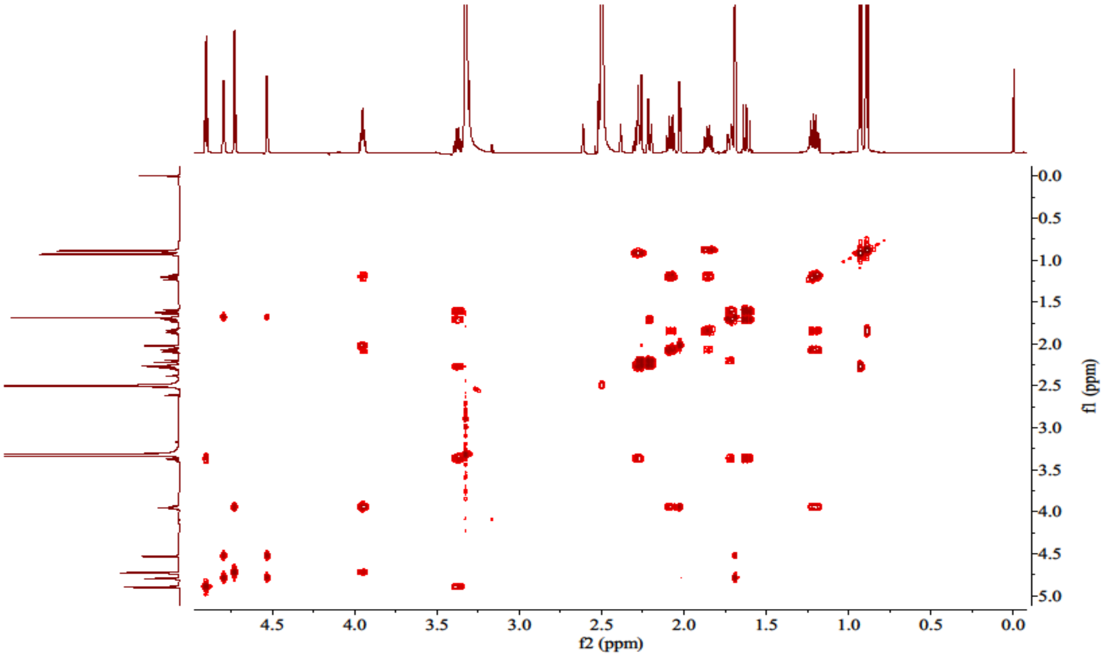

Fig. S148. HSQC spectrum of **17**

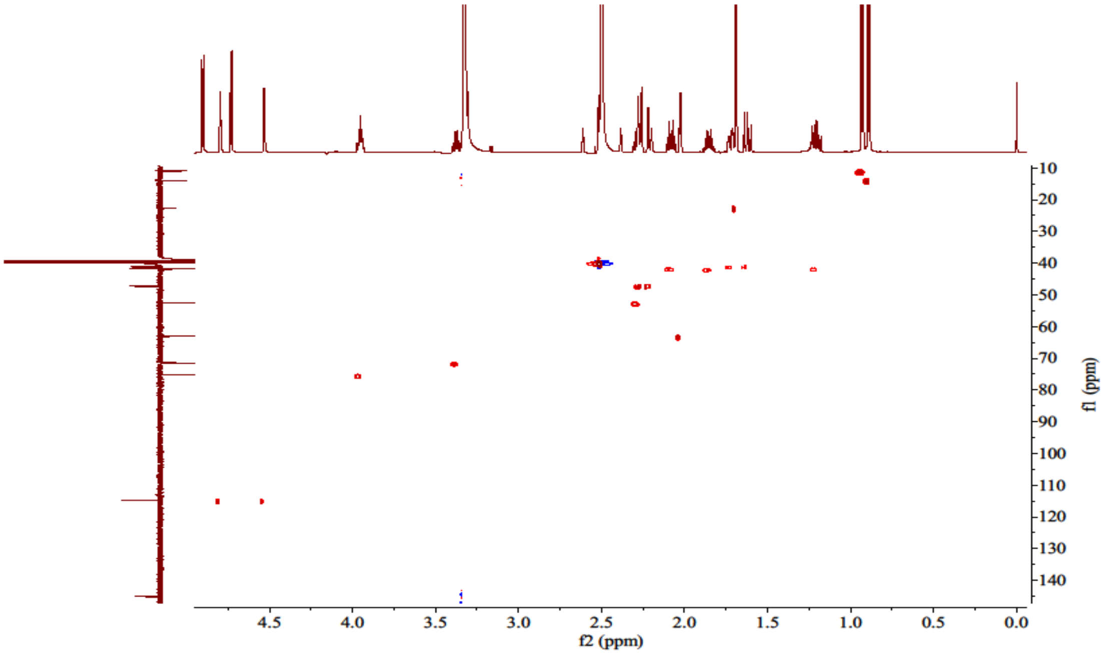

Fig. S149. HMBC spectrum of **17**

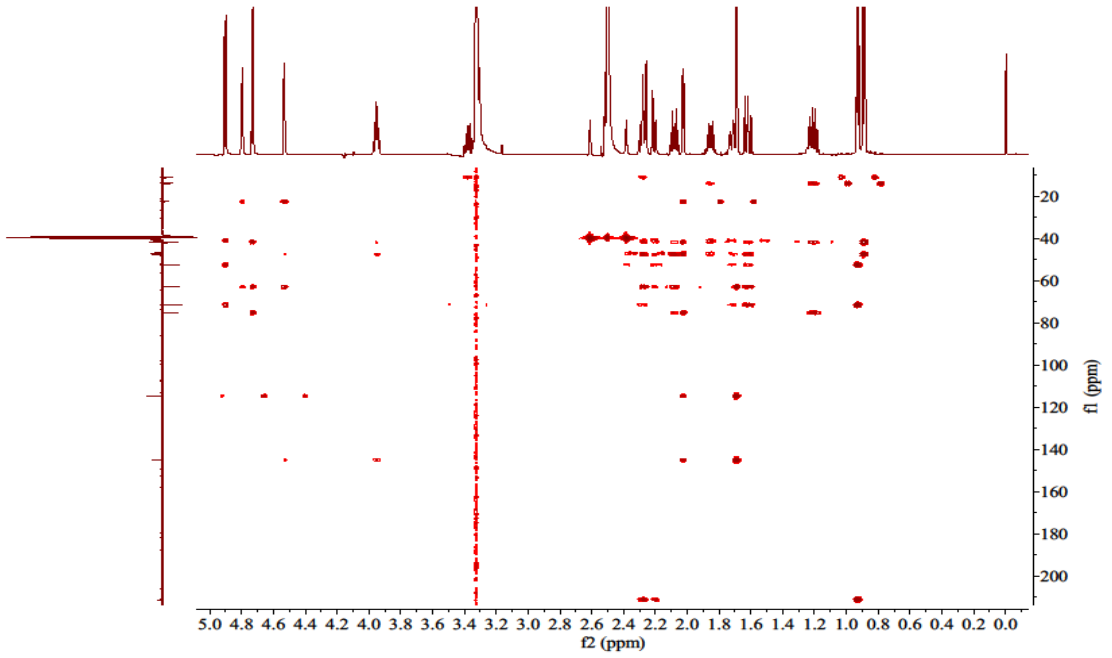

Fig. S150. NOESY spectrum of **17**

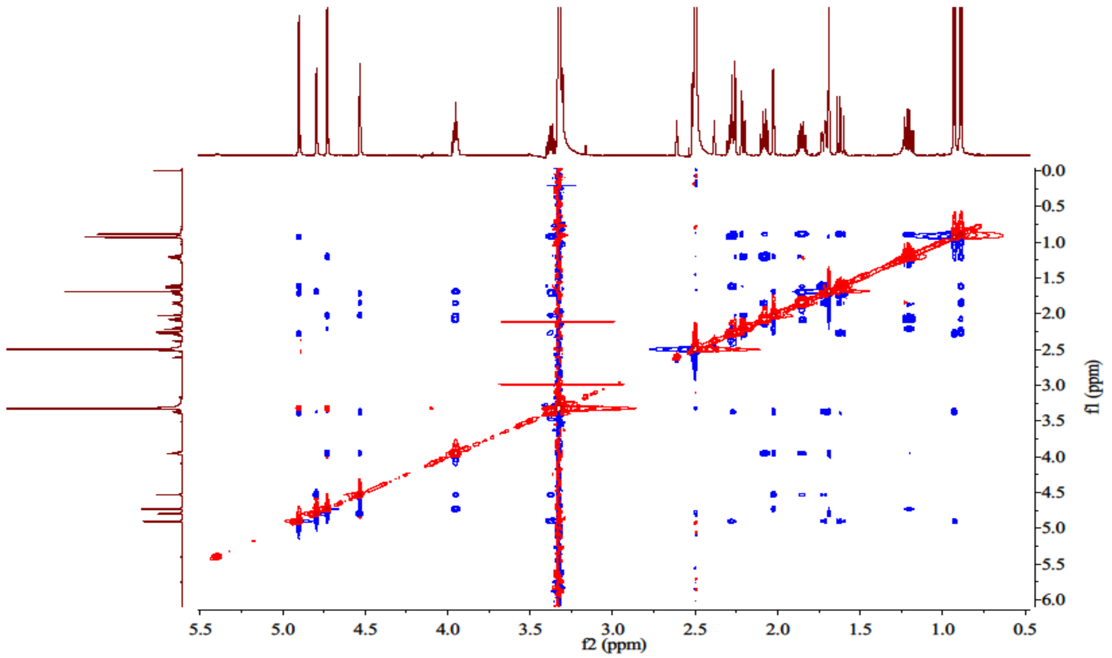

Fig. S151. HRESIMS spectrum of **17**

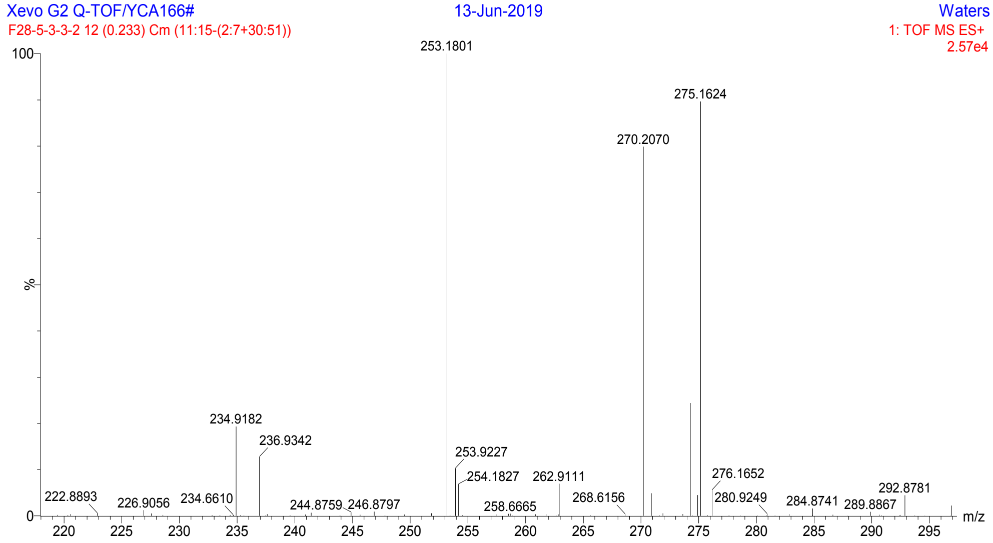

Mass	Calc. Mass	mDa	PPM	DBE	i-FIT	Norm	Conf(%)	Formula
253.1801	253.1804	-0.3	-1.2	3.5	61.1	n/a	n/a	C15 H25 O3

Fig. S152. IR spectrum of **17**

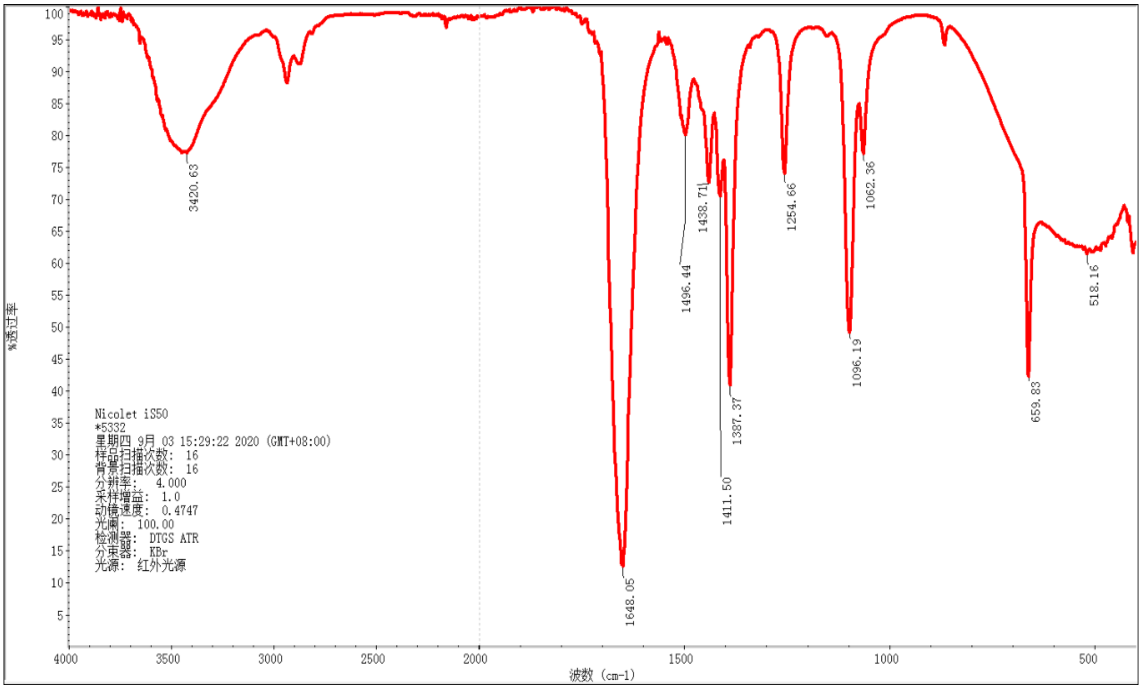

Fig. S153. UV spectrum of **17**

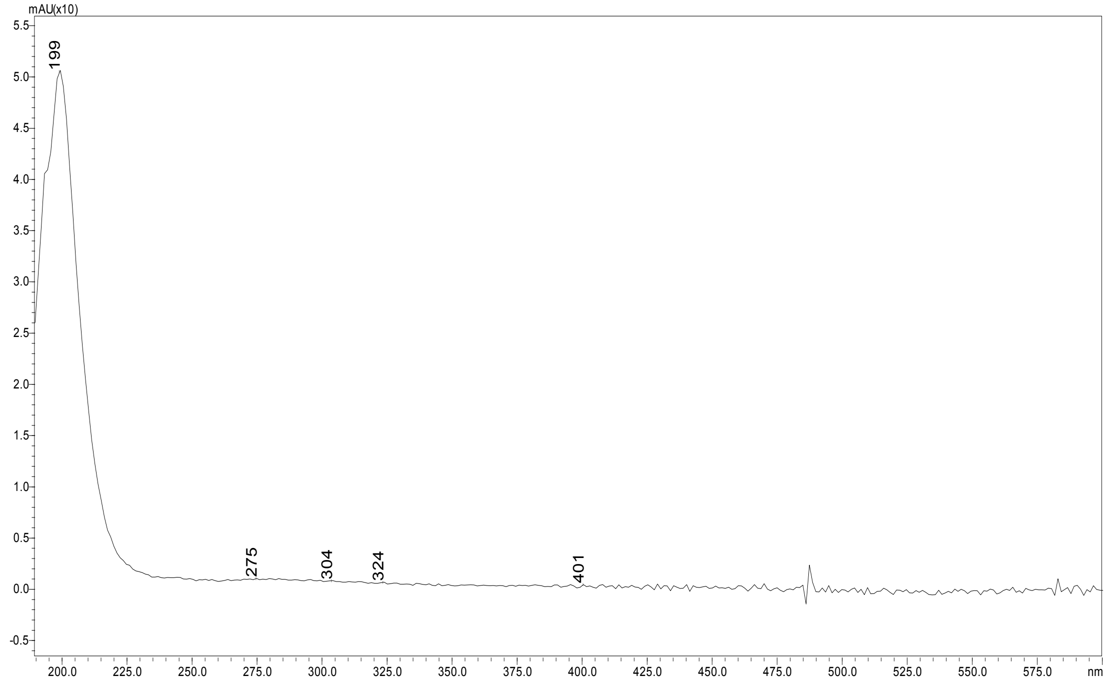

Fig. S154. ^{1}H NMR spectrum of **18** (400 MHz, DMSO-d6)

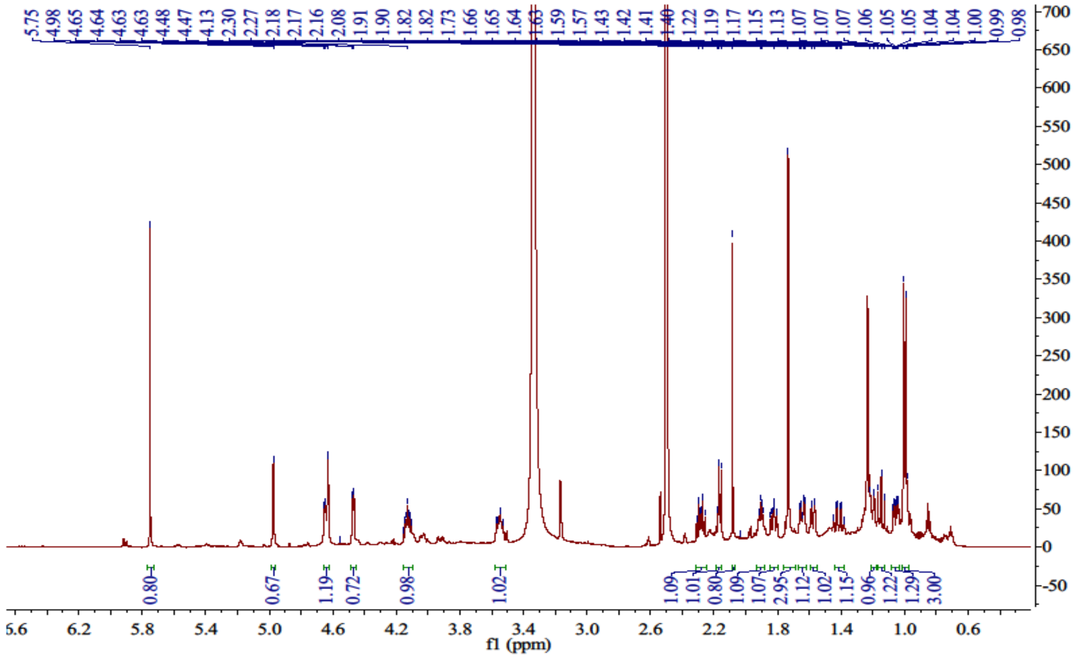

Fig. S155. ^{13}C NMR (APT) spectrum of **18** (100 MHz, DMSO-d6)

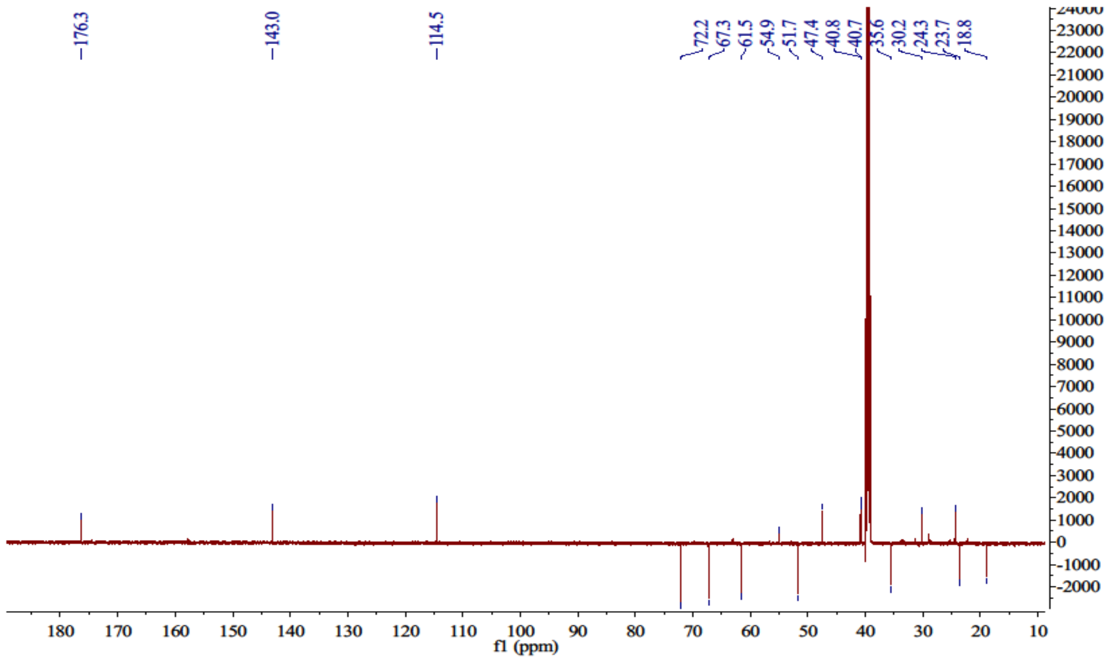

Fig. S156. ^1H-^1H COSY spectrum of **18**

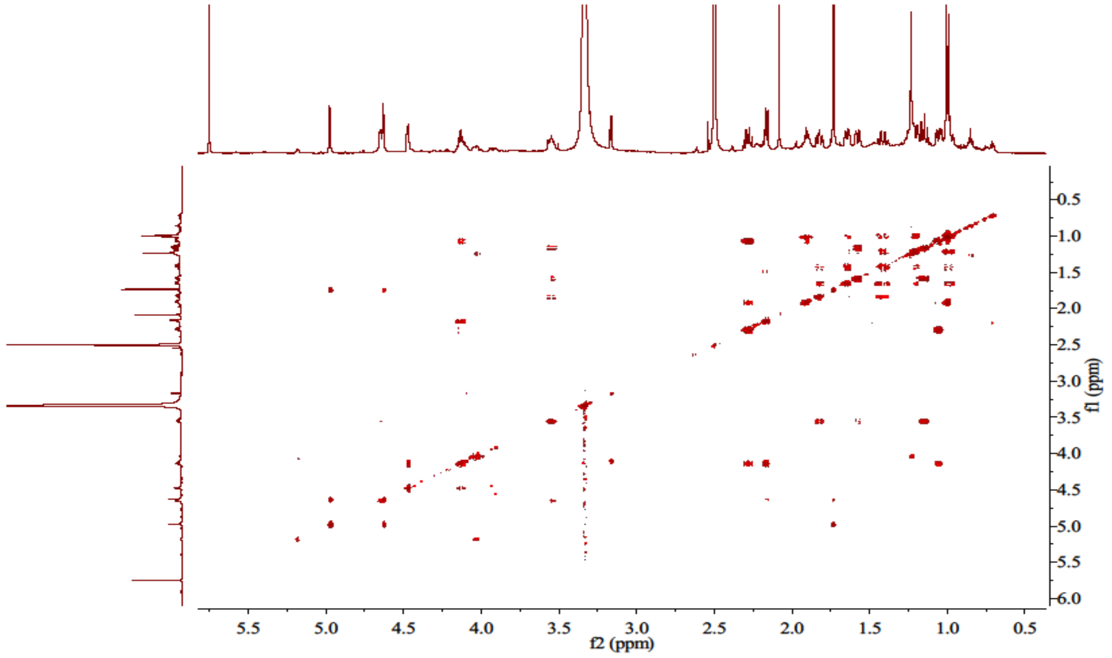

Fig. S157. HSQC spectrum of **18**

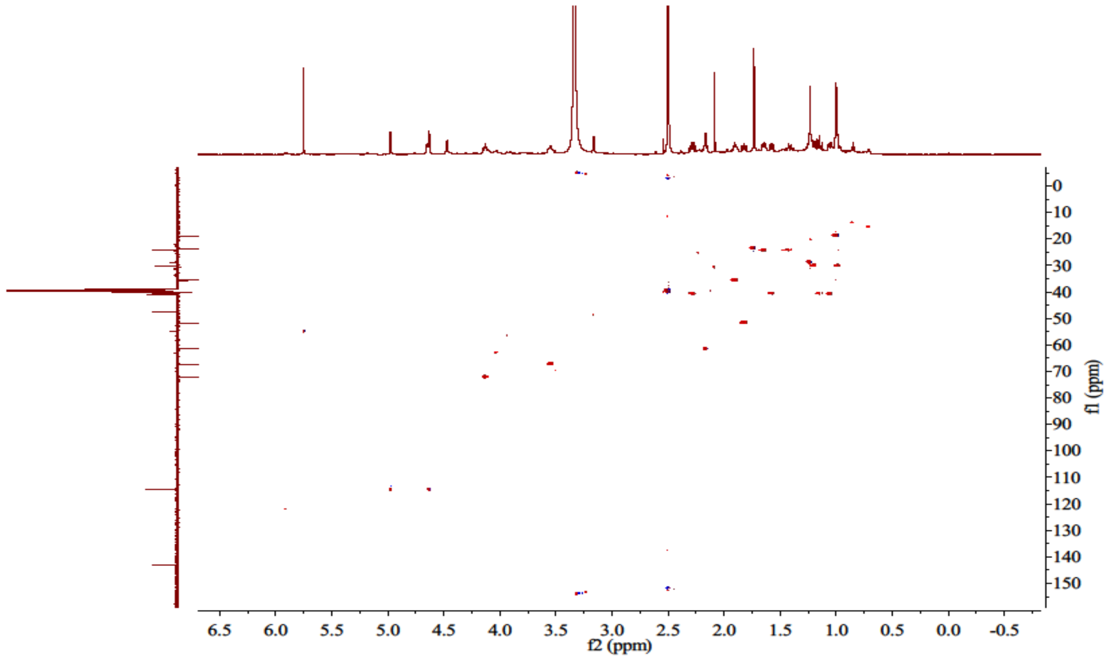

Fig. S158. HMBC spectrum of **18**

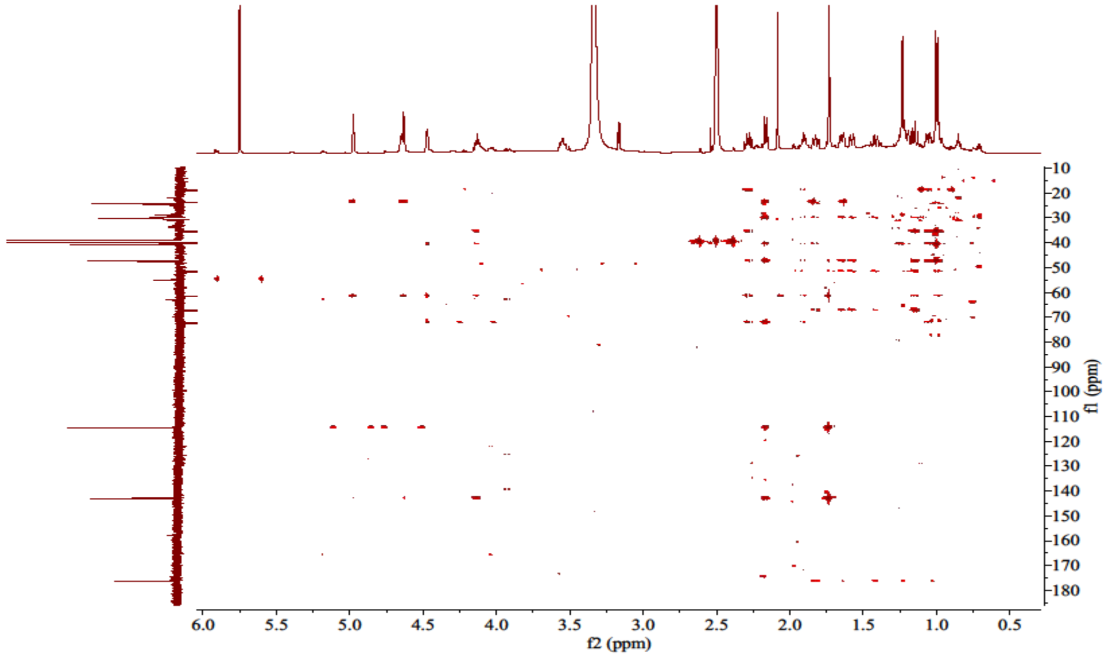

Fig. S159. NOESY spectrum of **18**

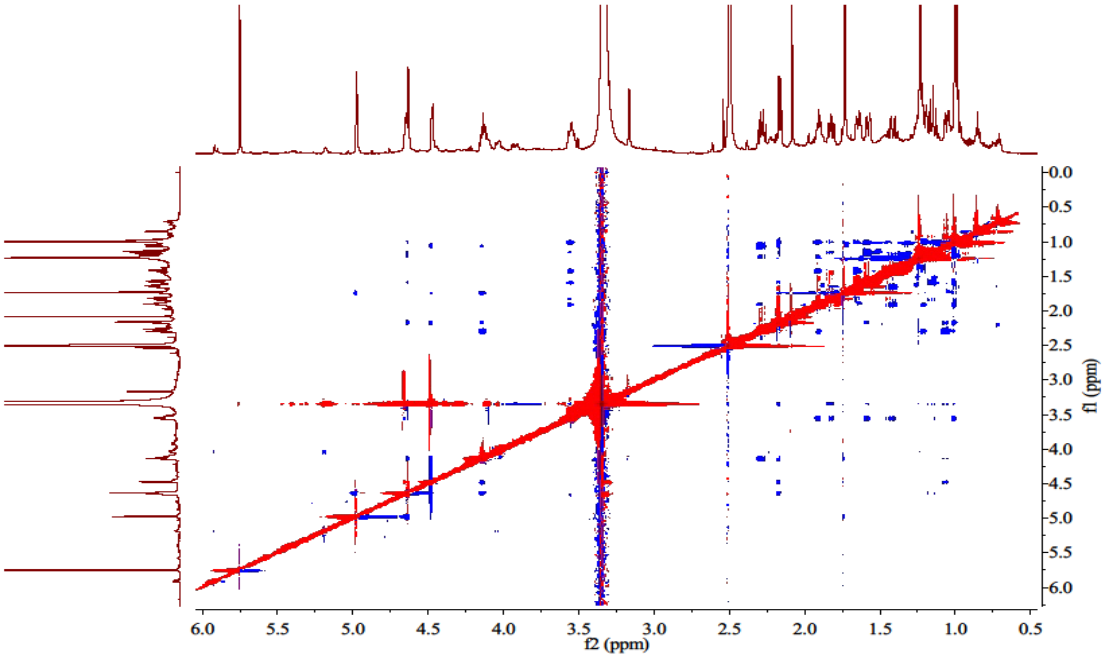

Fig. S160. HRESIMS spectrum of **18**

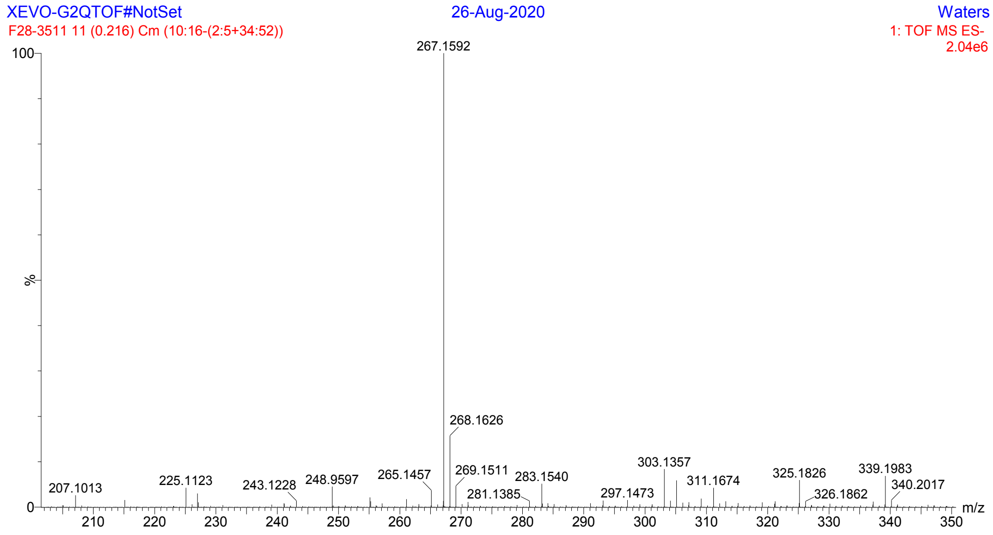

Mass	Calc. Mass	mDa	PPM	DBE	i-FIT	Norm	Conf(%)	Formula
267.1592	267.1596	-0.4	-1.5	4.5	544.2	n/a	n/a	C15 H23 O4

Fig. S161. IR spectrum of **18**

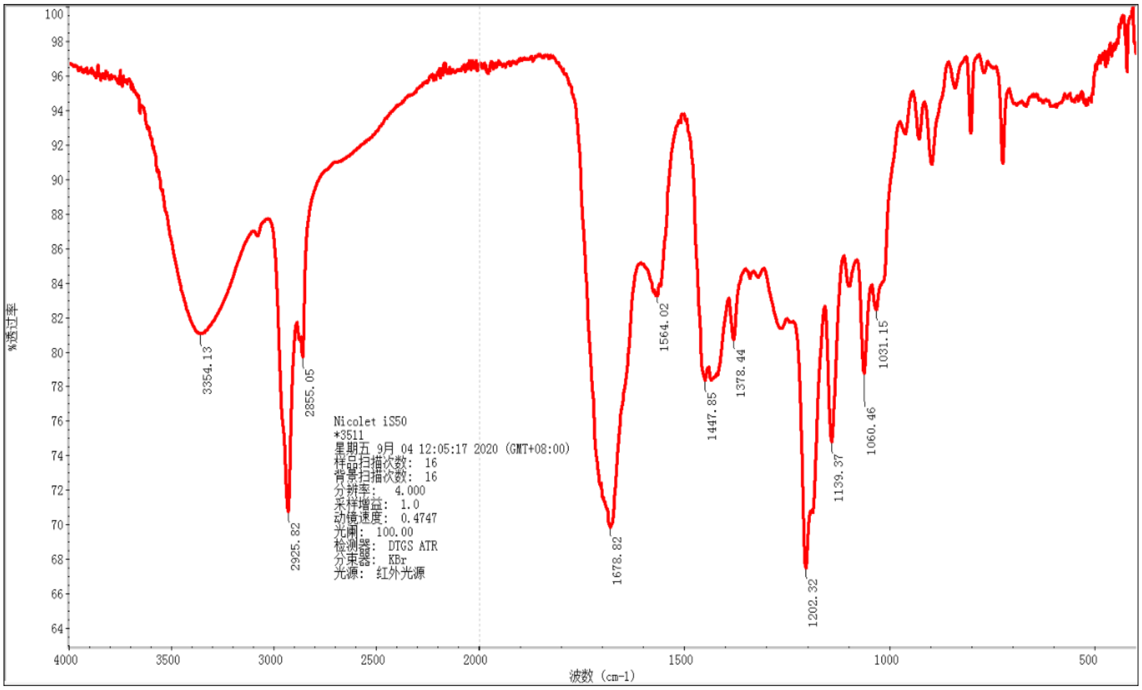

Fig. S162. UV spectrum of **18**

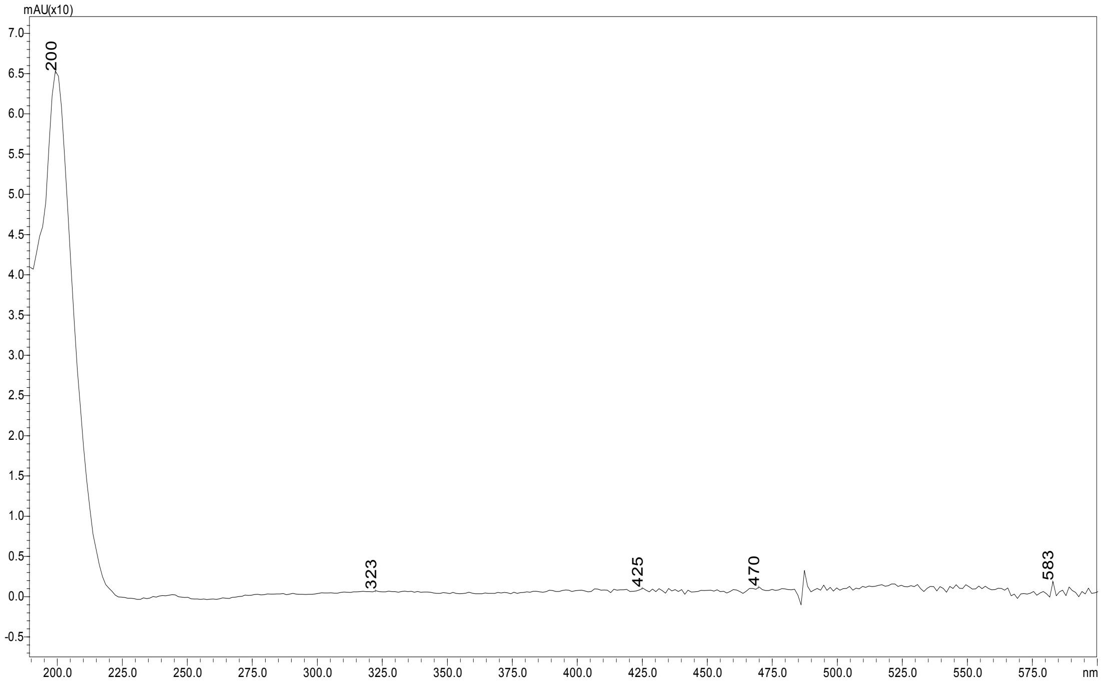

Fig. S163. Experimental and calculated ECD spectra of **3-18**

Low-energy conformers of (1*R*, 2*R*, 4*S*, 5*S*, 9*S*, 11*R*)-**3** obtained at the ωB97X/TZVP PCM/MeOH level of theory

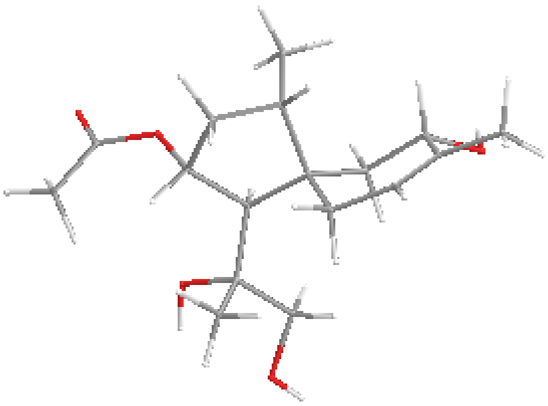

Conf. A 0.07%

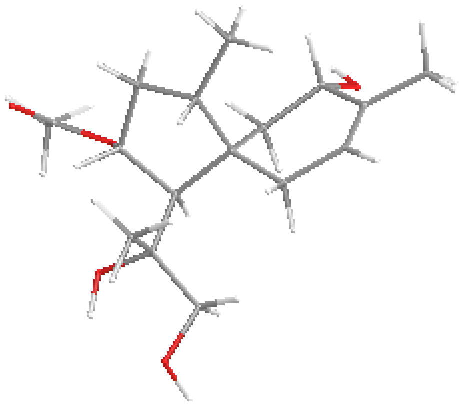

Conf. B 99.93%

conformers	Energy	Percentage (%)
Conf. A	-1041.0881233	0.07
Conf. B	1041.0950205	99.93

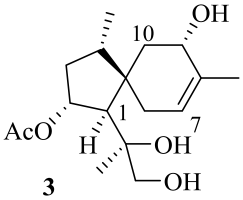

3

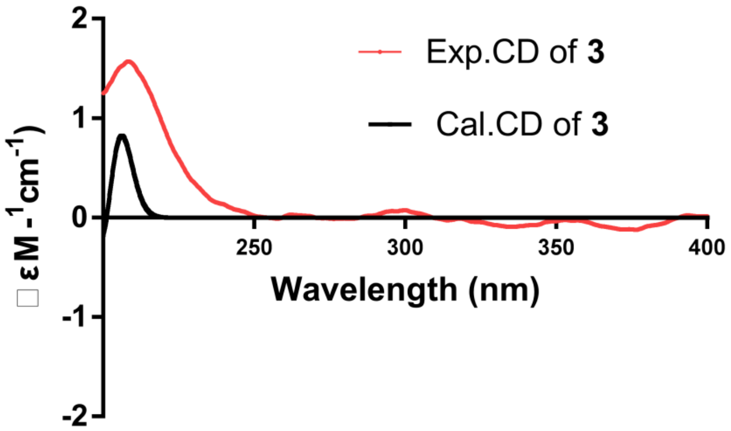

Low-energy conformers of (1*R*, 2*R*, 4*S*, 5*S*, 9*S*, 11*S*)-**4** obtained at the ωB97X/TZVP PCM/MeOH level of theory

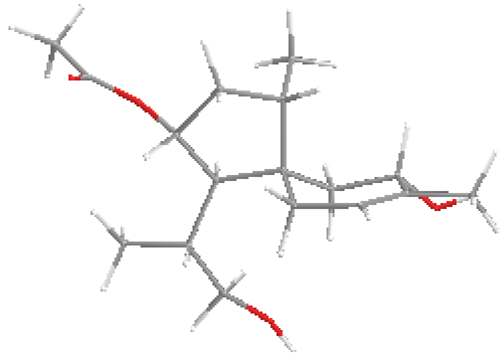

Conf.A 100%

Conformers	Energy	Percentage %
Conf.A	-965.8543358	100

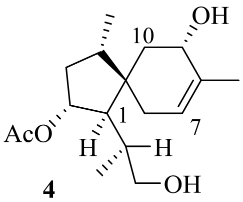

4

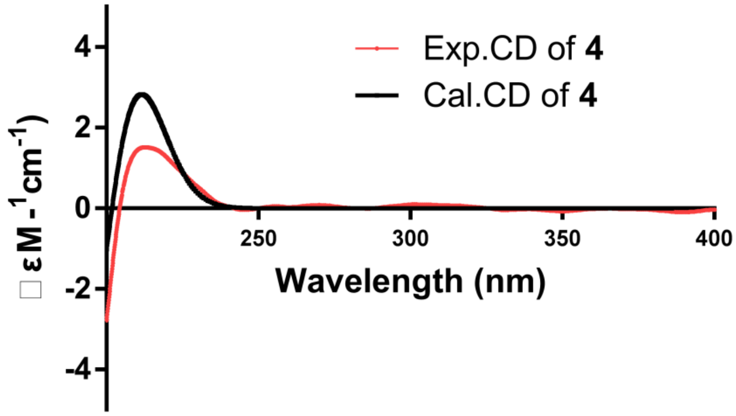

Low-energy conformers of (1*R*, 2*R*, 4*S*, 5*R*, 7*R*)-**6** obtained at the ωB97X/TZVP PCM/MeOH level of theory

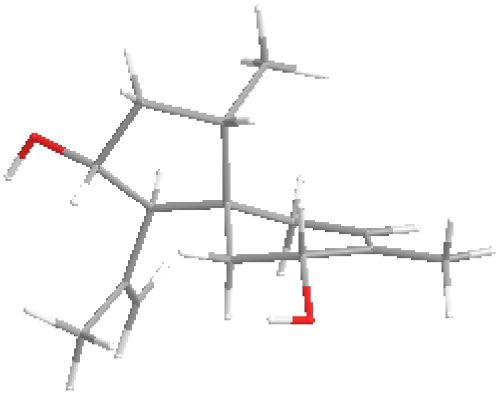

Conf.A100%

conformers	energy	percentage %
Conf.A	-736.6765253	100

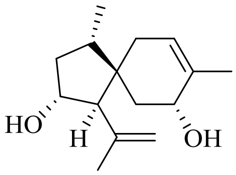

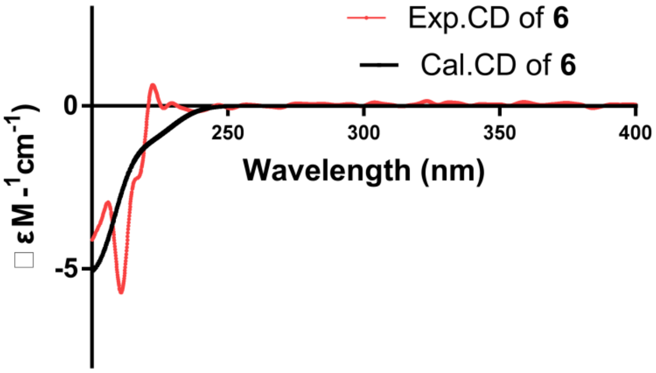

Low-energy conformers of (1*R*, 2*R*, 4*S*, 5*S*, 9*S*)-**5** obtained at the ωB97X/TZVP PCM/MeOH level of theory

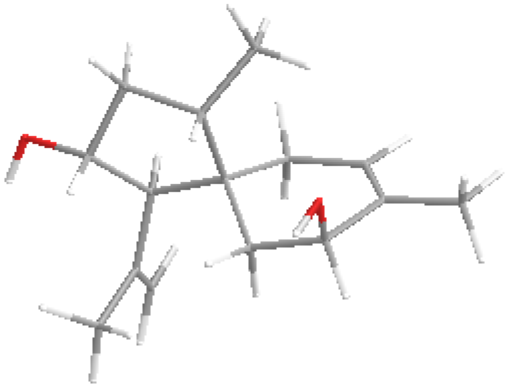

Conf. A 100%

conformers	energy	percentage %
Conf.A	-736.6768458	100

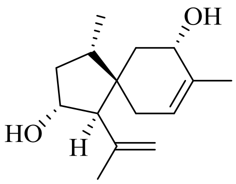

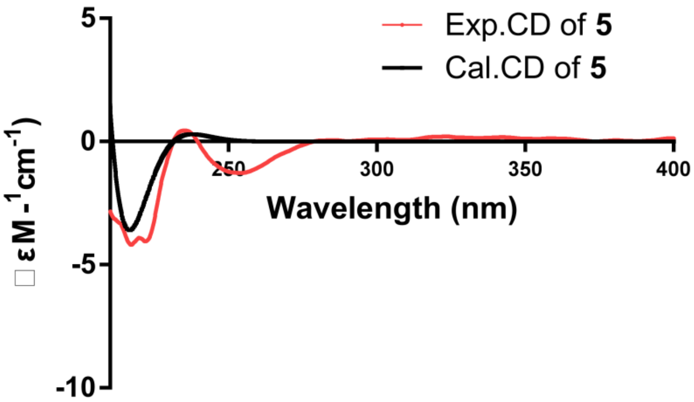

Low-energy conformers of (1*R*, 2*R*, 4*S*, 5*S*, 9*S*)-**7** obtained at the ωB97X/TZVP PCM/MeOH level of theory

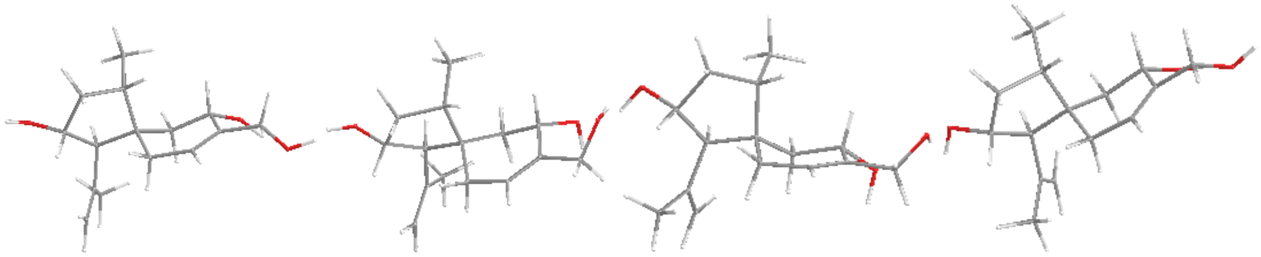

Conf. A: 18.25% Conf.B: 30.52% Conf. C: 35.45% Conf.D: 15.79%

conformers	energy	percentage %
Conf.A	-811.91735	18.25
Conf.B	-811.91783	30.52
Conf.C	-811.9179761	35.45
Conf.D	-811.917213	15.79

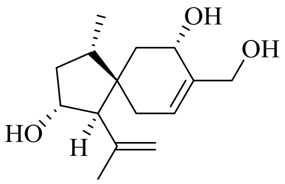

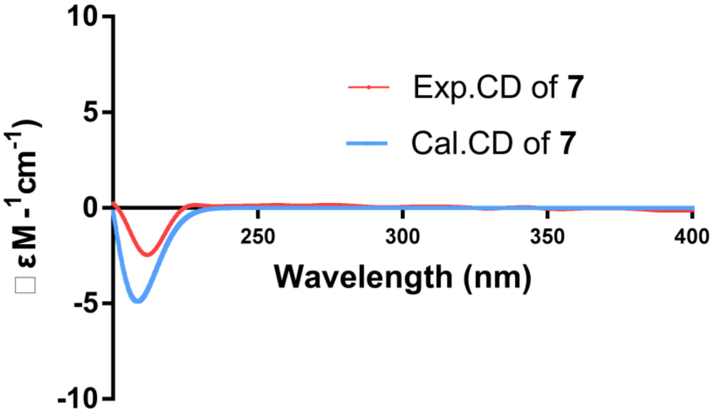

Low-energy conformers of (1*R*, 2*R*, 4*S*, 5*R*, 6*R*, 9*S*)-**8** obtained at the ωB97X/TZVP PCM/MeOH level of theory

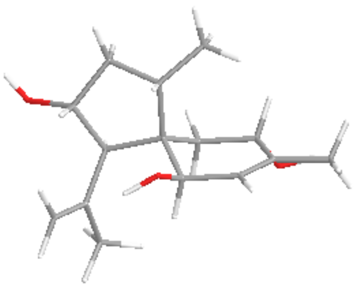
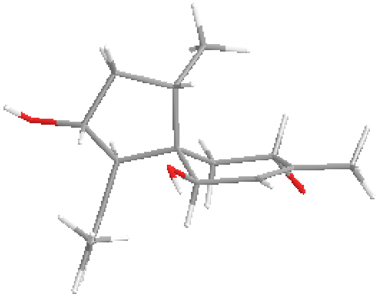
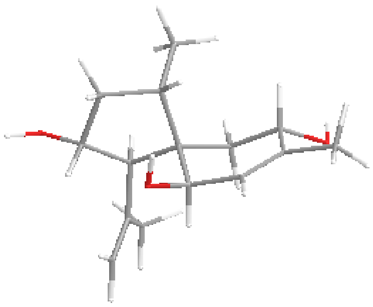

Conf. A 33.33% Conf.B 33.33% Conf. C 33.33%

conformers	energy	percentage %
Conf.A	-811.918	33.33
Conf.B	-811.916	33.33
Conf.C	-811.918	33.33

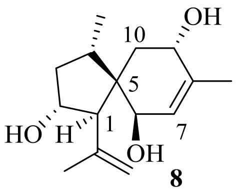

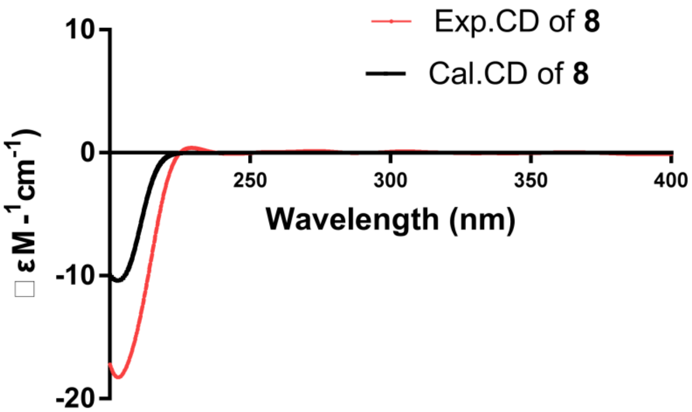

Low-energy conformers of (1*R*, 2*R*, 4*S*, 5*R*, 6*R*, 9*S*)-**9** obtained at the ωB97X/TZVP PCM/MeOH level of theory

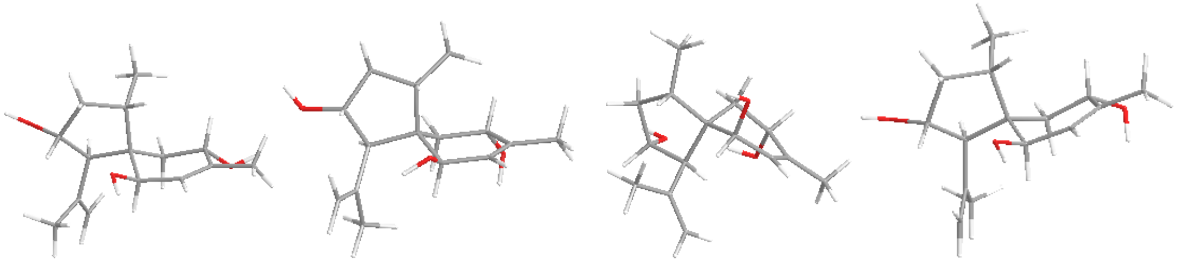

Conf.B 7.5%   Conf. C 57.95%   Conf.D 1.7%   Conf. E 31.93%

conformers	energy	percentage %
Conf.A	-811.915	0.92
Conf.B	-811.917	7.5
Conf.C	-811.919	57.95
Conf.D	-811.916	1.7
Conf.E	-811.918	31.93

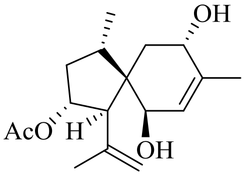

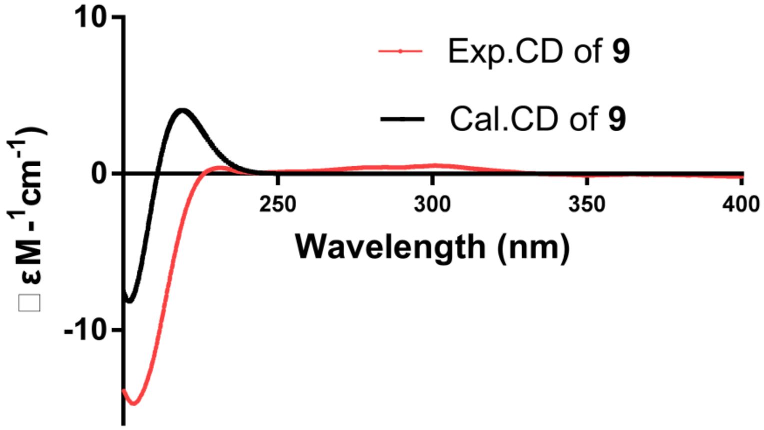

Low-energy conformers of (1*S*, 2*R*, 4*S*, 5*S*, 9*S*)-**10** obtained at the ωB97X/TZVP PCM/MeOH level of theory

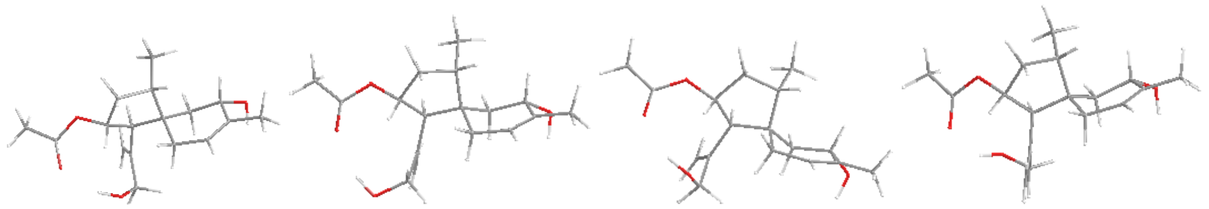

Conf. A 25% Conf.B 25% Conf. C 25% Conf.D 25%

conformers	energy	percentage %
Conf.A	-964.631	25
Conf.B	-964.63	25
Conf.C	-964.631	25
Conf.D	-964.63	25

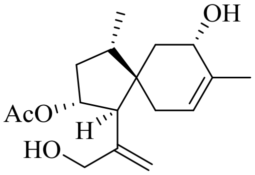

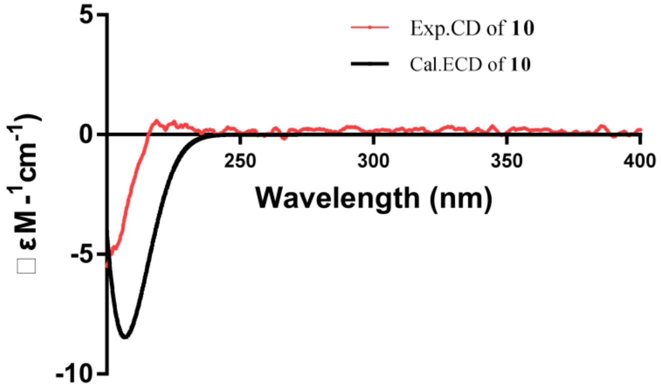

Low-energy conformers of (1*R*, 2*R*, 4*S*, 5*S*, 8*S*, 9*S*)-**11** obtained at the ωB97X/TZVP PCM/MeOH level of theory

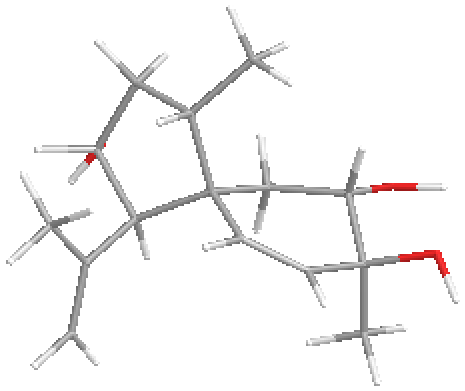

Conf.A 100%

conformers	energy	percentage %
Conf.A	-811.9215555	100

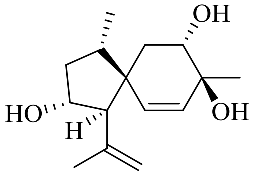

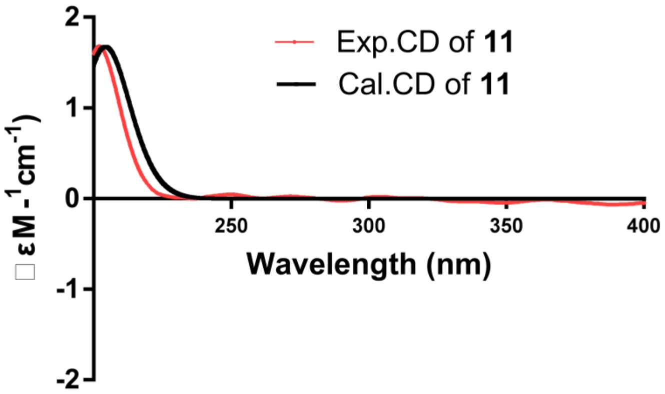

Low-energy conformers of (1*R*, 2*R*, 4*S*, 5*S*, 8*R*, 9*S*)-**13** obtained at the ωB97X/TZVP PCM/MeOH level of theory

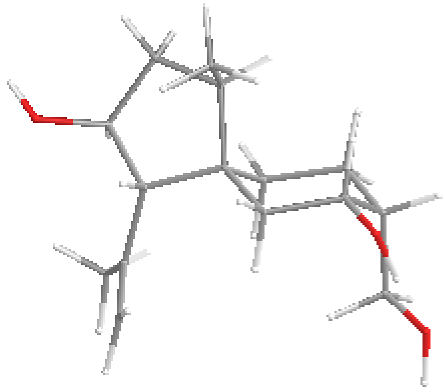

Conf.A 34.49%

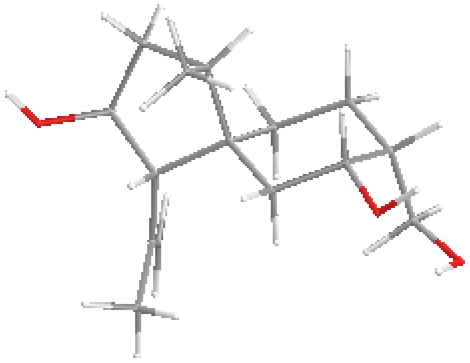

Conf.B 65.51%

conformers	energy	percentage %
Conf.A	-813.1436449	34.49
Conf.B	-813.1442499	65.51

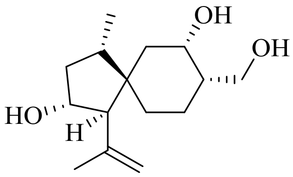

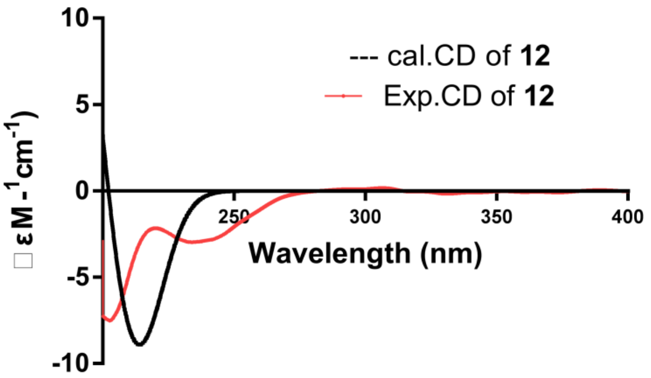

Low-energy conformers of (1*R*, 2*R*, 4*S*, 5*S*, 8*R*, 9*S*)-**13** obtained at the ωB97X/TZVP PCM/MeOH level of theory

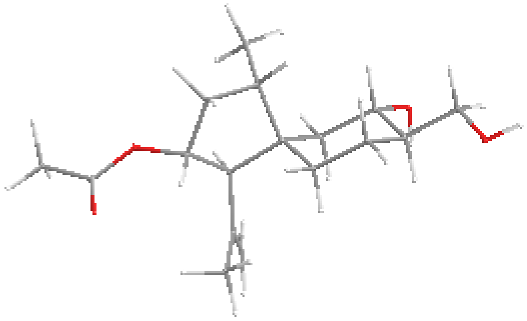

Conf.A 100%

conformers	energy	percentage %
Conf.A	-965.8578494	100

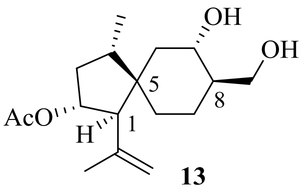

13

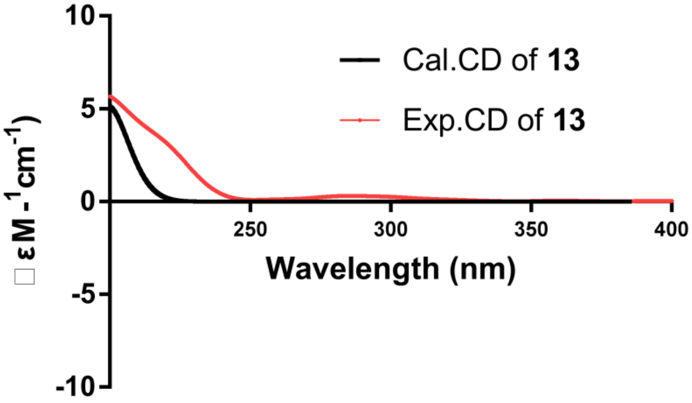

Low-energy conformers of (1*R*, 2*R*, 4*S*, 5*S*, 8*R*, 9*S*)-**14** obtained at the ωB97X/TZVP PCM/MeOH level of theory

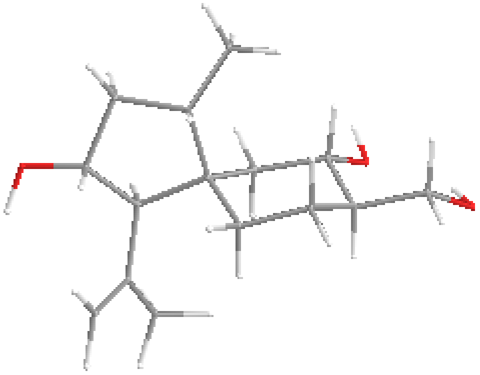

Conf.A 10.9%

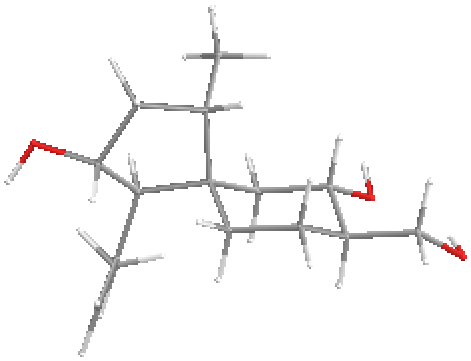

Conf.B 89.1%

conformers	energy	percentage %
Conf.A	-813.162182	10.9
Conf.B	-813.1641635	89.1

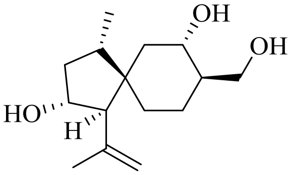

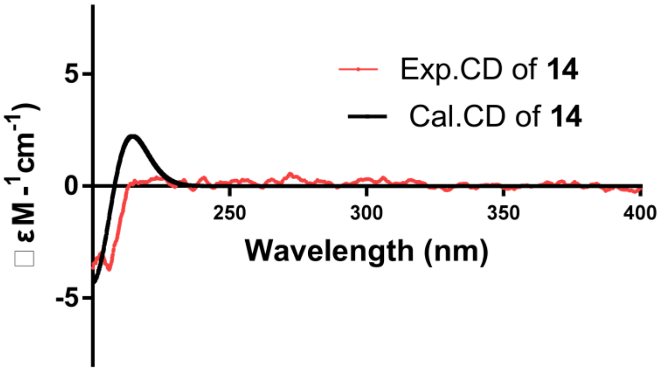

Low-energy conformers of (1*R*, 2*R*, 4*S*, 5*S*, 7S, 8*S*, 9*S*)-**15** obtained at the ωB97X/TZVP PCM/MeOH level of theory

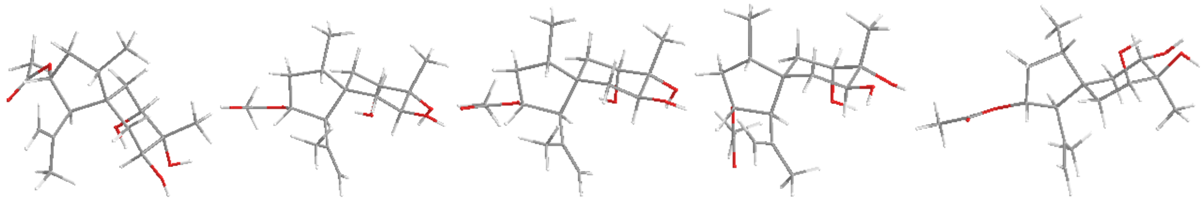

Conf.A: 1.84% Conf.B: 3.42% Conf.C: 3.42% Conf.D:8.83% Conf.E:15.48%

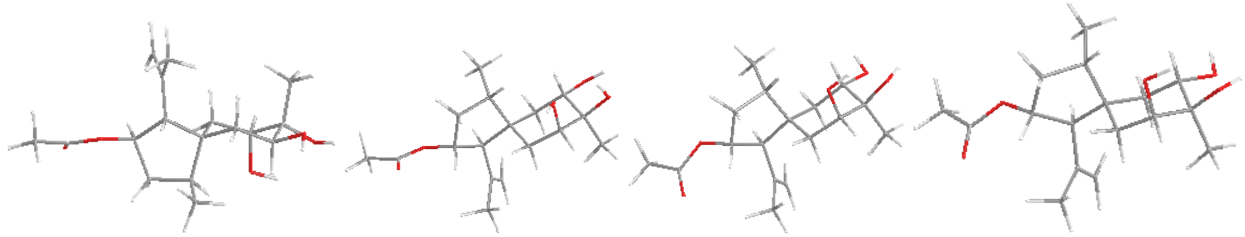

Conf.F: 23.79% Conf.G: 4.56% Conf.H: 18.00% Conf.I: 18.00%

conformers	energy	percentage %
Conf.A	-1041.108883	1.84
Conf.B	-1041.109467	3.42
Conf.C	-1041.109467	3.42
Conf.D	-1041.110362	8.83
Conf.E	-1041.110892	15.48
Conf.F	-1041.111298	23.79
Conf.G	-1041.109739	4.56
Conf.H	-1041.111035	18
Conf.I	-1041.111035	18

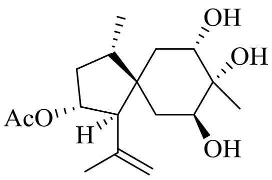

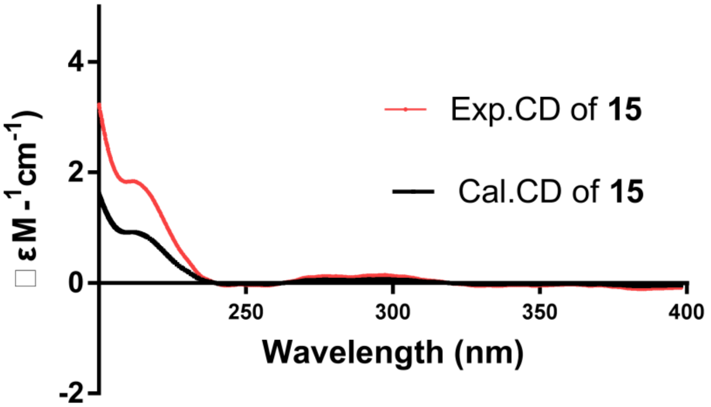

Low-energy conformers of (1*R*, 2*R*, 4*S*, 5*S*, 7S, 8*S*, 9*S*)-**16** obtained at the ωB97X/TZVP PCM/MeOH level of theory

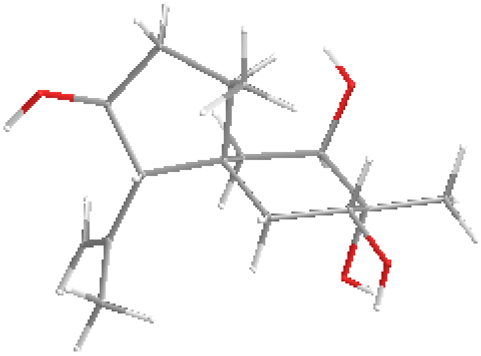

Conf.A: 80.91%

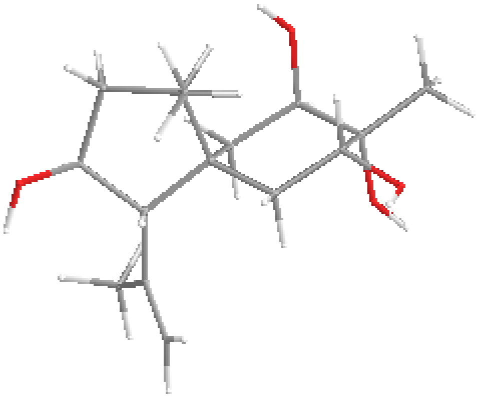

Conf.B: 19.09%

conformers	energy	percentage %
Conf.A	-888.41	80.91
Conf.B	-888.409	19.09

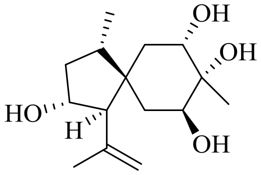

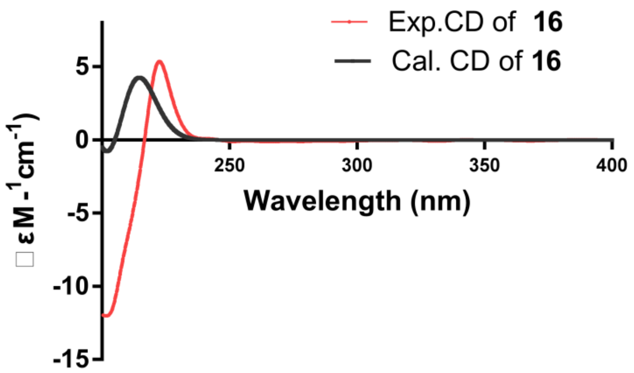

Low-energy conformers of (1*R*, 2*R*, 4*S*, 5*R*, 8*R*, 9*S*)-**17** obtained at the ωB97X/TZVP PCM/MeOH level of theory

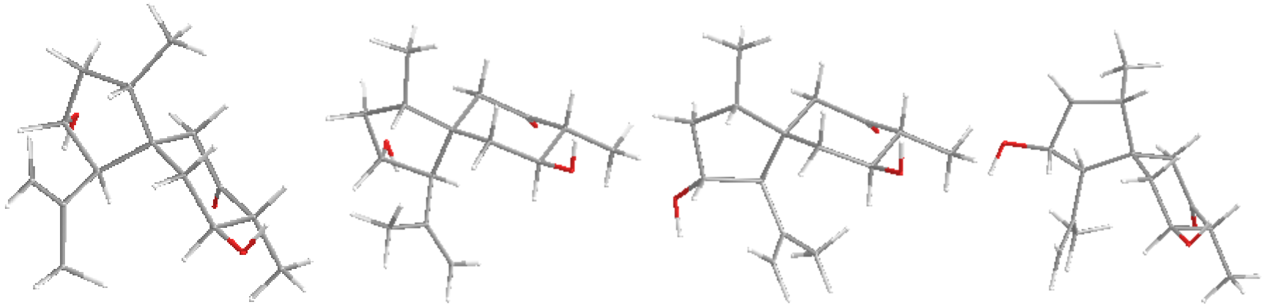

Conf. A 6.72% Conf. B: 58.81% Conf. C: 30.8% Conf. D: 3.67%

conformers	energy	percentage %
Conf.A	-811.943	6.72
Conf.B	-811.945	58.81
Conf.C	-811.944	30.8
Conf.D	-811.942	3.67

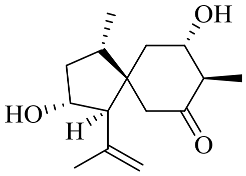

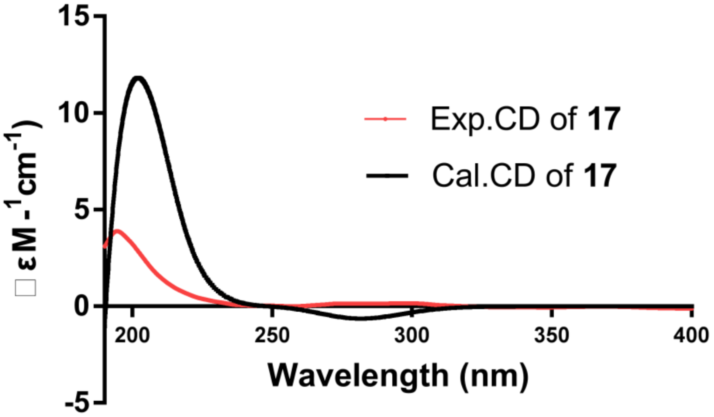

Low-energy conformers of (1*R*, 2*R*, 4*S*, 5*R*, 8*R*, 9*S*)-**18** obtained at the ωB97X/TZVP PCM/MeOH level of theory

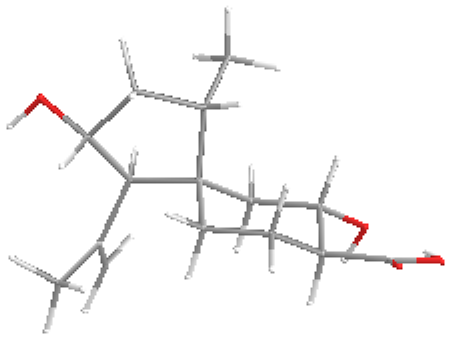

Conf.A 100%

conformers	energy	percentage %
Conf.A	-887.2163863	100

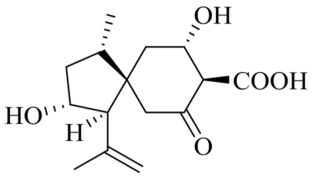

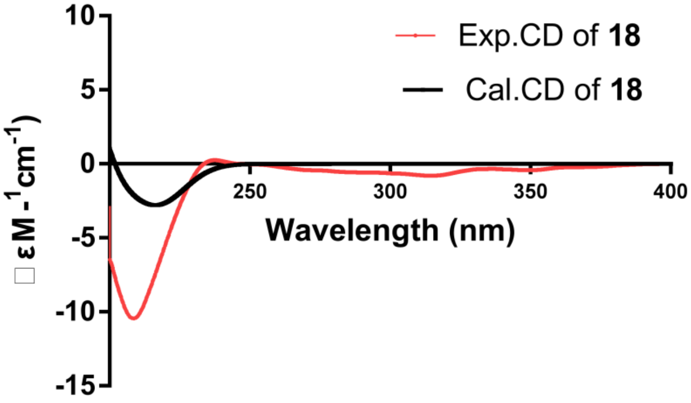

Fig. S164. ORTEP drawing of **1**.

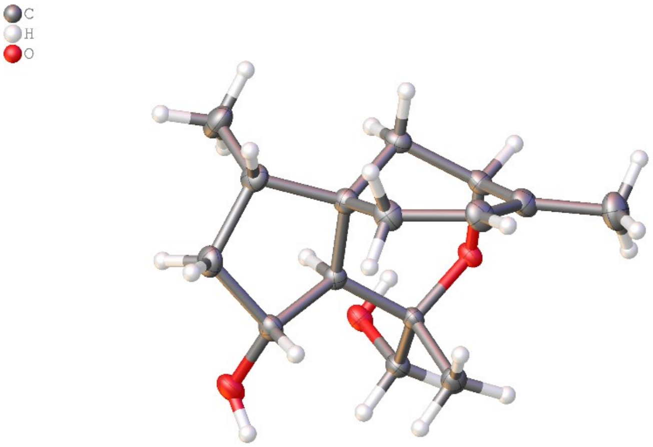

Table S8. Crystal data and structure refinement for **1.**

Identification code	54213
Empirical formula	$C_{15}H_{26}O_4$
Formula weight	270.36
Temperature/K	99.95(15)
Crystal system	tetragonal
Space group	$P4_12_12$
a/Å	8.72523(6)
b/Å	8.72523(6)
c/Å	38.7922(5)
α/°	90
β/°	90
γ/°	90
Volume/Å^3	2953.24(6)
Z	8
ρ_{calc}g/cm^3	1.216
μ/mm^{-1}	0.699
F(000)	1184.0
Crystal size/mm^3	0.09 × 0.06 × 0.05
Radiation	Cu $K\alpha$ (λ = 1.54184)
2Θ range for data collection/°	9.118 to 150.046
Index ranges	$-10 \leq h \leq 10$, $-10 \leq k \leq 10$, $-47 \leq l \leq 42$
Reflections collected	29545
Independent reflections	2987 [R_{int} = 0.0595, R_{sigma} = 0.0251]
Data/restraints/parameters	2987/0/180
Goodness-of-fit on F^2	1.053
Final R indexes [I>=2σ (I)]	R_1 = 0.0291, wR_2 = 0.0758
Final R indexes [all data]	R_1 = 0.0303, wR_2 = 0.0765

Largest diff. peak/hole / e Å^{-3}	0.15/-0.17
Flack parameter	0.00(6)

Table S9. Fractional Atomic Coordinates (×10^4) and Equivalent Isotropic Displacement Parameters (Å^2×10^3) for **1**. U_{eq} is defined as 1/3 of of the trace of the orthogonalised U_{IJ} tensor.

**Atom**	***x***	***y***	***z***	**U(eq)**
O1	5640.2(11)	1843.7(12)	7376.3(2)	17.2(2)
O12	8366.2(12)	2644.1(13)	7035.6(3)	20.0(2)
O2	6393.6(13)	1873.9(13)	6211.1(3)	24.9(2)
O1W	6315.4(14)	-1129.6(14)	6106.6(3)	29.2(3)
C5	3608.4(17)	3193.1(17)	6870.6(4)	18.5(3)
C10	3779.8(17)	3857.7(17)	7230.3(4)	19.2(3)
C11	5855.7(17)	1289.8(16)	7023.2(3)	16.7(3)
C6	2215.7(18)	2087.2(19)	6878.5(4)	23.7(3)
C2	4984.6(17)	2091.9(17)	6391.6(4)	19.7(3)
C12	7611.2(17)	1211.6(17)	6998.6(4)	19.5(3)
C4	3393.8(18)	4344.5(18)	6566.5(4)	22.2(3)
C1	5175.9(16)	2476.8(16)	6774.1(3)	16.2(3)
C9	4186.5(16)	2530.6(17)	7469.7(4)	18.9(3)
C8	2930.0(17)	1345.0(19)	7481.0(4)	23.0(3)
C7	2027.4(18)	1196(2)	7207.1(4)	25.0(3)
C3	4130.4(19)	3513.0(19)	6254.2(4)	24.7(3)
C13	5276.7(19)	-354.2(18)	6977.4(4)	22.9(3)
C14	4125(2)	5911.8(19)	6627.2(5)	30.7(4)
C15	2867(2)	364(2)	7798.2(4)	31.6(4)

Table S10. Anisotropic Displacement Parameters (Å^2×10^3) for **1**. The Anisotropic displacement factor exponent takes the form: $-2\pi^2[h^2a^{*2}U_{11}+2hka^*b^*U_{12}+\ldots]$.

**Atom**	**U_{11}**	**U_{22}**	**U_{33}**	**U_{23}**	**U_{13}**	**U_{12}**
O1	15.7(5)	22.5(5)	13.5(5)	-1.9(4)	0.7(4)	0.2(4)
O12	18.4(5)	25.1(6)	16.4(5)	-1.9(4)	2.3(4)	-1.5(4)
O2	29.8(6)	27.0(6)	17.9(5)	-4.2(4)	5.5(4)	-1.9(5)
O1W	34.3(6)	29.7(6)	23.7(5)	-5.0(5)	2.5(5)	7.9(5)
C5	18.1(7)	20.4(7)	17.1(7)	-0.7(5)	-2.3(5)	0.0(6)
C10	16.2(7)	22.1(7)	19.1(7)	-3.1(6)	0.8(5)	1.1(5)
C11	20.3(7)	17.3(7)	12.5(6)	-0.7(5)	1.4(5)	-0.1(5)
C6	18.0(7)	30.8(9)	22.3(7)	-2.0(6)	-3.0(6)	-3.6(6)
C2	22.5(7)	21.2(7)	15.5(7)	-1.6(5)	-0.1(6)	-2.4(6)
C12	20.7(7)	19.9(7)	18.0(7)	0.2(5)	2.4(5)	1.8(6)
C4	21.0(7)	23.8(8)	21.6(7)	1.2(6)	-5.2(6)	2.5(6)
C1	17.0(7)	16.2(7)	15.6(6)	-0.8(5)	-0.9(5)	-2.0(5)
C9	16.3(7)	24.5(7)	15.9(6)	-2.7(6)	2.0(5)	-0.6(6)
C8	19.7(7)	27.4(8)	22.0(7)	-0.2(6)	5.5(6)	-2.2(6)
C7	18.7(7)	29.7(8)	26.7(8)	-0.5(6)	3.2(6)	-7.1(6)

C3	29.7(8)	26.8(8)	17.6(7)	2.1(6)	-4.7(6)	-0.5(7)
C13	29.4(8)	18.6(7)	20.7(7)	-0.2(6)	1.5(6)	-2.5(6)
C14	42.7(10)	20.3(8)	29.0(8)	3.9(7)	-8.1(7)	1.7(7)
C15	28.3(9)	39.3(10)	27.2(8)	6.6(7)	3.0(7)	-8.4(7)

Table S11. Bond Lengths for **1.**

**Atom**	**Atom**	**Length/Å**	**Atom**	**Atom**	**Length/Å**
O1	C11	1.4648(16)	C11	C1	1.536(2)
O1	C9	1.4488(17)	C11	C13	1.531(2)
O12	C12	1.4201(19)	C6	C7	1.502(2)
O2	C2	1.4274(18)	C2	C1	1.5305(19)
C5	C10	1.519(2)	C2	C3	1.542(2)
C5	C6	1.552(2)	C4	C3	1.551(2)
C5	C4	1.561(2)	C4	C14	1.527(2)
C5	C1	1.550(2)	C9	C8	1.508(2)
C10	C9	1.526(2)	C8	C7	1.329(2)
C11	C12	1.536(2)	C8	C15	1.500(2)

Table S12. Bond Angles for **1.**

**Atom**	**Atom**	**Atom**	**Angle/°**	**Atom**	**Atom**	**Atom**	**Angle/°**
C9	O1	C11	118.87(10)	C1	C2	C3	102.20(12)
C10	C5	C6	107.23(12)	O12	C12	C11	114.66(12)
C10	C5	C4	117.42(12)	C3	C4	C5	103.85(12)
C10	C5	C1	106.80(11)	C14	C4	C5	114.18(13)
C6	C5	C4	108.74(12)	C14	C4	C3	111.49(14)
C1	C5	C6	116.43(12)	C11	C1	C5	117.45(12)
C1	C5	C4	100.54(11)	C2	C1	C5	103.09(11)
C5	C10	C9	107.00(12)	C2	C1	C11	120.28(12)
O1	C11	C12	101.58(11)	O1	C9	C10	111.41(11)
O1	C11	C1	108.44(11)	O1	C9	C8	111.10(12)
O1	C11	C13	112.04(11)	C8	C9	C10	111.66(13)
C1	C11	C12	112.08(12)	C7	C8	C9	118.34(14)
C13	C11	C12	106.27(13)	C7	C8	C15	125.35(15)
C13	C11	C1	115.55(12)	C15	C8	C9	116.22(14)
C7	C6	C5	115.11(13)	C8	C7	C6	124.27(15)
O2	C2	C1	114.25(12)	C2	C3	C4	107.86(12)
O2	C2	C3	110.74(12)				

Table S13. Torsion Angles for **1.**

A	B	C	D	Angle/°	A	B	C	D	Angle/°
O1	C11	C12	O12	-62.64(14)	C6	C5	C1	C2	69.65(15)
O1	C11	C1	C5	-42.76(16)	C12	C11	C1	C5	-154.09(12)
O1	C11	C1	C2	-169.54(12)	C12	C11	C1	C2	79.14(16)
O1	C9	C8	C7	-97.52(17)	C4	C5	C10	C9	-172.48(12)
O1	C9	C8	C15	79.12(16)	C4	C5	C6	C7	-164.20(13)
O2	C2	C1	C5	160.94(12)	C4	C5	C1	C11	177.67(12)
O2	C2	C1	C11	-65.93(17)	C4	C5	C1	C2	-47.58(13)
O2	C2	C3	C4	-141.17(13)	C1	C5	C10	C9	-60.65(14)
C5	C10	C9	O1	62.70(15)	C1	C5	C6	C7	83.19(17)
C5	C10	C9	C8	-62.18(15)	C1	C5	C4	C3	34.50(14)
C5	C6	C7	C8	2.1(2)	C1	C5	C4	C14	-87.12(15)
C5	C4	C3	C2	-9.94(16)	C1	C11	C12	O12	52.93(16)
C10	C5	C6	C7	-36.29(18)	C1	C2	C3	C4	-19.08(16)
C10	C5	C4	C3	149.83(13)	C9	O1	C11	C12	160.73(12)
C10	C5	C4	C14	28.21(19)	C9	O1	C11	C1	42.51(16)
C10	C5	C1	C11	54.61(16)	C9	O1	C11	C13	-86.22(15)
C10	C5	C1	C2	-170.63(12)	C9	C8	C7	C6	2.8(3)
C10	C9	C8	C7	27.5(2)	C3	C2	C1	C5	41.28(14)
C10	C9	C8	C15	-155.83(14)	C3	C2	C1	C11	174.41(13)
C11	O1	C9	C10	-55.20(16)	C13	C11	C12	O12	-179.96(12)
C11	O1	C9	C8	69.98(15)	C13	C11	C1	C5	83.96(16)
C6	C5	C10	C9	64.84(15)	C13	C11	C1	C2	-42.81(18)
C6	C5	C4	C3	-88.27(14)	C14	C4	C3	C2	113.46(14)
C6	C5	C4	C14	150.11(14)	C15	C8	C7	C6	-173.53(16)
C6	C5	C1	C11	-65.10(17)					

Table S14. Hydrogen Atom Coordinates (Å×10^4) and Isotropic Displacement Parameters (Å^2×10^3) for **1.**

**Atom**	***x***	***y***	***z***	**U(eq)**
H12	8150.05	3018.08	7223.3	30
H2	6517.21	958.4	6172.27	37
H1WA	6311.23	-1348.13	5893.24	44
H1WB	7010.56	-1699.21	6191.61	44
H10A	2829.28	4334.85	7303.08	23
H10B	4584.05	4625.05	7233.66	23
H6A	1290.82	2679.19	6839.47	28
H6B	2315.14	1367.71	6689.22	28
H2A	4337.31	1181.07	6366.05	24
H12A	7985.16	521.24	7175.6	23
H12B	7886.04	777.04	6776.87	23
H4	2296.05	4480.72	6522.68	27
H1	5899.24	3335.82	6778.66	19
H9	4295.63	2950.45	7702.76	23
H7	1226.96	494.5	7219.64	30
H3A	3343.98	3205.45	6091.6	30
H3B	4840.4	4192.16	6137.25	30
H13A	5618.57	-970.75	7167.37	34
H13B	5671.59	-768.34	6766	34
H13C	4176.95	-354.29	6970.02	34
H14A	3620.62	6411.52	6816.33	46
H14B	4021.23	6525.99	6423.22	46
H14C	5191.56	5783.19	6680.34	46
H15A	3852.49	-95.1	7836.84	47
H15B	2113.52	-426.09	7767.66	47
H15C	2595.84	984.55	7993.22	47

Figure S165. ORTEP of **2**

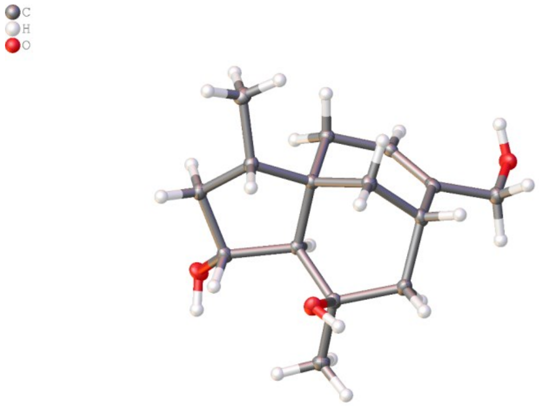

Table S15. Crystal data and structure refinement for **2.**

Identification code	542542
Empirical formula	$C_{15}H_{26}O_4$
Formula weight	270.36
Temperature/K	T
Crystal system	trigonal
Space group	*R*3
a/Å	24.3255(3)
b/Å	24.3255(3)
c/Å	6.21200(10)
α/°	90
β/°	90
γ/°	120
Volume/Å^3	3183.36(9)
Z	9
ρ_{calc}g/cm^3	1.269
μ/mm^{-1}	0.730
F(000)	1332.0
Crystal size/mm^3	0.13 × 0.03 × 0.03
Radiation	Cu *Kα* (λ = 1.54184)
2Θ range for data collection/°	7.268 to 146.45
Index ranges	$-30 \leq h \leq 30, -30 \leq k \leq 29, -7 \leq l \leq 7$
Reflections collected	7183
Independent reflections	2777 [R_{int} = 0.0847, R_{sigma} = 0.0885]
Data/restraints/parameters	2777/1/180
Goodness-of-fit on F^2	1.040
Final *R* indexes [I>=2σ (I)]	R_1 = 0.0288, wR_2 = 0.0764
Final *R* indexes [all data]	R_1 = 0.0409, wR_2 = 0.0770

Largest diff. peak/hole / e Å^{-3}	0.18/-0.18
Flack parameter	-0.05(9)

Table S16. Fractional Atomic Coordinates (×10^4) and Equivalent Isotropic Displacement Parameters (Å^2×10^3) for **2**. U_{eq} is defined as 1/3 of of the trace of the orthogonalised U_{IJ} tensor.

**Atom**	***x***	***y***	***z***	**U(eq)**
O11	-6472.3(6)	-8002.8(6)	-3864(2)	15.3(3)
O1W	-6850.4(7)	-7158.9(7)	-2633(2)	17.7(3)
O15	-4735.6(7)	-9049.9(7)	-9204(2)	18.8(3)
O2	-5195.3(7)	-6421.2(7)	-7297(2)	17.0(3)
C7	-4610.3(9)	-8051.9(9)	-6553(3)	13.7(4)
C5	-5083.7(8)	-7588.0(8)	-4033(3)	11.9(4)
C11	-6180.0(9)	-8012.2(9)	-5896(3)	13.1(4)
C4	-4947.4(9)	-7071.9(9)	-2334(3)	13.1(4)
C10	-5430.4(9)	-8264.8(9)	-3109(3)	13.4(4)
C1	-5484.5(8)	-7482.8(8)	-5798(3)	11.4(4)
C13	-6539.9(9)	-7910.5(10)	-7719(3)	16.1(4)
C14	-4399.0(9)	-6908.4(10)	-805(3)	16.2(4)
C8	-5152.0(9)	-8606.4(9)	-6518(3)	13.9(4)
C2	-5384.1(8)	-6807.1(8)	-5385(3)	12.9(4)
C15	-5267.5(9)	-9155.5(10)	-7929(3)	16.5(4)
C6	-4474.6(9)	-7500.7(9)	-5105(3)	12.8(4)
C3	-4870.6(9)	-6509.6(9)	-3666(3)	14.9(4)
C9	-5692.8(9)	-8728.5(9)	-4996(3)	14.0(4)
C12	-6221.4(9)	-8662.6(9)	-6175(3)	15.1(4)

Table S17. Anisotropic Displacement Parameters (Å^2×10^3) for **2**. The Anisotropic displacement factor exponent takes the form: $-2\pi^2[h^2a^{*2}U_{11}+2hka^*b^*U_{12}+\ldots]$.

**Atom**	**U_{11}**	**U_{22}**	**U_{33}**	**U_{23}**	**U_{13}**	**U_{12}**
O11	11.9(6)	14.7(7)	17.5(6)	0.7(5)	5.1(5)	5.3(6)
O1W	19.0(7)	16.7(7)	19.8(7)	1.6(6)	4.2(6)	10.7(6)
O15	16.5(7)	20.0(7)	23.2(7)	-6.8(6)	-2.6(5)	11.6(6)
O2	17.4(7)	16.0(7)	18.4(7)	6.8(5)	1.8(5)	9.0(6)
C7	14.5(9)	16.4(9)	14.2(9)	-0.2(7)	-0.3(7)	10.7(7)
C5	11.6(8)	11.7(9)	13.3(8)	-0.1(7)	0.7(7)	6.5(7)
C11	10.0(8)	12.8(9)	15.6(9)	0.6(7)	3.2(7)	5.1(7)
C4	13.5(9)	12.0(9)	13.3(9)	0.0(7)	1.4(7)	6.0(7)
C10	14.9(9)	12.0(9)	13.5(8)	2.2(7)	1.0(7)	6.9(7)
C1	10.3(9)	11.2(8)	12.4(8)	1.2(6)	1.3(6)	5.1(7)
C13	12.1(8)	17.2(9)	19.2(10)	-0.7(7)	-1.0(7)	7.4(8)
C14	17.3(9)	17.0(9)	13.0(9)	-0.6(7)	-1.2(7)	7.5(8)
C8	16.1(9)	16.0(9)	14.2(9)	-0.1(7)	-1.7(7)	11.6(8)
C2	11.9(9)	11.6(9)	15.8(8)	2.4(7)	2.5(7)	6.3(7)
C15	15.9(9)	14.4(9)	20.9(10)	-2.8(7)	-0.7(7)	8.8(8)
C6	11.7(8)	13.4(9)	13.8(8)	-0.5(7)	-0.5(7)	6.7(7)
C3	14.7(9)	11.9(8)	17.9(9)	-0.9(7)	-0.6(7)	6.5(7)
C9	14.4(9)	10.5(8)	17.7(9)	1.9(7)	2.3(7)	6.7(7)
C12	10.8(8)	13.0(9)	20.2(9)	-2.4(7)	0.4(7)	5.0(7)

Table S18. Bond Lengths for **2.**

Atom	Atom	Length/Å	Atom	Atom	Length/Å
O11	C11	1.454(2)	C11	C13	1.525(3)
O15	C15	1.427(2)	C11	C12	1.544(3)
O2	C2	1.439(2)	C4	C14	1.520(3)
C7	C8	1.334(3)	C4	C3	1.528(3)
C7	C6	1.508(2)	C10	C9	1.528(3)
C5	C4	1.544(2)	C1	C2	1.557(2)
C5	C10	1.537(3)	C8	C15	1.502(3)
C5	C1	1.571(2)	C8	C9	1.524(3)
C5	C6	1.539(2)	C2	C3	1.523(3)
C11	C1	1.532(2)	C9	C12	1.556(3)

Table S19. Bond Angles for **2.**

Atom	Atom	Atom	Angle/°	Atom	Atom	Atom	Angle/°
C8	C7	C6	123.77(17)	C11	C1	C5	113.92(15)
C4	C5	C1	104.06(14)	C11	C1	C2	114.72(15)
C10	C5	C4	113.28(15)	C2	C1	C5	105.94(14)
C10	C5	C1	112.49(14)	C7	C8	C15	122.73(17)
C10	C5	C6	106.52(14)	C7	C8	C9	121.84(17)
C6	C5	C4	112.52(15)	C15	C8	C9	115.42(16)
C6	C5	C1	107.98(15)	O2	C2	C1	112.53(15)
O11	C11	C1	107.19(14)	O2	C2	C3	110.49(15)
O11	C11	C13	108.49(15)	C3	C2	C1	105.99(15)
O11	C11	C12	109.37(15)	O15	C15	C8	114.48(16)
C1	C11	C12	110.16(15)	C7	C6	C5	111.57(15)
C13	C11	C1	112.06(15)	C2	C3	C4	104.49(15)
C13	C11	C12	109.51(15)	C10	C9	C12	110.29(15)
C14	C4	C5	116.32(16)	C8	C9	C10	109.31(16)
C14	C4	C3	113.94(16)	C8	C9	C12	111.44(16)
C3	C4	C5	103.76(14)	C11	C12	C9	114.75(16)
C9	C10	C5	107.87(15)				

Table S20. Hydrogen Bonds for **2.**

D	H	A	d(D-H)/Å	d(H-A)/Å	d(D-A)/Å	D-H-A/°
O11	H11	O15[1]	0.82	1.97	2.7674(19)	162.6
O1W	H1WA	O1W[2]	0.85	1.91	2.7537(16)	171.0
O1W	H1WB	O11	0.85	1.91	2.7447(19)	168.3
O15	H15	O2[3]	0.82	2.02	2.808(2)	160.1
O2	H2	O1W[4]	0.82	1.95	2.759(2)	167.4

[1]-5/3-Y,-4/3+X-Y,2/3+Z; [2]-2/3+Y-X,-4/3-X,-1/3+Z; [3]-1-Y,-1+X-Y,+Z; [4]-4/3-Y,-2/3+X-Y,-2/3+Z

Table S21. Torsion Angles for **2.**

A	B	C	D	Angle/°	A	B	C	D	Angle/°
O11	C11	C1	C5	64.58(19)	C1	C5	C4	C14	-160.77(16)
O11	C11	C1	C2	-57.8(2)	C1	C5	C4	C3	-34.81(17)
O11	C11	C12	C9	-82.6(2)	C1	C5	C10	C9	47.3(2)
O2	C2	C3	C4	-151.84(15)	C1	C5	C6	C7	-72.73(18)
C7	C8	C15	O15	4.0(3)	C1	C11	C12	C9	34.9(2)
C7	C8	C9	C10	-21.6(2)	C1	C2	C3	C4	-29.66(19)
C7	C8	C9	C12	100.5(2)	C13	C11	C1	C5	-176.51(16)
C5	C4	C3	C2	40.34(18)	C13	C11	C1	C2	61.2(2)
C5	C10	C9	C8	56.2(2)	C13	C11	C12	C9	158.62(16)
C5	C10	C9	C12	-66.7(2)	C14	C4	C3	C2	167.80(16)
C5	C1	C2	O2	128.70(15)	C8	C7	C6	C5	-14.8(2)
C5	C1	C2	C3	7.83(19)	C8	C9	C12	C11	-97.43(19)
C11	C1	C2	O2	-104.74(18)	C15	C8	C9	C10	157.19(16)
C11	C1	C2	C3	134.39(16)	C15	C8	C9	C12	-80.6(2)
C4	C5	C10	C9	164.93(15)	C6	C7	C8	C15	-178.01(17)
C4	C5	C1	C11	-110.47(16)	C6	C7	C8	C9	0.7(3)
C4	C5	C1	C2	16.58(18)	C6	C5	C4	C14	-44.1(2)
C4	C5	C6	C7	173.00(15)	C6	C5	C4	C3	81.83(18)
C10	C5	C4	C14	76.8(2)	C6	C5	C10	C9	-70.85(18)
C10	C5	C4	C3	-157.29(15)	C6	C5	C1	C11	129.77(16)
C10	C5	C1	C11	12.5(2)	C6	C5	C1	C2	-103.18(16)
C10	C5	C1	C2	139.58(15)	C9	C8	C15	O15	-174.83(16)
C10	C5	C6	C7	48.31(19)	C12	C11	C1	C5	-54.3(2)
C10	C9	C12	C11	24.2(2)	C12	C11	C1	C2	-176.66(15)

Table S22. Hydrogen Atom Coordinates (Å×10^4) and Isotropic Displacement Parameters (Å^2×10^3) for **2.**

**Atom**	***x***	***y***	***z***	**U(eq)**
H11	-6803.46	-8340.7	-3702.85	23
H1WA	-6925.04	-6987.72	-3699.47	27
H1WB	-6734.55	-7403.55	-3194.93	27
H15	-4421.2	-8921.11	-8433.75	28
H2	-5511.4	-6462.04	-7914.8	26
H7	-4298.28	-8002.36	-7526.54	16
H4	-5331.47	-7221.4	-1459.02	16
H10A	-5138.74	-8341.8	-2272.69	16
H10B	-5773.92	-8319.64	-2174.96	16
H1	-5293.07	-7472.55	-7196.15	14
H13A	-6550.39	-7526.53	-7466.69	24
H13B	-6965.77	-8263.05	-7776.3	24
H13C	-6329.92	-7877.98	-9060.76	24
H14A	-4005.27	-6686.15	-1576.96	24
H14B	-4441.65	-7291.7	-206.66	24
H14C	-4403.47	-6644	334.23	24
H2A	-5778.57	-6844.82	-4829.85	15
H15A	-5390.33	-9524.51	-7026.54	20
H15B	-5620.82	-9252.02	-8879.58	20
H6A	-4281.33	-7112.03	-5942.39	15
H6B	-4175.12	-7460.78	-3999.63	15
H3A	-4932.03	-6216.34	-2780.31	18
H3B	-4452.37	-6284.12	-4316.25	18
H9	-5878.23	-9161.81	-4435.11	17
H12A	-6201.58	-8737.59	-7699.54	18
H12B	-6631	-8990.47	-5649.14	18

Fig. S166. ORTEP drawing of **9**.

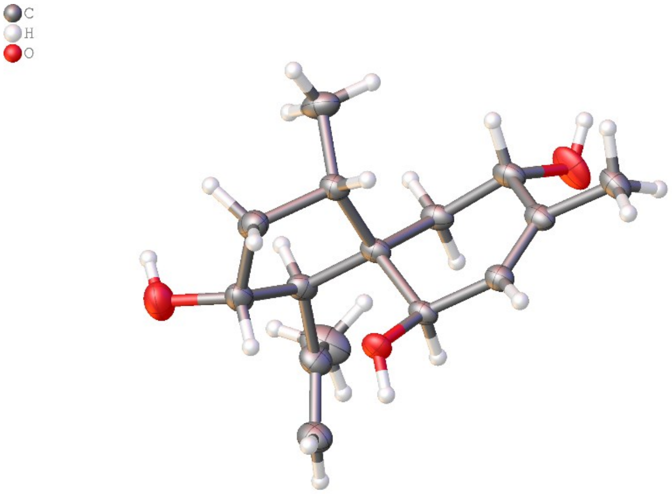

Table S23. Crystal data and structure refinement for **9.**

Identification code	5111G
Empirical formula	$C_{15}H_{24.5}O_{3.25}$
Formula weight	256.84
Temperature/K	100.00(10)
Crystal system	tetragonal
Space group	$P42_12$
a/Å	15.42700(10)
b/Å	15.42700(10)
c/Å	15.3826(2)
α/°	90
β/°	90
γ/°	90
Volume/Å^3	3660.94(7)
Z	8
ρ_{calc}g/cm^3	0.932
μ/mm^{-1}	0.514
F(000)	1124.0
Crystal size/mm^3	0.14 × 0.12 × 0.06
Radiation	Cu $K\alpha$ (λ = 1.54184)
2Θ range for data collection/°	8.106 to 150.23
Index ranges	$-17 \leq h \leq 19$, $-15 \leq k \leq 19$, $-19 \leq l \leq 18$
Reflections collected	15415
Independent reflections	3677 [R_{int} = 0.0342, R_{sigma} = 0.0278]
Data/restraints/parameters	3677/0/175
Goodness-of-fit on F^2	1.064
Final R indexes [I>=2σ (I)]	R_1 = 0.0366, wR_2 = 0.1017
Final R indexes [all data]	R_1 = 0.0386, wR_2 = 0.1037
Largest diff. peak/hole / e Å^{-3}	0.27/-0.29
Flack parameter	0.01(10)

Table S24. Fractional Atomic Coordinates (×10^4) and Equivalent Isotropic Displacement Parameters (Å^2×10^3) for **9**. U_{eq} is defined as 1/3 of of the trace of the orthogonalised U_{IJ} tensor.

**Atom**	***x***	***y***	***z***	**U(eq)**
O1W	5000	5000	5000	22.6(4)
O6	6367.0(8)	5067.1(8)	6102.0(7)	24.3(3)
O9	8546.0(10)	7852.6(9)	5739.6(11)	42.7(4)
O2	5743.0(10)	5472.6(10)	8753.8(9)	39.6(4)
C7	7470.0(11)	5880.3(11)	5362.9(11)	22.9(3)
C8	8172.5(11)	6374.4(11)	5359.0(11)	24.8(3)
C6	6767.0(10)	5907.1(10)	6041.0(11)	21.3(3)
C4	7673.1(11)	5547.0(11)	7387.2(12)	24.2(3)
C9	8338.2(11)	7011.8(11)	6084.0(12)	26.6(4)
C1	6375.4(11)	6389.9(11)	7608.8(11)	24.8(3)
C10	7570.3(11)	7099.2(11)	6704.4(12)	24.9(4)
C2	6149.0(11)	5478.9(12)	7918.4(12)	26.9(4)
C5	7107.8(11)	6243.8(10)	6926.8(11)	22.5(3)
C3	7023.4(12)	4984.4(13)	7903.5(11)	29.5(4)
C15	8862.7(11)	6314.1(12)	4671.2(13)	30.1(4)
C14	8357.5(13)	5919.6(13)	8008.0(13)	34.0(4)
C11	5616.3(12)	6960.3(12)	7353.4(12)	30.1(4)
C12	4899.8(13)	6629.4(12)	6923.8(13)	33.6(4)
C13	5650.6(16)	7855.1(14)	7626(2)	51.2(6)

Table S25. Anisotropic Displacement Parameters ($Å^2×10^3$) for **9**. The Anisotropic displacement factor exponent takes the form: $-2\pi^2[h^2a^{*2}U_{11}+2hka^*b^*U_{12}+…]$.

Atom	U_{11}	U_{22}	U_{33}	U_{23}	U_{13}	U_{12}
O1W	18.3(6)	18.3(6)	31.4(11)	0	0	-1.8(9)
O6	22.2(6)	21.9(6)	28.7(5)	-0.9(5)	-1.9(4)	-7.7(5)
O9	42.2(8)	23.3(7)	62.4(9)	-3.0(6)	14.8(7)	-16.6(6)
O2	41.7(8)	45.0(8)	32.1(6)	-2.9(6)	7.4(6)	-9.9(7)
C7	20.7(8)	19.4(7)	28.5(7)	0.5(6)	-0.4(6)	0.7(6)
C8	19.4(7)	20.0(7)	34.9(8)	4.5(7)	-0.6(7)	1.4(6)
C6	16.9(7)	16.3(7)	30.7(8)	0.0(6)	-0.2(6)	-1.8(6)
C4	20.8(7)	21.7(8)	30.0(8)	-0.5(7)	-1.0(7)	-0.9(7)
C9	20.4(7)	20.5(8)	39.0(9)	1.2(7)	-2.2(7)	-3.8(6)
C1	21.7(8)	25.1(8)	27.6(8)	-5.3(7)	-1.0(7)	-2.3(6)
C10	21.2(7)	17.5(7)	36.1(8)	-0.4(6)	-3.9(7)	-2.4(6)
C2	25.2(8)	30.6(9)	24.8(7)	-2.5(7)	0.6(6)	-5.7(7)
C5	18.3(7)	18.1(7)	31.1(8)	-1.6(6)	-1.1(6)	-1.5(6)
C3	29.2(8)	28.7(8)	30.6(8)	4.1(8)	-4.7(7)	-2.8(8)
C15	21.9(8)	25.1(8)	43.2(9)	3.9(7)	7.2(7)	-1.0(6)
C14	30.2(9)	30.6(9)	41.2(9)	0.6(8)	-12.5(8)	-2.4(7)
C11	25.2(8)	30.3(9)	34.7(8)	-3.4(7)	4.8(7)	4.6(7)
C12	27.7(9)	30.7(9)	42.4(9)	2.9(8)	-0.2(8)	4.4(7)
C13	41.8(12)	30.0(10)	81.8(17)	-12.3(11)	1.5(12)	6.3(9)

Table S26. Bond Lengths for **9.**

Atom	Atom	Length/Å	Atom	Atom	Length/Å
O6	C6	1.4384(19)	C4	C14	1.535(2)
O9	C9	1.437(2)	C9	C10	1.527(2)
O2	C2	1.430(2)	C1	C2	1.524(2)
C7	C8	1.325(2)	C1	C5	1.558(2)
C7	C6	1.505(2)	C1	C11	1.517(3)
C8	C9	1.509(2)	C10	C5	1.539(2)
C8	C15	1.504(2)	C2	C3	1.550(3)
C6	C5	1.550(2)	C11	C12	1.385(3)
C4	C5	1.555(2)	C11	C13	1.444(3)
C4	C3	1.546(2)			

Table S27. Bond Angles for **9.**

Atom	Atom	Atom	Angle/°	Atom	Atom	Atom	Angle/°
C8	C7	C6	125.21(16)	C11	C1	C5	117.99(14)
C7	C8	C9	120.68(15)	C9	C10	C5	115.02(14)
C7	C8	C15	123.14(17)	O2	C2	C1	112.80(15)
C15	C8	C9	116.12(15)	O2	C2	C3	113.03(15)
O6	C6	C7	109.24(13)	C1	C2	C3	104.46(14)
O6	C6	C5	112.95(13)	C6	C5	C4	111.04(13)
C7	C6	C5	111.96(13)	C6	C5	C1	113.23(13)
C3	C4	C5	104.98(13)	C4	C5	C1	101.54(13)
C14	C4	C5	114.22(14)	C10	C5	C6	104.42(13)
C14	C4	C3	109.67(15)	C10	C5	C4	115.73(13)
O9	C9	C8	110.70(14)	C10	C5	C1	111.22(13)
O9	C9	C10	108.88(14)	C4	C3	C2	107.19(14)
C8	C9	C10	112.83(13)	C12	C11	C1	121.73(16)
C2	C1	C5	104.07(13)	C12	C11	C13	121.35(18)
C11	C1	C2	116.03(14)	C13	C11	C1	116.82(17)

Table S28. Hydrogen Bonds for **9.**

D	H	A	d(D-H)/Å	d(H-A)/Å	d(D-A)/Å	D-H-A/°
O1W	H1WA	O6[1]	0.85	2.03	2.7076(11)	135.8
O1W	H1WB	O6[2]	0.85	1.93	2.7076(11)	151.0
O6	H6	O1W	0.82	1.89	2.7076(11)	174.7
O9	H9	O6[3]	0.82	1.91	2.696(2)	159.2

[1]1-X,1-Y,+Z; [2]+Y,+X,1-Z; [3]1/2+Y,3/2-X,+Z

Table S29. Torsion Angles for **9.**

A	B	C	D	Angle/˚	A	B	C	D	Angle/˚
O6	C6	C5	C4	50.05(17)	C2	C1	C11	C12	-37.7(2)
O6	C6	C5	C1	-63.43(17)	C2	C1	C11	C13	138.5(2)
O6	C6	C5	C10	175.44(12)	C5	C4	C3	C2	-13.28(18)
O9	C9	C10	C5	163.59(15)	C5	C1	C2	O2	157.76(14)
O2	C2	C3	C4	-136.17(15)	C5	C1	C2	C3	34.62(16)
C7	C8	C9	O9	-130.53(17)	C5	C1	C11	C12	86.7(2)
C7	C8	C9	C10	-8.2(2)	C5	C1	C11	C13	-97.0(2)
C7	C6	C5	C4	-73.78(17)	C3	C4	C5	C6	-86.87(16)
C7	C6	C5	C1	172.74(13)	C3	C4	C5	C1	33.78(16)
C7	C6	C5	C10	51.61(16)	C3	C4	C5	C10	154.34(14)
C8	C7	C6	O6	-151.74(16)	C15	C8	C9	O9	52.0(2)
C8	C7	C6	C5	-25.9(2)	C15	C8	C9	C10	174.30(15)
C8	C9	C10	C5	40.3(2)	C14	C4	C5	C6	152.96(15)
C6	C7	C8	C9	1.9(3)	C14	C4	C5	C1	-86.39(17)
C6	C7	C8	C15	179.18(16)	C14	C4	C5	C10	34.2(2)
C9	C10	C5	C6	-61.32(17)	C14	C4	C3	C2	109.86(16)
C9	C10	C5	C4	61.0(2)	C11	C1	C2	O2	-70.87(19)
C9	C10	C5	C1	176.22(14)	C11	C1	C2	C3	166.00(14)
C1	C2	C3	C4	-13.18(18)	C11	C1	C5	C6	-53.70(19)
C2	C1	C5	C6	76.51(15)	C11	C1	C5	C4	-172.80(14)
C2	C1	C5	C4	-42.59(15)	C11	C1	C5	C10	63.52(19)
C2	C1	C5	C10	-166.27(13)					

Table S30. Hydrogen Atom Coordinates (Å×10^4) and Isotropic Displacement Parameters (Å^2×10^3) for **9.**

**Atom**	***x***	***y***	***z***	**U(eq)**
H1WA	4575.61	5257.58	5236.64	34
H1WB	5190.56	5344.52	4613.5	34
H6	5932.83	5054.97	5794.62	36
H9	9056.15	7963.6	5839.63	64
H2	6079.59	5666.83	9117.52	59
H7	7406.09	5485.47	4910.51	27
H6A	6323.87	6315.23	5838.79	26
H4	7960.12	5187.82	6947.6	29
H9A	8838.02	6805.98	6418.79	32
H1	6651.54	6677.76	8104.62	30
H10A	7151.43	7492.09	6447.63	30
H10B	7773.2	7360.89	7240.42	30
H2A	5755.65	5211.91	7495.79	32
H3A	7231.6	4892.25	8491.3	35
H3B	6952.45	4424.55	7625.43	35
H15A	9406.67	6169.29	4937.28	45
H15B	8913.5	6861.41	4378.55	45
H15C	8708.6	5873.28	4258.79	45
H14A	8760.91	6267.27	7685.17	51
H14B	8661.38	5453.21	8286.93	51
H14C	8078.1	6271.22	8439.94	51
H12A	4427.94	6985.57	6805.59	40
H12B	4890.67	6050.99	6753.51	40
H13A	5573.87	7887.54	8244.64	77
H13B	5197.86	8175.3	7342.91	77
H13C	6202.81	8097.52	7472.37	77

Figure S167. ORTEP drawing of **14.**

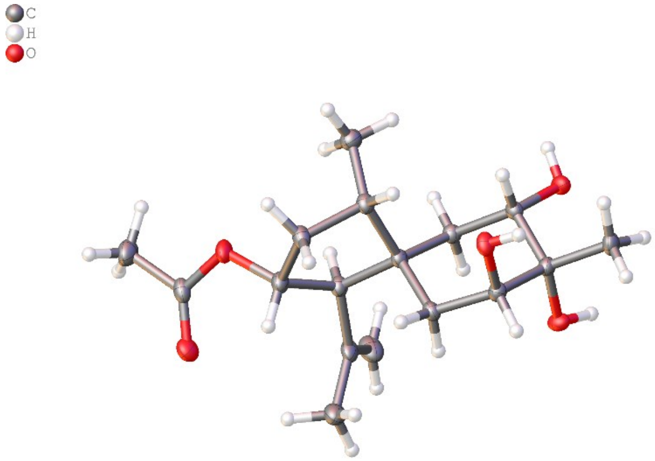

Table S31. Crystal data and structure refinement for **14.**

Identification code	54243
Empirical formula	$C_{17}H_{28}O_5$
Formula weight	312.39
Temperature/K	100.01(10)
Crystal system	orthorhombic
Space group	$P2_12_12$
a/Å	14.8206(3)
b/Å	11.2138(2)
c/Å	10.2074(2)
α/°	90
β/°	90
γ/°	90
Volume/Å^3	1696.42(6)
Z	4
ρ_{calc}g/cm^3	1.223
μ/mm^{-1}	0.723
F(000)	680.0
Crystal size/mm^3	0.22 × 0.13 × 0.05
Radiation	Cu $K\alpha$ (λ = 1.54184)
2Θ range for data collection/°	8.662 to 150.378
Index ranges	$-18 \le h \le 16$, $-14 \le k \le 10$, $-12 \le l \le 12$
Reflections collected	10268
Independent reflections	3404 [R_{int} = 0.0515, R_{sigma} = 0.0525]
Data/restraints/parameters	3404/0/216
Goodness-of-fit on F^2	1.046
Final R indexes [I>=2σ (I)]	R_1 = 0.0383, wR_2 = 0.0991
Final R indexes [all data]	R_1 = 0.0401, wR_2 = 0.1008
Largest diff. peak/hole / e Å^{-3}	0.24/-0.20
Flack parameter	-0.03(11)

Table S32. Fractional Atomic Coordinates (×10^4) and Equivalent Isotropic Displacement Parameters (Å^2×10^3) for **14**. U_{eq} is defined as 1/3 of of the trace of the orthogonalised U_{IJ} tensor.

**Atom**	***x***	***y***	***z***	**U(eq)**
O7	2625.7(10)	1865.0(13)	10496.9(14)	19.6(3)
O8	4789.9(9)	3141.1(13)	9613.4(15)	20.2(3)
O9	3773.3(9)	5330.7(12)	10094.9(14)	18.4(3)
O2	1491.1(9)	2749.0(14)	4791.0(13)	22.0(3)
O16	2327.0(11)	1904.4(14)	3209.7(14)	25.6(3)
C9	3363.7(13)	4233.2(16)	9691.6(19)	15.2(4)
C5	2814.4(13)	3027.5(17)	7735.9(18)	15.9(4)
C6	3308.2(13)	1949.2(16)	8351.6(18)	16.5(4)
C10	3288.2(13)	4163.8(17)	8206.3(19)	16.4(4)
C16	1711.0(15)	2547.5(19)	3541(2)	21.0(4)
C8	3927.9(12)	3199.5(17)	10230.4(19)	16.1(4)
C4	1778.1(13)	2974.1(17)	8013.6(18)	17.4(4)
C3	1448.0(14)	2032.8(19)	7032.3(19)	20.8(4)
C11	3730.8(14)	2756(2)	5549.8(19)	21.4(4)
C7	3469.6(12)	2025.0(17)	9834.0(19)	16.6(4)
C14	1280.9(15)	4162.2(19)	7820(2)	23.2(4)
C2	2036.1(13)	2165.1(19)	5806.8(19)	19.0(4)
C15	4044.7(14)	3300.2(18)	11710(2)	21.0(4)
C1	2825.9(13)	2985.0(18)	6195.7(18)	17.4(4)
C12	4256.1(16)	3693(2)	5232(2)	31.0(5)
C13	4016.4(15)	1518(2)	5241(2)	27.7(5)
C17	1109.3(16)	3210(2)	2608(2)	30.0(5)

Table S33. Anisotropic Displacement Parameters ($Å^2 \times 10^3$) for **14**. The Anisotropic displacement factor exponent takes the form: $-2\pi^2[h^2a^{*2}U_{11}+2hka^*b^*U_{12}+\ldots]$.

**Atom**	U_{11}	U_{22}	U_{33}	U_{23}	U_{13}	U_{12}
O7	20.5(7)	25.5(7)	12.8(7)	1.4(6)	0.2(5)	-5.6(6)
O8	14.3(6)	19.8(7)	26.5(8)	-3.4(6)	2.0(5)	0.4(6)
O9	17.0(7)	15.8(6)	22.5(7)	-4.2(5)	-1.8(6)	-0.8(5)
O2	19.3(7)	34.0(8)	12.7(7)	-2.2(6)	-1.5(5)	-0.8(6)
O16	28.3(8)	32.6(8)	15.9(7)	-2.5(6)	2.1(6)	1.3(7)
C9	15.2(8)	15.2(8)	15.3(9)	-1.6(7)	0.6(7)	-1.9(7)
C5	17.7(9)	18.0(9)	11.9(8)	-0.5(7)	0.1(7)	-0.3(7)
C6	17.6(9)	17.4(8)	14.6(9)	-2.6(7)	0.5(7)	-2.2(7)
C10	16.8(9)	17.6(8)	14.8(9)	0.5(7)	0.7(7)	-1.0(7)
C16	21.2(10)	27.5(10)	14.3(9)	-1.3(8)	-0.9(8)	-7.7(8)
C8	14.4(8)	19.7(9)	14.4(9)	-0.5(7)	-0.5(7)	-0.1(7)
C4	17.1(9)	21.5(9)	13.7(8)	-0.4(7)	1.0(7)	-2.3(7)
C3	19.4(9)	27.7(10)	15.4(9)	-2.9(8)	1.1(7)	-6.3(8)
C11	19.0(9)	33.8(11)	11.5(8)	-3.5(7)	0.3(7)	-1.8(8)
C7	16.7(9)	16.9(8)	16.2(9)	0.9(7)	-1.0(7)	-0.5(7)
C14	20.8(10)	26.8(10)	21.9(10)	-0.2(8)	-0.1(9)	2.4(8)
C2	17.7(9)	25.5(10)	13.6(9)	-0.6(7)	-1.0(7)	-1.5(8)
C15	24.1(10)	23.1(9)	15.7(9)	-0.3(8)	-5.1(8)	-0.6(8)
C1	16.6(9)	22.0(9)	13.5(9)	-0.3(7)	0.0(7)	-2.5(7)
C12	26.8(11)	42.5(13)	23.6(11)	-6.6(10)	7.8(9)	-7.1(9)
C13	22.9(10)	38.7(12)	21.6(10)	-7.1(9)	1.1(9)	3.4(9)
C17	26.8(11)	45.8(13)	17.4(10)	-0.9(10)	-2.8(8)	3.1(10)

Table S34. Bond Lengths for **14.**

Atom	Atom	Length/Å	Atom	Atom	Length/Å
O7	C7	1.433(2)	C6	C7	1.534(3)
O8	C8	1.426(2)	C16	C17	1.501(3)
O9	C9	1.433(2)	C8	C7	1.536(2)
O2	C16	1.337(3)	C8	C15	1.525(3)
O2	C2	1.468(2)	C4	C3	1.535(3)
O16	C16	1.211(3)	C4	C14	1.535(3)
C9	C10	1.522(3)	C3	C2	1.532(3)
C9	C8	1.531(3)	C11	C1	1.516(3)
C5	C6	1.547(3)	C11	C12	1.347(3)
C5	C10	1.532(3)	C11	C13	1.486(3)
C5	C4	1.563(3)	C2	C1	1.540(3)
C5	C1	1.573(3)			

Table S35. Bond Angles for **14.**

Atom	Atom	Atom	Angle/°	Atom	Atom	Atom	Angle/°
C16	O2	C2	117.69(16)	C15	C8	C9	111.21(16)
O9	C9	C10	111.17(15)	C15	C8	C7	112.01(15)
O9	C9	C8	108.40(15)	C3	C4	C5	102.78(15)
C10	C9	C8	111.05(16)	C3	C4	C14	111.09(17)
C6	C5	C4	111.18(16)	C14	C4	C5	114.53(16)
C6	C5	C1	112.16(16)	C2	C3	C4	106.55(16)
C10	C5	C6	107.81(15)	C12	C11	C1	118.9(2)
C10	C5	C4	115.18(16)	C12	C11	C13	120.9(2)
C10	C5	C1	109.48(16)	C13	C11	C1	120.15(19)
C4	C5	C1	101.00(14)	O7	C7	C6	108.82(15)
C7	C6	C5	115.54(15)	O7	C7	C8	111.64(15)
C9	C10	C5	112.85(15)	C6	C7	C8	112.10(15)
O2	C16	C17	112.16(19)	O2	C2	C3	107.86(15)
O16	C16	O2	123.4(2)	O2	C2	C1	109.50(16)
O16	C16	C17	124.4(2)	C3	C2	C1	106.24(16)
O8	C8	C9	111.43(16)	C11	C1	C5	116.70(16)
O8	C8	C7	103.91(15)	C11	C1	C2	117.32(16)
O8	C8	C15	109.84(15)	C2	C1	C5	105.51(15)
C9	C8	C7	108.24(15)				

Table S36. Hydrogen Bonds for **14.**

D	H	A	d(D-H)/Å	d(H-A)/Å	d(D-A)/Å	D-H-A/°
O8	H8	O9[1]	0.93(4)	1.89(4)	2.777(2)	158(3)
O9	H9	O7[2]	0.83(4)	1.95(4)	2.761(2)	167(3)
O7	H7	O16[3]	0.79(4)	2.06(4)	2.805(2)	157(3)

[1]1-X,1-Y,+Z; [2]1/2-X,1/2+Y,2-Z; [3]+X,+Y,1+Z

Table S37. Torsion Angles for **14.**

A	B	C	D	Angle/°	A	B	C	D	Angle/°
O8	C8	C7	O7	173.23(15)	C10	C5	C1	C2	-157.01(16)
O8	C8	C7	C6	-64.38(19)	C16	O2	C2	C3	-154.34(17)
O9	C9	C10	C5	-177.40(15)	C16	O2	C2	C1	90.5(2)
O9	C9	C8	O8	-68.16(19)	C8	C9	C10	C5	61.8(2)
O9	C9	C8	C7	178.18(15)	C4	C5	C6	C7	-77.7(2)
O9	C9	C8	C15	54.7(2)	C4	C5	C10	C9	71.0(2)
O2	C2	C1	C5	131.09(16)	C4	C5	C1	C11	-167.39(17)
O2	C2	C1	C11	-97.0(2)	C4	C5	C1	C2	-35.11(19)
C9	C8	C7	O7	-68.22(18)	C4	C3	C2	O2	-105.39(18)
C9	C8	C7	C6	54.2(2)	C4	C3	C2	C1	11.9(2)
C5	C6	C7	O7	72.1(2)	C3	C2	C1	C5	14.8(2)
C5	C6	C7	C8	-51.9(2)	C3	C2	C1	C11	146.78(17)
C5	C4	C3	C2	-34.1(2)	C14	C4	C3	C2	88.83(19)
C6	C5	C10	C9	-53.8(2)	C2	O2	C16	O16	0.7(3)
C6	C5	C4	C3	-77.20(18)	C2	O2	C16	C17	-179.22(17)
C6	C5	C4	C14	162.20(16)	C15	C8	C7	O7	54.7(2)
C6	C5	C1	C11	-48.9(2)	C15	C8	C7	C6	177.12(16)
C6	C5	C1	C2	83.36(19)	C1	C5	C6	C7	170.05(15)
C10	C9	C8	O8	54.2(2)	C1	C5	C10	C9	-176.06(16)
C10	C9	C8	C7	-59.41(19)	C1	C5	C4	C3	41.97(18)
C10	C9	C8	C15	177.15(16)	C1	C5	C4	C14	-78.6(2)
C10	C5	C6	C7	49.4(2)	C12	C11	C1	C5	-90.4(2)
C10	C5	C4	C3	159.80(16)	C12	C11	C1	C2	142.9(2)
C10	C5	C4	C14	39.2(2)	C13	C11	C1	C5	91.3(2)
C10	C5	C1	C11	70.7(2)	C13	C11	C1	C2	-35.3(3)

Table S38. Hydrogen Atom Coordinates (Å×10^4) and Isotropic Displacement Parameters (Å^2×10^3) for **14.**

Atom	*x*	*y*	*z*	U(eq)
H9A	2756.54	4187.69	10068.62	18
H6A	2959.42	1235.88	8171.64	20
H6B	3887.39	1859.35	7920.16	20
H10A	3888.51	4194.77	7829.34	20
H10B	2956.2	4852.25	7892.2	20
H4	1676.79	2685.9	8908	21
H3A	1510.94	1238.99	7398.52	25
H3B	818.18	2164.75	6818.21	25
H7A	3869.1	1366.99	10084.4	20
H14A	1499	4734.52	8444.09	35
H14B	645.38	4043.57	7947.7	35
H14C	1387.27	4451.41	6948.86	35
H2	2256.16	1387.67	5506.29	23
H15A	4294.27	2571.34	12045.09	31
H15B	3468.7	3443.27	12110.86	31
H15C	4444.65	3949.4	11906.93	31
H1	2647.27	3785.62	5911.49	21
H12A	4805.7	3568.63	4814.36	37
H12B	4069.17	4463.42	5431.1	37
H13A	3930.2	1021.65	5997.65	42
H13B	4642.29	1514.45	4999.31	42
H13C	3661.14	1216.56	4527.85	42
H17A	656.6	2678.22	2273.99	45
H17B	1462.75	3514.84	1894.62	45
H17C	823.89	3859.46	3058.65	45
H8	5160(20)	3790(30)	9840(40)	49(9)
H9	3390(20)	5860(30)	10010(30)	41(8)
H7	2700(20)	1860(30)	11260(30)	38(8)

Fig. S169. Comparison of the ^{1}H NMR spectrum of the hydrolyzed product of **9** to that of **8**

A. ^{1}H NMR spectrum of the hydrolyzed product of **9** in DMSO-d6

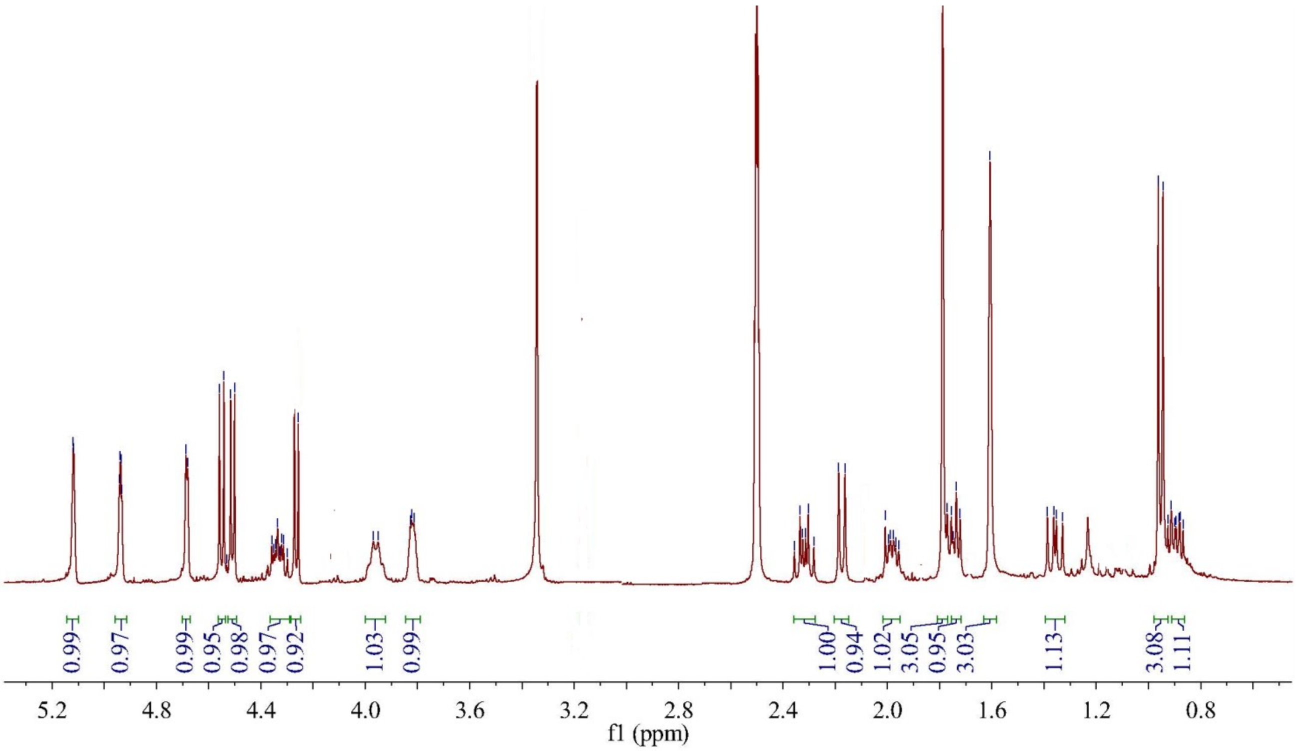

B. ^{1}H NMR spectrum of **8** in DMSO-d6

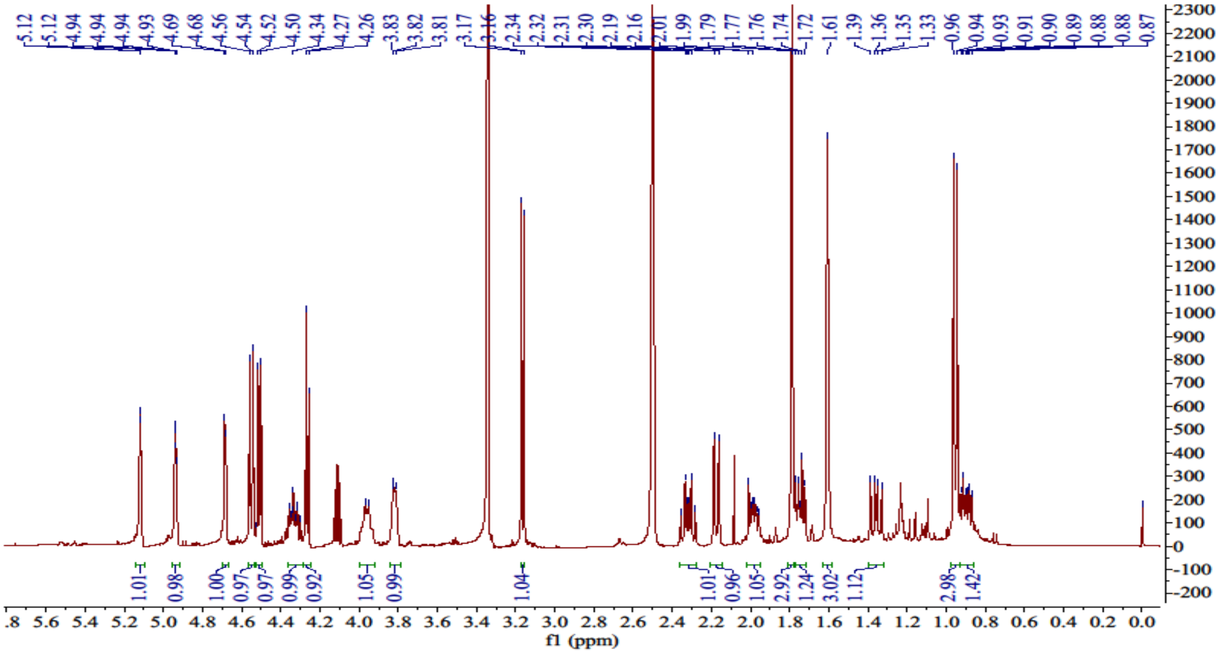

Fig. S170. Comparison of the ^{1}H NMR spectrum of the hydrolyzed product of **13** to that of **14**

A. ^{1}H NMR spectrum of the hydrolyzed product of **13** in DMSO-d6

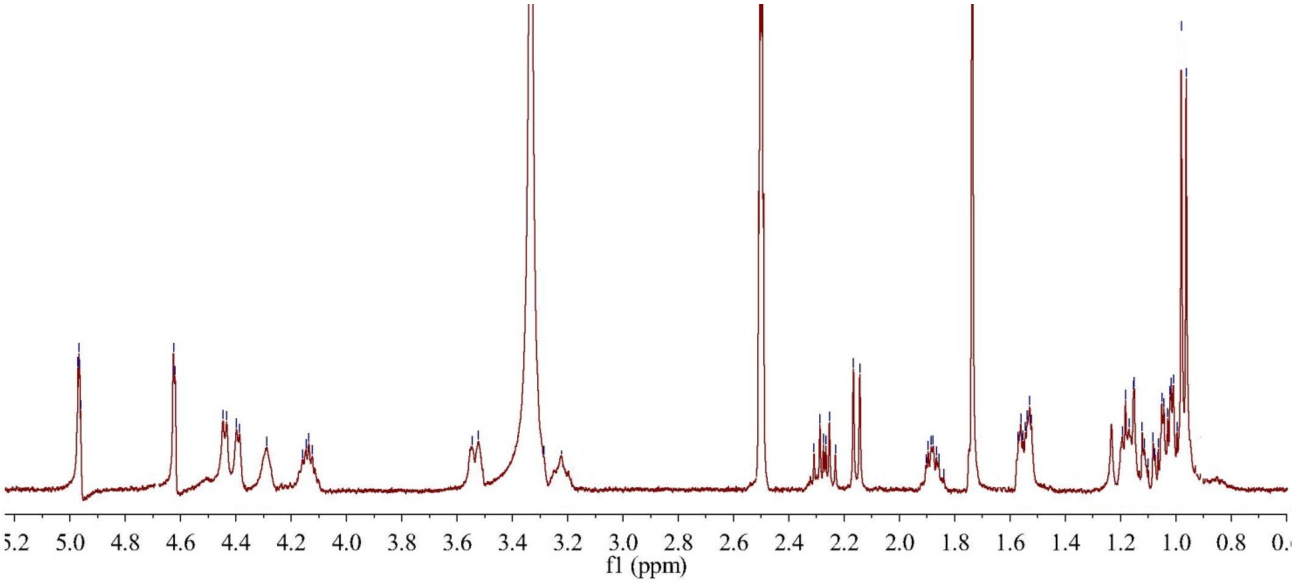

B. ^{1}H NMR spectrum of **14** in DMSO-d6

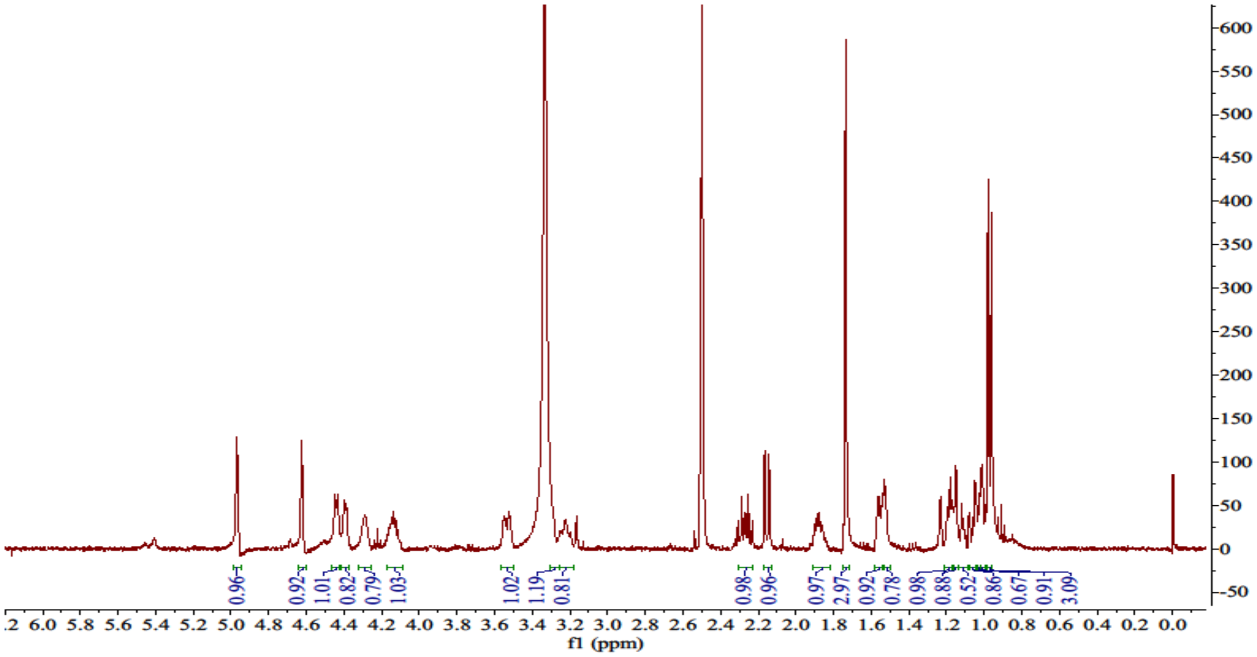

Fig. S171. Comparison of the ^{1}H NMR spectrum of the hydrolyzed product of **15** to that of **16**

A. ^{1}H NMR spectrum of the hydrolyzed product of **15** in DMSO-d6

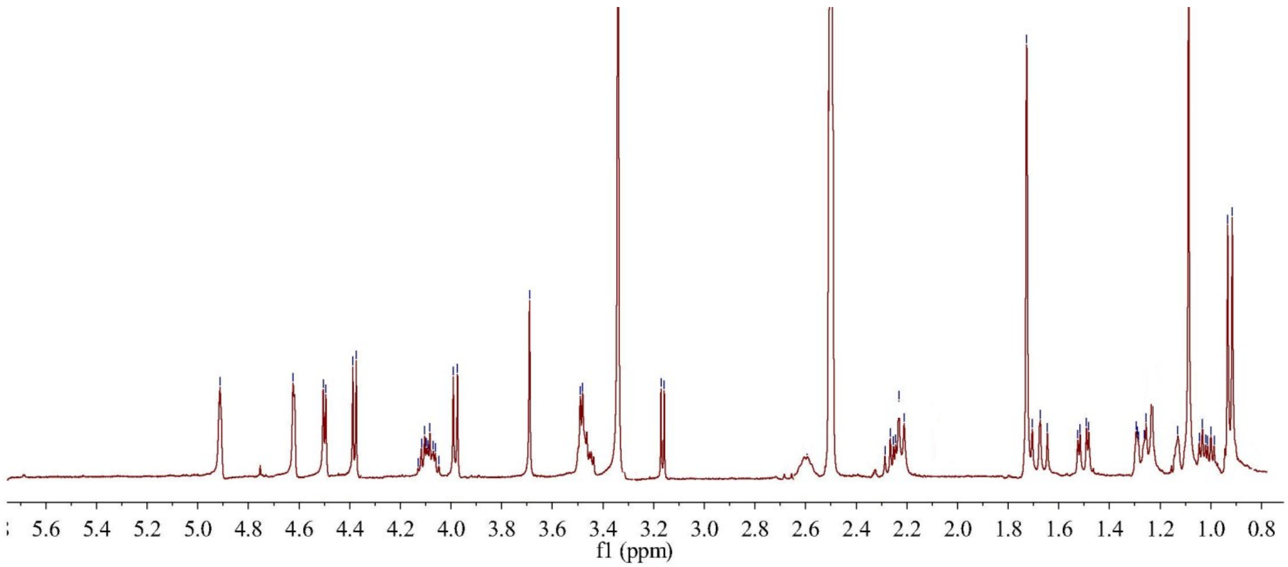

B. ^{1}H NMR spectrum of **16** in DMSO-d6

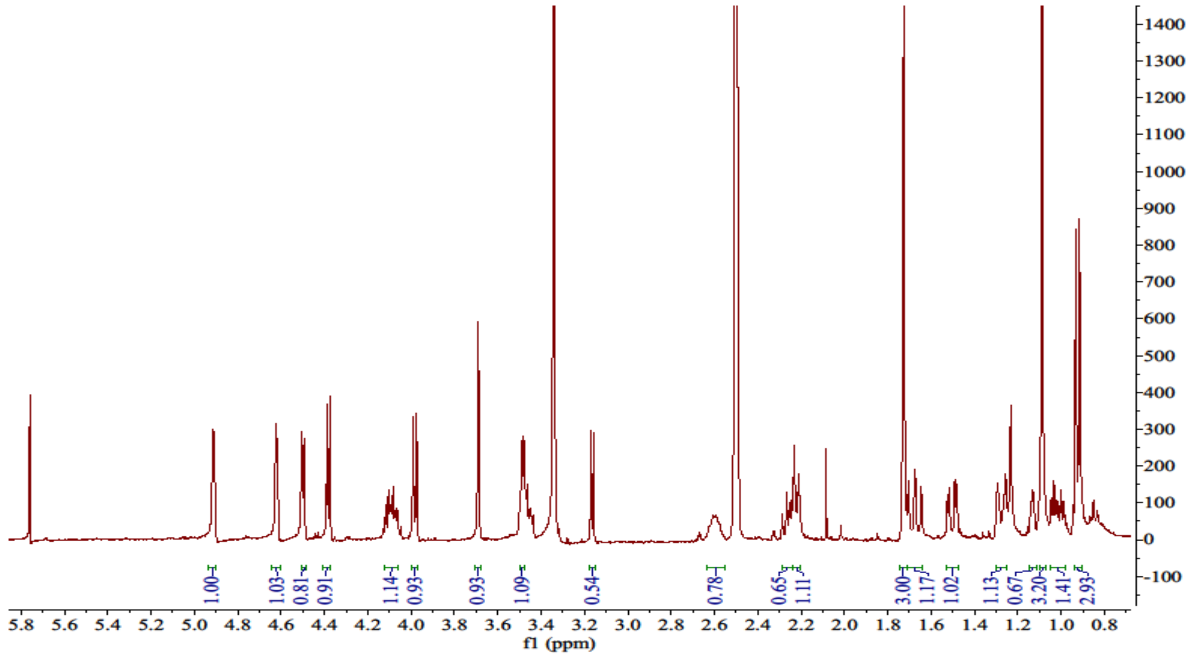

Fig. S172. Comparison of the ^{1}H NMR spectrum of the hydrolyzed product of adametacorenol A to that of **5**

A. ^{1}H NMR spectrum of the hydrolyzed product of adametacorenol A in DMSO-d6

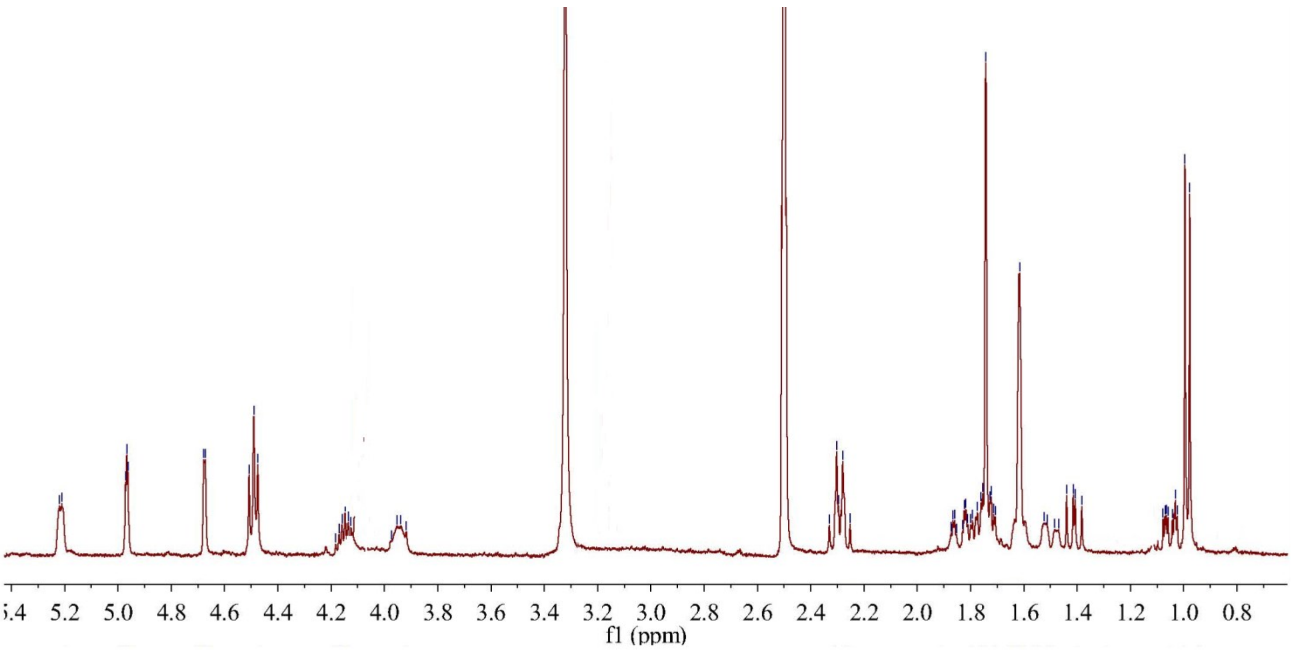

B. ^{1}H NMR spectrum of **5** in DMSO-d6

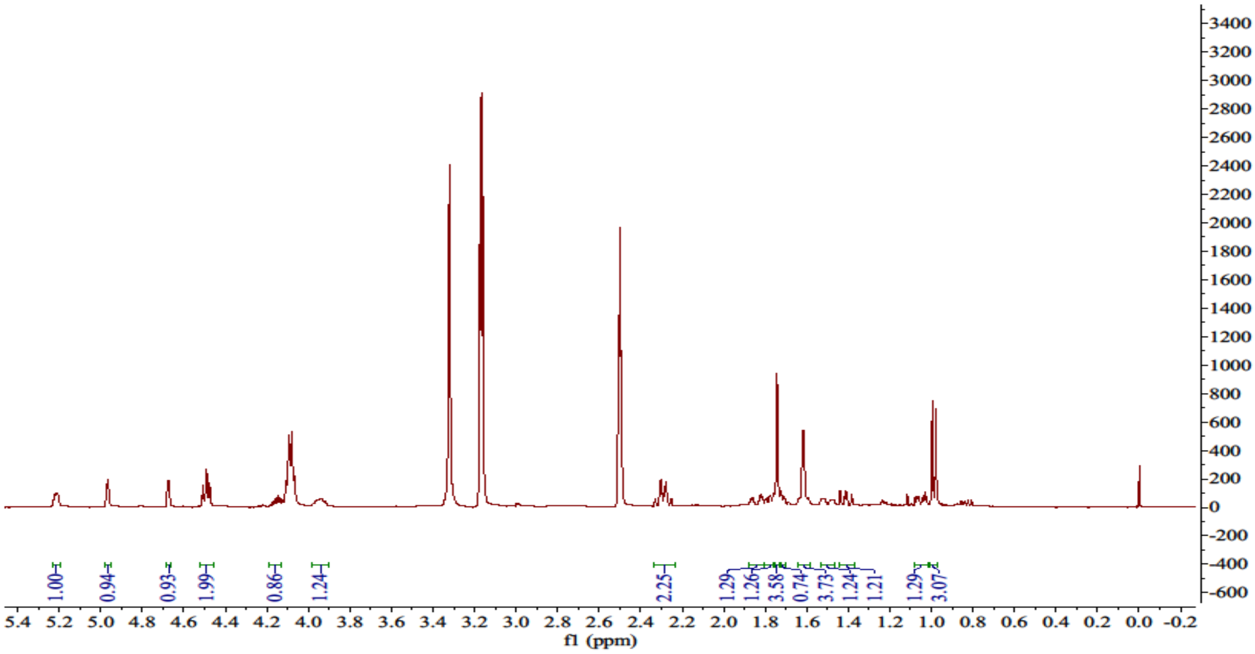

Fig. S173. Comparison of the ^{1}H NMR spectrum of the hydrolyzed product of adametacorenol B to that of **7**

A. ^{1}H NMR spectrum of the hydrolyzed product of adametacorenol B in DMSO-d6

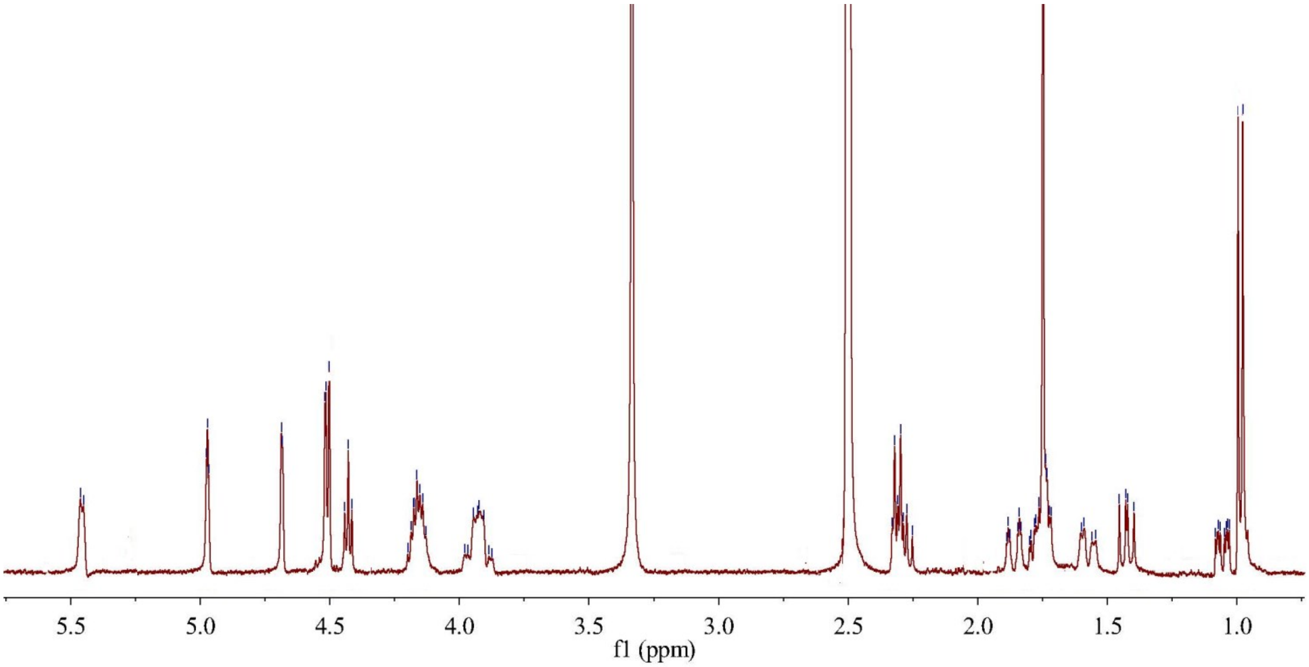

B. ^{1}H NMR spectrum of **7** in DMSO-d6

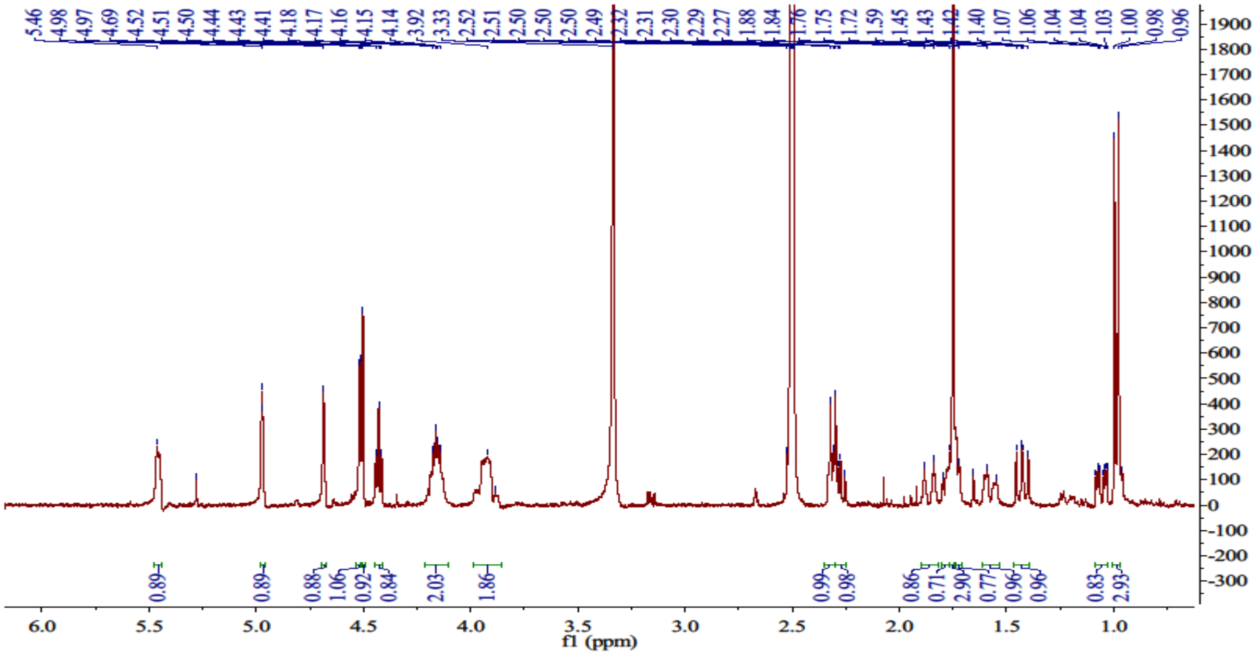

Supplement: Supplementary file 12 [file DataSheet1.PDF]
